# Tristetraprolin overexpression drives hematopoietic changes in young and middle-aged mice generating dominant mitigating effects on induced inflammation in murine models

**DOI:** 10.1007/s11357-023-00879-2

**Published:** 2023-08-03

**Authors:** Mayuri Tanaka-Yano, Le Zong, Bongsoo Park, Hagai Yanai, Ferda Tekin-Turhan, Perry J. Blackshear, Isabel Beerman

**Affiliations:** 1 Epigenetics and Stem Cell Unit, Translational Gerontology Branch, National Institute On Aging, National Institutes of Health, 251 Bayview Boulevard, Baltimore, MD 21224 USA; 2 Signal Transduction Laboratory, National Institute of Environmental Health Sciences, Research Triangle Park, Durham, NC 27709 USA

**Keywords:** Tristetraprolin, Hematopoietic system, Overexpression

## Abstract

Tristetraprolin (TTP), encoded by *Zfp36* in mice, is one of the best-characterized tandem zinc-finger mRNA binding proteins involved in mRNA deadenylation and decay. TTPΔARE mice lack an AU-rich motif in the 3′-untranslated regions of TTP mRNA, leading to increased TTP mRNA stability and more TTP protein, resulting in elevated mRNA decay rates of TTP targets. We examined the effect of TTP overexpression on the hematopoietic system in both young and middle-aged mice using TTPΔARE mice and found alterations in blood cell frequencies, with loss of platelets and B220 cells and gains of eosinophils and T cells. TTPΔARE mice also have skewed primitive populations in the bone marrow, with increases in myeloid-biased hematopoietic stem cells (HSCs) but decreases in granulocyte/macrophage-biased multipotent progenitors (MPP3) in both young and middle-aged mice. Changes in the primitive cells’ frequencies were associated with transcriptional alterations in the TTP overexpression cells specific to age as well as cell type. Regardless of age, there was a consistent elevation of transcripts regulated by TNFα and TGFβ signaling pathways in both the stem and multipotent progenitor populations. HSCs with TTP overexpression had decreased reconstitution potential in murine transplants but generated hematopoietic environments that mitigated the inflammatory response to the collagen antibody-induced arthritis (CAIA) challenge, which models rheumatoid arthritis and other autoimmune disorders. This dampening of the inflammatory response was even present when there was only a small frequency of TTP overexpressing cells present in the middle-aged mice. We provide an analysis of the early hematopoietic compartments with elevated TTP expression in both young and middle-aged mice which inhibits the reconstitution potential of the HSCs but generates a hematopoietic system that provides dominant repression of induced inflammation.

**Supplementary Information:**

The online version contains supplementary material available at 10.1007/s11357-023-00879-2.

## Introduction

*Zfp36* family members encode zinc finger binding proteins that regulate gene expression by altering mRNA stability. In the mouse, there are four family members: *Zfp36*, which encodes TTP, the focus of this manuscript, *Zfp36L1*, *Zfp36L2*, and *Zfp36L3* [1, 2]. These proteins contain tandem CCCH-type zinc-finger motifs that bind to AU-rich elements (ARE) that reside largely in 3′-untranslated regions of target mRNAs [3]. mRNA with ARE regions bound by TTP are directed towards degradation. Targets of ZPF36 or TTP include transcripts encoding inflammatory cytokines and chemokines, such as *Tnf*, *Nfkb1*, and *Ifng* [4–6], and TTP also self-regulates by binding to its own ARE domains to promote its own mRNA decay.

Studies of *Zfp36* in the hematopoietic system, using a model with complete “whole body” loss of TTP, demonstrate increased granulocyte frequency and decreased B lymphopoiesis [7], associated with increased frequencies of granulocyte and monocyte progenitors (GMPs) and multipotent progenitor populations (MPPs) [8]. Decreases in *Zfp36* expression levels have been associated with myeloid disorders, and aberrant expression of other *Zfp36* family members, *Zfp36l1* and *Zfp36l2*, has been associated with various leukemias [9, 10]. Recently, it was determined that ZFP36 also plays a role in regulating T cell homeostasis and autoimmunity [11].

The aging hematopoietic system endures significant alterations, including peripheral blood cell composition changes, increased frequency of hematological diseases, and mitigated immune responses. Perturbed functional hematopoiesis and blood cell composition in rheumatoid arthritis (RA) resemble those seen in the elderly [12], yet the relative contributions of the bone marrow (BM) environment, cell-intrinsic processes, and systemic factors to the aging process in hematopoietic stem cells (HSCs) in RA are still unclear.

We have previously demonstrated that increased stability of TTP can confer protection against inflammatory responses in various mouse models of human disease, including RA, psoriasis, multiple sclerosis, and others [2, 13] but it is unclear how the increased TTP affects the early hematopoietic progenitor compartment, and whether the mitigation of inflammation is exclusively driven by the hematopoietic compartment. Using the TTPΔARE mouse model, we examined the effects of overexpression of this mRNA decay regulator on the hematopoietic system, with a focus on primitive BM populations, and determine that even low frequencies of hematopoietic cells overexpressing TTP will exert a dominant effect of mitigating inflammatory responses in collagen antibody-induced arthritis (CAIA).

## Results

### Blood composition alterations at steady state in young and middle-aged mice with TTP overexpression

**Fig. 1:**
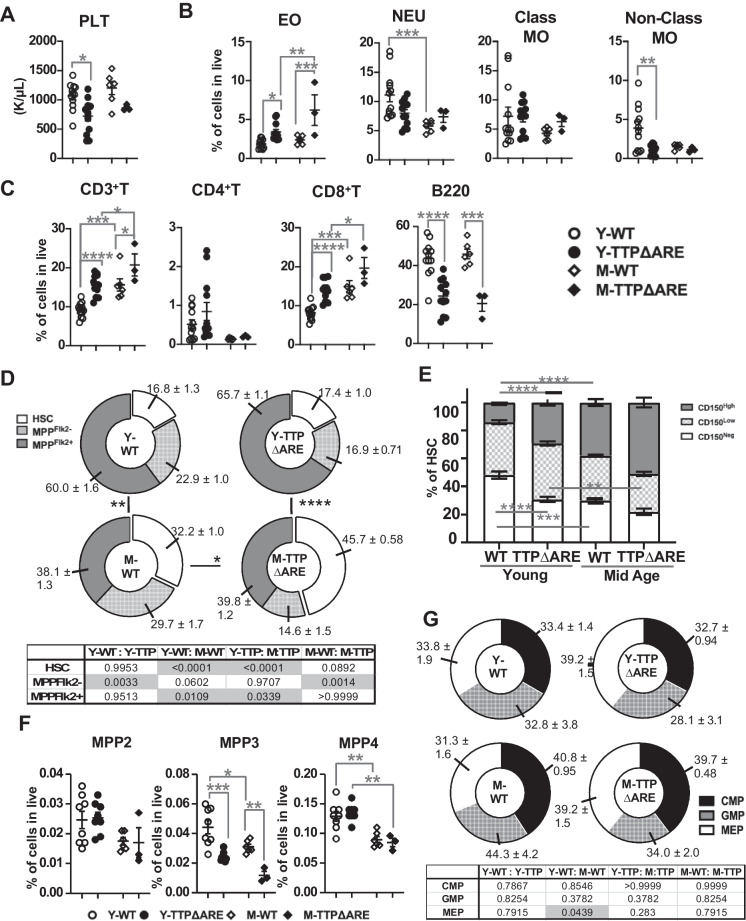
Analysis of blood and bone marrow in young and middle-aged WT and TTPΔARE mice. **A** Platelet (Plt) counts from a complete blood cell analysis. **B**, **C** Flow cytometry analysis plotted as the frequency of live cells for **B** myeloid cells: eosinophils (EO), neutrophils (NEU), classical monocytes (class MO) and non-classical monocytes (non-class MO), and **C** lymphoid cells: CD3^+^T, CD4^+^T, CD8^+^T, and B220^+^ cells. **D** Composition of the LSK compartment within the bone marrow including HSC, MPP^Flk2−^, and MPP^Flk2+^ cells. **E** Frequency of lineage-biased HSCs, including CD150^Neg^, CD150^Low^, and CD150.^High^ cells. **F** Frequency of multipotent progenitor populations MPP2, MPP3, and MPP4 in live cells. **G** Composition of myeloid progenitors (MPs), including common myeloid progenitors (CMPs), granulocyte-myeloid progenitors (GMPs), megakaryocyte-erythroid progenitors (MEPs), and others. Data were pooled from two to four separate experiments: blood analysis: Y-WT = 12, Y-TTPΔARE = 12, M-WT = 6, and M-TTPΔARE = 3; bone marrow analysis: Y-WT = 16, Y-TTPΔARE = 16, M-WT = 6, and M-TTPΔARE = 3. Results are expressed as mean ± SEM. *P < 0.05; **P < 0.01; ***P < 0.001; ****P < 0.0001, between indicated groups, as calculated by chi-squared test (**D**, **G**) and one-way ANOVA with Tukey’s multiple comparisons test (**A**–**G**)

To examine if systemic alterations occurred in the adult hematopoietic system of mice overexpressing the TTP protein, we evaluated blood composition changes in homozygous young-adult mice (Y-TTPΔARE) and their wild-type littermates (Y-WT). Given age-associated increases in inflammation and the role of TTP in mediating the decay of several inflammatory cytokines [14–16], we also examined the blood cell compositions from 1-year-old (middle-aged) TTPΔARE and WT littermates (M-TTPΔARE and M-WT). Complete blood counts showed no significant alterations in the total number of white or red blood cells in the TTPΔARE mice (data not shown), but young TTPΔARE mice had significantly decreased platelet counts (Fig. 1A). We also performed flow cytometry to define frequencies of distinct leukocyte populations in peripheral blood (Supplemental Fig. 1A) and found increased levels of eosinophils and CD3^+^ T cells (Fig. 1B, C), and a loss of B-cells, associated with overexpression of TPP in both young and middle-aged mice (Fig. 1B, C). Loss of non-classical monocytes and increased CD8^+^ T cells was exclusive to the overexpression of TTP in young mice compared to their wild-type littermates. The age-associated significant differences in the T cell populations seen in the WT mice were maintained in the comparisons between the TTPΔARE young and middle-aged mice (Fig. 1C), but the age-associated loss of WT neutrophils was not significant in the TTPΔARE comparison (Fig. 1B).

### Myeloid-biased HSCs accumulate in TTPΔARE mice

To explore if the changes in the blood population frequencies of the TTPΔARE mice could be driven by alterations in the TTP overexpressing progenitor bone marrow (BM) populations, we analyzed fifteen stem and progenitor populations (Supplemental Fig. 1B). Within the Lineage^−^Sca1^+^cKit^+^ (LSK) population, containing the most primitive stem cells and MPPs, there were significant changes in the HSC and Flk2^+^ multipotent progenitor (MPP^Flk2^) compartments driven by age, but these were not affected by TTP overexpression. There was a significant decrease in frequency of the MPP^Flk2−^population in TTP overexpressing mouse bone marrow in both age groups (Fig. 1D). Age-associated differences were seen in the LSK compartment in the WT, and TTP overexpression also led to significant differences in the overall composition of the LSK compartment in the middle-aged mice (Fig. 1D). To more fully characterize the stem cell compartment, we used CD150 expression levels to evaluate whether TTP overexpression alters the frequency of the lineage-biased subsets of the HSCs [17]. We found that in young mice, elevated TTP led to increased myeloid-biased HSCs (CD150^high^) and decreased CD150^Neg^ (multipotent progenitor with lymphoid bias) (Fig. 1E). In concordance with previously reported age-associated changes, the middle-aged WT gain CD150^High^ and lose CD150^Neg^ percentages, but TTP overexpression in middle-aged mice did not further exacerbate these changes (Fig. 1E). Using an alternative gating strategy that sub-fractionates MPP [18], we established that the MPP2 and MPP4 populations were not significantly affected by the overexpression of TTP, but that the MPP3 population (with biased granulocyte/monocyte production and limited lymphoid potential) was significantly reduced in both the young and middle-aged TTPΔARE mice (Fig. 1F). However, the increase in myeloid-biased HSCs and reduction of MPP3s driven by TTP overexpression did not lead to altered frequencies of common myeloid progenitors (CMP), granulocyte-monocyte progenitors (GMP), or megakaryocyte-erythrocyte progenitors (MEPs, Fig. 1G), although a significant decrease in megakaryocyte progenitors (MkPs) was observed (Supplemental Fig. 2).

### Transcriptional changes driven by TTP overexpression are cell type- and age-specific in hematopoietic progenitors

**Fig. 2:**
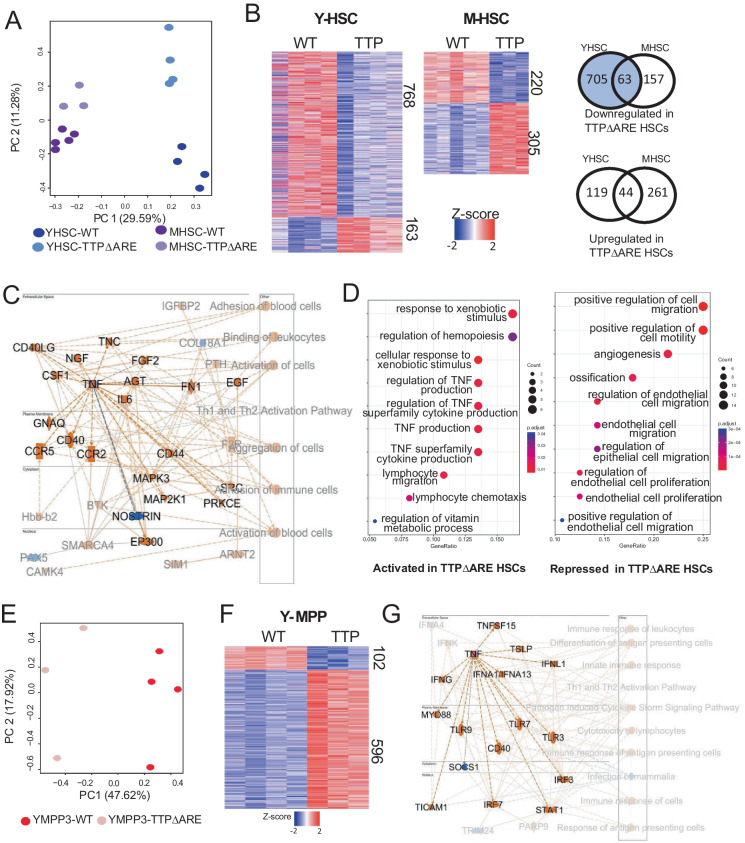
Transcript analysis of HSCs and MPP3s from young and middle-aged WT and TTPΔARE mice. **A** PCA of HSCs isolated from young and middle-aged WT and TTPΔARE mice: Y-WT = 4, Y-TTPΔARE = 4, M-WT = 5, and M-TTPΔARE = 3. **B** Heatmaps of differentially expressed transcripts (DET) between WT and TTPΔARE HSCs and the overlap seen in down- and up-regulated genes between young and middle age. **C** IPA graphical overview of predicted pathways that are activated (orange) or repressed (blue) in young TTPΔARE HSCs. **D** GO pathway enrichment of overlapping DETs between young and middle-aged TTPΔARE HSCs. **E** PCA of MPP3 cells isolated from young WT and TTPΔARE mice: Y-WT = 4, Y-TTPΔARE = 3. **F** Heatmaps of differentially expressed transcripts (DET) between young WT and TTPΔARE MPP3 cells. **G** IPA graphical overview of predicted pathways that are activated (orange) or repressed (blue) in young TTPΔARE MPP3s. More complete legends for IPA can be found in Supplemental Fig. 3A

Given the changes in the composition of the HSC compartment (increased myeloid-biased HSCs) and significant loss of myeloid-biased MPPs (MPP3), we sought to examine how TTP overexpression might specifically affect the transcriptional profiles of these cells. We first performed the bulk mRNA-seq on HSCs (LSKCD34^−^Flk2^−^CD150^+^) isolated from young and middle-aged TTPΔARE and age-matched WT mice (Y-WT = 4; Y-TTPΔARE = 4; M-WT = 5; M-TTPΔARE = 3). PCA of global transcripts showed that the biggest driver of variation was age, with PC1 separating the HSCs by donor age, and TTP expression drove the separation on the PC2 axis (Fig. 2A). Interestingly, TTP-driven changes in transcript levels were more pronounced in the young TTP overexpressing HSCs (Y-TTPΔARE vs Y-WT), compared to the changes seen in the middle-aged mice. Examining differentially expressed genes (DEGs) between TTPΔARE and WT Y-HSCs, we found that TTP overexpressing Y-HSCs contained 768 transcripts that were decreased in expression and 163 that were increased (Fig. 2B, Supplemental Table 1). Fewer total differentially expressed genes were found between the middle-aged HSCs (220 down, 305 up: Supplemental Table 1). As an internal validation, *Zfp36* mRNA was one of the transcripts with significantly increased expression in both comparisons, with levels increased by 1.97 and 2.01 log-fold in young and middle-aged TTPΔARE mice, respectively. The levels of transcripts encoding other TTP family members were not significantly changed (Supplemental Table 1, Supplemental Fig. 3A).

Given the overlap in some features of aging, such as decreased B220 and increased CD150^high^ populations, we explored if overexpression of TTP led to age-associated expression changes. To identify genes with altered expression associated with aging, we used the HSC aging signature website [19] and selected transcripts with altered expression in aged HSCs compared to young HSCs reported in at least 3 published datasets and found 431 transcripts. Of these, 57 were also significantly changed in young TTP overexpressing HSCs compared to young WT HSC; however, only 18 of those 57 genes changed in the same direction as during aging (16 up in both Y-TTP HSC and aged HSC, 2 down in both Y-TTP HSC and aged HSC) (Supplemental Table 2, Supplemental Fig. 3B). Middle-aged TTPΔARE HSC compared to middle-aged WT HSCs had 77 of the 431 age-associated HSC genes with significant differences, with 36 in the same direction (32 up in both M-TTP HSC and aged HSC, 4 down in both M-TTP HSC and aged HSC) (Supplemental Table 2, Supplemental Fig. 3B). Thus, there does not appear to be a direct correlation between aging signatures and TTP overexpression.

To explore which pathways were altered in the setting of overexpression of TTP in the hematopoietic system, we performed IPA (QIAGEN) on DEGs from the WT compared to the TTPΔARE HSCs in young and middle-aged mice. We observed that while the majority of DEGs from young TTPΔARE HSCs had reduced expression compared to WT (768), the most significantly affected pathways predicted were increased, and surprisingly, by a key decay target of TTP, *Tnf* mRNA (Fig. 2C, Supplemental Fig. 3C). This was also observed in the analysis of pathways enriched in the middle-aged TTPΔARE HSCs. *Tnf* and *Tgfb1* mRNA, both validated targets of TTP [20], were predicted as two of the most significantly increased upstream regulators in both young and middle-aged TTPΔARE HSCs, yet with different presumed effects on downstream inflammatory pathways (Supplemental 3D-E). Given the critical role of TNF in HSCs, largely through extrinsic signaling through TNF receptors [21, 22], this upregulation of pathways involved in TNF production may be a compensatory mechanism for the HSCs as there is no significant decrease in TNF levels in the TTPΔARE HSCs though it is a direct target of TTP (Supplemental Fig. 3A). GO term analysis of overlapping DEGs between the young and middle-aged TTPΔARE HSCs compared to age-matched WT HSCs also highlighted TNF production as a consistently activated pathway and shows repression of cell migration and motility pathways (Fig. 2D).

To determine if these changes were specific to the stem cell compartment, we also analyzed the MPP3 cells purified from young TTPΔARE and WT mice. We chose this progenitor population to evaluate because the cell numbers in this population were consistently decreased in the TTP overexpressing mice, and thus likely to be affected by the overexpression of TTP. There was a robust separation between the transcription profiles of the WT and TTPΔARE MPP3 cells (*n* = 4 WT, 3 TTPΔARE, Fig. 2E) consistent with the changes seen in HSCs. In contrast to the young HSCs, there were significantly more transcripts upregulated in the setting of TTP overexpression (596) compared to downregulation (102) (Fig. 2F). While downregulated genes in the young TTPΔARE MPPs were largely in pathways required for cell adhesion and wound healing, TTPΔARE MPP3s had increased expression of genes involved in lymphocyte activation and cytokine production (Suppl Fig. 3G). Similar to the TTPΔARE HSC, IPA pathways that were enriched in the TTPΔARE MPP3s showed robust increases in pathways involved in immune response and TNF activation (Fig. 2G).

Given the robust predictions for elevated TNF pathways, and previous reports demonstrating *Tnf* mRNA is a direct target of TTP, we wanted to evaluate the accumulation of other potential target transcripts of TTP-directed mRNA degradation. Using targets curated in POSTAR3 from publicly available TTP CLIP-seq data [20] (Supplemental Table 3), only ~ 7% of downregulated mRNAs (55 of 768) in young TTPΔARE HSCs were predicted to be direct targets of TTP. A similar frequency of reported TTP targets was found in the downregulated transcripts from middle-aged HSCs (16 of 220). Analysis of TTP targets’ expression differences that overlapped between the young and middle-aged HSC profiles showed only four transcripts with consistent, significant downregulation in both: *Socs3*, *Il1r1*, *Ncam1*, and *Pbx1*. Only *Il1r1* was also decreased in the MPP3s overexpressing ZFP36, showing consistent decreases in expression in all three comparisons of primitive marrow cells overexpressing TTP.

To further evaluate the unique transcript level changes in hematopoietic progenitor cells, we also compared the changes in transcript levels seen when TTP was overexpressed in HeLa cells. In these cells, transcripts involved in innate immunity, including type I interferon signaling and viral response pathways, were upregulated when a tenfold increase of *Zpf36* was present [23]. While *Zpf36* mRNA was the only consistently overexpressed transcript in all four comparisons between cells overexpressing TTP compared to their WT counterpart (Supplemental Table 4), both the MPP3 and HeLa cells exhibited more transcripts with upregulation (MPP3 596, HeLa 596) compared to downregulation (MPP3 102, HeLa 231, Fig. 2F) [23], in contrast to the mostly decreased expression seen with TTP overexpression in the young HSCs. We also saw similarities in the pathways enriched in both the MPP3 and HeLa cells (Fig. 2G).

### HSCs with TTP overexpression have reduced reconstitution potential and skewed lineage output, but ameliorate CAIA-induced inflammation

**Fig. 3:**
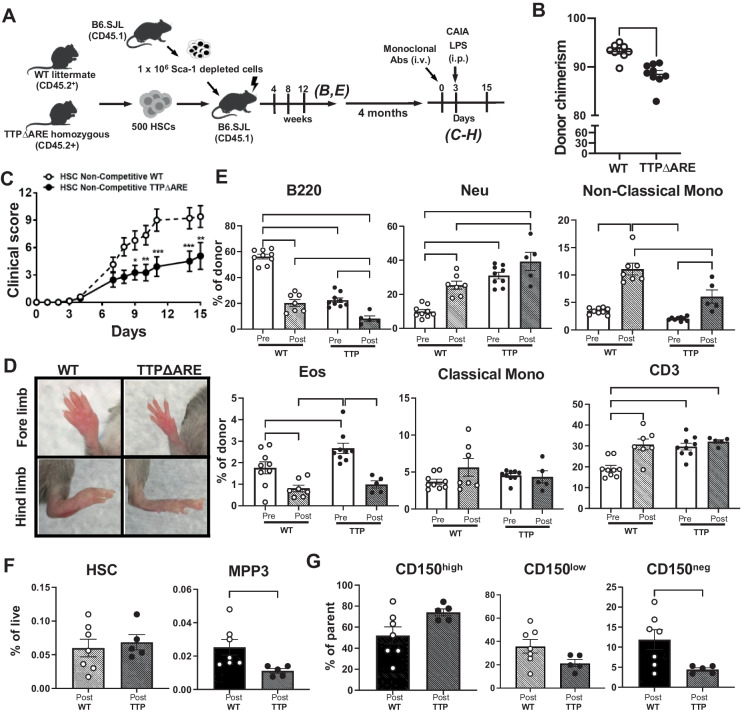
Non-competitive HSC transplantation. **A** Schematic of non-competitive TTPΔARE and WT littermate HSC transplants, with CAIA mouse induction, post-recovery after mAb cocktail injection, and LPS injection 3 days later. **B** Chimerism at 12 weeks post-transplant (WT = 10, TTPΔARE = 9). **C** CAIA clinical scores during the inflammation challenge (WT = 7, TTPΔARE = 5). **D** Representative limb images at day 14 post-CAIA antibody injection. **E** Frequency of donor-derived cells in blood pre- and post-CAIA. **F**, **G** Frequency of donor-derived cell populations in the bone marrow. Frequency of live (**F**) and frequency of parent (LSK CD34^−^Flk2.^−^) (**G**). Results are expressed as mean ± SEM. *P < 0.05; **P < 0.01; ***P < 0.001; ****P < 0.0001 calculated by Mann–Whitney *U* test (**B**, **E**, **F**, **G**) and two-way ANOVA with Bonferroni’s multiple comparisons test (**C**, **E**)

To determine if the transcript changes seen in the HSC compartment in the presence of TTP overexpression were associated with the hematopoietic phenotypes defined above (Fig. 1), we non-competitively transplanted 500 CD150^+^ HSCs (LSKCD34^+^Flk2^−^CD150^+^) purified from young TTPΔARE or WT mice into lethally irradiated CD45.1 recipient mice. We monitored lineage output every 4 weeks (Fig. 3A) by flow cytometric analysis of peripheral blood (PB) over a 3-month period and saw a significantly lower total donor chimerism (frequency of live cells derived from donor HSCs) of mice transplanted with TTPΔARE HSC, even under non-competitive (non-comp) conditions (Fig. 3B).

To examine whether mice with TTP overexpression exclusively in hematopoietic cells would be resistant to the inflammatory response in a disease-challenge model, we maintained the non-competitive HSC recipient mice for an additional 4 months to ensure that the mice would survive the LPS challenge after lethal irradiation. Thus, the recipient animals were middle-aged (with hematopoietic systems derived from either WT or TTPΔARE HSCs) when we challenged them with the collagen antibody-induced arthritis model of inflammation (CAIA, Chondrex). The limbs were scored for the duration of the experiment, and a clinical score was provided for each mouse. Mice with hematopoietic systems overexpressing TTP demonstrated significant suppression of the arthritis symptoms seen compared to WT (Fig. 3C, D).

To explore whether the composition of the blood contributed to the mitigation of inflammation, we compared peripheral blood samples from the TTP overexpressing HSCs, both before and at the end of the CAIA challenge. We saw that the blood composition in the mice reconstituted by the TTPΔARE HSCs before the challenge (WT-Pre vs TTP-Pre) showed similar cell frequencies to those seen in the TTPΔARE donor mice, including significant loss of B220^+^ cells and significant increases in eosinophils, neutrophils, and CD3^+^ cells (Figs. 1B, C and 3E). This supports the hypothesis that the composition of blood cells in the TTPΔARE donor mice is independent of non-hematopoietic cells overexpressing TTP. We then compared changes in the blood cell populations at the end of the CAIA challenge (WT-Post vs WT-Pre, TTP-Post vs TTP-Pre) to determine if the change in composition is associated with the response. The blood cells in the WT and TTP reconstituted mice had the same trajectories after the CAIA challenge, with losses of eosinophils and B220^+^ cells, and increases in neutrophils and non-classical monocytes after the inflammatory challenge (Fig. 3E). Only non-classical monocytes exhibited significant differences between the WT-Post and TTP-Post comparisons that were not seen in the baseline comparisons (Fig. 3E).

Following the CAIA challenge, we euthanized the animals to examine if any of the blood changes post-CAIA were reflected in primitive bone marrow progenitor populations. We did not see significant differences in the frequency of HSCs or the CD150^High^ subset of HSCs derived from the WT or TTPΔARE donors (Fig. 3F, G), but we did see significant losses of MPP3 and lymphoid-biased CD150^Neg^ cells (similar to donor TTPΔARE marrow: Figs. 1D-F and 3F, G). We saw the same phenotypes of reduced overall reconstitution from non-competitive TTPΔARE transplants when reconstituting the mice with whole bone marrow rather than purified HSCs (Supplemental Fig. 4A, B). This reduced reconstitution was also associated with overall suppression of the CAIA clinical scores in recipients with TTPΔARE donor whole bone marrow compared to WT donors (Supplemental Fig. 4C, D).

### TTP overexpressing cells have a dominant phenotype in a CAIA-induced model of inflammation

**Fig. 4:**
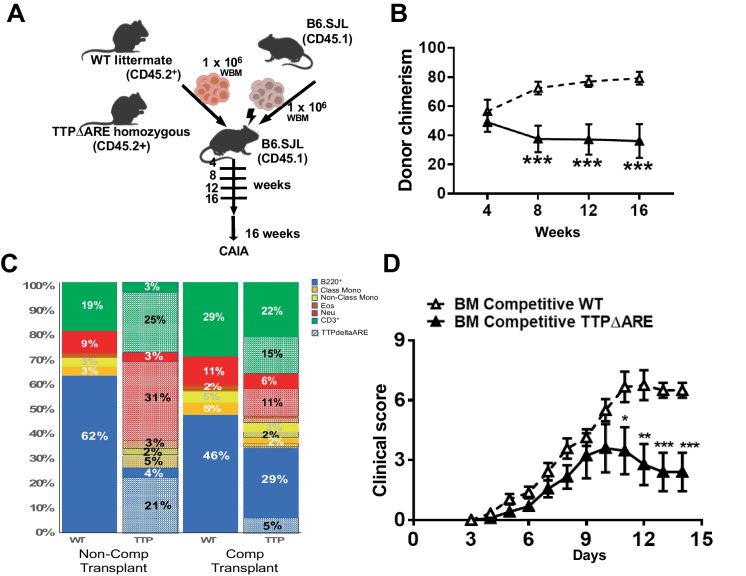
TTP overexpression-ameliorated CAIA inflammation in BM competitive transplants. **A** Schematic of competitive bone marrow transplantation and the inflammatory challenge induced by mAb cocktail injection at day 0, followed by LPS injection at day 3 (WT = 6, TTPΔARE = 8). **B** Donor chimerism in peripheral blood. Statistical significance was determined using an unpaired non-parametric Mann–Whitney *t*-test. **C** Composition of the blood from recipient animals of the non-competitive and competitive transplants using WT or TTPΔARE bone marrow. **D** Clinical scores after CAIA and LPS injection. Statistical significance was determined by two-way ANOVA (WT = 6, TTPΔARE = 8). Results are expressed as mean ± SEM with *P < 0.05; **P < 0.01; ***P < 0.001

Even though the TTPΔARE cells had overall reduced reconstitution capacity (Fig. 3B, Supplemental Fig. 4B), the recipient animals had robust protection against the CAIA inflammatory challenge. To explore how TTP overexpressing bone marrow could compete against WT cells, we performed competitive whole bone marrow (WBM) transplants, mixing equal numbers of competitor (CD45.1) marrow cells with either WT or TTPΔARE bone marrow cells (CD45.2) into lethally irradiated CD45.1^+^ mice (Fig. 4A). Overall blood chimerism in the competitive marrow transplants showed significantly reduced reconstitution potential of TTP overexpressing bone marrow over the 4 months (Fig. 4B).

Given the significant differences in the composition of TTPΔARE blood (Fig. 1A–C) that were reproduced in the transplants with TTPΔARE bone marrow cells (pre-CAIA challenge: Fig. 3E), we examined the overall composition of blood in the competitive transplants compared to frequencies in the non-competitive HSC transplants (Fig. 4C). In competitive transplants, there will be cells derived from both WT and donor (either WT or TTPΔARE) present and that may affect the total frequencies of the different cell populations. We saw a less dramatic difference in the total frequency of B220^+^ cells between the WT and TTPΔARE donors (blue bars) in the competitive transplant compared to the non-competitive transplant (non-comp: 62% vs 25%, comp: 46% vs 34%). Importantly, only a small fraction of the B-cells were derived from the TTPΔARE bone marrow (hashed blue bar, 5% of 34% total) in the competitive setting (Fig. 4C). Similarly, a significant increase in total neutrophil frequency was seen in the TTPΔARE non-competitive marrow was lost in the competitive transplants (red bars, non-com: 9% vs 34%, comp: 11% vs 17%). Thus, the major differences in total blood cell composition were less pronounced in the competitive transplants, with TTPΔARE marrow having minor contributions to the overall peripheral blood, in agreement with the less competitive overall reconstitution potential of TTPΔARE cells.

Interestingly, even though only approximately 30% of the total blood cells were overexpressing TTP in the competitive transplants, recipients of TTPΔARE marrow were still able to robustly mitigate the inflammation response derived from the CAIA challenge (Fig. 4D). This suggests that even a small fraction of TTPΔARE cells can generate a dominant response attenuating the CAIA-induced inflammation, and the response is not simply correlated to changes in the composition of the blood cells.

## Discussion

TTP or ZFP36 has been implicated in playing key roles in inflammatory diseases such as RA, and overexpression of TTP throughout the body that can mitigate the inflammation response in models of experimental arthritis [2] and other inflammatory disease models (reviewed in [2, 13]. We focused on the role of TTP overexpression in the hematopoietic stem and progenitor cells in young and middle-aged mice and determined the exclusive expression of TTPΔARE in hematopoietic cells could lead to the mitigation of inflammation challenge in middle-aged mice.

Increased expression of TTP in the hematopoietic system led to significant changes in the blood composition of young and middle-aged mice, with pronounced decreases in B220^+^ cells and increases in the frequency of eosinophils and a subset of T cells (Fig. 1). In the middle-aged mice, extensive changes in the frequency of primitive bone marrow cells were associated with age, rather than changes in the levels of TTP expression. However, significant changes in the composition of the stem cell compartment (using CD150 to separate lineage-biased HSCs), with increased frequency of myeloid-biased HSCs, seen in young TTPΔARE mice, were lost by middle age. This may be attributable to age-associated increases in myeloid-biased HSCs in the WT animals during aging [17]. As aging is associated with increased inflammation, we hypothesized that if blood parameter changes were associated only with increased inflammation, some of these changes in frequencies might be mitigated in the TTP overexpressing mice; however, we did not observe any age-associated changes in the WT blood parameters being mitigated in the TTP overexpressing mice (Fig. 1). Surprisingly, HSC aging is also associated with increased *Zpf36* expression [19], possibly as a response to upregulation of systemic inflammatory signaling, but this elevated expression of TTP alone cannot counteract “inflammaging.”

TTP regulates inflammatory signals, and cytokine signaling can affect HSC’s functional potential and differentiation choices [24, 25]. To assess whether altered gene expression in early progenitors could contribute to the altered composition of the peripheral blood, we examined the transcript changes and found that the overexpression of TTP had a larger effect on the transcription profiles of the young stem cells, with the majority of changes associated with decreased expression. Focusing on the overlapping signatures in both young and middle-aged HSCs from the TTP overexpressing mice, we found that the increased levels of TTP led to the upregulation of pathways that are targets of TNF and IL-6, without directly affecting the mRNA levels of *Tnf*, *Il10*, or *Il6*: all of which are reported targets of degradation from TTP. We propose that some of downregulated genes represent novel TTP targets in the young HSCs as 527 of the 768 downregulated transcripts have AU-rich domains in their 3′UTR [26]. We also propose that many of these transcriptional changes are secondary, and it will be interesting to discriminate primary from secondary effects using ChIP to define HSC-specific TTP binding and transcriptional regulation.

Upon differentiation, the increase in *Zpf36* mRNA levels was associated with large changes in the MPP3 population, with a predominance of upregulated transcripts having involvement in immune response and decreases in pathways important for adhesion and cell junction assembly. This signature was reminiscent of profiles generated from supra-physiologic levels of ZPF36 in HeLa cells [23] but unique from the stem cell phenotype.

Of the predicted direct targets of TTP from CLIP-seq data [20], only *Il1r1* was downregulated in all three hematopoietic cell types. This downregulation of *Il1r1* may be particularly relevant to hematopoiesis due to its function as a receptor for IL-1, a major pro-inflammatory cytokine that promotes HSC cycling response to stress signals. The impaired myeloid reconstitution previously reported in IL-1R1 knockout mice [27] was not observed in the transplanted/challenged HSCs with overexpression of TTP (and thus decreased expression of *Il1r1).* Instead, the reconstitution potential of young HSCs with reduced *Il1r1* (TTPΔARE donors) was decreased, similar to results in transplants of *Il1r1*^*−/−*^ hematopoietic cells exposed to chronic Il1B exposure [28]. This leads us back to the increased TNF response as a potential mechanism decreasing the reconstitution potential, as it was recently reported that TNF stimulation of HSCs leads to the overall loss of reconstitution potential [29]. At homeostatic conditions in both the young and middle-aged mice, TTP overexpression drives what appears to be a compensatory response by increasing the expression of genes involved in TNF production in the HSC compartment (Fig. 2D).

Though there was decreased reconstitution potential of TTPΔARE HSCs in the stem cell and whole BM transplants, the changes in blood composition and mitigation inflammation reproduce the phenotypes reported in mice with global TTP overexpression [30]. Mice with selective TTP overexpression in hematopoietic cells were able to mitigate the CAIA inflammatory challenge, inducing symptoms similar to those found in human RA, with a significant reduction in the signs of joint inflammation. In competitive transplant experiments, the TTPΔARE donor contribution to the peripheral blood of recipient mice was significantly lower compared to the WT donors, though the total blood (derived from contributions of both the competitive and donor marrow) differences were not as dramatically affected as in the non-competitive transplants (Fig. 4C). This suggests that it is not simply the changes in cell composition that are driving the mitigation of inflammation. In both the non-competitive transplants and competitive transplants, recipients of the TTPΔARE cells had an overall reduction in total B-cells (blue bars). However, in the TTPΔARE competitive transplants, there was an attenuation of the neutrophil expansion seen in the non-competitive transplant (red bars). Even though few global changes were seen in mice with low frequencies of TTPΔARE-derived cells, these cells overexpressing TTP were able to robustly mitigate the inflammation challenge of the CAIA model. These data suggest that, even at low abundance, TTP overexpressing cells could conceivably confer protection against the inflammatory stimuli seen in diseases such as RA and possibly mitigate inflammaging signaling. We found cell-specific changes in the transcriptional profiles between the stem and progenitor cells, and this suggests that transcriptional regulation by TTP will be unique for many hematopoietic cell types. As *Zfp36* expression increases with aging in HSCs, in an environment of increased inflammation, it is likely the inflammatory reduction seen in the CAIA challenge is not driven by the HSC response, but instead by the small frequency of differentiated cells that also overexpress TTP. This still provides a potential for an autologous therapeutic strategy for inflammatory diseases such as RA or during aging, with modification of patients’ hematopoietic stem cells to overexpress TTP, which leads to their progeny also expressing higher levels of TTP, and even if these differentiated cells are present in small numbers, they may provide long-term relief of symptoms and signs associated with chronic inflammatory conditions.

## Methods

### Animals

Homozygous TTPΔARE and WT TTPΔARE littermate control mice were described previously [30]. Only male mice were used for the experiments (young mice, 4–5 months old and middle-aged mice, 1 year old). Recipient mice used for transplantation, and as sources of competitor cells and Sca1-depleted rescue cells, were CD45.1 mice purchased from the Jackson Laboratory. Female CD45.1 mice, also from Jackson Laboratory, were used as transplant recipients (4–5 months old).

### Complete blood cell counts

PB from mice was retro-orbitally collected into lithium heparin-coated Microvette tubes (Sarstedt), and the tubes were gently rotated for 10 min. Complete blood cell counts were analyzed using a Hemavet 950FS (Drew Scientific) according to the manufacturer’s instructions.

### Flow cytometry and cell sorting

Details of mouse antibodies and viability dyes used for each staining panel are shown in Supplemental Fig. 1. HSCs, progenitors, and PB cells were analyzed as previously described [17, 31–33]. Cell acquisition and analysis were performed using FACSAria III (BD Biosciences) and BD FACSDiva™ software (BD Biosciences). Phenotypically defined CD150^+^HSC and MPP3 cells were sorted using a BD FACSAria III cell sorter. Analysis was performed using FlowJo software, version 10.6 (FlowJo LLC, OR, USA).

### RNA-seq and data analysis

RNA was purified with TRIzol™ Reagent (Thermo Fisher Scientific) and Direct-zol RNA Microprep (Zymo). cDNA libraries were prepared with the SMART-seq® v4 Ultra® Low Input RNA Kit for Sequencing (TaKaRa), according to the manufacturer’s protocol. Sequencing libraries were constructed using Nextera XT DNA Library Preparation Kit (Illumina), with 125 pg input cDNA. Sequencing was carried out on an Illumina HiSeq 2500 instrument using 2 × 105 bp reads. A total of 1.1 billion sequencing reads were used, with an average of 46 million reads per sample. To analyze transcription datasets, an index sequence for STAR was built using the GENCODE M22 reference feature including protein-coding and non-coding genes. Before sequence alignment, we applied Trim Galore! (version 0.4.3) with the Cutadapt package (version 1.12) [34] to remove avoidable genomic fragments (e.g., adapter dimers) and low-quality nucleotide sequences from the reads. Then, adapter-trimmed sequencing reads were mapped to the mouse reference genome (10 mm) using STAR aligner [35], and the raw counts were calculated using the featureCounts package (gene-level) [36]. We generated differentially expressed transcript (DET) lists with edgeR using the following cutoffs: fold change > 1.2, logCPM > 1.5, and false discovery rate (FDR) < 0.05 [37]. To find significant gene ontology (GO) terms using DETs, we used clusterProfiler [38]. GSEA was used for pathway analysis [39]. All original RNA-seq data were deposited into the NCBI’s Gene Expression Omnibus database (GEO GSE226334).

### BM and HSC transplantation

Recipient mice were irradiated with 9.56 Gy using a γ-ray source. Six hours after irradiation, irradiated mice were then anesthetized with isoflurane and given BM cells or sorted CD150^+^ cells via retro-orbital injection. The reconstitution of donor cells in PB was evaluated at 4, 8, and 12 weeks, and also at 16 weeks. The CAIA model was induced after 16 weeks (see below), and BM cells were harvested for immunophenotyping of HSCs and progenitors.

For the non-competitive HSC transplantation, Sca-1 depleted cells were isolated from BM of CD45.1^+^ mice using immunomagnetic beads (Stem Cell Technologies) and a biotinylated anti-mouse Sca-1 antibody (BioLegend). After depletion, 500 HSCs from WT or TTPΔARE were mixed with 1 × 10^6^ Sca-1 depleted cells, and the mixture was injected into lethally irradiated mice. For WBM non-competitive transplants, 1 × 10^6^ marrow cells were injected into lethally irradiated mice. For the competitive BM transplantation, competitor cells were obtained from BM in CD45.1^+^ mice. WT or TTPΔARE marrow cells (1 × 10^6^) were mixed with 1 × 10^6^ competitor cells, and the cells were injected into lethally irradiated mice.

### CAIA

Arthritis was induced in transplants by intravenous injection of a cocktail of five monoclonal antibodies to type II collagen (5.0 mg at day 0), followed by intraperitoneal injection of 5 μg of lipopolysaccharide (LPS) on day 3 (Chondrex). The severity of arthritis was assessed according to paw swelling and redness on a scale of 0–4. The arthritis score for each mouse was expressed as the sum of the scores of the four limbs. Thus, the maximum score an animal could attain was 16. Mice were euthanized on day 14 or 15, and blood and serum were collected on day 7 and the final day of the experiment. Clinical development of arthritis in the paws was assessed by the arthritis score: 0, normal; 1, erythema and mild swelling confined to the tarsal or ankle joints; 2, erythema and mild swelling extending from the ankle to the tarsal joints; 3, erythema and moderate swelling extending from the ankle to metatarsal joints; 4, erythema and severe swelling encompassing the ankle, foot, and digits, or ankylosis of the limb [40].

### Statistical analysis

All data are expressed as mean plus or minus standard error of the mean (SEM). Statistical analysis was performed using Prism software versions 7 and 8 (GraphPad, La Jolla, CA). Data were calculated with unpaired *t*-tests for comparisons between WT and TTPΔARE, one-way ANOVA with Tukey’s multiple comparisons tests, or two-way ANOVA with Bonferroni’s multiple comparisons tests, according to the experimental design. A *P* value less than 0.05 was considered significant. All statistical analyses and *n* are reported in the figure legends.

### Study approval

All animal studies were approved by the Institutional Animal Care and Use Committee at National Institute on Aging and were performed in accordance with guidelines from the National Institutes of Health (469-TGB-2025).

### Supplementary Information

Below is the link to the electronic supplementary material.

Supplementary file1 (PDF 1585 KB)

Supplementary file2 (PDF 417 KB)

Supplementary file3 (PDF 531 KB)

Supplementary file4 (PDF 348 KB)

Supplementary file5 (PDF 72 KB)

## Data Availability

Data underlying this article are available in the article, on GEO(GSE226334), and in its online supplementary material. Any other data not presented in the article will be readily provided by request.
