# Supplementary material for: Tristetraprolin overexpression drives hematopoietic changes in young and middle-aged mice generating dominant mitigating effects on induced inflammation in murine models
Source: GeroScience. 2023 Aug 3;46(1):1271–84. doi: 10.1007/s11357-023-00879-2 (PMC10828162; doi:10.1007/s11357-023-00879-2)
Supplement: Supplementary file 1 — Supplementary file1 (PDF 1585 KB) [file 11357_2023_879_MOESM1_ESM.pdf]

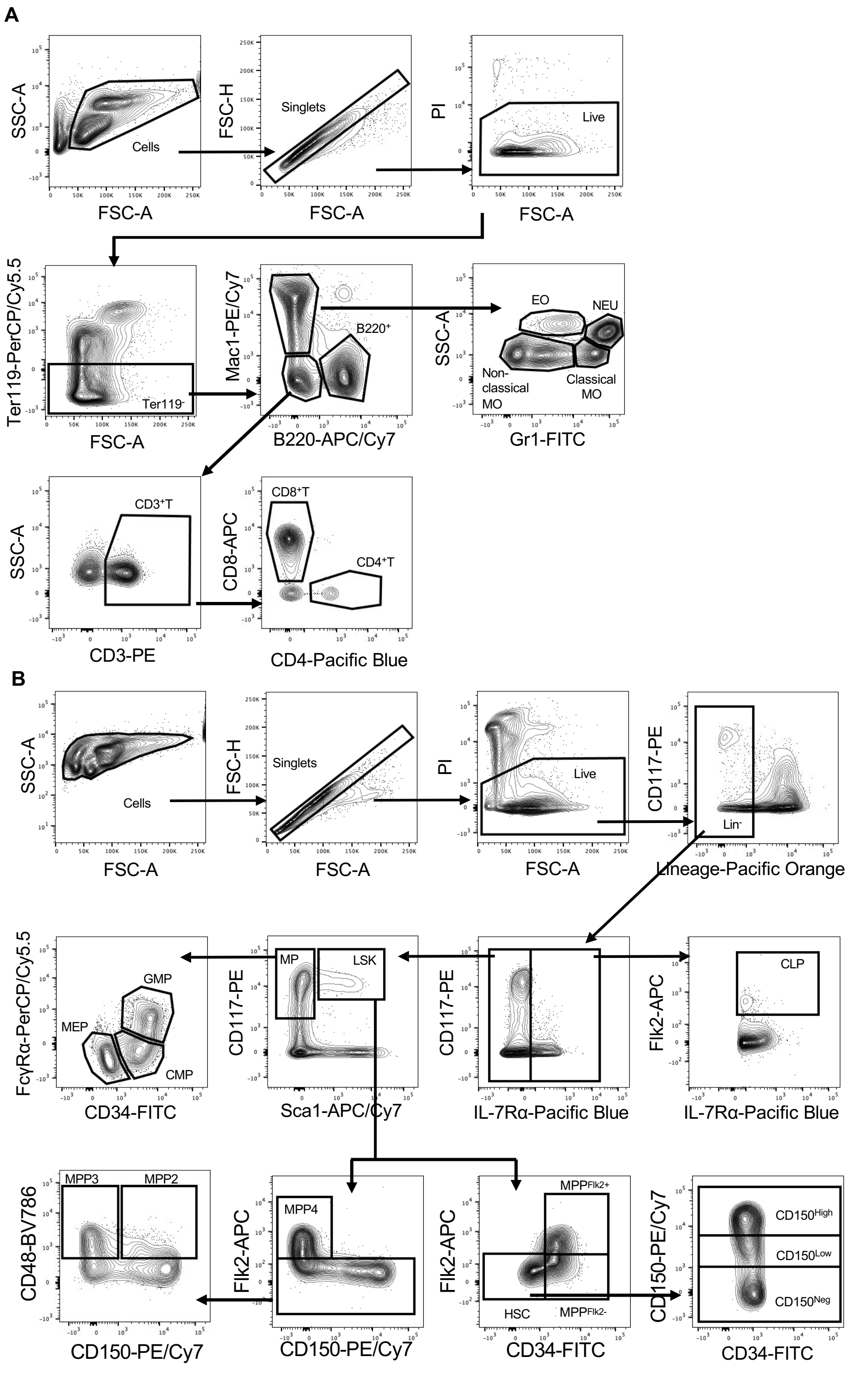

Supplemental Figure 1. Flow cytometry gating strategy used to define cell populations
(A) Peripheral blood gating (B) Bone marrow.

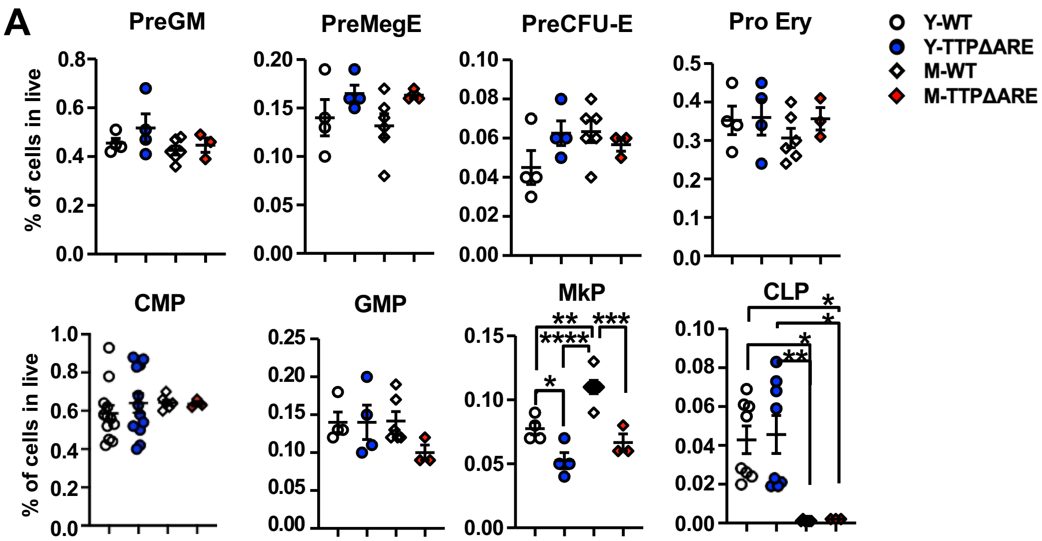

Supplemental Figure 2. Progenitor population frequency in TTP overexpressing and control mice (A) Frequency of Pre GM, Pre MegE, Pre CFU-E, Pro Ery, CMO, GMP, MkP, and CLP in live cells in BM. Results expressed as mean ± SEM. *$P < 0.05$; **$P < 0.01$; ***$P < 0.001$; between indicated groups calculated one-way ANOVA with Tukey's multiple comparisons test

Supplementary Figure 3.

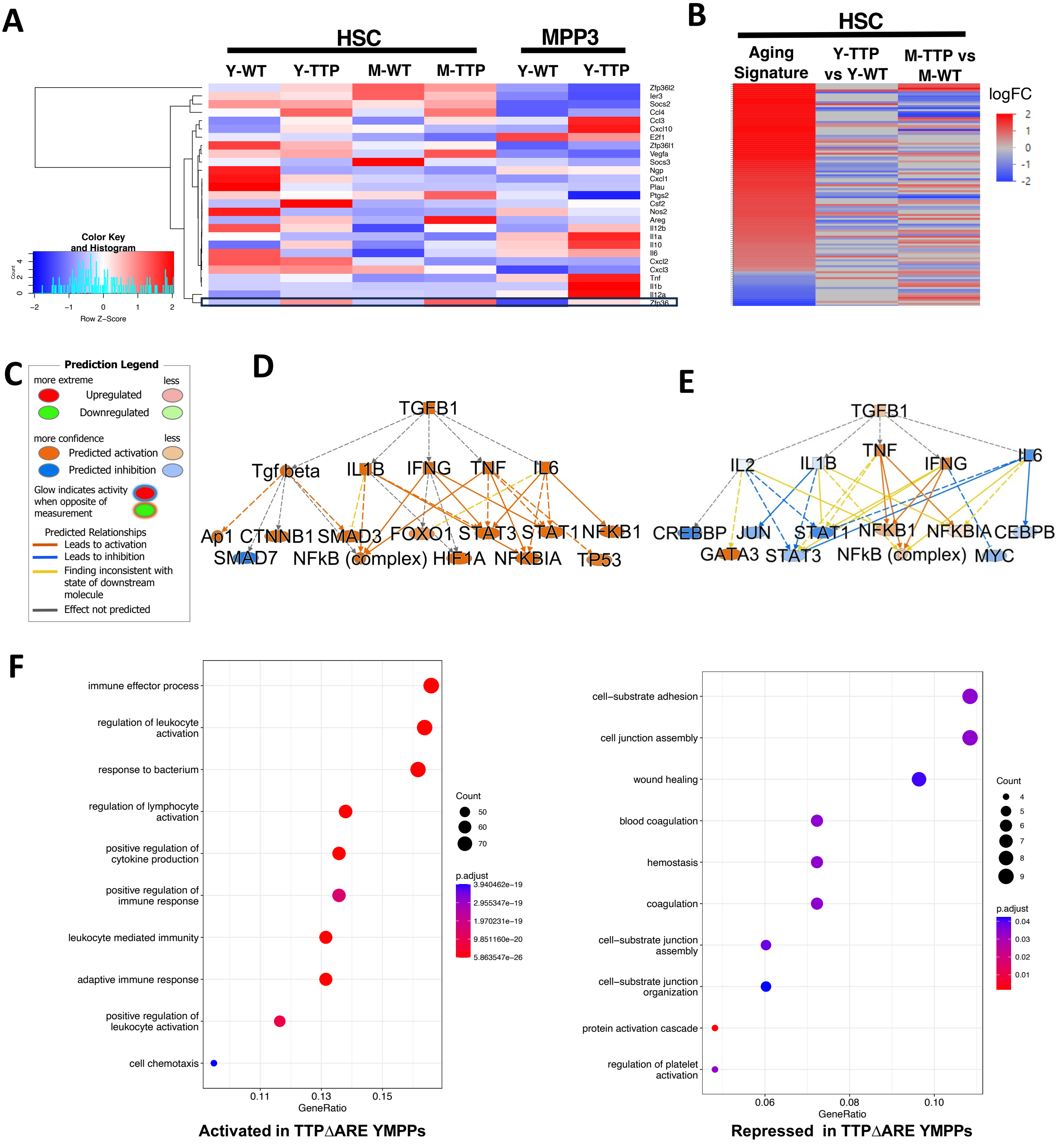

Supplemental Figure 3. A) Heatmap of average expression levels of Zfp36, family members, and genes affected by TTP clustered by gene expression levels B) Log2Fold Change of HSCs overexpressing TTP from young or middle-aged mice relative to Aging Signature Log2Fold Change in aged vs young mice C) IPA prediction legend D-E) Upstream regulator predictions significant for TGFb1 and TNF for both young (D) and middle-aged (E) HSCs comparing WT and TTPΔARE. (F) GO pathway enrichment for DEGs between TTPΔARE and WT YMPPs

Supplementary Figure 4.

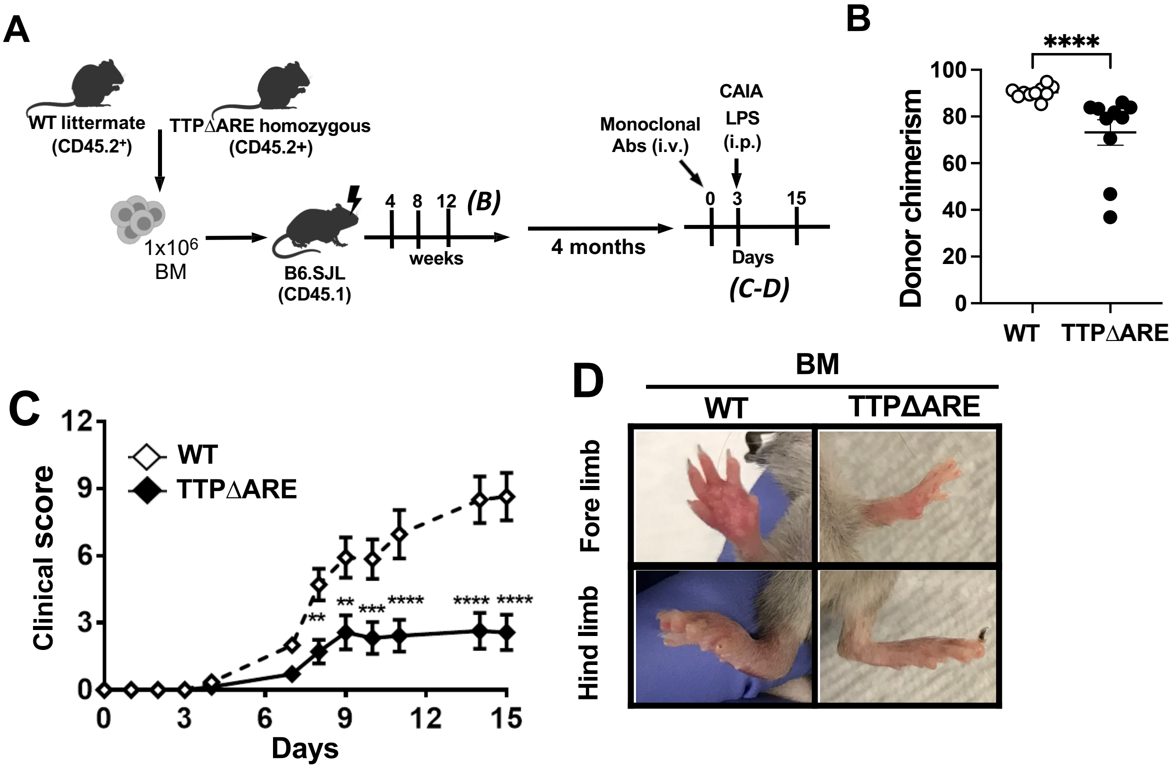

Supplementary Figure 4. (A) Schematic of non-competitive TTP∆ARE and WT littermate whole bone marrow transplant and CAIA mouse induction post recovery by mAb cocktail injection and LPS injection 3 days later (B) Chimerism at 12 weeks post-transplant (WT=10, TTP∆ARE =10) (C) Clinical score determined every day. Statistical significance determined using two-way ANOVA. (D) Typical mouse fore- and hin-limb at day 14. Results are expressed as mean ± SEM. *$P < 0.05$; **$P < 0.01$; ***$P < 0.001$; ****$P < 0.0001$: calculated by Mann-Whitney *U* test (B) and two-way ANOVA with Bonferroni's multiple comparisons test (C)
