# Supplementary material for: Tristetraprolin overexpression drives hematopoietic changes in young and middle-aged mice generating dominant mitigating effects on induced inflammation in murine models
Source: GeroScience. 2023 Aug 3;46(1):1271–84. doi: 10.1007/s11357-023-00879-2 (PMC10828162; doi:10.1007/s11357-023-00879-2)
Supplement: Supplementary file 2 — Supplementary file2 (PDF 417 KB) [file 11357_2023_879_MOESM2_ESM.pdf]

Supplemental Table 1: DEGs WT vs TTP 1.2fold

Young WT_ HSC vs Young TTPdeltaARE_HSC

Expression_Direction	Gene	logFC	PValue	FDR
WT<TPPdeltaARE	Igkv1-117	10.6466834	9.27E-11	8.51E-08
WT<TPPdeltaARE	Ighv5-17	8.05638023	5.97E-07	7.82E-05
WT<TPPdeltaARE	Ighv1-81	7.74907604	0.00014975	0.0036163
WT<TPPdeltaARE	Ighv1-59	6.1428661	4.22E-05	0.00143785
WT<TPPdeltaARE	Igkv9-120	5.89884236	0.00138317	0.01807117
WT<TPPdeltaARE	Ighv5-16	5.5654595	0.00065578	0.01070071
WT<TPPdeltaARE	Iglc1	4.83018432	0.00091647	0.01341017
WT<TPPdeltaARE	Iglv2	4.34081046	3.01E-05	0.00115378
WT<TPPdeltaARE	Iglj1	4.15165396	0.00035052	0.00683694
WT<TPPdeltaARE	Iglv3	3.78235584	0.00044066	0.00795118
WT<TPPdeltaARE	Pdcd1	3.60391572	9.83E-09	3.21E-06
WT<TPPdeltaARE	Iglv1	3.59795931	0.00092063	0.01345066
WT<TPPdeltaARE	Hba-a1	3.4677015	1.94E-16	9.32E-13
WT<TPPdeltaARE	Car1	3.34601703	1.72E-13	3.68E-10
WT<TPPdeltaARE	Atp1b2	2.79012388	9.43E-10	5.34E-07
WT<TPPdeltaARE	Trdc	2.73907832	0.00151329	0.01926192
WT<TPPdeltaARE	Ermap	2.70832308	9.29E-06	0.00049314
WT<TPPdeltaARE	Hbb-bs	2.46169625	5.98E-10	3.49E-07
WT<TPPdeltaARE	Ccr7	2.38686183	0.00362487	0.03608201
WT<TPPdeltaARE	Fasl	2.0710559	4.78E-06	0.00031323
WT<TPPdeltaARE	Gm15915	2.00165213	1.82E-08	5.07E-06
WT<TPPdeltaARE	Cd8a	1.95646407	1.00E-07	1.88E-05
WT<TPPdeltaARE	Gm48719	1.92318002	8.36E-07	9.42E-05
WT<TPPdeltaARE	Il7r	1.8746698	7.23E-05	0.00216758
WT<TPPdeltaARE	Klrc1	1.82620967	7.91E-05	0.00230043
WT<TPPdeltaARE	Gm26740	1.82122401	3.94E-05	0.00138148
WT<TPPdeltaARE	Cxcr6	1.80020883	3.57E-07	5.21E-05
WT<TPPdeltaARE	Gm37509	1.7865038	5.81E-10	3.49E-07
WT<TPPdeltaARE	Klf1	1.78135321	3.86E-06	0.00027862
WT<TPPdeltaARE	Sphk1	1.77602947	0.00393771	0.03836383
WT<TPPdeltaARE	Gpr68	1.76823355	9.63E-05	0.00265547
WT<TPPdeltaARE	Cd40lg	1.7253929	8.57E-07	9.55E-05
WT<TPPdeltaARE	Ppm1j	1.69354298	1.71E-06	0.00015659
WT<TPPdeltaARE	Cd2	1.68474602	2.05E-06	0.00017652
WT<TPPdeltaARE	Cd3e	1.662245	1.57E-06	0.00014658
WT<TPPdeltaARE	Ccr5	1.64308593	1.56E-07	2.81E-05
WT<TPPdeltaARE	Tspo2	1.64240289	3.34E-05	0.00124097
WT<TPPdeltaARE	Il2rb	1.61350341	5.06E-05	0.00165367
WT<TPPdeltaARE	Ccl3	1.60582141	7.33E-18	7.07E-14
WT<TPPdeltaARE	Slfn1	1.60451826	0.00015653	0.00371935
WT<TPPdeltaARE	Tcrg-C4	1.58859254	0.00020025	0.00448192
WT<TPPdeltaARE	Hbb-bt	1.57923623	2.14E-15	8.26E-12

WT<TPPdeltaARE	Ccr2	1.57414751	7.79E-11	8.34E-08
WT<TPPdeltaARE	Tcrg-C2	1.54221788	2.51E-06	0.000201
WT<TPPdeltaARE	Cd6	1.52568394	1.47E-07	2.68E-05
WT<TPPdeltaARE	Ccl5	1.51773497	5.19E-06	0.00033464
WT<TPPdeltaARE	Cd226	1.50131498	5.49E-05	0.00176206
WT<TPPdeltaARE	Trbc2	1.469914	5.67E-06	0.0003525
WT<TPPdeltaARE	Cxcr3	1.46807046	4.14E-05	0.00142519
WT<TPPdeltaARE	Insm1	1.43011351	0.00326311	0.03353781
WT<TPPdeltaARE	Mt2	1.4260981	0.00147373	0.01892085
WT<TPPdeltaARE	Gm45774	1.42063885	4.74E-05	0.00157467
WT<TPPdeltaARE	Per1	1.41832687	1.07E-08	3.33E-06
WT<TPPdeltaARE	Cxcl10	1.39102564	8.22E-07	9.31E-05
WT<TPPdeltaARE	Ifi209	1.3745309	0.00015452	0.00368067
WT<TPPdeltaARE	Cd7	1.33887167	2.42E-05	0.00098643
WT<TPPdeltaARE	Gimap3	1.32606615	2.46E-06	0.00019974
WT<TPPdeltaARE	Iglc2	1.30458927	0.00458192	0.04301113
WT<TPPdeltaARE	Cxcr1	1.3027044	6.83E-06	0.00039512
WT<TPPdeltaARE	Sbspon	1.29851195	3.85E-08	9.28E-06
WT<TPPdeltaARE	Trac	1.28334671	0.00018248	0.00417644
WT<TPPdeltaARE	Gm2682	1.27234006	0.00178807	0.0217263
WT<TPPdeltaARE	Cd3d	1.26438513	0.00011974	0.00307246
WT<TPPdeltaARE	Ms4a4b	1.25667934	0.00053899	0.00924169
WT<TPPdeltaARE	Cd5	1.25030449	0.00399398	0.03875525
WT<TPPdeltaARE	Xcl1	1.24811459	0.00178505	0.02170333
WT<TPPdeltaARE	Zap70	1.23391127	0.00035307	0.00687077
WT<TPPdeltaARE	Ccr3	1.22802241	4.96E-06	0.00032303
WT<TPPdeltaARE	Cd3g	1.22551036	6.60E-05	0.0020264
WT<TPPdeltaARE	Sema4a	1.21848056	2.50E-07	4.05E-05
WT<TPPdeltaARE	Lpxn	1.19229612	9.18E-05	0.002559
WT<TPPdeltaARE	Gm14964	1.17326111	1.71E-06	0.00015659
WT<TPPdeltaARE	Tcf7	1.15873643	0.00283521	0.03020306
WT<TPPdeltaARE	Gimap7	1.14584862	3.93E-08	9.36E-06
WT<TPPdeltaARE	Cst7	1.14545049	0.00020489	0.00454881
WT<TPPdeltaARE	Slc25a21	1.14091492	0.00097887	0.01404599
WT<TPPdeltaARE	Hopx	1.13568171	2.84E-06	0.00021828
WT<TPPdeltaARE	C1qc	1.13277425	0.00298501	0.03134826
WT<TPPdeltaARE	Gm49838	1.1047489	0.00011701	0.00302661
WT<TPPdeltaARE	Fosl2	1.08841548	0.00046581	0.00826568
WT<TPPdeltaARE	Igha	1.08269248	0.00134768	0.01772953
WT<TPPdeltaARE	C1qa	1.08146156	4.72E-05	0.00157224
WT<TPPdeltaARE	P2rx7	1.0444049	5.18E-05	0.00167818
WT<TPPdeltaARE	Gm21887	1.00720134	0.00164507	0.02046618
WT<TPPdeltaARE	Gm48512	0.99650349	9.69E-06	0.0004979
WT<TPPdeltaARE	Tmem158	0.98083154	7.24E-06	0.00040951
WT<TPPdeltaARE	Zfp36	0.98022902	1.40E-08	4.08E-06

WT<TPPdeltaARE	Gm4956	0.97591222	0.00458211	0.04301113
WT<TPPdeltaARE	Adgre1	0.96477484	0.00281052	0.03003123
WT<TPPdeltaARE	C1qb	0.96372876	0.00018056	0.00415219
WT<TPPdeltaARE	Tmem255b	0.93959973	0.00034116	0.00668817
WT<TPPdeltaARE	Gkn3	0.93929278	6.73E-05	0.00204864
WT<TPPdeltaARE	Cyp26b1	0.90752859	0.00040647	0.00748144
WT<TPPdeltaARE	Id2	0.90744846	2.66E-05	0.00106311
WT<TPPdeltaARE	Itgb7	0.89967789	1.34E-05	0.00063586
WT<TPPdeltaARE	Tbx21	0.89609948	0.00389802	0.03811196
WT<TPPdeltaARE	Thbs1	0.88895195	0.00143994	0.01859859
WT<TPPdeltaARE	Slc16a11	0.8879271	0.0004023	0.0074259
WT<TPPdeltaARE	Lmcd1	0.87896508	0.00257115	0.02823282
WT<TPPdeltaARE	Gm47566	0.86918301	0.00079063	0.01215977
WT<TPPdeltaARE	Mt1	0.86856095	0.00178186	0.02167822
WT<TPPdeltaARE	Hopxos	0.86341929	7.08E-05	0.00213237
WT<TPPdeltaARE	Slc17a8	0.85764225	0.00100088	0.0142242
WT<TPPdeltaARE	Abtb2	0.85016401	0.0026274	0.02865459
WT<TPPdeltaARE	Gpr65	0.83494872	0.00036904	0.00704837
WT<TPPdeltaARE	Uchl1	0.823608	5.33E-05	0.00171957
WT<TPPdeltaARE	Gimap4	0.81027148	0.00026281	0.0054956
WT<TPPdeltaARE	Krt80	0.79978096	9.45E-05	0.00261155
WT<TPPdeltaARE	Hif3a	0.79734909	0.00485742	0.0449603
WT<TPPdeltaARE	Rgs11	0.79452554	4.13E-05	0.00142519
WT<TPPdeltaARE	Sema4f	0.79201625	0.00030906	0.00629271
WT<TPPdeltaARE	Slc40a1	0.78485318	0.00098279	0.01408134
WT<TPPdeltaARE	Klrb1c	0.78350721	0.00083538	0.01261648
WT<TPPdeltaARE	Gm47794	0.77036281	0.00436814	0.04159016
WT<TPPdeltaARE	Art2b	0.76549973	0.00047938	0.00841361
WT<TPPdeltaARE	Scn3b	0.75438919	0.00353212	0.03546184
WT<TPPdeltaARE	Spire2	0.74476297	0.00263102	0.02867786
WT<TPPdeltaARE	Itgb5	0.74291616	0.0007991	0.01225319
WT<TPPdeltaARE	Ccdc92	0.71768871	0.00030031	0.00615669
WT<TPPdeltaARE	Gm17041	0.71717497	0.00060929	0.01009601
WT<TPPdeltaARE	Ddx4	0.71607947	0.00270435	0.02921269
WT<TPPdeltaARE	Fbp1	0.69830449	0.0013872	0.01811163
WT<TPPdeltaARE	Myl10	0.69790783	0.00230514	0.02619243
WT<TPPdeltaARE	Cxcr4	0.69670449	5.24E-06	0.00033684
WT<TPPdeltaARE	Cish	0.69400017	0.0009303	0.01354284
WT<TPPdeltaARE	Hk3	0.69018003	1.81E-06	0.00016095
WT<TPPdeltaARE	Gm8989	0.68146025	0.00121274	0.0164317
WT<TPPdeltaARE	Ckb	0.6792676	0.00525487	0.0474761
WT<TPPdeltaARE	Gem	0.67139171	0.000397	0.00734935
WT<TPPdeltaARE	Gm17040	0.65835651	0.00546332	0.04867482
WT<TPPdeltaARE	Gas6	0.65551262	0.00145818	0.01877122
WT<TPPdeltaARE	Rab19	0.65454768	0.00021622	0.00471884

WT<TPPdeltaARE	Tsc22d3	0.65450472	0.0013375	0.01764205
WT<TPPdeltaARE	Pde3b	0.64782346	0.0005403	0.00924464
WT<TPPdeltaARE	Gpr183	0.63236564	0.00066557	0.01081107
WT<TPPdeltaARE	Cacnb1	0.6312386	0.00059255	0.00992372
WT<TPPdeltaARE	Nfil3	0.61940286	0.00271439	0.02928838
WT<TPPdeltaARE	Nkg7	0.6182839	0.00176007	0.02150617
WT<TPPdeltaARE	Sult1a1	0.61462671	0.00373415	0.03694089
WT<TPPdeltaARE	Ccl4	0.61432361	0.00011841	0.00305064
WT<TPPdeltaARE	Cfp	0.60578197	0.00221785	0.02551652
WT<TPPdeltaARE	Dusp6	0.58916855	0.00051188	0.00888682
WT<TPPdeltaARE	Zcchc18	0.58847833	0.00031281	0.00634409
WT<TPPdeltaARE	Ntn4	0.58573648	0.00335813	0.03416812
WT<TPPdeltaARE	Gm13881	0.57752858	0.00191018	0.02281586
WT<TPPdeltaARE	Padi4	0.55980703	0.00015255	0.00365654
WT<TPPdeltaARE	Lgals1	0.54781382	0.0001089	0.00290055
WT<TPPdeltaARE	Myc	0.54691716	0.0022998	0.02614717
WT<TPPdeltaARE	Otos	0.54575005	0.00110882	0.01529435
WT<TPPdeltaARE	Gm35551	0.53901175	0.00031812	0.00637272
WT<TPPdeltaARE	Ces2g	0.536618	0.00226179	0.025837
WT<TPPdeltaARE	Pfkfb3	0.52121098	0.00284142	0.0302525
WT<TPPdeltaARE	Pik3ip1	0.51076584	0.00104956	0.01472872
WT<TPPdeltaARE	Gfi1	0.50158756	0.00453127	0.04270031
WT<TPPdeltaARE	Pik3r1	0.49396637	0.0048871	0.04513553
WT<TPPdeltaARE	Stk17b	0.47657518	0.00283501	0.03020306
WT<TPPdeltaARE	Rdh10	0.46101626	0.00351286	0.03531368
WT<TPPdeltaARE	Slc2a3	0.45734983	0.00099342	0.01413899
WT<TPPdeltaARE	Neo1	0.45727992	0.00282097	0.03008457
WT<TPPdeltaARE	Btg1	0.45297414	0.00224265	0.02571889
WT<TPPdeltaARE	N4bp2l1	0.43777483	0.00296149	0.03120327
WT<TPPdeltaARE	Mycn	0.42442344	0.00249492	0.02763191
WT<TPPdeltaARE	Arhgef25	0.41703136	0.00527081	0.04753102
WT>TPPdeltaARE	Fgfr1	-0.4117631	0.00498059	0.04579168
WT>TPPdeltaARE	9930014A18	-0.4178423	0.00305025	0.03187707
WT>TPPdeltaARE	Pbx1	-0.4271063	0.00127285	0.01703508
WT>TPPdeltaARE	S1pr1	-0.4300268	0.00352528	0.03542007
WT>TPPdeltaARE	Zfp229	-0.4328006	0.00527975	0.04758938
WT>TPPdeltaARE	Atxn1	-0.441577	0.00461455	0.04314969
WT>TPPdeltaARE	Gda	-0.4433096	0.00464822	0.04337817
WT>TPPdeltaARE	Pla2g15	-0.4495445	0.00461377	0.04314969
WT>TPPdeltaARE	Plekho1	-0.4510923	0.00431015	0.0411274
WT>TPPdeltaARE	Mical3	-0.462686	0.00382422	0.03754286
WT>TPPdeltaARE	Chn2	-0.4747504	0.00316331	0.03273142
WT>TPPdeltaARE	Dhcr24	-0.4771667	0.00228657	0.02604289
WT>TPPdeltaARE	Fmnl3	-0.4903699	0.00555208	0.0493828
WT>TPPdeltaARE	Prune2	-0.4906479	0.00524225	0.04738432

WT>TPPdeltaARE	Sorbs2	-0.4928936	0.00313652	0.03256674
WT>TPPdeltaARE	Tecpr2	-0.4996881	0.00221499	0.02549887
WT>TPPdeltaARE	Usp40	-0.5034579	0.00062209	0.01027276
WT>TPPdeltaARE	Sash1	-0.508865	0.0036239	0.03608201
WT>TPPdeltaARE	Adgrg3	-0.5102481	0.00227248	0.02594354
WT>TPPdeltaARE	Bicdl1	-0.5152568	0.00206628	0.02413869
WT>TPPdeltaARE	Gng7	-0.5156809	0.00243394	0.02723838
WT>TPPdeltaARE	Csf3r	-0.5169537	0.00080021	0.01225319
WT>TPPdeltaARE	Plxnb2	-0.5202141	0.0004702	0.00832833
WT>TPPdeltaARE	Aldoc	-0.5204514	0.00036982	0.00705625
WT>TPPdeltaARE	Igsf3	-0.5216249	0.00396334	0.03851615
WT>TPPdeltaARE	Dennd2c	-0.53096	0.00462882	0.04323901
WT>TPPdeltaARE	3-Mar	-0.5311103	0.00181182	0.02190434
WT>TPPdeltaARE	B3gnt3	-0.5376014	0.00472439	0.04394002
WT>TPPdeltaARE	Kank2	-0.5410493	0.00348337	0.03510878
WT>TPPdeltaARE	Cask	-0.5429478	0.0006694	0.0108312
WT>TPPdeltaARE	Myo15	-0.5430917	0.00429697	0.04103413
WT>TPPdeltaARE	Tigd5	-0.5480709	0.00412075	0.03970649
WT>TPPdeltaARE	Serpinc1	-0.5506464	0.00232472	0.0263372
WT>TPPdeltaARE	Slco3a1	-0.5517891	0.00038163	0.00721717
WT>TPPdeltaARE	Dnaaf3	-0.5524237	0.0004148	0.00757681
WT>TPPdeltaARE	Snx7	-0.5528626	0.00192739	0.02297009
WT>TPPdeltaARE	Zc3h6	-0.5553306	0.004128	0.03973856
WT>TPPdeltaARE	Car7	-0.5565891	0.00548578	0.04883045
WT>TPPdeltaARE	Mmp16	-0.5665028	0.00129017	0.01719421
WT>TPPdeltaARE	Dusp22	-0.5716903	0.00324561	0.03341136
WT>TPPdeltaARE	Dmd	-0.5718322	0.00047869	0.00840914
WT>TPPdeltaARE	Fam135a	-0.573298	0.00122023	0.01647631
WT>TPPdeltaARE	Nlrx1	-0.573363	0.00173403	0.0213387
WT>TPPdeltaARE	Nipal2	-0.5768683	0.00215814	0.02505394
WT>TPPdeltaARE	Igsf10	-0.577729	0.00098915	0.01413242
WT>TPPdeltaARE	Asb4	-0.5787354	0.00416516	0.03995091
WT>TPPdeltaARE	Dtwd2	-0.5827108	0.00040745	0.00749227
WT>TPPdeltaARE	Zfp711	-0.583517	0.00560486	0.04977475
WT>TPPdeltaARE	Speg	-0.5857922	0.00086824	0.0130006
WT>TPPdeltaARE	Zfp948	-0.5920146	0.00224114	0.02571889
WT>TPPdeltaARE	Podxl	-0.5932126	0.00237141	0.02668923
WT>TPPdeltaARE	Nav1	-0.5933703	0.0016443	0.02046618
WT>TPPdeltaARE	Epb41l5	-0.5955337	0.00107289	0.01493908
WT>TPPdeltaARE	Gp1ba	-0.5958317	0.00026424	0.00551706
WT>TPPdeltaARE	Sema6b	-0.5973482	0.00461479	0.04314969
WT>TPPdeltaARE	Syn3	-0.603537	0.00257464	0.02825511
WT>TPPdeltaARE	Fgr	-0.6036989	0.00410164	0.0396481
WT>TPPdeltaARE	Rmi2	-0.6041205	0.00247642	0.02745862
WT>TPPdeltaARE	1700001G11	-0.6078593	0.00224547	0.02572678

WT>TPPdeltaARE	Ccdc78	-0.6085754	0.00412085	0.03970649
WT>TPPdeltaARE	Skida1	-0.6101916	0.0040452	0.03920404
WT>TPPdeltaARE	Rhoc	-0.6163544	0.00514846	0.04687844
WT>TPPdeltaARE	Arhgef28	-0.6188756	0.00087484	0.01305889
WT>TPPdeltaARE	Lpl	-0.6192325	0.00354889	0.03558307
WT>TPPdeltaARE	Fbn1	-0.6194306	0.00532618	0.04780658
WT>TPPdeltaARE	Ldlr	-0.6222868	0.00159637	0.02002864
WT>TPPdeltaARE	4933421O10	-0.6227145	0.00043378	0.00785661
WT>TPPdeltaARE	Slfn5	-0.6239855	0.00064356	0.01054025
WT>TPPdeltaARE	Celf4	-0.626038	0.00195981	0.02325582
WT>TPPdeltaARE	Ncam1	-0.6263669	0.00036041	0.00693845
WT>TPPdeltaARE	Utp14b	-0.6300805	0.0049033	0.04523285
WT>TPPdeltaARE	Sec14l2	-0.63211	0.0004492	0.00803909
WT>TPPdeltaARE	Stk36	-0.6324096	0.0026466	0.02881504
WT>TPPdeltaARE	1700025G04	-0.6357641	0.00021494	0.00469619
WT>TPPdeltaARE	Met	-0.6381409	0.00174561	0.0214129
WT>TPPdeltaARE	4430402I18R	-0.6411081	0.00121863	0.01646853
WT>TPPdeltaARE	Auts2	-0.643027	0.0006077	0.01008248
WT>TPPdeltaARE	Jun	-0.6433959	0.00015156	0.00364173
WT>TPPdeltaARE	Upp2	-0.6437899	0.00169418	0.02094202
WT>TPPdeltaARE	2900092N22	-0.6449465	0.00361624	0.03608201
WT>TPPdeltaARE	Garnl3	-0.6471808	0.00361773	0.03608201
WT>TPPdeltaARE	Specc1	-0.6472331	1.92E-05	0.00083298
WT>TPPdeltaARE	Gm45435	-0.6544219	0.0053099	0.04772198
WT>TPPdeltaARE	Gm43379	-0.6558246	0.0034142	0.03455625
WT>TPPdeltaARE	Ryr3	-0.6569939	0.00148693	0.01902693
WT>TPPdeltaARE	Myl4	-0.6570042	0.0008184	0.012438
WT>TPPdeltaARE	Pcdhb17	-0.657328	0.00411119	0.03967291
WT>TPPdeltaARE	Ly96	-0.6575064	8.03E-05	0.00232325
WT>TPPdeltaARE	Tesmin	-0.6599753	0.00410546	0.0396481
WT>TPPdeltaARE	Rasal2	-0.6608491	0.00053166	0.0091479
WT>TPPdeltaARE	Gp5	-0.660928	0.00163573	0.02041596
WT>TPPdeltaARE	Mpv17l	-0.6612948	0.00516152	0.04689655
WT>TPPdeltaARE	Gm19409	-0.6618823	0.00391695	0.03822937
WT>TPPdeltaARE	Ank3	-0.6629972	0.00166595	0.02067258
WT>TPPdeltaARE	Grm1	-0.6644775	0.00250177	0.02769192
WT>TPPdeltaARE	Unc79	-0.6647023	0.001769	0.0215353
WT>TPPdeltaARE	Epha7	-0.6648862	0.00101648	0.01438228
WT>TPPdeltaARE	Exoc3l2	-0.6651866	0.00398079	0.03866409
WT>TPPdeltaARE	Calml4	-0.6653214	0.00088243	0.01311126
WT>TPPdeltaARE	B930082K07	-0.6653703	0.00083879	0.01265802
WT>TPPdeltaARE	Fam229b	-0.6657994	0.00066831	0.01082274
WT>TPPdeltaARE	4833412C15I	-0.6664236	0.00348692	0.03512622
WT>TPPdeltaARE	Slc1a3	-0.6664622	0.00118921	0.01622983
WT>TPPdeltaARE	4930432K21I	-0.6679205	0.00376214	0.03712247

WT>TPPdeltaARE	Slc25a47	-0.6682674	0.00141112	0.01831225
WT>TPPdeltaARE	Zfp334	-0.6683572	0.0013986	0.0181988
WT>TPPdeltaARE	Kank3	-0.6689447	0.00545582	0.04867385
WT>TPPdeltaARE	Tspoap1	-0.6695197	0.00518904	0.04707033
WT>TPPdeltaARE	Lyplal1	-0.6718288	0.00053922	0.00924169
WT>TPPdeltaARE	Bambi	-0.6750673	0.00115526	0.01584557
WT>TPPdeltaARE	Clnk	-0.6751256	0.00183426	0.02210627
WT>TPPdeltaARE	Gm48342	-0.6753942	0.00095748	0.01383184
WT>TPPdeltaARE	Igf2bp3	-0.6760413	0.00043905	0.00792959
WT>TPPdeltaARE	Fcgrt	-0.6761389	0.00134484	0.01772674
WT>TPPdeltaARE	Gm38248	-0.6803121	0.00039196	0.00729457
WT>TPPdeltaARE	Disc1	-0.6804738	0.00269468	0.02915734
WT>TPPdeltaARE	Gm42798	-0.6826073	0.00506581	0.04633661
WT>TPPdeltaARE	Hs3st1	-0.6869849	0.00014328	0.00350408
WT>TPPdeltaARE	D430020J02I	-0.6874466	0.00097298	0.01398233
WT>TPPdeltaARE	Dhrs3	-0.6895701	0.00059045	0.00990723
WT>TPPdeltaARE	Gm43769	-0.691961	0.00394878	0.03843277
WT>TPPdeltaARE	Bmpr1a	-0.6969513	0.00072174	0.01142868
WT>TPPdeltaARE	Klhl32	-0.6975125	0.00391796	0.03822937
WT>TPPdeltaARE	Epb41l4a	-0.6978032	0.00252483	0.0278512
WT>TPPdeltaARE	D130007C19	-0.6982152	0.00396328	0.03851615
WT>TPPdeltaARE	Junos	-0.6983336	0.00052259	0.00904032
WT>TPPdeltaARE	Etv1	-0.6997782	0.0024512	0.02732394
WT>TPPdeltaARE	Ccdc30	-0.7000063	0.00051277	0.00889423
WT>TPPdeltaARE	Tdo2	-0.7007809	0.00469384	0.04367696
WT>TPPdeltaARE	4930512B01	-0.7007823	0.00037859	0.00717382
WT>TPPdeltaARE	Gm12802	-0.701313	0.00356921	0.03573102
WT>TPPdeltaARE	Dsc2	-0.7018756	0.00096813	0.01393766
WT>TPPdeltaARE	Hectd2os	-0.701947	0.00051055	0.0088717
WT>TPPdeltaARE	Kcnj2	-0.7024916	0.00203346	0.02390898
WT>TPPdeltaARE	Tulp3	-0.7025106	0.0017247	0.02126471
WT>TPPdeltaARE	A630072L19I	-0.7080181	0.00452995	0.04270031
WT>TPPdeltaARE	Gm49309	-0.709231	0.00415101	0.03987743
WT>TPPdeltaARE	Gm44567	-0.7107652	0.00529697	0.04765546
WT>TPPdeltaARE	Gm10269	-0.7121542	0.00228994	0.02605046
WT>TPPdeltaARE	Gm49783	-0.7124673	0.00022819	0.00493236
WT>TPPdeltaARE	Hist1h1d	-0.7138255	0.00250887	0.02773859
WT>TPPdeltaARE	Csf2rb	-0.7144865	3.31E-06	0.00024713
WT>TPPdeltaARE	Cacna1e	-0.7157326	0.00104436	0.01466905
WT>TPPdeltaARE	Olfr920	-0.7173734	0.00513001	0.04676458
WT>TPPdeltaARE	Gm5532	-0.7183492	0.00158087	0.01988578
WT>TPPdeltaARE	Gm42467	-0.7238392	0.00099165	0.01413242
WT>TPPdeltaARE	Tulp1	-0.7249314	0.00428257	0.04093724
WT>TPPdeltaARE	Tbc1d30	-0.7263403	0.00174028	0.02136106
WT>TPPdeltaARE	Prlr	-0.7282674	0.00291665	0.03086587

WT>TPPdeltaARE	Gm38042	-0.7367254	0.00260474	0.02852039
WT>TPPdeltaARE	B930095G15	-0.7380857	0.00172355	0.02126412
WT>TPPdeltaARE	Gm7598	-0.7381814	0.00319752	0.03298687
WT>TPPdeltaARE	Gm37125	-0.7394063	0.00142191	0.01841501
WT>TPPdeltaARE	Gm44677	-0.7397677	0.00201416	0.02378361
WT>TPPdeltaARE	Hydin	-0.7437834	0.00303673	0.03177023
WT>TPPdeltaARE	Adam28	-0.7450807	0.00526359	0.04748817
WT>TPPdeltaARE	Gm30346	-0.7464886	0.00245331	0.02732817
WT>TPPdeltaARE	Gm16548	-0.7474489	4.05E-05	0.00140992
WT>TPPdeltaARE	Gm37968	-0.7479134	0.00379474	0.03738675
WT>TPPdeltaARE	Mmp14	-0.7479675	4.14E-06	0.00029197
WT>TPPdeltaARE	1700067K01I	-0.7482059	0.00518381	0.04707033
WT>TPPdeltaARE	Akr1c14	-0.748767	0.00295239	0.03112836
WT>TPPdeltaARE	Ppm1e	-0.7497548	0.0018907	0.02264486
WT>TPPdeltaARE	Dtna	-0.7502235	0.00108811	0.01508559
WT>TPPdeltaARE	Ston2	-0.7505809	0.00048814	0.00853617
WT>TPPdeltaARE	Mmp2	-0.7512307	0.00056168	0.00954509
WT>TPPdeltaARE	Acot3	-0.7523201	0.00074834	0.01171509
WT>TPPdeltaARE	Gm47111	-0.7525916	0.00538816	0.04820576
WT>TPPdeltaARE	Lrrc74b	-0.7540273	0.00273663	0.02947881
WT>TPPdeltaARE	Fhdc1	-0.7547192	0.00190221	0.02276865
WT>TPPdeltaARE	Gm47754	-0.755023	0.00305267	0.03188508
WT>TPPdeltaARE	Nup210l	-0.7598236	0.00034864	0.00681402
WT>TPPdeltaARE	Tenm4	-0.7610949	0.00075883	0.01181214
WT>TPPdeltaARE	6430548M08	-0.7611332	0.00139742	0.0181988
WT>TPPdeltaARE	Gm4258	-0.7616697	0.00152789	0.01939659
WT>TPPdeltaARE	Tnfrsf11a	-0.761712	0.0013215	0.01752402
WT>TPPdeltaARE	Vamp9	-0.7627488	0.00190701	0.02280926
WT>TPPdeltaARE	Acacb	-0.7632804	0.00038671	0.00726351
WT>TPPdeltaARE	Gm42735	-0.7636541	0.00053951	0.00924169
WT>TPPdeltaARE	Dnhd1	-0.7667441	0.00546032	0.04867385
WT>TPPdeltaARE	Zfp985	-0.7679792	7.47E-05	0.00221412
WT>TPPdeltaARE	Gm7890	-0.7685195	0.00449688	0.04255193
WT>TPPdeltaARE	Antxr1	-0.7685457	0.00501787	0.04606921
WT>TPPdeltaARE	Rian	-0.7691916	0.00073993	0.01164377
WT>TPPdeltaARE	Notch4	-0.7693781	0.0030673	0.03198588
WT>TPPdeltaARE	Ltc4s	-0.769654	0.00106776	0.01490356
WT>TPPdeltaARE	Tmem108	-0.7699515	0.00330414	0.03382028
WT>TPPdeltaARE	Plce1	-0.7700468	0.00154891	0.01962462
WT>TPPdeltaARE	Cubn	-0.7704814	0.00079325	0.01219032
WT>TPPdeltaARE	Zfp112	-0.7714723	0.00341312	0.03455625
WT>TPPdeltaARE	Cacna1c	-0.7746228	0.00035042	0.00683694
WT>TPPdeltaARE	Dsg2	-0.7754763	1.11E-05	0.00054632
WT>TPPdeltaARE	Gm26601	-0.7756733	0.00193094	0.02297427
WT>TPPdeltaARE	Tnfaip8l1	-0.776743	0.00099223	0.01413242

WT>TPPdeltaARE	Il1r1	-0.776767	1.99E-05	0.00085031
WT>TPPdeltaARE	5830487J09F	-0.7783206	0.004311	0.0411274
WT>TPPdeltaARE	Cplx2	-0.7786279	0.00017988	0.00414643
WT>TPPdeltaARE	Acox2	-0.7795628	0.00186296	0.0223615
WT>TPPdeltaARE	Rnf17	-0.7806368	0.00028216	0.00582171
WT>TPPdeltaARE	Atp9a	-0.7811783	0.00016821	0.00393871
WT>TPPdeltaARE	Ttll13	-0.7831975	0.00425627	0.04072624
WT>TPPdeltaARE	4930532G15	-0.7835035	0.00089496	0.01323613
WT>TPPdeltaARE	Camsap2	-0.7842115	0.00052183	0.00903523
WT>TPPdeltaARE	Dnah2	-0.7850981	0.00491181	0.04526807
WT>TPPdeltaARE	Angpt2	-0.7864702	0.00169749	0.02096941
WT>TPPdeltaARE	Meis2	-0.7871765	0.00064376	0.01054025
WT>TPPdeltaARE	Col19a1	-0.7888974	0.00451243	0.04262338
WT>TPPdeltaARE	Plod1	-0.7895733	0.00011455	0.00298704
WT>TPPdeltaARE	Scn1a	-0.790343	0.00328261	0.03366606
WT>TPPdeltaARE	AC160336.1	-0.790747	0.00112824	0.01551909
WT>TPPdeltaARE	Adra1a	-0.7914318	0.00288034	0.03059921
WT>TPPdeltaARE	Tln2	-0.7938763	0.00118013	0.01612927
WT>TPPdeltaARE	Tsnaxip1	-0.7943752	0.00147019	0.01890058
WT>TPPdeltaARE	D930020B18	-0.7945653	0.00319119	0.03295677
WT>TPPdeltaARE	Cpe	-0.7952295	0.00253724	0.02793237
WT>TPPdeltaARE	Socs3	-0.7953552	0.00021348	0.00466959
WT>TPPdeltaARE	Clmn	-0.7961355	0.00203121	0.02390898
WT>TPPdeltaARE	Gm37101	-0.7981477	0.00216086	0.02507035
WT>TPPdeltaARE	Aox1	-0.7986938	0.00155946	0.01969131
WT>TPPdeltaARE	Muc6	-0.8019466	0.00533953	0.04788187
WT>TPPdeltaARE	Fau-ps2	-0.8053025	0.00052481	0.00907046
WT>TPPdeltaARE	Shroom1	-0.8063248	9.84E-05	0.00269775
WT>TPPdeltaARE	Ajuba	-0.8071071	0.00013094	0.00327739
WT>TPPdeltaARE	Gm21284	-0.8098486	0.00340444	0.0345118
WT>TPPdeltaARE	Ahrr	-0.8103564	0.00121334	0.0164317
WT>TPPdeltaARE	Tspan8	-0.81119	0.00198574	0.02349116
WT>TPPdeltaARE	3110080O07	-0.8129833	0.00084603	0.01273741
WT>TPPdeltaARE	Dscam	-0.8131722	0.00504924	0.04629107
WT>TPPdeltaARE	Minar2	-0.8140961	0.00155896	0.01969131
WT>TPPdeltaARE	Card10	-0.8142687	0.00072605	0.01147809
WT>TPPdeltaARE	Shcbp1l	-0.8144402	0.0004773	0.00840769
WT>TPPdeltaARE	Gm36963	-0.8172271	0.0012833	0.01713822
WT>TPPdeltaARE	Gm43609	-0.8176404	0.00156302	0.01969977
WT>TPPdeltaARE	Abca4	-0.8179241	0.00010774	0.00288373
WT>TPPdeltaARE	Cttnbp2	-0.8181164	0.0017555	0.02149316
WT>TPPdeltaARE	Gm37233	-0.8191428	0.00089778	0.01324837
WT>TPPdeltaARE	Opcml	-0.8207339	0.00238525	0.0267915
WT>TPPdeltaARE	D7Ertd443e	-0.8212481	0.00528517	0.04761598
WT>TPPdeltaARE	Efemp2	-0.8216025	0.00032299	0.00644999

WT>TPPdeltaARE	Zfp9	-0.822547	0.00267827	0.02905929
WT>TPPdeltaARE	Iqch	-0.8227163	0.00278436	0.02984497
WT>TPPdeltaARE	Csmd2	-0.8241698	0.00328102	0.03366606
WT>TPPdeltaARE	Pak7	-0.8244881	0.00053347	0.00917087
WT>TPPdeltaARE	Fmn1	-0.8246055	0.00090801	0.01332692
WT>TPPdeltaARE	Cux2	-0.8257765	0.0005589	0.00950619
WT>TPPdeltaARE	Spef2	-0.8302838	5.85E-05	0.00185723
WT>TPPdeltaARE	Stamos	-0.8311854	0.0002443	0.00519067
WT>TPPdeltaARE	Lgr6	-0.8330941	0.00076351	0.01185628
WT>TPPdeltaARE	A430073D23	-0.8341675	0.00410319	0.0396481
WT>TPPdeltaARE	Dscaml1	-0.8345254	0.00483115	0.0447601
WT>TPPdeltaARE	Enpp3	-0.8371795	0.00227381	0.02594354
WT>TPPdeltaARE	Edaradd	-0.8392858	0.00033103	0.00656976
WT>TPPdeltaARE	Cdr2l	-0.8396694	0.00040144	0.00741728
WT>TPPdeltaARE	Slc38a11	-0.8403163	0.00070157	0.01117349
WT>TPPdeltaARE	Gm38352	-0.8422158	0.00288967	0.03066106
WT>TPPdeltaARE	Timp3	-0.8431917	0.00096843	0.01393766
WT>TPPdeltaARE	I830077J02R	-0.8432479	0.00203797	0.02394735
WT>TPPdeltaARE	3110039I08R	-0.8432801	0.00222141	0.02554227
WT>TPPdeltaARE	Dapk2	-0.8434003	0.00345604	0.03492466
WT>TPPdeltaARE	Sh3rf2	-0.8436064	0.00216759	0.02511826
WT>TPPdeltaARE	Syt1	-0.8439991	0.00368487	0.03654713
WT>TPPdeltaARE	Ryr2	-0.8455251	0.00094532	0.01370753
WT>TPPdeltaARE	Adamts12	-0.8455982	0.0013231	0.01752402
WT>TPPdeltaARE	Xist	-0.8477905	0.00357249	0.03574526
WT>TPPdeltaARE	D230049E03	-0.8479244	0.00125761	0.01688877
WT>TPPdeltaARE	Ggt5	-0.8490192	3.73E-05	0.00133955
WT>TPPdeltaARE	Gprc5c	-0.8510763	0.00186991	0.02242375
WT>TPPdeltaARE	Csf2rb2	-0.8520602	0.00011921	0.00306309
WT>TPPdeltaARE	Lingo2	-0.8520642	0.00371349	0.03677422
WT>TPPdeltaARE	Slc2a13	-0.8521863	0.0018565	0.02233243
WT>TPPdeltaARE	Gas7	-0.8562892	0.00139816	0.0181988
WT>TPPdeltaARE	Gm5514	-0.8587164	0.00070795	0.01124721
WT>TPPdeltaARE	Akap6	-0.8598907	0.00467096	0.04354815
WT>TPPdeltaARE	Gm38292	-0.8599909	0.00262042	0.02859467
WT>TPPdeltaARE	Vmn2r-ps20	-0.8616663	0.00082126	0.01246357
WT>TPPdeltaARE	Rhoj	-0.8623288	4.80E-08	1.12E-05
WT>TPPdeltaARE	Slc4a11	-0.8625088	0.00312294	0.03247215
WT>TPPdeltaARE	Scn2a	-0.8626106	0.00080578	0.01228814
WT>TPPdeltaARE	Bmf	-0.8632218	0.0003383	0.00667283
WT>TPPdeltaARE	Rai2	-0.8634918	0.00210041	0.02450182
WT>TPPdeltaARE	Grip1	-0.8635915	0.00437704	0.0416543
WT>TPPdeltaARE	A530041M06	-0.8654499	6.19E-05	0.00193159
WT>TPPdeltaARE	Skint3	-0.8654541	1.71E-05	0.00076423
WT>TPPdeltaARE	Gm45130	-0.8655034	0.00021833	0.00475414

WT>TPPdeltaARE	Gm49883	-0.8676319	0.00167679	0.02076703
WT>TPPdeltaARE	Spats2l	-0.8683845	0.00369977	0.03665719
WT>TPPdeltaARE	Lmo7	-0.8700554	0.00225908	0.025837
WT>TPPdeltaARE	Gm43162	-0.8705731	0.00039179	0.00729457
WT>TPPdeltaARE	Syt12	-0.8709212	0.00095921	0.01384636
WT>TPPdeltaARE	Arhgap20	-0.8734255	0.00102189	0.01442697
WT>TPPdeltaARE	Lama2	-0.8755487	0.00331525	0.03387492
WT>TPPdeltaARE	Vash2	-0.8758024	0.00223222	0.02565122
WT>TPPdeltaARE	Efcab5	-0.8760649	0.00048805	0.00853617
WT>TPPdeltaARE	Gli2	-0.8797151	0.00133437	0.01763697
WT>TPPdeltaARE	Tenm2	-0.8803473	0.0004765	0.00840135
WT>TPPdeltaARE	Dlgap1	-0.8803875	0.00056748	0.00963171
WT>TPPdeltaARE	Gm37124	-0.8808325	0.00031469	0.0063495
WT>TPPdeltaARE	Sall3	-0.8818886	0.00210179	0.02450304
WT>TPPdeltaARE	Bicc1	-0.8835357	0.00368171	0.03653466
WT>TPPdeltaARE	AI463229	-0.8844831	0.00134882	0.0177306
WT>TPPdeltaARE	Gm45555	-0.8846788	0.0001531	0.00365809
WT>TPPdeltaARE	B430010I23F	-0.8861663	7.15E-05	0.00214999
WT>TPPdeltaARE	Slco2a1	-0.8868704	6.53E-07	8.23E-05
WT>TPPdeltaARE	Gm44027	-0.8879199	0.00087721	0.013074
WT>TPPdeltaARE	Gm43571	-0.8881653	0.00289093	0.03066106
WT>TPPdeltaARE	Slc6a1	-0.8882353	0.00439909	0.0417816
WT>TPPdeltaARE	Fam20a	-0.888459	0.00041822	0.00761764
WT>TPPdeltaARE	Gm4117	-0.8892299	5.61E-06	0.00035044
WT>TPPdeltaARE	Sv2b	-0.8896389	0.00244406	0.0272882
WT>TPPdeltaARE	Veph1	-0.8904838	0.00010689	0.00287285
WT>TPPdeltaARE	Adamtsl1	-0.8906326	0.00206359	0.02413869
WT>TPPdeltaARE	Usp35	-0.8907832	0.00075402	0.01176578
WT>TPPdeltaARE	Gm2155	-0.8920479	0.00060038	0.00999125
WT>TPPdeltaARE	Slc10a1	-0.89208	0.00040912	0.00750875
WT>TPPdeltaARE	Itga1	-0.892555	0.00069416	0.01108305
WT>TPPdeltaARE	Dmtn	-0.8937798	0.00088976	0.01317945
WT>TPPdeltaARE	Dnah10	-0.8970591	0.00099147	0.01413242
WT>TPPdeltaARE	Rnase4	-0.8974419	0.00186356	0.0223615
WT>TPPdeltaARE	Robo1	-0.9004335	0.00061313	0.01014213
WT>TPPdeltaARE	Clmp	-0.901452	0.0008041	0.0122791
WT>TPPdeltaARE	Sema3f	-0.9042592	0.00451647	0.04262338
WT>TPPdeltaARE	Atp6v1c2	-0.9063056	0.00145306	0.01874287
WT>TPPdeltaARE	Mpo	-0.9071861	0.00032823	0.00653441
WT>TPPdeltaARE	Zfp947	-0.9074709	0.00065694	0.01071062
WT>TPPdeltaARE	Gm14508	-0.9075024	0.00287827	0.03059409
WT>TPPdeltaARE	Car10	-0.907713	0.00143324	0.01852444
WT>TPPdeltaARE	A930024N18	-0.9078758	0.00032234	0.00644377
WT>TPPdeltaARE	Adamts9	-0.9080726	0.00011575	0.00300204
WT>TPPdeltaARE	Sycp2l	-0.9091274	0.00058315	0.00983186

WT>TPPdeltaARE	Gm50012	-0.9104047	0.00022893	0.00494026
WT>TPPdeltaARE	Gm5833	-0.911099	1.62E-05	0.00073772
WT>TPPdeltaARE	Ptprg	-0.9127025	0.00026942	0.00559481
WT>TPPdeltaARE	Selp	-0.9128292	8.56E-08	1.63E-05
WT>TPPdeltaARE	Olfml2a	-0.9140061	0.00107196	0.01493908
WT>TPPdeltaARE	She	-0.9148331	0.00127292	0.01703508
WT>TPPdeltaARE	Guca1b	-0.9162454	9.07E-05	0.00254787
WT>TPPdeltaARE	Fat3	-0.9173766	0.0001507	0.00363006
WT>TPPdeltaARE	Pax6	-0.9185155	0.00062641	0.01032635
WT>TPPdeltaARE	Megf11	-0.9197502	0.00020168	0.00449828
WT>TPPdeltaARE	Acvr1c	-0.9209366	0.00020641	0.00455921
WT>TPPdeltaARE	B230216N24	-0.9219971	2.28E-05	0.00094553
WT>TPPdeltaARE	Nrg1	-0.9223558	0.0004508	0.00804796
WT>TPPdeltaARE	Lin28b	-0.9260896	0.00059271	0.00992372
WT>TPPdeltaARE	Duxf3	-0.9270206	0.00538492	0.04819913
WT>TPPdeltaARE	Gm37204	-0.9283434	6.50E-05	0.00200031
WT>TPPdeltaARE	Ffar2	-0.9292631	1.49E-05	0.00069151
WT>TPPdeltaARE	BC051142	-0.9302971	6.15E-05	0.00192027
WT>TPPdeltaARE	Gm16587	-0.9309433	1.68E-07	2.98E-05
WT>TPPdeltaARE	Scn8a	-0.9323738	0.00506465	0.04633661
WT>TPPdeltaARE	Osbp2	-0.9334459	4.97E-05	0.00163323
WT>TPPdeltaARE	Slc7a2	-0.9356401	7.71E-05	0.0022579
WT>TPPdeltaARE	Svep1	-0.9357976	0.00020635	0.00455921
WT>TPPdeltaARE	Lrp3	-0.9360009	0.00031794	0.00637272
WT>TPPdeltaARE	Pm20d1	-0.936848	8.42E-05	0.00241133
WT>TPPdeltaARE	Ugt1a6a	-0.9371115	8.53E-05	0.00243116
WT>TPPdeltaARE	Tmcc3	-0.9376357	2.93E-05	0.00113301
WT>TPPdeltaARE	Adgb	-0.9402223	0.00037762	0.00716823
WT>TPPdeltaARE	Gm13483	-0.9404019	9.39E-05	0.00259949
WT>TPPdeltaARE	Olfr77	-0.9415137	0.00018193	0.00416883
WT>TPPdeltaARE	Kcnk9	-0.9416025	0.00041737	0.00760932
WT>TPPdeltaARE	C230071H17	-0.9424938	6.42E-05	0.00198216
WT>TPPdeltaARE	Gm44095	-0.9441144	0.0001458	0.00354309
WT>TPPdeltaARE	Gm37470	-0.9441494	0.00119293	0.01625807
WT>TPPdeltaARE	Myt1l	-0.9443313	0.00112269	0.0154538
WT>TPPdeltaARE	Nrl	-0.944416	0.00530827	0.04772198
WT>TPPdeltaARE	Epas1	-0.9456738	0.00098647	0.01411303
WT>TPPdeltaARE	Prkd1	-0.9468338	0.00074499	0.0116816
WT>TPPdeltaARE	Trpm1	-0.9486876	0.00077243	0.01195628
WT>TPPdeltaARE	Ifi203-ps	-0.9488365	7.38E-06	0.00041508
WT>TPPdeltaARE	Lhx4	-0.9488769	0.00281129	0.03003123
WT>TPPdeltaARE	Ctsl	-0.9489621	0.00116945	0.016006
WT>TPPdeltaARE	Usp29	-0.9515485	0.00273423	0.02946941
WT>TPPdeltaARE	Cspg5	-0.9536801	0.00219439	0.02532219
WT>TPPdeltaARE	Abca8b	-0.9542449	4.29E-05	0.00144886

WT>TPPdeltaARE	Tshb	-0.9555763	0.00133672	0.01764205
WT>TPPdeltaARE	Gm34589	-0.9579022	0.00050381	0.00878636
WT>TPPdeltaARE	Raph1	-0.9591711	1.41E-05	0.00066602
WT>TPPdeltaARE	Rassf9	-0.9605597	0.0005494	0.00935284
WT>TPPdeltaARE	Ptprk	-0.9607641	4.39E-06	0.00030322
WT>TPPdeltaARE	Id3	-0.9616635	0.00302351	0.03166635
WT>TPPdeltaARE	Zcchc14	-0.9638469	2.67E-06	0.00020983
WT>TPPdeltaARE	Sfxn5	-0.9642407	4.61E-06	0.00031188
WT>TPPdeltaARE	Gm15666	-0.9644083	6.49E-05	0.00200031
WT>TPPdeltaARE	Gdap1	-0.966361	0.00013554	0.003372
WT>TPPdeltaARE	Cc2d2b	-0.967395	0.00011426	0.00298371
WT>TPPdeltaARE	Gm29340	-0.9680824	0.00014177	0.00348037
WT>TPPdeltaARE	Adgrl3	-0.9683062	0.00231835	0.02629604
WT>TPPdeltaARE	AW822073	-0.9685163	0.00044928	0.00803909
WT>TPPdeltaARE	Gm26710	-0.9685258	0.00151021	0.01923548
WT>TPPdeltaARE	Gm43560	-0.9689702	0.00014804	0.00357941
WT>TPPdeltaARE	Sis	-0.9700465	9.23E-05	0.00257133
WT>TPPdeltaARE	Tcaf2	-0.9704977	2.06E-05	0.00087086
WT>TPPdeltaARE	Armcx4	-0.9708807	0.00050787	0.0088412
WT>TPPdeltaARE	Lgr5	-0.9709959	0.000222	0.0048151
WT>TPPdeltaARE	Unc80	-0.971573	0.00059596	0.00994349
WT>TPPdeltaARE	Ces4a	-0.9773358	0.00027818	0.00574578
WT>TPPdeltaARE	Emp1	-0.9776504	0.00102366	0.01444143
WT>TPPdeltaARE	Ank2	-0.9778121	2.73E-05	0.00108356
WT>TPPdeltaARE	S1pr3	-0.9778725	8.94E-06	0.00048367
WT>TPPdeltaARE	Foxp2	-0.977888	5.91E-06	0.00036148
WT>TPPdeltaARE	Cfap69	-0.9780622	0.00093045	0.01354284
WT>TPPdeltaARE	Trim2	-0.979306	4.76E-07	6.55E-05
WT>TPPdeltaARE	Pla2r1	-0.9798297	1.96E-05	0.0008388
WT>TPPdeltaARE	Ttn	-0.9809271	3.07E-09	1.31E-06
WT>TPPdeltaARE	Il17rd	-0.983579	0.00039609	0.00733945
WT>TPPdeltaARE	Adam23	-0.9836388	5.99E-05	0.00188552
WT>TPPdeltaARE	Prkg2	-0.9839675	0.00018902	0.00429045
WT>TPPdeltaARE	Slc18a1	-0.9843895	0.00439642	0.0417816
WT>TPPdeltaARE	Gm11639	-0.984607	3.06E-05	0.00116786
WT>TPPdeltaARE	4933438K21l	-0.985852	0.00105447	0.01476793
WT>TPPdeltaARE	Spats2	-0.9874318	1.44E-05	0.00067194
WT>TPPdeltaARE	Smok2b	-0.989659	0.00043236	0.00783816
WT>TPPdeltaARE	Tead1	-0.990121	0.00016148	0.0038182
WT>TPPdeltaARE	Gm28720	-0.9933439	0.00108969	0.01509656
WT>TPPdeltaARE	Epha4	-0.9934244	0.00014462	0.00352334
WT>TPPdeltaARE	Ptprm	-0.9954036	0.00020432	0.00454136
WT>TPPdeltaARE	Nek11	-0.9965819	0.00024999	0.0052766
WT>TPPdeltaARE	Gm16192	-0.9974466	0.00058476	0.00985051
WT>TPPdeltaARE	Gm44199	-1.0004828	9.25E-06	0.00049241

WT>TPPdeltaARE	Cdh6	-1.0052791	0.00091998	0.01345066
WT>TPPdeltaARE	Vmn2r53	-1.0061736	0.00021091	0.00462918
WT>TPPdeltaARE	Npas3	-1.006281	1.03E-06	0.00010932
WT>TPPdeltaARE	Magi1	-1.0068236	0.00019679	0.00442517
WT>TPPdeltaARE	Cnr1	-1.0072683	0.00059582	0.00994349
WT>TPPdeltaARE	Plscr2	-1.0075549	2.08E-06	0.00017764
WT>TPPdeltaARE	Cecr2	-1.0088005	1.51E-06	0.00014307
WT>TPPdeltaARE	Lama3	-1.0100975	0.000105	0.00283802
WT>TPPdeltaARE	Kcnma1	-1.0104675	0.00035565	0.00688992
WT>TPPdeltaARE	Enkur	-1.0105086	8.21E-05	0.00236903
WT>TPPdeltaARE	Gm48611	-1.0117865	0.00166497	0.02067258
WT>TPPdeltaARE	Lonrf2	-1.0132669	0.00014103	0.0034723
WT>TPPdeltaARE	Hand2os1	-1.0136411	0.00038721	0.00726565
WT>TPPdeltaARE	Gm26522	-1.015642	2.65E-05	0.0010629
WT>TPPdeltaARE	Gm48855	-1.0213669	0.00011048	0.00292843
WT>TPPdeltaARE	5830416I19R	-1.0219005	0.0004737	0.00837276
WT>TPPdeltaARE	Me3	-1.0233753	0.00039233	0.00729457
WT>TPPdeltaARE	Fn1	-1.0234659	3.28E-05	0.00122754
WT>TPPdeltaARE	Ifi204	-1.0238444	2.24E-05	0.00093584
WT>TPPdeltaARE	Map1b	-1.0239797	1.40E-05	0.00066131
WT>TPPdeltaARE	Flnc	-1.0256963	0.00044489	0.00799016
WT>TPPdeltaARE	Tspan18	-1.0279778	3.64E-05	0.00132292
WT>TPPdeltaARE	Celf3	-1.0307214	0.00022213	0.0048151
WT>TPPdeltaARE	Zfp300	-1.0310604	4.38E-05	0.00147377
WT>TPPdeltaARE	Hist1h2ac	-1.0349994	9.91E-06	0.00050672
WT>TPPdeltaARE	Ppp2r2b	-1.0355994	6.69E-05	0.0020421
WT>TPPdeltaARE	Dnah9	-1.0374229	0.00039233	0.00729457
WT>TPPdeltaARE	Gm46136	-1.0378408	0.00023808	0.00510353
WT>TPPdeltaARE	Olfml3	-1.0388368	2.30E-05	0.00095001
WT>TPPdeltaARE	1700018A04	-1.0408765	0.0002504	0.0052794
WT>TPPdeltaARE	Elavl3	-1.0452164	0.00267355	0.02905004
WT>TPPdeltaARE	Pik3c2g	-1.0466845	0.00060795	0.01008248
WT>TPPdeltaARE	Pdgfrl	-1.0482075	9.50E-05	0.0026222
WT>TPPdeltaARE	Ldb2	-1.0494556	0.00120873	0.01642682
WT>TPPdeltaARE	A4galt	-1.0505927	0.00106802	0.01490356
WT>TPPdeltaARE	Mgam	-1.0515804	0.00022613	0.00489634
WT>TPPdeltaARE	Rtn4rl1	-1.0527984	9.02E-05	0.00253704
WT>TPPdeltaARE	D130043K22	-1.0535233	0.00143247	0.01852444
WT>TPPdeltaARE	Vmn2r57	-1.0558198	0.00014257	0.00349099
WT>TPPdeltaARE	Otoa	-1.0579244	0.00011309	0.00296178
WT>TPPdeltaARE	Rbms3	-1.0588472	0.0001274	0.003222
WT>TPPdeltaARE	Zmat4	-1.0608617	0.00017849	0.00411934
WT>TPPdeltaARE	Gm14221	-1.0615604	0.00104197	0.01464604
WT>TPPdeltaARE	Irs1	-1.0666731	0.00026829	0.00558627
WT>TPPdeltaARE	Homer2	-1.0695883	0.00074395	0.0116748

WT>TPPdeltaARE	Wscd2	-1.0700195	8.75E-05	0.0024725
WT>TPPdeltaARE	10-Sep	-1.0706718	4.17E-05	0.0014321
WT>TPPdeltaARE	Bend6	-1.0713825	7.39E-05	0.00220205
WT>TPPdeltaARE	Hcn1	-1.0766139	9.96E-05	0.00272045
WT>TPPdeltaARE	Gnao1	-1.0766778	0.00026293	0.0054956
WT>TPPdeltaARE	Ccbe1	-1.0793195	0.00019475	0.00438941
WT>TPPdeltaARE	Nxpe2	-1.0797031	1.91E-06	0.000169
WT>TPPdeltaARE	Tshz2	-1.0830175	1.10E-05	0.00054442
WT>TPPdeltaARE	Caskin1	-1.0836965	0.00316043	0.03272682
WT>TPPdeltaARE	E430014B02	-1.0840945	6.42E-05	0.00198216
WT>TPPdeltaARE	Rbfox3	-1.0842208	0.00092151	0.01345329
WT>TPPdeltaARE	Arfgef3	-1.0845885	8.59E-06	0.00046655
WT>TPPdeltaARE	Abi3bp	-1.0857424	5.38E-06	0.00034122
WT>TPPdeltaARE	Dnah7b	-1.086108	3.91E-05	0.00137495
WT>TPPdeltaARE	Platr1	-1.0872876	0.00035594	0.00688992
WT>TPPdeltaARE	Pde7b	-1.0879665	9.69E-06	0.0004979
WT>TPPdeltaARE	Plpp3	-1.0892073	0.00011752	0.00303578
WT>TPPdeltaARE	Srgap1	-1.0899405	5.62E-06	0.00035044
WT>TPPdeltaARE	Epha3	-1.0900984	0.00018553	0.00422618
WT>TPPdeltaARE	Col12a1	-1.090223	0.00010314	0.00279943
WT>TPPdeltaARE	Cdc42ep2	-1.0912229	4.34E-05	0.00146453
WT>TPPdeltaARE	Gm47585	-1.0925609	2.25E-05	0.00093706
WT>TPPdeltaARE	Trpm3	-1.0931757	9.40E-08	1.78E-05
WT>TPPdeltaARE	Gk5	-1.0945299	4.77E-06	0.00031323
WT>TPPdeltaARE	Creb5	-1.098631	1.06E-05	0.00053131
WT>TPPdeltaARE	Col14a1	-1.1005628	0.00014353	0.00350564
WT>TPPdeltaARE	Id4	-1.1010193	0.00060412	0.01004489
WT>TPPdeltaARE	Zfp423	-1.1023157	2.52E-05	0.00102164
WT>TPPdeltaARE	Serpinh1	-1.1038886	1.43E-08	4.09E-06
WT>TPPdeltaARE	Cdr2	-1.1040449	2.37E-05	0.00097177
WT>TPPdeltaARE	Gm44369	-1.1096186	0.00025334	0.00533573
WT>TPPdeltaARE	Mpp2	-1.1125648	2.17E-06	0.00018185
WT>TPPdeltaARE	Grin2b	-1.1139992	0.00034193	0.00669641
WT>TPPdeltaARE	Megf6	-1.1146042	0.00361979	0.03608201
WT>TPPdeltaARE	Gm37706	-1.1166252	3.02E-05	0.00115706
WT>TPPdeltaARE	Dnase1l3	-1.1170805	0.00100383	0.01423232
WT>TPPdeltaARE	Phkg1	-1.1174642	9.98E-06	0.00050722
WT>TPPdeltaARE	Sdk2	-1.1185544	0.00012673	0.00321447
WT>TPPdeltaARE	Six4	-1.1206507	0.00086746	0.01299904
WT>TPPdeltaARE	Crispld2	-1.1226756	4.67E-06	0.00031323
WT>TPPdeltaARE	Myzap	-1.1236055	0.00010977	0.00291365
WT>TPPdeltaARE	Gm37985	-1.123955	4.75E-06	0.00031323
WT>TPPdeltaARE	5430419D17	-1.1287651	0.00330961	0.03383525
WT>TPPdeltaARE	Dock4	-1.1291184	1.92E-09	9.03E-07
WT>TPPdeltaARE	Fermt2	-1.1292362	3.34E-06	0.00024876

WT>TPPdeltaARE	Pdgfra	-1.1293854	6.12E-05	0.00191374
WT>TPPdeltaARE	Gm37899	-1.1303238	8.51E-07	9.54E-05
WT>TPPdeltaARE	Ush2a	-1.1309523	1.66E-05	0.00074902
WT>TPPdeltaARE	Gm37176	-1.1331919	3.39E-05	0.00125524
WT>TPPdeltaARE	Rnd1	-1.1347712	2.75E-06	0.00021452
WT>TPPdeltaARE	Gm44680	-1.1352448	7.95E-06	0.00043777
WT>TPPdeltaARE	Gpr4	-1.1359252	1.16E-08	3.49E-06
WT>TPPdeltaARE	Fmo1	-1.1365988	0.00024832	0.00525284
WT>TPPdeltaARE	Reep1	-1.1373767	0.00023263	0.00500345
WT>TPPdeltaARE	Npnt	-1.1384913	2.10E-05	0.00088714
WT>TPPdeltaARE	Lpin3	-1.1387149	4.94E-06	0.00032256
WT>TPPdeltaARE	A2ml1	-1.1401651	2.28E-05	0.00094553
WT>TPPdeltaARE	Nrxn3	-1.1431467	6.74E-07	8.27E-05
WT>TPPdeltaARE	Csrnp3	-1.1441417	2.38E-05	0.00097177
WT>TPPdeltaARE	Olfr157	-1.1444034	9.53E-06	0.00049451
WT>TPPdeltaARE	Itgb6	-1.1445269	2.47E-06	0.00020017
WT>TPPdeltaARE	Palmd	-1.1445486	0.00012825	0.00323459
WT>TPPdeltaARE	Nr6a1	-1.1451803	8.42E-08	1.62E-05
WT>TPPdeltaARE	Trank1	-1.1454475	0.00173194	0.02132667
WT>TPPdeltaARE	Hmcn1	-1.1461343	2.32E-08	6.21E-06
WT>TPPdeltaARE	Cped1	-1.1464592	2.73E-05	0.00108356
WT>TPPdeltaARE	Prom1	-1.1488064	3.88E-05	0.0013743
WT>TPPdeltaARE	Osbpl6	-1.1497362	9.26E-05	0.00257494
WT>TPPdeltaARE	Myo1b	-1.1499187	3.18E-07	4.75E-05
WT>TPPdeltaARE	Vmn1r209	-1.1529102	0.00016867	0.00394244
WT>TPPdeltaARE	Robo2	-1.1548869	0.00018015	0.00414775
WT>TPPdeltaARE	Col25a1	-1.1588811	3.37E-06	0.00024943
WT>TPPdeltaARE	Sgpp2	-1.1606135	1.42E-05	0.00066971
WT>TPPdeltaARE	A930038B10	-1.1634914	2.70E-05	0.00107436
WT>TPPdeltaARE	Pcdh15	-1.1639627	1.75E-07	3.02E-05
WT>TPPdeltaARE	Gm37142	-1.1648786	0.00077574	0.01199097
WT>TPPdeltaARE	Tspan12	-1.1653598	6.35E-08	1.36E-05
WT>TPPdeltaARE	Cgnl1	-1.1671373	0.00316427	0.03273142
WT>TPPdeltaARE	Tfpi	-1.1680442	0.00022065	0.00479505
WT>TPPdeltaARE	Etl4	-1.1758193	2.43E-07	4.04E-05
WT>TPPdeltaARE	Nrp1	-1.1894531	0.00047821	0.0084084
WT>TPPdeltaARE	Vmn2r113	-1.1897303	0.00024578	0.00521624
WT>TPPdeltaARE	Tgfbi	-1.1916635	5.55E-11	6.29E-08
WT>TPPdeltaARE	C130073E24I	-1.1960865	0.00010278	0.00279369
WT>TPPdeltaARE	Pclo	-1.1972237	1.10E-08	3.37E-06
WT>TPPdeltaARE	Kcna2	-1.1976441	0.00164272	0.02046338
WT>TPPdeltaARE	Slc1a2	-1.1978992	3.86E-06	0.00027862
WT>TPPdeltaARE	Gm43112	-1.203086	0.00128703	0.01716422
WT>TPPdeltaARE	Fblim1	-1.2053626	6.80E-05	0.00206684
WT>TPPdeltaARE	Rapgefl1	-1.2073391	7.69E-07	8.96E-05

WT>TPPdeltaARE	Nav3	-1.2099448	7.76E-06	0.00042986
WT>TPPdeltaARE	Erc2	-1.2141186	1.41E-05	0.00066602
WT>TPPdeltaARE	Rab3c	-1.2181303	1.00E-06	0.00010733
WT>TPPdeltaARE	B130024G19	-1.2189834	3.54E-07	5.21E-05
WT>TPPdeltaARE	Arpp21	-1.2203086	1.47E-06	0.00014007
WT>TPPdeltaARE	Dpep2	-1.2213971	4.73E-07	6.55E-05
WT>TPPdeltaARE	Limch1	-1.2241678	3.42E-05	0.00126015
WT>TPPdeltaARE	Nrep	-1.2252686	3.27E-05	0.00122754
WT>TPPdeltaARE	Zfp13	-1.225855	3.66E-05	0.00132613
WT>TPPdeltaARE	Pkhd1	-1.2282304	2.48E-05	0.00100973
WT>TPPdeltaARE	Poln	-1.233799	1.44E-05	0.00067194
WT>TPPdeltaARE	Galnt14	-1.234614	0.00031307	0.00634409
WT>TPPdeltaARE	Tfpi2	-1.2427945	6.82E-06	0.00039512
WT>TPPdeltaARE	Ptger2	-1.2440461	0.00019122	0.00432507
WT>TPPdeltaARE	Svopl	-1.2484087	3.38E-05	0.00125349
WT>TPPdeltaARE	Celsr3	-1.252005	9.73E-06	0.00049892
WT>TPPdeltaARE	Kdr	-1.2528301	0.00033255	0.0065863
WT>TPPdeltaARE	Kank1	-1.2531717	0.00078863	0.0121399
WT>TPPdeltaARE	Unc5c	-1.2539902	2.60E-05	0.00104701
WT>TPPdeltaARE	Sod3	-1.2607395	1.95E-05	0.0008388
WT>TPPdeltaARE	Fam135b	-1.2609195	1.06E-05	0.00053087
WT>TPPdeltaARE	Mrc1	-1.2616868	0.00182623	0.02203715
WT>TPPdeltaARE	Zfp536	-1.2628042	1.87E-05	0.00081892
WT>TPPdeltaARE	Adgrb3	-1.2628146	7.43E-06	0.0004164
WT>TPPdeltaARE	Slc19a3	-1.2675288	3.01E-06	0.00022656
WT>TPPdeltaARE	Gm37137	-1.2694234	2.67E-06	0.00020983
WT>TPPdeltaARE	Stab1	-1.2697527	2.81E-05	0.0011068
WT>TPPdeltaARE	Grik4	-1.2714292	2.18E-06	0.00018185
WT>TPPdeltaARE	Stox2	-1.2722267	0.00011195	0.00294893
WT>TPPdeltaARE	Vstm4	-1.2736508	4.98E-05	0.00163526
WT>TPPdeltaARE	Pi15	-1.275758	4.74E-06	0.00031323
WT>TPPdeltaARE	Spon1	-1.2778557	0.00035612	0.00688992
WT>TPPdeltaARE	Mapt	-1.2778579	2.39E-05	0.00097514
WT>TPPdeltaARE	Sema6d	-1.2823677	2.20E-06	0.00018202
WT>TPPdeltaARE	Nr2f2	-1.2824976	4.57E-06	0.00031069
WT>TPPdeltaARE	Gm34354	-1.283924	6.84E-05	0.00207669
WT>TPPdeltaARE	Atp8b1	-1.2882798	0.0031481	0.03265106
WT>TPPdeltaARE	Rhbdl2	-1.2887473	4.26E-06	0.00029765
WT>TPPdeltaARE	Nrp2	-1.2892254	0.00021263	0.00465627
WT>TPPdeltaARE	Akr1c19	-1.2957228	9.57E-09	3.18E-06
WT>TPPdeltaARE	Il13ra1	-1.2962821	0.00025483	0.00536125
WT>TPPdeltaARE	Akap12	-1.2993701	0.00061817	0.01021673
WT>TPPdeltaARE	Ttc6	-1.3044773	1.15E-06	0.00011836
WT>TPPdeltaARE	Bace2	-1.3063947	9.58E-07	0.00010376
WT>TPPdeltaARE	Abca13	-1.30847	1.74E-06	0.00015723

WT>TPPdeltaARE	Crygn	-1.3094601	0.00045527	0.00810105
WT>TPPdeltaARE	Fndc7	-1.3130069	1.13E-06	0.0001178
WT>TPPdeltaARE	Rnf165	-1.3169293	2.99E-06	0.00022633
WT>TPPdeltaARE	Dnah7a	-1.317922	2.50E-06	0.000201
WT>TPPdeltaARE	AI115009	-1.3236527	7.92E-06	0.00043742
WT>TPPdeltaARE	Kif1a	-1.3261271	7.86E-07	9.01E-05
WT>TPPdeltaARE	Synpo	-1.3263671	2.57E-06	0.00020452
WT>TPPdeltaARE	Tmem117	-1.3310117	5.97E-08	1.32E-05
WT>TPPdeltaARE	Efna5	-1.3333903	6.42E-07	8.14E-05
WT>TPPdeltaARE	Adgrv1	-1.3362696	0.00016089	0.00380892
WT>TPPdeltaARE	Fcgr2b	-1.3377824	1.95E-05	0.0008388
WT>TPPdeltaARE	Igf2bp1	-1.3405042	0.00010869	0.00290055
WT>TPPdeltaARE	Gm1110	-1.3443457	6.26E-07	8.05E-05
WT>TPPdeltaARE	Ddc	-1.3465032	7.64E-07	8.96E-05
WT>TPPdeltaARE	Sele	-1.3492562	6.87E-07	8.38E-05
WT>TPPdeltaARE	Thbd	-1.3510202	1.14E-05	0.00056014
WT>TPPdeltaARE	Cdh2	-1.3527376	0.00029518	0.00607108
WT>TPPdeltaARE	Gm10715	-1.3530715	0.0013536	0.01778133
WT>TPPdeltaARE	Sult5a1	-1.3533628	7.25E-06	0.00040951
WT>TPPdeltaARE	Abca12	-1.3557087	5.62E-05	0.0017955
WT>TPPdeltaARE	Fgd6	-1.3564755	1.54E-05	0.00071104
WT>TPPdeltaARE	BC106179	-1.3616211	9.52E-07	0.0001036
WT>TPPdeltaARE	Gm45694	-1.3671386	1.70E-06	0.00015659
WT>TPPdeltaARE	Gm21738	-1.3721173	1.44E-05	0.00067194
WT>TPPdeltaARE	Frmd5	-1.3726983	1.07E-06	0.00011253
WT>TPPdeltaARE	Prokr2	-1.3768718	5.14E-05	0.00167226
WT>TPPdeltaARE	Gm38263	-1.3782318	4.12E-07	5.80E-05
WT>TPPdeltaARE	Gm10718	-1.3783794	3.98E-05	0.0013919
WT>TPPdeltaARE	Col26a1	-1.382091	1.46E-06	0.00014007
WT>TPPdeltaARE	Pard3	-1.3839558	0.0002774	0.00573571
WT>TPPdeltaARE	Hecw1	-1.3872473	6.96E-07	8.44E-05
WT>TPPdeltaARE	Kirrel	-1.3919548	2.16E-09	9.88E-07
WT>TPPdeltaARE	Rab30	-1.3926413	6.39E-06	0.00038011
WT>TPPdeltaARE	Gm10717	-1.3946657	7.39E-05	0.00220205
WT>TPPdeltaARE	Pcdh12	-1.3976016	9.38E-05	0.00259949
WT>TPPdeltaARE	Kcnj8	-1.3998978	0.00012769	0.00322514
WT>TPPdeltaARE	Heph	-1.4009954	0.0013668	0.01793032
WT>TPPdeltaARE	Lrp2	-1.4053394	3.57E-09	1.41E-06
WT>TPPdeltaARE	Gm15851	-1.4058626	0.00014242	0.00349099
WT>TPPdeltaARE	Gm47123	-1.4060991	5.61E-09	2.04E-06
WT>TPPdeltaARE	Aox4	-1.4077025	0.00012515	0.00318166
WT>TPPdeltaARE	Olfr1372-ps1	-1.4079842	6.17E-07	7.97E-05
WT>TPPdeltaARE	Adam12	-1.4089927	8.23E-05	0.00237077
WT>TPPdeltaARE	Nwd2	-1.4102378	5.00E-07	6.79E-05
WT>TPPdeltaARE	Sox2ot	-1.4148926	5.92E-07	7.82E-05

WT>TPPdeltaARE	Nfib	-1.4173127	2.10E-07	3.51E-05
WT>TPPdeltaARE	Col6a3	-1.4175002	7.57E-06	0.00042213
WT>TPPdeltaARE	Btnl9	-1.4198244	3.73E-05	0.00133955
WT>TPPdeltaARE	Mafb	-1.4203112	0.00030407	0.00621397
WT>TPPdeltaARE	Lgi1	-1.4222792	3.50E-05	0.00127836
WT>TPPdeltaARE	Elavl4	-1.4235076	3.67E-09	1.41E-06
WT>TPPdeltaARE	Fam189a2	-1.4266708	6.47E-08	1.37E-05
WT>TPPdeltaARE	Adamts1	-1.4269824	5.87E-05	0.00185947
WT>TPPdeltaARE	Fat4	-1.4293509	8.04E-08	1.56E-05
WT>TPPdeltaARE	Sox17	-1.430038	9.40E-06	0.00049416
WT>TPPdeltaARE	C1qtnf1	-1.432436	3.84E-05	0.00136373
WT>TPPdeltaARE	Parva	-1.433801	3.27E-05	0.00122754
WT>TPPdeltaARE	Adcy4	-1.4344067	3.08E-06	0.00023111
WT>TPPdeltaARE	Flt1	-1.4384705	1.39E-06	0.0001362
WT>TPPdeltaARE	Dlc1	-1.4392084	3.56E-09	1.41E-06
WT>TPPdeltaARE	Fbxl7	-1.440593	1.75E-06	0.00015723
WT>TPPdeltaARE	Gm45762	-1.440794	7.45E-08	1.47E-05
WT>TPPdeltaARE	Vldlr	-1.4427016	6.47E-06	0.00038121
WT>TPPdeltaARE	C530043K16I	-1.4465352	5.94E-07	7.82E-05
WT>TPPdeltaARE	Npr1	-1.4470626	0.00075517	0.01177424
WT>TPPdeltaARE	Gm10722	-1.4500609	3.32E-05	0.00123597
WT>TPPdeltaARE	Jph1	-1.4505942	1.15E-09	5.98E-07
WT>TPPdeltaARE	Pcdh17	-1.4533217	6.76E-08	1.40E-05
WT>TPPdeltaARE	F8	-1.4568826	7.20E-05	0.00216028
WT>TPPdeltaARE	S100a16	-1.4604133	0.0001284	0.00323459
WT>TPPdeltaARE	Gm10801	-1.4673539	6.94E-06	0.00039675
WT>TPPdeltaARE	Lifr	-1.4744303	8.29E-05	0.00237861
WT>TPPdeltaARE	Adgrg6	-1.4765066	2.88E-05	0.00112092
WT>TPPdeltaARE	Osmr	-1.4781653	2.07E-06	0.00017724
WT>TPPdeltaARE	Abcc9	-1.481263	0.00065962	0.01074515
WT>TPPdeltaARE	Gm11168	-1.48345	3.72E-05	0.00133955
WT>TPPdeltaARE	Gm26870	-1.4847295	1.18E-05	0.00057516
WT>TPPdeltaARE	Gm10800	-1.4887273	1.28E-05	0.0006098
WT>TPPdeltaARE	Gli3	-1.4905751	6.93E-09	2.47E-06
WT>TPPdeltaARE	Gpr182	-1.49538	6.19E-06	0.00037045
WT>TPPdeltaARE	Prelp	-1.4959543	0.00024745	0.00524015
WT>TPPdeltaARE	Dnah6	-1.4962084	2.18E-06	0.00018185
WT>TPPdeltaARE	Tspan15	-1.5009521	4.02E-07	5.70E-05
WT>TPPdeltaARE	Hecw2	-1.5030059	2.20E-09	9.88E-07
WT>TPPdeltaARE	Vmn1r13	-1.5036405	3.23E-07	4.78E-05
WT>TPPdeltaARE	Disp2	-1.5046155	9.44E-06	0.00049416
WT>TPPdeltaARE	Asxl3	-1.5076842	3.83E-08	9.28E-06
WT>TPPdeltaARE	Stap2	-1.5077926	1.04E-09	5.70E-07
WT>TPPdeltaARE	Fam167b	-1.5208465	1.62E-05	0.00073795
WT>TPPdeltaARE	Colec12	-1.5284769	1.02E-05	0.00051571

WT>TPPdeltaARE	Erbb2	-1.5335106	0.00052528	0.00907046
WT>TPPdeltaARE	Cdh5	-1.5342343	0.00021056	0.00462918
WT>TPPdeltaARE	Gpm6a	-1.553699	9.10E-05	0.00254787
WT>TPPdeltaARE	Dab2	-1.5554945	1.99E-05	0.00085031
WT>TPPdeltaARE	Gm34866	-1.5559103	3.81E-10	2.63E-07
WT>TPPdeltaARE	Clca3a1	-1.5564231	1.32E-19	2.55E-15
WT>TPPdeltaARE	Lrrc32	-1.5566812	4.49E-06	0.00030792
WT>TPPdeltaARE	Spaar	-1.5626224	1.20E-09	6.06E-07
WT>TPPdeltaARE	Papln	-1.5649054	2.64E-05	0.0010582
WT>TPPdeltaARE	Pparg	-1.5698582	1.45E-05	0.00067582
WT>TPPdeltaARE	Bcar1	-1.5771269	1.38E-06	0.0001362
WT>TPPdeltaARE	Gm11747	-1.5850973	4.22E-05	0.00143785
WT>TPPdeltaARE	Galnt15	-1.5880138	0.00031765	0.00637272
WT>TPPdeltaARE	Stab2	-1.5922433	8.28E-05	0.00237659
WT>TPPdeltaARE	Pxdn	-1.5946803	9.57E-06	0.00049451
WT>TPPdeltaARE	Il13ra2	-1.6064017	0.00010859	0.00290055
WT>TPPdeltaARE	Scara5	-1.6096232	3.63E-09	1.41E-06
WT>TPPdeltaARE	Cdkl1	-1.6127321	0.00012677	0.00321447
WT>TPPdeltaARE	Gm10719	-1.6215225	5.50E-06	0.00034608
WT>TPPdeltaARE	C5ar1	-1.6240804	0.00044644	0.00800309
WT>TPPdeltaARE	Eln	-1.6280008	1.48E-07	2.68E-05
WT>TPPdeltaARE	Syt15	-1.6299721	0.00068274	0.0109825
WT>TPPdeltaARE	B630019A10	-1.6319534	1.77E-05	0.00078741
WT>TPPdeltaARE	Ackr1	-1.6558715	8.90E-05	0.00251052
WT>TPPdeltaARE	Plvap	-1.6609386	2.09E-05	0.00088452
WT>TPPdeltaARE	Pald1	-1.67168	1.40E-06	0.00013666
WT>TPPdeltaARE	Ptprb	-1.6918223	3.17E-07	4.75E-05
WT>TPPdeltaARE	Sema6a	-1.6959889	5.97E-10	3.49E-07
WT>TPPdeltaARE	Amotl1	-1.7009556	2.26E-08	6.12E-06
WT>TPPdeltaARE	Scamp5	-1.712943	7.58E-06	0.00042213
WT>TPPdeltaARE	Cald1	-1.7225689	4.92E-09	1.82E-06
WT>TPPdeltaARE	Prickle2	-1.7255669	5.27E-06	0.00033747
WT>TPPdeltaARE	Cdh4	-1.7338032	2.73E-08	7.03E-06
WT>TPPdeltaARE	Dysf	-1.73855	3.44E-05	0.00126494
WT>TPPdeltaARE	Hrct1	-1.7438269	8.89E-07	9.84E-05
WT>TPPdeltaARE	Stc1	-1.748955	2.90E-09	1.27E-06
WT>TPPdeltaARE	Aplnr	-1.750232	1.47E-11	2.02E-08
WT>TPPdeltaARE	C5ar2	-1.7590547	3.79E-06	0.00027774
WT>TPPdeltaARE	Hspg2	-1.7758244	1.76E-06	0.0001575
WT>TPPdeltaARE	Nid1	-1.7887298	1.00E-05	0.00050854
WT>TPPdeltaARE	Galnt18	-1.7895456	1.06E-05	0.00053087
WT>TPPdeltaARE	Ushbp1	-1.7950448	5.01E-06	0.00032504
WT>TPPdeltaARE	Ubd	-1.7964573	1.39E-06	0.0001362
WT>TPPdeltaARE	Dclk1	-1.7988514	1.44E-08	4.09E-06
WT>TPPdeltaARE	Mcam	-1.8001146	1.15E-06	0.00011836

WT>TPPdeltaARE	Tspan7	-1.8071028	1.06E-06	0.00011203
WT>TPPdeltaARE	Lgmn	-1.8149063	1.01E-08	3.22E-06
WT>TPPdeltaARE	Mmrn2	-1.8241984	1.57E-06	0.00014658
WT>TPPdeltaARE	Enpp6	-1.8378811	1.30E-10	1.09E-07
WT>TPPdeltaARE	Gm46565	-1.8426839	3.15E-07	4.75E-05
WT>TPPdeltaARE	Lipg	-1.854098	4.37E-05	0.00147073
WT>TPPdeltaARE	Acvrl1	-1.8552241	3.21E-05	0.00121497
WT>TPPdeltaARE	Flt4	-1.8819537	1.46E-06	0.00014007
WT>TPPdeltaARE	Rgs4	-1.8999392	6.08E-06	0.00036791
WT>TPPdeltaARE	Nrcam	-1.9095251	2.79E-07	4.41E-05
WT>TPPdeltaARE	D030007L05I	-1.9329436	3.18E-09	1.33E-06
WT>TPPdeltaARE	Igfbp3	-1.9619115	4.35E-09	1.64E-06
WT>TPPdeltaARE	Sparc	-1.9792238	7.64E-07	8.96E-05
WT>TPPdeltaARE	Afap1l2	-1.9980974	4.33E-06	0.00030048
WT>TPPdeltaARE	Tmem132e	-2.0098262	2.02E-06	0.00017529
WT>TPPdeltaARE	Adamts4	-2.016905	3.89E-15	1.25E-11
WT>TPPdeltaARE	Igkj2	-2.0402209	0.00089394	0.01323132
WT>TPPdeltaARE	Adh6b	-2.0510825	1.02E-06	0.00010823
WT>TPPdeltaARE	Dchs1	-2.1174227	1.72E-09	8.49E-07
WT>TPPdeltaARE	Ccdc9b	-2.2381114	8.91E-09	3.07E-06
WT>TPPdeltaARE	Adamts5	-2.2829182	1.44E-10	1.15E-07
WT>TPPdeltaARE	Camp	-2.2893885	0.0043194	0.04118712
WT>TPPdeltaARE	Ighj1	-2.3084768	0.00064589	0.01056613
WT>TPPdeltaARE	Lrg1	-2.4273315	1.94E-10	1.49E-07
WT>TPPdeltaARE	Sgk2	-2.4316607	3.64E-07	5.28E-05
WT>TPPdeltaARE	Sema3a	-2.7155211	1.30E-16	8.33E-13
WT>TPPdeltaARE	Ltf	-2.7830634	0.00031532	0.0063495
WT>TPPdeltaARE	Ngp	-2.9484222	0.00122262	0.01647631
WT>TPPdeltaARE	H2-Bl	-3.004459	5.59E-12	8.29E-09
WT>TPPdeltaARE	Ighm	-3.8106429	0.00095315	0.01378421
WT>TPPdeltaARE	Igfbp2	-3.8219193	1.77E-12	3.10E-09
WT>TPPdeltaARE	Igkv3-10	-5.4790859	0.00011638	0.00301456
WT>TPPdeltaARE	Ighv1-18	-6.5973367	0.00098484	0.01410023
WT>TPPdeltaARE	Ighv7-1	-7.2241435	4.14E-05	0.00142519

Middle Age WT_ HSC vs Middle Age TTPdeltaARE_HSC

Expression_Direction	Gene	logFC	PValue	FDR
WT<TPPdeltaARE	Ighv1-71	3.77889206	0.00119389	0.03200516
WT<TPPdeltaARE	Ndnf	2.5407146	2.24E-28	1.22E-24
WT<TPPdeltaARE	Nrsn2	2.38571458	1.32E-16	1.14E-13
WT<TPPdeltaARE	Ms4a4b	2.04390994	5.97E-05	0.00312665
WT<TPPdeltaARE	Tmc1	2.0319616	1.21E-07	1.60E-05
WT<TPPdeltaARE	Gimap3	1.94599751	4.72E-24	1.28E-20
WT<TPPdeltaARE	Eya4	1.94275929	1.50E-09	3.94E-07
WT<TPPdeltaARE	Cxcr6	1.92901294	4.95E-06	0.00039981

WT<TPPdeltaARE	Insm1	1.88953412	1.47E-07	1.90E-05
WT<TPPdeltaARE	Ubash3a	1.80966487	4.84E-07	5.51E-05
WT<TPPdeltaARE	P2rx7	1.79833885	1.17E-17	1.12E-14
WT<TPPdeltaARE	Nckap5	1.75207685	3.98E-07	4.67E-05
WT<TPPdeltaARE	Ccl3	1.67837889	2.75E-12	1.32E-09
WT<TPPdeltaARE	Siglecg	1.6749287	4.14E-10	1.21E-07
WT<TPPdeltaARE	Gimap7	1.63145256	1.63E-19	2.04E-16
WT<TPPdeltaARE	Kcnk10	1.59438713	1.53E-10	5.41E-08
WT<TPPdeltaARE	Ak5	1.53063189	3.61E-10	1.07E-07
WT<TPPdeltaARE	Dipk2b	1.51932517	4.06E-05	0.00231178
WT<TPPdeltaARE	Slc27a2	1.51541755	2.48E-06	0.00023084
WT<TPPdeltaARE	Slc38a11	1.47629872	8.62E-06	0.00061627
WT<TPPdeltaARE	Abcd2	1.45439021	2.47E-08	4.24E-06
WT<TPPdeltaARE	Abi3bp	1.43827214	3.32E-13	1.85E-10
WT<TPPdeltaARE	Kif5c	1.3510038	9.79E-07	0.0001036
WT<TPPdeltaARE	Shisa9	1.3466493	2.02E-08	3.53E-06
WT<TPPdeltaARE	1110032F04l	1.34111377	4.08E-05	0.00231178
WT<TPPdeltaARE	Trpc1	1.33328607	2.09E-11	8.50E-09
WT<TPPdeltaARE	Dusp26	1.33048028	1.19E-06	0.00012225
WT<TPPdeltaARE	Rnf39	1.31932686	9.80E-09	1.96E-06
WT<TPPdeltaARE	Igf1	1.30146148	5.50E-06	0.00043187
WT<TPPdeltaARE	Ildr1	1.29347268	4.29E-08	6.67E-06
WT<TPPdeltaARE	Bcat1	1.28299647	6.14E-09	1.35E-06
WT<TPPdeltaARE	Aldh3a1	1.27892151	2.21E-09	5.47E-07
WT<TPPdeltaARE	Stxbp6	1.26368391	5.51E-06	0.00043187
WT<TPPdeltaARE	Ndrg4	1.25798145	4.04E-09	9.28E-07
WT<TPPdeltaARE	Cyp26b1	1.25340804	2.34E-06	0.00022145
WT<TPPdeltaARE	Gm42047	1.25324656	2.91E-08	4.80E-06
WT<TPPdeltaARE	Il2rb	1.24328891	0.00095265	0.02714543
WT<TPPdeltaARE	Gm43534	1.22673128	3.09E-06	0.00027703
WT<TPPdeltaARE	Icam5	1.21677587	9.70E-08	1.34E-05
WT<TPPdeltaARE	Mab21l2	1.20562496	6.30E-11	2.33E-08
WT<TPPdeltaARE	Itgb5	1.20511041	6.01E-06	0.00046458
WT<TPPdeltaARE	Kcnb2	1.1964424	0.00070094	0.02147485
WT<TPPdeltaARE	Luzp2	1.19448491	6.76E-06	0.00051228
WT<TPPdeltaARE	Pls1	1.18546904	6.29E-07	6.93E-05
WT<TPPdeltaARE	Gas6	1.17805779	5.15E-14	3.36E-11
WT<TPPdeltaARE	Gm4117	1.16494049	0.0001162	0.00530508
WT<TPPdeltaARE	Creb5	1.16334741	0.0013669	0.03505028
WT<TPPdeltaARE	Gpr150	1.15508178	4.43E-07	5.08E-05
WT<TPPdeltaARE	Hopx	1.13200746	2.96E-09	7.00E-07
WT<TPPdeltaARE	Card11	1.13079987	7.53E-09	1.57E-06
WT<TPPdeltaARE	Tgm5	1.12933906	7.67E-08	1.12E-05
WT<TPPdeltaARE	Myl10	1.12733424	1.27E-12	6.29E-10
WT<TPPdeltaARE	Mcc	1.11690642	2.95E-08	4.81E-06

WT<TPPdeltaARE	Sidt1	1.11664876	1.79E-09	4.49E-07
WT<TPPdeltaARE	Psg16	1.11152728	3.48E-06	0.00029831
WT<TPPdeltaARE	Zan	1.08596364	0.00190494	0.04435455
WT<TPPdeltaARE	Jakmip1	1.08152456	8.24E-06	0.00059934
WT<TPPdeltaARE	Plek	1.08051308	6.03E-08	9.10E-06
WT<TPPdeltaARE	Synpo2	1.07238177	0.00024759	0.00960572
WT<TPPdeltaARE	Ank2	1.07237733	7.47E-05	0.00374415
WT<TPPdeltaARE	Yap1	1.06978627	8.01E-06	0.00058571
WT<TPPdeltaARE	Fcer2a	1.06914022	5.23E-06	0.00041393
WT<TPPdeltaARE	Clec4g	1.06635504	1.55E-08	2.85E-06
WT<TPPdeltaARE	Car12	1.06544071	0.00012403	0.00558449
WT<TPPdeltaARE	Gm37509	1.06042936	2.76E-07	3.38E-05
WT<TPPdeltaARE	Ncam2	1.05754776	3.82E-06	0.00032029
WT<TPPdeltaARE	Schip1	1.05114039	9.35E-07	9.96E-05
WT<TPPdeltaARE	Sema4a	1.04595139	0.00042607	0.01462006
WT<TPPdeltaARE	Sbspon	1.04249758	4.17E-08	6.53E-06
WT<TPPdeltaARE	Hopxos	1.0418325	4.08E-05	0.00231178
WT<TPPdeltaARE	Tnfrsf25	1.03771885	4.16E-08	6.53E-06
WT<TPPdeltaARE	Slc17a8	1.03671636	8.06E-10	2.27E-07
WT<TPPdeltaARE	Fbp1	1.01685062	3.42E-10	1.06E-07
WT<TPPdeltaARE	Hoxc6	1.00807545	7.12E-05	0.00361665
WT<TPPdeltaARE	Zfp36	1.00672337	5.69E-11	2.16E-08
WT<TPPdeltaARE	Islr	1.00211308	1.53E-08	2.83E-06
WT<TPPdeltaARE	Cfap57	0.99138201	0.00079489	0.02381611
WT<TPPdeltaARE	Lamb3	0.98176743	3.19E-05	0.00187074
WT<TPPdeltaARE	Snx31	0.97197143	1.42E-08	2.65E-06
WT<TPPdeltaARE	Prom2	0.97083878	6.33E-12	2.71E-09
WT<TPPdeltaARE	Cd28	0.97039112	1.97E-06	0.00019006
WT<TPPdeltaARE	Tmem255b	0.96586793	7.95E-07	8.58E-05
WT<TPPdeltaARE	Chst2	0.9635125	2.81E-06	0.00025705
WT<TPPdeltaARE	Tubg2	0.9622439	8.29E-06	0.00059954
WT<TPPdeltaARE	Dock4	0.95714317	0.00029953	0.01114625
WT<TPPdeltaARE	Esrrg	0.95289646	0.00062512	0.0197458
WT<TPPdeltaARE	Gm14964	0.95258355	2.63E-09	6.39E-07
WT<TPPdeltaARE	Ptprg	0.95100477	9.81E-05	0.00463463
WT<TPPdeltaARE	Dok3	0.94488487	5.82E-05	0.00305789
WT<TPPdeltaARE	Gm15816	0.93902511	0.00047792	0.0159623
WT<TPPdeltaARE	Gem	0.93805643	2.95E-09	7.00E-07
WT<TPPdeltaARE	Xdh	0.93695032	7.91E-09	1.63E-06
WT<TPPdeltaARE	Cx3cl1	0.93638731	8.15E-08	1.16E-05
WT<TPPdeltaARE	Unc5cl	0.93381149	1.29E-06	0.00013086
WT<TPPdeltaARE	Rab19	0.93308121	6.58E-09	1.41E-06
WT<TPPdeltaARE	Ebi3	0.9180567	1.01E-05	0.00070325
WT<TPPdeltaARE	Otos	0.91374316	9.79E-08	1.34E-05
WT<TPPdeltaARE	Mmp15	0.91068409	0.00075049	0.02263615

WT<TPPdeltaARE	Adora2b	0.91057557	6.46E-05	0.00334758
WT<TPPdeltaARE	Bhlhe41	0.90468402	1.75E-05	0.00115336
WT<TPPdeltaARE	Tmem30b	0.90428026	0.00050155	0.01649464
WT<TPPdeltaARE	Rorb	0.90133905	0.00021281	0.00862813
WT<TPPdeltaARE	Tbx1	0.89672432	1.23E-05	0.00083163
WT<TPPdeltaARE	Spry4	0.89473637	1.21E-07	1.60E-05
WT<TPPdeltaARE	Ovgp1	0.893256	0.00013149	0.00583951
WT<TPPdeltaARE	Slc16a11	0.880129	0.00013039	0.00580869
WT<TPPdeltaARE	Celf4	0.8792585	1.10E-05	0.00076388
WT<TPPdeltaARE	Scube3	0.87795666	0.00062066	0.01964309
WT<TPPdeltaARE	Elovl4	0.87753064	0.00029787	0.01110977
WT<TPPdeltaARE	Ciart	0.8673591	0.00019976	0.00824254
WT<TPPdeltaARE	Gimap4	0.86604523	1.83E-08	3.30E-06
WT<TPPdeltaARE	Emp2	0.86595057	3.01E-07	3.67E-05
WT<TPPdeltaARE	Slamf8	0.8642384	0.00013378	0.0059252
WT<TPPdeltaARE	Proser2	0.85908559	0.00017931	0.00755184
WT<TPPdeltaARE	Rdh10	0.85641297	6.71E-08	9.94E-06
WT<TPPdeltaARE	Dzank1	0.85197486	0.00074782	0.02261347
WT<TPPdeltaARE	Net1	0.85184346	7.45E-08	1.09E-05
WT<TPPdeltaARE	Chrm3	0.84500254	0.00186503	0.04361281
WT<TPPdeltaARE	Ank1	0.84199782	4.68E-05	0.00263033
WT<TPPdeltaARE	Flrt3	0.84100365	0.00032997	0.01203186
WT<TPPdeltaARE	Gm28053	0.83764067	0.00010889	0.00501331
WT<TPPdeltaARE	Bcl6b	0.83601038	5.04E-05	0.00277359
WT<TPPdeltaARE	Nrap	0.83550176	5.75E-05	0.00304026
WT<TPPdeltaARE	Itgb7	0.83456642	2.01E-07	2.56E-05
WT<TPPdeltaARE	Gm42686	0.82842959	8.91E-05	0.0043304
WT<TPPdeltaARE	Fbxo2	0.82597901	0.00045682	0.01540933
WT<TPPdeltaARE	Cd200r4	0.82196688	4.59E-06	0.00037199
WT<TPPdeltaARE	Gm16712	0.82158517	7.30E-05	0.00369574
WT<TPPdeltaARE	Camk1d	0.81913777	4.78E-05	0.00266941
WT<TPPdeltaARE	Nr0b2	0.81497038	1.63E-06	0.00016033
WT<TPPdeltaARE	Nrxn2	0.81495851	7.40E-06	0.00055039
WT<TPPdeltaARE	Glt8d2	0.81051246	7.34E-05	0.00370382
WT<TPPdeltaARE	Rasef	0.8090759	0.00051761	0.01690695
WT<TPPdeltaARE	Hbb-bt	0.8089415	0.00058716	0.01876479
WT<TPPdeltaARE	Sox5	0.80749379	1.69E-06	0.00016457
WT<TPPdeltaARE	Gm49871	0.80274388	0.00125767	0.03333127
WT<TPPdeltaARE	Ttc34	0.80260731	0.0005015	0.01649464
WT<TPPdeltaARE	Il18r1	0.80014714	0.00020157	0.00829647
WT<TPPdeltaARE	Slc1a1	0.79146268	0.0010579	0.0293744
WT<TPPdeltaARE	Sh3gl3	0.7908446	5.77E-05	0.0030445
WT<TPPdeltaARE	Xlr4a	0.79026857	0.00054514	0.01773487
WT<TPPdeltaARE	Nsun7	0.78365663	0.00027897	0.01050109
WT<TPPdeltaARE	Dusp4	0.77298494	6.33E-08	9.47E-06

WT<TPPdeltaARE	Kcnj2	0.7641131	0.00170519	0.04111376
WT<TPPdeltaARE	Slc25a21	0.75940776	0.00170825	0.04112674
WT<TPPdeltaARE	Gm11802	0.75909169	4.14E-07	4.83E-05
WT<TPPdeltaARE	P2ry10	0.75765556	0.00202677	0.04665866
WT<TPPdeltaARE	Pvt1	0.7567806	0.00096253	0.02733145
WT<TPPdeltaARE	Cd86	0.75240622	0.00062917	0.01979686
WT<TPPdeltaARE	Il1r2	0.75160868	0.00201114	0.04636441
WT<TPPdeltaARE	Rps6kl1	0.74968104	0.00170316	0.04111376
WT<TPPdeltaARE	Pcbp4	0.74847666	6.20E-05	0.0032379
WT<TPPdeltaARE	Ehd2	0.74641423	2.00E-05	0.00129367
WT<TPPdeltaARE	Pcdhb22	0.74471928	0.00209701	0.04785238
WT<TPPdeltaARE	Gpr160	0.74408305	1.40E-06	0.0001406
WT<TPPdeltaARE	Kazald1	0.73672306	4.29E-07	4.96E-05
WT<TPPdeltaARE	Haao	0.72845378	0.00127985	0.03353749
WT<TPPdeltaARE	4930539E08I	0.72520718	0.00072359	0.02204454
WT<TPPdeltaARE	A530010L16I	0.72163024	0.0010238	0.02871736
WT<TPPdeltaARE	Pdgfc	0.717863	0.00010005	0.00468612
WT<TPPdeltaARE	Gm20528	0.71721344	2.93E-05	0.00176518
WT<TPPdeltaARE	Pir	0.71594904	0.0020901	0.04777918
WT<TPPdeltaARE	Bpifb5	0.71483189	0.00017606	0.00747304
WT<TPPdeltaARE	Plk2	0.7121169	0.00049419	0.01633838
WT<TPPdeltaARE	Ugt1a7c	0.70805577	3.42E-06	0.00029615
WT<TPPdeltaARE	Papss2	0.70762524	0.00209918	0.04785238
WT<TPPdeltaARE	Atp8b5	0.70714564	0.00010277	0.00479544
WT<TPPdeltaARE	Gm11511	0.70583128	0.00218899	0.04948462
WT<TPPdeltaARE	Gm35028	0.70180653	0.00039844	0.01393603
WT<TPPdeltaARE	Pglyrp1	0.69921902	3.03E-06	0.00027609
WT<TPPdeltaARE	AB124611	0.69818873	0.00128755	0.03361457
WT<TPPdeltaARE	Lsp1	0.69289168	8.44E-07	9.05E-05
WT<TPPdeltaARE	Tnfaip8l2	0.69278172	4.70E-05	0.00263052
WT<TPPdeltaARE	Fosl1	0.69003407	0.00045758	0.01540933
WT<TPPdeltaARE	2900011O08	0.68751089	6.28E-07	6.93E-05
WT<TPPdeltaARE	Ankrd29	0.6869339	0.00119378	0.03200516
WT<TPPdeltaARE	Slc6a13	0.68580617	2.73E-05	0.0016819
WT<TPPdeltaARE	Spo11	0.68529319	8.96E-06	0.00063802
WT<TPPdeltaARE	Fam171b	0.68449496	8.36E-06	0.00060054
WT<TPPdeltaARE	Mroh2a	0.68154363	0.00086804	0.02544646
WT<TPPdeltaARE	Hpgds	0.68150086	1.13E-05	0.00077085
WT<TPPdeltaARE	Ccl4	0.68146346	0.00045099	0.0152503
WT<TPPdeltaARE	Ablim1	0.67948483	3.27E-05	0.00189755
WT<TPPdeltaARE	Gab3	0.67336795	0.00026624	0.01013877
WT<TPPdeltaARE	Car11	0.66471027	0.00100429	0.02822224
WT<TPPdeltaARE	Mc5r	0.66443377	0.00108579	0.02999548
WT<TPPdeltaARE	Insig1	0.66347524	9.81E-05	0.00463463
WT<TPPdeltaARE	4833407H14	0.66260417	2.32E-05	0.00145262

WT<TPPdeltaARE	Gm37549	0.65995602	0.00064305	0.02011728
WT<TPPdeltaARE	Gbp9	0.65903866	1.11E-05	0.00076423
WT<TPPdeltaARE	Anxa6	0.65701615	7.11E-06	0.00053672
WT<TPPdeltaARE	Hk3	0.65484224	8.82E-08	1.24E-05
WT<TPPdeltaARE	Thrb	0.65390596	1.45E-05	0.00096667
WT<TPPdeltaARE	C130026I21R	0.64995965	0.00129104	0.03361457
WT<TPPdeltaARE	Gm15701	0.64806638	0.00087988	0.02565501
WT<TPPdeltaARE	Rab34	0.64573268	3.25E-05	0.00189173
WT<TPPdeltaARE	Sec16b	0.64360089	0.00023463	0.00928077
WT<TPPdeltaARE	9930111J21F	0.64299875	0.00018367	0.00769585
WT<TPPdeltaARE	Ckb	0.64002082	4.03E-05	0.00230713
WT<TPPdeltaARE	4833418N02	0.64001404	0.00033881	0.01221721
WT<TPPdeltaARE	Gfi1	0.63947215	7.36E-06	0.00055018
WT<TPPdeltaARE	Ghdc	0.63384449	0.00025029	0.00966686
WT<TPPdeltaARE	A530040E14	0.63345152	5.14E-05	0.00282226
WT<TPPdeltaARE	Zbtb7b	0.6321812	0.00020511	0.00841267
WT<TPPdeltaARE	Arhgap30	0.63130119	9.58E-05	0.00456357
WT<TPPdeltaARE	Uchl1	0.62793708	0.00024254	0.00950267
WT<TPPdeltaARE	Bgn	0.62759013	7.89E-06	0.00057911
WT<TPPdeltaARE	Adrb2	0.62651523	0.00022529	0.00901855
WT<TPPdeltaARE	Tox	0.62410044	3.68E-05	0.00211387
WT<TPPdeltaARE	Gjb3	0.62073076	0.00016082	0.00693458
WT<TPPdeltaARE	Rasa4	0.62021692	0.00023517	0.00928077
WT<TPPdeltaARE	Abcb1a	0.61698256	0.00019748	0.00819001
WT<TPPdeltaARE	Krt80	0.61683695	0.00066251	0.02053193
WT<TPPdeltaARE	Gpx3	0.61646211	0.00036623	0.01297663
WT<TPPdeltaARE	Trim58	0.61611476	0.00047331	0.01584079
WT<TPPdeltaARE	Clec14a	0.61493522	0.0003409	0.01226576
WT<TPPdeltaARE	Gm15411	0.61486564	0.00011149	0.0051187
WT<TPPdeltaARE	Ankmy1	0.61259823	0.00054699	0.01775968
WT<TPPdeltaARE	Clec9a	0.6078748	0.00014485	0.00631238
WT<TPPdeltaARE	Fam20c	0.60540754	0.00105619	0.0293744
WT<TPPdeltaARE	Bcam	0.60150553	5.81E-06	0.0004529
WT<TPPdeltaARE	Gm9877	0.5945446	0.00154671	0.03848833
WT<TPPdeltaARE	Triqk	0.59291176	1.60E-05	0.00105834
WT<TPPdeltaARE	Gadd45a	0.59179298	0.00083288	0.02463729
WT<TPPdeltaARE	Ntn4	0.58830267	0.00070658	0.02160713
WT<TPPdeltaARE	Sytl1	0.58757097	3.29E-06	0.00028798
WT<TPPdeltaARE	Trib3	0.58687791	0.00033716	0.01218489
WT<TPPdeltaARE	Gsn	0.58604899	9.05E-05	0.00436779
WT<TPPdeltaARE	Gm35551	0.58542331	0.00016833	0.00718236
WT<TPPdeltaARE	Maf	0.5852734	0.00019973	0.00824254
WT<TPPdeltaARE	Car2	0.58178613	1.56E-05	0.00103081
WT<TPPdeltaARE	Lmcd1	0.58050456	0.00164278	0.03996369
WT<TPPdeltaARE	Akap17b	0.58032968	0.00097885	0.02765045

WT<TPPdeltaARE	Mmp16	0.57377012	0.00066261	0.02053193
WT<TPPdeltaARE	Arhgap15	0.57059375	1.83E-05	0.00118557
WT<TPPdeltaARE	Arhgap15os	0.56807882	0.00115869	0.0315284
WT<TPPdeltaARE	Zc3h6	0.56667512	0.000667	0.02062882
WT<TPPdeltaARE	Ppp1r15a	0.56659199	0.00110514	0.03032448
WT<TPPdeltaARE	Ifitm7	0.56508892	0.00010306	0.00479544
WT<TPPdeltaARE	Smad6	0.56426585	0.0003548	0.01263831
WT<TPPdeltaARE	Vegfa	0.56059421	0.00018521	0.00774032
WT<TPPdeltaARE	Bdh2	0.55543971	0.00046075	0.01548389
WT<TPPdeltaARE	Npas2	0.55501778	0.000321	0.01181047
WT<TPPdeltaARE	Gucy1a1	0.55358702	0.00010795	0.00498727
WT<TPPdeltaARE	Osgin1	0.55113539	2.69E-05	0.00166585
WT<TPPdeltaARE	Cd276	0.550433	0.00130513	0.03387305
WT<TPPdeltaARE	Pqlc3	0.54790699	9.45E-05	0.00451891
WT<TPPdeltaARE	Alox12	0.54759153	0.00155093	0.03853452
WT<TPPdeltaARE	Cirbp	0.5475227	0.00035247	0.01259851
WT<TPPdeltaARE	Gm6634	0.54450014	0.0022204	0.04994133
WT<TPPdeltaARE	Tinagl1	0.54236703	0.00016761	0.0071757
WT<TPPdeltaARE	Rora	0.53833103	0.00024811	0.00960572
WT<TPPdeltaARE	Gm10499	0.53704343	0.00013044	0.00580869
WT<TPPdeltaARE	Padi4	0.53486016	2.05E-05	0.00130926
WT<TPPdeltaARE	Gm15441	0.53433036	0.00126529	0.03342461
WT<TPPdeltaARE	Txnip	0.53172384	0.00140235	0.0356581
WT<TPPdeltaARE	5330406M23	0.53051644	0.00179761	0.042586
WT<TPPdeltaARE	Aspa	0.52942363	0.00119261	0.03200516
WT<TPPdeltaARE	Tpm4	0.52629082	0.0002773	0.01046211
WT<TPPdeltaARE	Zbtb46	0.52517778	0.00043801	0.01496683
WT<TPPdeltaARE	Gm13881	0.52470607	0.0019155	0.04447405
WT<TPPdeltaARE	Cav2	0.52371388	2.79E-05	0.00169698
WT<TPPdeltaARE	Clec1a	0.52091632	0.00087086	0.02548322
WT<TPPdeltaARE	Clip3	0.52041611	0.00041408	0.01429907
WT<TPPdeltaARE	Cdkn1c	0.50950655	0.00011981	0.00543927
WT<TPPdeltaARE	Mirt1	0.50807934	0.00080793	0.02407402
WT<TPPdeltaARE	Gas2l3	0.50382611	0.0001239	0.00558449
WT<TPPdeltaARE	Cd24a	0.50369941	2.79E-05	0.00169698
WT<TPPdeltaARE	Rgcc	0.49956008	0.00176911	0.04211942
WT<TPPdeltaARE	Pygm	0.49922411	0.00092973	0.02658537
WT<TPPdeltaARE	Lzts1	0.49573501	0.0016238	0.03956097
WT<TPPdeltaARE	Pkn3	0.49534782	0.00103194	0.02882899
WT<TPPdeltaARE	Slc25a33	0.49482316	0.00117747	0.03187983
WT<TPPdeltaARE	Gpc1	0.49246756	0.00023262	0.00922505
WT<TPPdeltaARE	Pde3b	0.490162	0.0004412	0.01500944
WT<TPPdeltaARE	Tef	0.49013409	0.00071101	0.02170177
WT<TPPdeltaARE	Apobr	0.48598743	0.00092515	0.02654511
WT<TPPdeltaARE	P2ry14	0.48560103	0.000958	0.02725021

WT<TPPdeltaARE	H2-T-ps	0.48525974	0.00182021	0.04299644
WT<TPPdeltaARE	Etv5	0.47479322	0.00041198	0.01425643
WT<TPPdeltaARE	Syne1	0.47459224	0.00102543	0.02871736
WT<TPPdeltaARE	Cyth1	0.47305039	0.00073548	0.02232317
WT<TPPdeltaARE	Fam169b	0.47204183	0.00127372	0.03343061
WT<TPPdeltaARE	Diaph3	0.47131115	0.0015811	0.03886922
WT<TPPdeltaARE	St3gal6	0.46950254	0.00158859	0.03893607
WT<TPPdeltaARE	Ppcdc	0.45690665	0.0002465	0.00960572
WT<TPPdeltaARE	Gm44732	0.4567649	0.00118209	0.03189881
WT<TPPdeltaARE	Camk2d	0.45380964	0.00034334	0.01232625
WT<TPPdeltaARE	Tnip3	0.44585242	0.00114942	0.03137766
WT<TPPdeltaARE	Sh2d3c	0.43900626	0.00088601	0.02574157
WT<TPPdeltaARE	Pcsk4	0.43135649	0.00156608	0.03867509
WT<TPPdeltaARE	Il1rapl2	0.42853914	0.00091374	0.02631848
WT<TPPdeltaARE	Traip	0.42473045	0.00208603	0.047753
WT<TPPdeltaARE	Stk26	0.42360999	0.00183276	0.04315251
WT<TPPdeltaARE	Pik3r1	0.42318033	0.00136788	0.03505028
WT<TPPdeltaARE	Procr	0.42116227	0.00064039	0.02007263
WT<TPPdeltaARE	Fcho1	0.42034222	0.0006806	0.02096993
WT<TPPdeltaARE	Il21r	0.4201405	0.00177016	0.04211942
WT<TPPdeltaARE	Glipr2	0.41969113	0.00129779	0.03373637
WT<TPPdeltaARE	Trpv2	0.41727844	0.00065019	0.02030172
WT<TPPdeltaARE	Slamf1	0.4112211	0.00143237	0.03619563
WT<TPPdeltaARE	Gm2a	0.41082111	0.00039258	0.01376048
WT<TPPdeltaARE	Stap1	0.39926145	0.00060734	0.01929644
WT<TPPdeltaARE	Hoxa9	0.37488991	0.00158514	0.03891001
WT<TPPdeltaARE	Traf3ip3	0.35425548	0.00200185	0.04621553
WT>TPPdeltaARE	Heatr1	-0.3766191	0.00161413	0.03938422
WT>TPPdeltaARE	Jak3	-0.3893445	0.00153645	0.03835007
WT>TPPdeltaARE	Cbx5	-0.4056573	0.00157762	0.03884236
WT>TPPdeltaARE	Peg13	-0.4062502	0.00131816	0.03408848
WT>TPPdeltaARE	Eps8	-0.4416569	0.00160865	0.03936849
WT>TPPdeltaARE	Cad	-0.4478675	0.00090353	0.02615752
WT>TPPdeltaARE	Trim62	-0.4483008	0.00109131	0.03008257
WT>TPPdeltaARE	Foxk2	-0.4515475	0.00222145	0.04994133
WT>TPPdeltaARE	Pbx1	-0.4613583	0.001191	0.03200516
WT>TPPdeltaARE	Nckipsd	-0.4761151	0.00056556	0.01814567
WT>TPPdeltaARE	Il1r1	-0.479363	0.00109783	0.03017448
WT>TPPdeltaARE	Gm15657	-0.5096594	0.00028162	0.01055189
WT>TPPdeltaARE	Xbp1	-0.5110384	0.00032233	0.01183112
WT>TPPdeltaARE	Cxcr4	-0.5120929	0.00087831	0.02565501
WT>TPPdeltaARE	Rhoj	-0.5145321	0.00014451	0.00631238
WT>TPPdeltaARE	Fry	-0.5226979	0.00098425	0.02775488
WT>TPPdeltaARE	S1pr1	-0.524265	0.00021158	0.00862138
WT>TPPdeltaARE	Cdc6	-0.5313505	0.00164931	0.04000303

WT>TPPdeltaARE	Podxl	-0.5406137	9.67E-05	0.00459709
WT>TPPdeltaARE	Gm26885	-0.5461363	0.00025761	0.00987576
WT>TPPdeltaARE	1700025G04	-0.5478498	0.0006353	0.01995139
WT>TPPdeltaARE	Fgfr1	-0.5555782	7.91E-05	0.00389619
WT>TPPdeltaARE	Pdxp	-0.5577216	0.00096654	0.02735002
WT>TPPdeltaARE	Pim1	-0.563565	0.00040171	0.01402033
WT>TPPdeltaARE	Pcdhgc3	-0.5779858	0.00108174	0.02998519
WT>TPPdeltaARE	Pclo	-0.5792308	0.00092669	0.02654511
WT>TPPdeltaARE	Dhx40	-0.5817347	0.00082252	0.02441948
WT>TPPdeltaARE	Ak4	-0.5850053	6.49E-05	0.00334758
WT>TPPdeltaARE	Gm16587	-0.5902251	0.00210337	0.04788093
WT>TPPdeltaARE	Nsg1	-0.5931466	0.00014242	0.00624002
WT>TPPdeltaARE	Chek1	-0.5957387	0.00143201	0.03619563
WT>TPPdeltaARE	Ppp1r3d	-0.5957774	5.54E-05	0.00298165
WT>TPPdeltaARE	Klhl13	-0.5991213	0.00150812	0.03775854
WT>TPPdeltaARE	Adgrl1	-0.6017367	0.00171753	0.04122834
WT>TPPdeltaARE	Ajuba	-0.6100772	0.00051596	0.01688688
WT>TPPdeltaARE	Gm16548	-0.6126638	0.00049043	0.01627997
WT>TPPdeltaARE	Cldn5	-0.6175679	0.00012503	0.00561371
WT>TPPdeltaARE	Brca1	-0.6212511	0.00050395	0.01652695
WT>TPPdeltaARE	Rbm19	-0.6213358	8.49E-05	0.00414072
WT>TPPdeltaARE	Dynll1	-0.6261516	0.0014546	0.03647465
WT>TPPdeltaARE	Mcm10	-0.6294763	0.00109264	0.03008257
WT>TPPdeltaARE	Ecm2	-0.6300073	0.00091394	0.02631848
WT>TPPdeltaARE	3-Mar	-0.6343983	7.67E-05	0.00380925
WT>TPPdeltaARE	Muc13	-0.6353874	3.35E-05	0.00193597
WT>TPPdeltaARE	Selp	-0.6405145	0.00022433	0.00901855
WT>TPPdeltaARE	Gm19265	-0.6421183	0.00190764	0.04435455
WT>TPPdeltaARE	Tspan12	-0.6466574	0.00083062	0.02461503
WT>TPPdeltaARE	Prkca	-0.6525854	3.00E-05	0.00177967
WT>TPPdeltaARE	Zfp948	-0.6614111	0.00013886	0.00611692
WT>TPPdeltaARE	Nav1	-0.6614161	4.34E-05	0.00244698
WT>TPPdeltaARE	Mmp14	-0.6666806	9.45E-06	0.00066674
WT>TPPdeltaARE	Per1	-0.6697549	9.97E-05	0.00468612
WT>TPPdeltaARE	E2f1	-0.6734541	0.00136185	0.03501059
WT>TPPdeltaARE	Plbd1	-0.6738454	0.00010801	0.00498727
WT>TPPdeltaARE	Psma8	-0.6773466	2.09E-05	0.00132864
WT>TPPdeltaARE	Sdc3	-0.681543	2.25E-05	0.00141742
WT>TPPdeltaARE	Igsf10	-0.684523	2.97E-05	0.00177967
WT>TPPdeltaARE	E2f7	-0.6968328	0.00048226	0.01607446
WT>TPPdeltaARE	Gstm2	-0.6973893	8.31E-06	0.00059954
WT>TPPdeltaARE	Cd34	-0.7008977	6.17E-07	6.89E-05
WT>TPPdeltaARE	Actn1	-0.7014291	2.84E-05	0.0017157
WT>TPPdeltaARE	Cdk6	-0.7016848	0.00024129	0.00947643
WT>TPPdeltaARE	Serpinb1a	-0.7024136	1.09E-06	0.00011369

WT>TPPdeltaARE	Ifi203-ps	-0.7046038	0.00079924	0.02390254
WT>TPPdeltaARE	Plppr2	-0.7050416	0.00113144	0.03099392
WT>TPPdeltaARE	Igfbp7	-0.7306176	7.49E-05	0.00374415
WT>TPPdeltaARE	Slc12a4	-0.7379015	0.00040634	0.01415156
WT>TPPdeltaARE	Slfn9	-0.7409602	7.57E-06	0.00056058
WT>TPPdeltaARE	Gm16897	-0.7511859	1.29E-05	0.00086359
WT>TPPdeltaARE	Cebpd	-0.7558198	1.77E-05	0.00115987
WT>TPPdeltaARE	Grhl1	-0.7558749	0.0003846	0.01351
WT>TPPdeltaARE	Clec4e	-0.7580173	1.30E-05	0.00086599
WT>TPPdeltaARE	Pawr	-0.7681623	0.00017775	0.00751853
WT>TPPdeltaARE	Scara3	-0.76868	0.00172638	0.04131907
WT>TPPdeltaARE	Pde9a	-0.7701465	2.01E-05	0.00129763
WT>TPPdeltaARE	Cxadr	-0.7739009	0.00065547	0.02042734
WT>TPPdeltaARE	Csf3r	-0.7789142	2.48E-06	0.00023084
WT>TPPdeltaARE	Gpx8	-0.7854194	0.00030214	0.0112178
WT>TPPdeltaARE	Gpc3	-0.786977	0.00144487	0.03639864
WT>TPPdeltaARE	Ncam1	-0.7878295	0.00024745	0.00960572
WT>TPPdeltaARE	Gm16175	-0.8009067	0.00056506	0.01814567
WT>TPPdeltaARE	Spint1	-0.8010544	0.00203817	0.04685491
WT>TPPdeltaARE	Gm37274	-0.8070302	0.00044053	0.01500944
WT>TPPdeltaARE	Rps4l	-0.8087784	3.09E-06	0.00027703
WT>TPPdeltaARE	Shroom4	-0.8095048	7.79E-06	0.00057421
WT>TPPdeltaARE	Slc16a4	-0.814832	0.00031573	0.01165789
WT>TPPdeltaARE	Steap3	-0.8350282	0.00115123	0.03137766
WT>TPPdeltaARE	B4galt2	-0.8383395	0.0018645	0.04361281
WT>TPPdeltaARE	Rin1	-0.8493851	0.00044294	0.01500944
WT>TPPdeltaARE	Rasgef1b	-0.8511978	2.57E-08	4.37E-06
WT>TPPdeltaARE	Gm48375	-0.8592305	0.00028365	0.01060385
WT>TPPdeltaARE	BC037039	-0.8604001	3.11E-05	0.00183648
WT>TPPdeltaARE	Ldb2	-0.8677786	0.000191	0.00794153
WT>TPPdeltaARE	Fermt2	-0.8744862	0.00178304	0.04236407
WT>TPPdeltaARE	Cnr2	-0.8767879	0.00055857	0.01802801
WT>TPPdeltaARE	Gm37035	-0.8796461	8.93E-05	0.0043304
WT>TPPdeltaARE	Gm26716	-0.8801714	0.00026838	0.01019672
WT>TPPdeltaARE	Ggt5	-0.8818907	0.00011443	0.00523903
WT>TPPdeltaARE	Bcl3	-0.8931617	4.87E-05	0.00270859
WT>TPPdeltaARE	Gm37194	-0.8973613	0.00016588	0.00713379
WT>TPPdeltaARE	P2ry1	-0.897829	7.62E-07	8.28E-05
WT>TPPdeltaARE	Nradd	-0.8994183	7.17E-06	0.00053834
WT>TPPdeltaARE	Insl3	-0.9011634	0.0001825	0.00766641
WT>TPPdeltaARE	Epas1	-0.9011816	5.58E-05	0.00299169
WT>TPPdeltaARE	Myo1e	-0.9110808	9.88E-07	0.00010393
WT>TPPdeltaARE	Serpinh1	-0.9110841	2.71E-08	4.56E-06
WT>TPPdeltaARE	Rps6ka3	-0.9113099	3.22E-08	5.15E-06
WT>TPPdeltaARE	Gm10644	-0.9129601	0.00022311	0.00900111

WT>TPPdeltaARE	Nxpe2	-0.9154699	1.80E-05	0.00117557
WT>TPPdeltaARE	Tsix	-0.9197409	9.98E-05	0.00468612
WT>TPPdeltaARE	Gipc2	-0.9252962	0.00011744	0.00534676
WT>TPPdeltaARE	A730091E23	-0.9289061	5.87E-06	0.0004554
WT>TPPdeltaARE	Gm37524	-0.9369879	0.00022485	0.00901855
WT>TPPdeltaARE	Nrtn	-0.9418843	0.0007429	0.0225065
WT>TPPdeltaARE	Slc22a3	-0.9431194	1.34E-07	1.76E-05
WT>TPPdeltaARE	Fcrla	-0.9500555	0.00103296	0.02882899
WT>TPPdeltaARE	Flt4	-0.9747606	7.96E-05	0.00390694
WT>TPPdeltaARE	Pim2	-0.9814792	4.43E-06	0.00036318
WT>TPPdeltaARE	Plac8	-0.9943274	5.71E-05	0.00302997
WT>TPPdeltaARE	Itga2b	-0.9951582	3.57E-10	1.07E-07
WT>TPPdeltaARE	Igha	-1.0014902	0.00138346	0.03528784
WT>TPPdeltaARE	Gm35189	-1.0018896	5.00E-06	0.00040137
WT>TPPdeltaARE	Oas2	-1.0057432	0.00050195	0.01649464
WT>TPPdeltaARE	Ffar2	-1.0064394	5.07E-06	0.00040502
WT>TPPdeltaARE	Adcy4	-1.0131894	0.00024529	0.00958763
WT>TPPdeltaARE	Xist	-1.017582	1.24E-06	0.00012643
WT>TPPdeltaARE	Pak6	-1.0299033	0.00038016	0.01341185
WT>TPPdeltaARE	Gm13431	-1.0313303	0.00032302	0.01183112
WT>TPPdeltaARE	Tjp1	-1.0319665	2.16E-05	0.00136779
WT>TPPdeltaARE	Plxnb2	-1.0351776	3.07E-13	1.79E-10
WT>TPPdeltaARE	Gm5833	-1.0373878	6.35E-09	1.38E-06
WT>TPPdeltaARE	Fgr	-1.0539995	2.98E-08	4.81E-06
WT>TPPdeltaARE	Lgmn	-1.084784	2.57E-07	3.17E-05
WT>TPPdeltaARE	Rian	-1.0955763	5.67E-05	0.00302575
WT>TPPdeltaARE	Map3k6	-1.1095101	0.00014212	0.00624002
WT>TPPdeltaARE	Gm11149	-1.1104266	3.52E-06	0.0002987
WT>TPPdeltaARE	Lrrn1	-1.1263723	5.14E-06	0.000409
WT>TPPdeltaARE	Nrep	-1.1263848	2.81E-05	0.00170151
WT>TPPdeltaARE	Pparg	-1.1312284	5.68E-05	0.00302575
WT>TPPdeltaARE	Itgb3	-1.1367958	1.51E-14	1.07E-11
WT>TPPdeltaARE	Prkd1	-1.1424471	6.40E-06	0.00048781
WT>TPPdeltaARE	Osmr	-1.1427045	4.47E-06	0.00036458
WT>TPPdeltaARE	Ripor3	-1.1474186	1.24E-05	0.00083502
WT>TPPdeltaARE	Ggt1	-1.1629385	6.24E-06	0.0004792
WT>TPPdeltaARE	Tgm1	-1.162955	1.38E-08	2.61E-06
WT>TPPdeltaARE	Igkc	-1.1690628	0.00211971	0.04818569
WT>TPPdeltaARE	Gli3	-1.1724367	0.00127368	0.03343061
WT>TPPdeltaARE	Tnfaip8l1	-1.2333158	0.00012272	0.00555615
WT>TPPdeltaARE	Depp1	-1.2372609	0.00090623	0.02618914
WT>TPPdeltaARE	Zg16	-1.250205	3.39E-06	0.00029567
WT>TPPdeltaARE	Iglv3	-1.2650115	0.00140554	0.03568366
WT>TPPdeltaARE	Spats2	-1.290759	2.35E-10	7.99E-08
WT>TPPdeltaARE	Pdgfrb	-1.2908042	2.07E-11	8.50E-09

WT>TPPdeltaARE	Col18a1	-1.3083275	2.48E-11	9.62E-09
WT>TPPdeltaARE	Gpr4	-1.3179599	7.68E-14	4.81E-11
WT>TPPdeltaARE	Rbpjl	-1.3205759	5.37E-12	2.43E-09
WT>TPPdeltaARE	Vcam1	-1.3279121	1.11E-06	0.00011472
WT>TPPdeltaARE	Mmrn2	-1.3319803	2.30E-06	0.00021966
WT>TPPdeltaARE	Stab2	-1.345092	9.06E-05	0.00436779
WT>TPPdeltaARE	Serping1	-1.3681316	3.09E-09	7.20E-07
WT>TPPdeltaARE	Abca4	-1.3725277	6.04E-12	2.66E-09
WT>TPPdeltaARE	Lox	-1.4065379	4.17E-06	0.00034539
WT>TPPdeltaARE	Fabp4	-1.4337611	3.46E-07	4.17E-05
WT>TPPdeltaARE	S100a8	-1.5013421	3.69E-06	0.0003115
WT>TPPdeltaARE	Clu	-1.5023266	1.55E-17	1.41E-14
WT>TPPdeltaARE	Slpi	-1.5191709	2.57E-05	0.00159713
WT>TPPdeltaARE	Ackr1	-1.5610075	6.26E-06	0.0004792
WT>TPPdeltaARE	S100a9	-1.5843906	1.17E-07	1.58E-05
WT>TPPdeltaARE	Sparc	-1.5844946	5.87E-09	1.31E-06
WT>TPPdeltaARE	Fzd1	-1.5870275	2.46E-08	4.24E-06
WT>TPPdeltaARE	Abcc9	-1.5872854	3.19E-06	0.0002814
WT>TPPdeltaARE	Gpm6a	-1.6220736	6.17E-10	1.76E-07
WT>TPPdeltaARE	Matn4	-1.6274574	4.41E-25	1.44E-21
WT>TPPdeltaARE	Ighj2	-1.7119167	0.00025812	0.00987576
WT>TPPdeltaARE	Socs3	-1.7259924	1.10E-08	2.13E-06
WT>TPPdeltaARE	Clca3a1	-1.7581403	8.54E-21	1.39E-17
WT>TPPdeltaARE	Fgf3	-1.7979897	2.76E-14	1.88E-11
WT>TPPdeltaARE	Gm16299	-1.8951776	1.30E-08	2.49E-06
WT>TPPdeltaARE	Gm34866	-1.9327071	5.68E-21	1.03E-17
WT>TPPdeltaARE	Plvap	-1.9415518	1.16E-07	1.58E-05
WT>TPPdeltaARE	Ushbp1	-1.9435105	1.70E-09	4.33E-07
WT>TPPdeltaARE	Tgfbi	-2.0388855	1.33E-13	8.02E-11
WT>TPPdeltaARE	Tspan7	-2.1554525	3.36E-10	1.06E-07
WT>TPPdeltaARE	Mrc1	-2.1715042	1.81E-10	6.26E-08
WT>TPPdeltaARE	Iglv1	-2.2101748	0.00033447	0.01216846
WT>TPPdeltaARE	Gm26906	-2.2203621	8.08E-15	5.99E-12
WT>TPPdeltaARE	Dlk1	-2.2219784	8.57E-22	2.00E-18
WT>TPPdeltaARE	Meg3	-2.227692	6.31E-20	9.35E-17
WT>TPPdeltaARE	Muc1	-2.2800693	9.56E-09	1.95E-06
WT>TPPdeltaARE	Igkj4	-2.4690731	0.00132684	0.03421873
WT>TPPdeltaARE	Vldlr	-2.6701872	2.11E-16	1.72E-13
WT>TPPdeltaARE	C4b	-2.6834082	5.18E-34	4.22E-30
WT>TPPdeltaARE	Lrg1	-2.8240854	6.87E-11	2.49E-08
WT>TPPdeltaARE	Igkv5-43	-2.892368	1.21E-06	0.00012367
WT>TPPdeltaARE	Tmem215	-2.9090775	1.17E-09	3.22E-07
WT>TPPdeltaARE	Rgs4	-3.56514	3.40E-13	1.85E-10
WT>TPPdeltaARE	Igkv1-117	-4.8645284	0.00161331	0.03938422
WT>TPPdeltaARE	Ighv1-55	-5.7594361	0.00020832	0.00850989

WT>TPPdeltaARE	Ighv1-34	-5.82545	0.00080246	0.02395482
WT>TPPdeltaARE	Ighv7-3	-6.1731209	0.00121766	0.03242919
WT>TPPdeltaARE	Igkv6-13	-6.8713943	9.89E-09	1.96E-06
WT>TPPdeltaARE	Igkv10-96	-7.5121406	3.12E-06	0.00027753
WT>TPPdeltaARE	Igkv6-17	-7.5231434	1.27E-09	3.44E-07
WT>TPPdeltaARE	Igkv19-93	-7.5545182	3.00E-05	0.00177967
WT>TPPdeltaARE	Ighv1-75	-7.6351054	6.43E-07	7.03E-05
WT>TPPdeltaARE	Ighv1-18	-7.6563689	2.17E-06	0.00020834
WT>TPPdeltaARE	Igkv4-59	-8.2728209	0.00164857	0.04000303
WT>TPPdeltaARE	Ighv2-2	-8.833843	7.06E-09	1.49E-06
WT>TPPdeltaARE	Ighg2c	-9.4781817	1.84E-08	3.30E-06
WT>TPPdeltaARE	Ighv5-16	-10.238275	1.06E-05	0.00073508
WT>TPPdeltaARE	Igkv9-124	-10.571607	3.34E-10	1.06E-07
WT>TPPdeltaARE	Igkv17-127	-10.963849	2.05E-26	8.34E-23
WT>TPPdeltaARE	Igkv17-121	-11.231941	1.44E-06	0.00014178
WT>TPPdeltaARE	Ighv1-22	-11.585662	6.52E-109	1.06E-104
WT>TPPdeltaARE	Ighv1-4	-11.879971	7.30E-19	8.50E-16
WT>TPPdeltaARE	Igkv5-48	-11.906767	2.42E-06	0.00022818
WT>TPPdeltaARE	Ighv1-58	-12.067973	1.42E-06	0.00014148
WT>TPPdeltaARE	Ighv14-2	-12.929963	2.43E-11	9.62E-09
WT>TPPdeltaARE	Igkv1-135	-14.666346	9.37E-08	1.31E-05
WT>TPPdeltaARE	Igkv15-103	-15.089772	2.53E-07	3.15E-05

Young WT_ MPP3 vs Young TTPdeltaARE_MPP3

Expression_Direction	Gene	logFC	PValue	FDR
WT<TPPdeltaARE	Ighv10-1	12.8758135	1.85E-89	2.91E-85
WT<TPPdeltaARE	Ighg3	7.66920957	6.76E-08	3.55E-06
WT<TPPdeltaARE	Slfn1	6.40299056	4.82E-57	2.53E-53
WT<TPPdeltaARE	Gpr33	5.95598847	1.09E-52	2.87E-49
WT<TPPdeltaARE	Iglc1	5.93524499	0.00154125	0.02991964
WT<TPPdeltaARE	Ifi205	5.50080942	3.91E-50	7.71E-47
WT<TPPdeltaARE	Cldn1	5.45268119	2.95E-24	6.55E-22
WT<TPPdeltaARE	Xcr1	5.42531192	8.27E-29	2.96E-26
WT<TPPdeltaARE	Iglj1	5.36752271	0.00115871	0.02375135
WT<TPPdeltaARE	Tlr13	5.27883352	3.70E-39	3.24E-36
WT<TPPdeltaARE	Mycl	5.22850575	3.62E-45	3.80E-42
WT<TPPdeltaARE	Cd209a	5.11740253	1.05E-17	1.33E-15
WT<TPPdeltaARE	Batf3	5.00531617	2.46E-34	1.44E-31
WT<TPPdeltaARE	Gpr141b	4.98323236	2.10E-27	6.35E-25
WT<TPPdeltaARE	Cxcl9	4.95958121	7.69E-62	6.06E-58
WT<TPPdeltaARE	Ccl8	4.88183914	1.61E-32	7.46E-30
WT<TPPdeltaARE	Zfp366	4.70930754	5.61E-54	1.77E-50
WT<TPPdeltaARE	Cd226	4.59524178	2.41E-42	2.24E-39
WT<TPPdeltaARE	Clec9a	4.58772838	2.10E-44	2.07E-41
WT<TPPdeltaARE	Gm36723	4.52615958	1.26E-23	2.62E-21

WT<TPPdeltaARE	Tlr11	4.4658517	1.17E-22	2.22E-20
WT<TPPdeltaARE	Olfm1	4.46571368	1.34E-33	6.60E-31
WT<TPPdeltaARE	Klrd1	4.45495025	1.70E-46	2.23E-43
WT<TPPdeltaARE	Plbd1	4.4537877	9.60E-22	1.68E-19
WT<TPPdeltaARE	Adam8	4.39860138	2.43E-38	2.02E-35
WT<TPPdeltaARE	Gramd2	4.3805796	5.93E-33	2.83E-30
WT<TPPdeltaARE	Gpr34	4.34406017	7.52E-30	3.12E-27
WT<TPPdeltaARE	Ifitm6	4.3074131	4.65E-29	1.75E-26
WT<TPPdeltaARE	Arhgap22	4.29473649	3.24E-30	1.38E-27
WT<TPPdeltaARE	Gpr141	4.27655572	3.19E-12	2.59E-10
WT<TPPdeltaARE	Prss34	4.22798625	8.99E-07	3.81E-05
WT<TPPdeltaARE	Plxdc1	4.21572594	1.14E-24	2.60E-22
WT<TPPdeltaARE	Ccr5	4.1971889	6.88E-18	8.89E-16
WT<TPPdeltaARE	Klrk1	4.1925997	2.21E-35	1.59E-32
WT<TPPdeltaARE	Igkv19-93	4.17558423	0.00212064	0.03909671
WT<TPPdeltaARE	Mefv	4.17468654	8.36E-08	4.32E-06
WT<TPPdeltaARE	Iglv1	4.16253079	0.00184838	0.03471094
WT<TPPdeltaARE	Scimp	4.13712157	2.10E-32	9.48E-30
WT<TPPdeltaARE	Ccr2	4.13018793	8.75E-51	1.97E-47
WT<TPPdeltaARE	Gm6377	4.06914574	1.61E-15	1.69E-13
WT<TPPdeltaARE	Lilra6	4.06407943	8.72E-21	1.40E-18
WT<TPPdeltaARE	Gpr35	4.06321224	2.93E-31	1.28E-28
WT<TPPdeltaARE	Slamf8	4.03328332	3.59E-38	2.83E-35
WT<TPPdeltaARE	Naaa	4.01682308	4.51E-37	3.38E-34
WT<TPPdeltaARE	Jaml	4.00337704	2.13E-18	2.89E-16
WT<TPPdeltaARE	Dnase1l3	3.98824488	6.89E-25	1.62E-22
WT<TPPdeltaARE	1-Mar	3.98627053	2.74E-28	9.40E-26
WT<TPPdeltaARE	Tlr3	3.97389772	1.09E-25	2.68E-23
WT<TPPdeltaARE	Adgrg5	3.9726857	1.19E-20	1.88E-18
WT<TPPdeltaARE	Prss30	3.942335	1.13E-25	2.73E-23
WT<TPPdeltaARE	Car1	3.92932936	1.96E-05	0.0006306
WT<TPPdeltaARE	7-Sep	3.92802453	5.09E-48	7.29E-45
WT<TPPdeltaARE	H2-Eb1	3.92637235	2.36E-54	9.32E-51
WT<TPPdeltaARE	Rab7b	3.92131368	4.93E-22	8.93E-20
WT<TPPdeltaARE	Kdr	3.92079226	2.35E-23	4.69E-21
WT<TPPdeltaARE	Phf11a	3.85297813	3.37E-24	7.38E-22
WT<TPPdeltaARE	P2ry6	3.84828269	4.62E-29	1.75E-26
WT<TPPdeltaARE	Klf2	3.83885115	1.32E-45	1.60E-42
WT<TPPdeltaARE	Ms4a4c	3.82091575	1.47E-26	4.16E-24
WT<TPPdeltaARE	Cxcr3	3.79540399	6.72E-29	2.46E-26
WT<TPPdeltaARE	Bcl2a1d	3.739132	1.23E-23	2.59E-21
WT<TPPdeltaARE	Serpinb10	3.68930375	2.76E-29	1.09E-26
WT<TPPdeltaARE	Mpeg1	3.66578404	2.05E-22	3.75E-20
WT<TPPdeltaARE	Gm15512	3.66138681	1.53E-21	2.62E-19
WT<TPPdeltaARE	Gm10684	3.63425325	3.53E-26	9.77E-24

WT<TPPdeltaARE	Mcemp1	3.61238191	2.37E-15	2.44E-13
WT<TPPdeltaARE	Pid1	3.5822274	2.12E-14	2.04E-12
WT<TPPdeltaARE	Id2	3.5686595	2.46E-35	1.69E-32
WT<TPPdeltaARE	Kmo	3.56656915	1.24E-33	6.28E-31
WT<TPPdeltaARE	Rtn1	3.56528631	7.19E-25	1.67E-22
WT<TPPdeltaARE	Atf3	3.53400727	3.83E-13	3.38E-11
WT<TPPdeltaARE	Itga8	3.50636678	2.15E-23	4.35E-21
WT<TPPdeltaARE	Rasgrp1	3.50165543	4.82E-07	2.18E-05
WT<TPPdeltaARE	Ptpro	3.49672963	1.91E-34	1.16E-31
WT<TPPdeltaARE	Ly86	3.49571811	7.11E-21	1.16E-18
WT<TPPdeltaARE	H2-Aa	3.45765793	5.06E-48	7.29E-45
WT<TPPdeltaARE	Ckb	3.45505288	3.88E-26	1.05E-23
WT<TPPdeltaARE	Sowahc	3.45196352	1.54E-19	2.25E-17
WT<TPPdeltaARE	Cd36	3.42728374	1.03E-15	1.13E-13
WT<TPPdeltaARE	Adam19	3.3972549	1.64E-11	1.27E-09
WT<TPPdeltaARE	Sema4a	3.39454029	5.44E-21	9.02E-19
WT<TPPdeltaARE	Itgax	3.38882112	1.24E-17	1.57E-15
WT<TPPdeltaARE	Il2rb	3.3760208	7.73E-15	7.62E-13
WT<TPPdeltaARE	Gm30489	3.36438132	9.36E-09	5.53E-07
WT<TPPdeltaARE	Cfh	3.34933895	5.89E-21	9.68E-19
WT<TPPdeltaARE	H2-Ab1	3.33298655	1.84E-49	3.23E-46
WT<TPPdeltaARE	Hbb-bs	3.30411685	4.51E-10	2.99E-08
WT<TPPdeltaARE	Ifi209	3.29491734	4.69E-17	5.77E-15
WT<TPPdeltaARE	Cysltr1	3.27407806	2.20E-13	2.00E-11
WT<TPPdeltaARE	Ifi211	3.25996812	3.64E-15	3.73E-13
WT<TPPdeltaARE	Zbtb46	3.2565766	3.29E-35	2.16E-32
WT<TPPdeltaARE	Cd40	3.23797631	6.97E-15	6.91E-13
WT<TPPdeltaARE	Irf8	3.2197426	3.55E-20	5.44E-18
WT<TPPdeltaARE	C1qtnf1	3.20334323	5.70E-05	0.00169154
WT<TPPdeltaARE	Cdh1	3.19136268	5.07E-05	0.00152104
WT<TPPdeltaARE	Col23a1	3.18783815	3.14E-16	3.64E-14
WT<TPPdeltaARE	Mcub	3.16930854	8.21E-24	1.75E-21
WT<TPPdeltaARE	Gprc5c	3.15087481	1.63E-13	1.52E-11
WT<TPPdeltaARE	Cd74	3.14896244	3.45E-19	4.90E-17
WT<TPPdeltaARE	F13a1	3.14352608	1.10E-05	0.00038416
WT<TPPdeltaARE	Gm14964	3.13210797	1.65E-09	1.06E-07
WT<TPPdeltaARE	Hba-a1	3.12997429	3.69E-08	2.03E-06
WT<TPPdeltaARE	1600010M07	3.09862279	7.91E-18	1.01E-15
WT<TPPdeltaARE	Hp	3.09851934	1.12E-08	6.56E-07
WT<TPPdeltaARE	Fam149a	3.09298508	1.46E-20	2.28E-18
WT<TPPdeltaARE	Pld4	3.06646426	1.52E-19	2.24E-17
WT<TPPdeltaARE	Fndc7	3.05303762	5.13E-22	9.18E-20
WT<TPPdeltaARE	Mcpt8	3.05188559	6.13E-08	3.24E-06
WT<TPPdeltaARE	Ly6i	3.01336012	5.47E-07	2.43E-05
WT<TPPdeltaARE	Ear2	3.01178483	6.34E-20	9.52E-18

WT<TPPdeltaARE	Qpct	2.99664635	5.21E-23	1.01E-20
WT<TPPdeltaARE	Asb2	2.98519055	7.75E-22	1.37E-19
WT<TPPdeltaARE	Ciita	2.9809301	4.41E-35	2.78E-32
WT<TPPdeltaARE	Fcmr	2.97315074	1.46E-05	0.00048792
WT<TPPdeltaARE	Tifab	2.95403837	5.77E-15	5.80E-13
WT<TPPdeltaARE	Fcgr4	2.94662766	1.82E-21	3.05E-19
WT<TPPdeltaARE	Aif1	2.90051773	1.37E-15	1.46E-13
WT<TPPdeltaARE	Rab39	2.88626503	5.11E-18	6.66E-16
WT<TPPdeltaARE	Hck	2.87944236	8.73E-34	4.74E-31
WT<TPPdeltaARE	Klf4	2.86770948	1.82E-15	1.89E-13
WT<TPPdeltaARE	Gpr183	2.82702165	1.12E-16	1.34E-14
WT<TPPdeltaARE	Iglc2	2.82557522	0.00034249	0.00843534
WT<TPPdeltaARE	Acvrl1	2.81485849	7.81E-27	2.28E-24
WT<TPPdeltaARE	Abca9	2.81343957	1.82E-05	0.00059111
WT<TPPdeltaARE	Strip2	2.79782737	9.96E-15	9.75E-13
WT<TPPdeltaARE	Treml4	2.78925663	9.68E-19	1.35E-16
WT<TPPdeltaARE	Ifi204	2.77150125	1.13E-21	1.97E-19
WT<TPPdeltaARE	Cd22	2.76278631	1.52E-22	2.82E-20
WT<TPPdeltaARE	Cd83	2.74435961	9.25E-08	4.71E-06
WT<TPPdeltaARE	Pmaip1	2.73737903	7.13E-11	5.11E-09
WT<TPPdeltaARE	Gm37787	2.71782768	0.00024178	0.00619707
WT<TPPdeltaARE	Ifi207	2.71023811	4.47E-19	6.29E-17
WT<TPPdeltaARE	Sema4f	2.70299673	1.28E-15	1.38E-13
WT<TPPdeltaARE	Nostrin	2.67848636	4.87E-12	3.90E-10
WT<TPPdeltaARE	Adgre1	2.67465383	1.06E-20	1.68E-18
WT<TPPdeltaARE	Xdh	2.6563742	7.94E-34	4.47E-31
WT<TPPdeltaARE	Tlr1	2.62706591	4.94E-18	6.51E-16
WT<TPPdeltaARE	Grap	2.60693394	6.41E-10	4.21E-08
WT<TPPdeltaARE	Gbp4	2.59072027	3.91E-09	2.39E-07
WT<TPPdeltaARE	Slfn2	2.58286975	8.31E-23	1.60E-20
WT<TPPdeltaARE	Cd300lg	2.57630664	1.61E-15	1.69E-13
WT<TPPdeltaARE	Cxcl16	2.57265834	2.73E-13	2.43E-11
WT<TPPdeltaARE	Slamf7	2.57026434	1.16E-05	0.00040442
WT<TPPdeltaARE	Dpep2	2.53488977	1.00E-12	8.58E-11
WT<TPPdeltaARE	Pirb	2.52942642	1.08E-29	4.37E-27
WT<TPPdeltaARE	Ms4a2	2.51152481	4.22E-10	2.80E-08
WT<TPPdeltaARE	Nrp1	2.50470311	8.46E-26	2.17E-23
WT<TPPdeltaARE	Ifi30	2.48529716	1.40E-23	2.86E-21
WT<TPPdeltaARE	Cd300c2	2.47120721	1.71E-16	2.03E-14
WT<TPPdeltaARE	Rnd3	2.45512518	8.88E-28	2.74E-25
WT<TPPdeltaARE	Ccl6	2.44741395	4.10E-18	5.47E-16
WT<TPPdeltaARE	Grap2	2.44125732	6.14E-16	6.82E-14
WT<TPPdeltaARE	Gdpd5	2.4358041	1.08E-15	1.18E-13
WT<TPPdeltaARE	H2-DMb2	2.43462689	8.52E-26	2.17E-23
WT<TPPdeltaARE	Ppfia4	2.42304293	9.95E-11	7.03E-09

WT<TPPdeltaARE	Ccr9	2.40642673	4.53E-07	2.06E-05
WT<TPPdeltaARE	Cxcl10	2.40230473	9.75E-08	4.94E-06
WT<TPPdeltaARE	L1cam	2.37797624	3.46E-07	1.61E-05
WT<TPPdeltaARE	Hdc	2.37690042	3.22E-12	2.60E-10
WT<TPPdeltaARE	Elane	2.36247092	4.14E-13	3.62E-11
WT<TPPdeltaARE	Gpr68	2.35973346	1.89E-19	2.71E-17
WT<TPPdeltaARE	Anpep	2.33388879	4.87E-20	7.39E-18
WT<TPPdeltaARE	Clec5a	2.33328187	1.72E-11	1.32E-09
WT<TPPdeltaARE	Notch4	2.33209312	1.46E-14	1.42E-12
WT<TPPdeltaARE	Lilrb4a	2.32431882	1.11E-08	6.52E-07
WT<TPPdeltaARE	Cd7	2.30979067	1.67E-17	2.08E-15
WT<TPPdeltaARE	Cst3	2.30659789	1.48E-26	4.16E-24
WT<TPPdeltaARE	Mpzl3	2.29442192	6.21E-13	5.41E-11
WT<TPPdeltaARE	Ahnak	2.29349344	2.03E-13	1.86E-11
WT<TPPdeltaARE	Slc45a3	2.27097192	4.39E-07	2.00E-05
WT<TPPdeltaARE	C3	2.25375558	7.18E-07	3.13E-05
WT<TPPdeltaARE	Igsf6	2.24461766	1.20E-12	1.02E-10
WT<TPPdeltaARE	Nrxn2	2.23391516	5.92E-11	4.36E-09
WT<TPPdeltaARE	Cd6	2.23306439	4.44E-06	0.0001672
WT<TPPdeltaARE	Btla	2.21590262	5.78E-28	1.82E-25
WT<TPPdeltaARE	Gm21188	2.21342543	5.04E-08	2.68E-06
WT<TPPdeltaARE	Cyp27a1	2.19699244	4.29E-24	9.27E-22
WT<TPPdeltaARE	Lrrc3	2.16649247	2.55E-13	2.30E-11
WT<TPPdeltaARE	Itgae	2.1638907	2.88E-16	3.37E-14
WT<TPPdeltaARE	Hepacam2	2.15418629	0.00075791	0.01666228
WT<TPPdeltaARE	Gm26740	2.14246758	9.46E-12	7.42E-10
WT<TPPdeltaARE	Clec4a3	2.13629135	1.81E-05	0.00058994
WT<TPPdeltaARE	Ctsh	2.10515118	1.82E-24	4.09E-22
WT<TPPdeltaARE	Nucb2	2.10452326	2.48E-13	2.25E-11
WT<TPPdeltaARE	P3h2	2.08916281	9.22E-16	1.02E-13
WT<TPPdeltaARE	Gimap3	2.07874642	1.01E-07	5.09E-06
WT<TPPdeltaARE	Rasgrp3	2.07469036	0.00019159	0.00506245
WT<TPPdeltaARE	Cyp4f18	2.0596099	9.71E-05	0.00273476
WT<TPPdeltaARE	Slc46a3	2.04759866	1.94E-18	2.65E-16
WT<TPPdeltaARE	Trbc2	2.04517137	0.00014227	0.00386642
WT<TPPdeltaARE	Slc2a6	2.04275519	8.65E-06	0.0003084
WT<TPPdeltaARE	Gm37759	2.03415906	0.0001175	0.00324379
WT<TPPdeltaARE	Pik3r6	2.02984841	4.89E-08	2.62E-06
WT<TPPdeltaARE	Cx3cr1	2.02154316	5.68E-09	3.43E-07
WT<TPPdeltaARE	Cadm1	2.00742343	2.01E-07	9.87E-06
WT<TPPdeltaARE	Cttnbp2nl	1.99189729	7.09E-10	4.64E-08
WT<TPPdeltaARE	Atp1a3	1.99097044	1.27E-05	0.00043654
WT<TPPdeltaARE	Adcy4	1.98012463	5.15E-10	3.39E-08
WT<TPPdeltaARE	Ifi213	1.97256759	1.13E-15	1.22E-13
WT<TPPdeltaARE	Serpina3f	1.96621448	1.36E-07	6.74E-06

WT<TPPdeltaARE	Gcsam	1.9609811	5.83E-16	6.61E-14
WT<TPPdeltaARE	Fcgr3	1.95822298	1.93E-13	1.79E-11
WT<TPPdeltaARE	Mx1	1.95447193	2.98E-10	2.02E-08
WT<TPPdeltaARE	Cd300lb	1.94823913	2.29E-07	1.12E-05
WT<TPPdeltaARE	Ly6a2	1.94500655	7.14E-05	0.00208098
WT<TPPdeltaARE	Gria2	1.93226938	5.67E-05	0.00168666
WT<TPPdeltaARE	Clec12a	1.92719472	9.76E-12	7.62E-10
WT<TPPdeltaARE	Hbb-bt	1.9166981	4.09E-10	2.73E-08
WT<TPPdeltaARE	Gbp9	1.91646137	3.47E-09	2.15E-07
WT<TPPdeltaARE	Cybb	1.91372469	1.82E-05	0.00058994
WT<TPPdeltaARE	Lgals3	1.90246729	4.64E-11	3.48E-09
WT<TPPdeltaARE	Gm43109	1.90176559	1.57E-06	6.33E-05
WT<TPPdeltaARE	Vcam1	1.89806544	1.01E-05	0.00035534
WT<TPPdeltaARE	Gm21887	1.8925577	7.40E-08	3.85E-06
WT<TPPdeltaARE	Dbn1	1.88652395	6.14E-16	6.82E-14
WT<TPPdeltaARE	Wfdc18	1.87636043	4.27E-11	3.22E-09
WT<TPPdeltaARE	Ctnnd2	1.87508383	6.15E-07	2.71E-05
WT<TPPdeltaARE	Gimap4	1.85968093	2.42E-06	9.44E-05
WT<TPPdeltaARE	Filip1l	1.85948781	2.99E-10	2.02E-08
WT<TPPdeltaARE	Dse	1.8569335	4.44E-06	0.0001672
WT<TPPdeltaARE	Tubb2a	1.83904588	5.44E-12	4.31E-10
WT<TPPdeltaARE	A530010L16I	1.83419605	1.98E-08	1.13E-06
WT<TPPdeltaARE	St3gal5	1.82887033	7.89E-17	9.50E-15
WT<TPPdeltaARE	Fcgr2b	1.82387555	6.70E-17	8.13E-15
WT<TPPdeltaARE	Tmem51	1.79061371	7.47E-14	7.05E-12
WT<TPPdeltaARE	Zfp36	1.77503805	2.01E-13	1.85E-11
WT<TPPdeltaARE	Adap2	1.77467507	6.41E-06	0.00023436
WT<TPPdeltaARE	Gas7	1.76005517	2.43E-07	1.17E-05
WT<TPPdeltaARE	Shtn1	1.75987468	2.53E-10	1.73E-08
WT<TPPdeltaARE	Vmn2r97	1.7574194	0.00010491	0.00293221
WT<TPPdeltaARE	Ms4a3	1.75254526	5.93E-05	0.00175696
WT<TPPdeltaARE	Ccr7	1.75124442	1.86E-08	1.06E-06
WT<TPPdeltaARE	Cacna1s	1.74371815	0.00042534	0.01015851
WT<TPPdeltaARE	Fcrl1	1.74344637	4.43E-08	2.40E-06
WT<TPPdeltaARE	Tppp3	1.73725003	6.24E-11	4.51E-09
WT<TPPdeltaARE	Itga1	1.72324784	1.30E-08	7.54E-07
WT<TPPdeltaARE	Kynu	1.72158484	4.80E-09	2.92E-07
WT<TPPdeltaARE	Gbp8	1.71929598	1.96E-05	0.00063121
WT<TPPdeltaARE	Sult1a1	1.71563965	3.32E-08	1.84E-06
WT<TPPdeltaARE	H2-DMb1	1.7107831	1.58E-19	2.29E-17
WT<TPPdeltaARE	Apba1	1.70987615	7.69E-11	5.48E-09
WT<TPPdeltaARE	Plk2	1.70344891	0.00088654	0.01885892
WT<TPPdeltaARE	Ccr3	1.69538041	0.00150131	0.02936125
WT<TPPdeltaARE	Itgb7	1.68187945	4.53E-16	5.21E-14
WT<TPPdeltaARE	Mertk	1.66289039	0.00059919	0.0134736

WT<TPPdeltaARE	Ctsg	1.654661	0.00199146	0.03688771
WT<TPPdeltaARE	B430306N03	1.65244708	2.63E-13	2.36E-11
WT<TPPdeltaARE	Fcgr1	1.64856171	4.81E-09	2.92E-07
WT<TPPdeltaARE	Ms4a6d	1.64317584	4.30E-08	2.34E-06
WT<TPPdeltaARE	Clec4a1	1.6394403	0.00039947	0.00967267
WT<TPPdeltaARE	Chd7	1.63221398	6.89E-11	4.96E-09
WT<TPPdeltaARE	Mafb	1.6304411	0.00031991	0.00796652
WT<TPPdeltaARE	Evl	1.6185851	1.09E-10	7.63E-09
WT<TPPdeltaARE	Gapt	1.61139761	8.42E-13	7.29E-11
WT<TPPdeltaARE	Degs2	1.60930236	8.05E-11	5.72E-09
WT<TPPdeltaARE	Aldh3b1	1.60868884	0.00180851	0.03418178
WT<TPPdeltaARE	Papss2	1.60644153	0.00088966	0.01887279
WT<TPPdeltaARE	Tmem156	1.60586804	3.58E-09	2.21E-07
WT<TPPdeltaARE	C1qa	1.59989503	0.00032875	0.00816082
WT<TPPdeltaARE	Traf1	1.59977909	1.10E-10	7.65E-09
WT<TPPdeltaARE	Gm36161	1.59514916	3.27E-08	1.82E-06
WT<TPPdeltaARE	Art3	1.59469491	2.79E-05	0.00086941
WT<TPPdeltaARE	E330020D12	1.59382047	0.00021233	0.00555047
WT<TPPdeltaARE	Gm34680	1.59242851	0.00012423	0.00341158
WT<TPPdeltaARE	Clec4a2	1.58926368	8.44E-08	4.33E-06
WT<TPPdeltaARE	Il1rl1	1.58917388	6.16E-05	0.00181872
WT<TPPdeltaARE	Gm5431	1.58801883	1.04E-06	4.34E-05
WT<TPPdeltaARE	Mrvi1	1.58757469	2.09E-07	1.02E-05
WT<TPPdeltaARE	Clec2i	1.58509755	1.52E-10	1.05E-08
WT<TPPdeltaARE	Zmynd15	1.58010329	3.38E-06	0.00012853
WT<TPPdeltaARE	Prdm1	1.57554684	0.00281801	0.04925294
WT<TPPdeltaARE	Lifr	1.5706293	3.11E-05	0.00096283
WT<TPPdeltaARE	Dab2	1.56893959	9.27E-08	4.71E-06
WT<TPPdeltaARE	Crispld2	1.56831287	3.23E-07	1.51E-05
WT<TPPdeltaARE	Slc11a1	1.54485979	7.45E-06	0.00026817
WT<TPPdeltaARE	C1qb	1.54017075	8.81E-05	0.00251661
WT<TPPdeltaARE	Pla2g7	1.5327317	6.39E-07	2.81E-05
WT<TPPdeltaARE	P2rx7	1.52335888	4.62E-05	0.00139713
WT<TPPdeltaARE	Trim7	1.51820143	7.30E-10	4.75E-08
WT<TPPdeltaARE	Cd52	1.51731423	1.09E-07	5.45E-06
WT<TPPdeltaARE	Ctss	1.51142977	8.89E-05	0.00252856
WT<TPPdeltaARE	Gbp6	1.50841879	3.77E-05	0.00115031
WT<TPPdeltaARE	Il1rapl2	1.50787136	0.00280462	0.04917604
WT<TPPdeltaARE	Tfcp2l1	1.50409812	6.85E-07	2.99E-05
WT<TPPdeltaARE	Gpr18	1.50207821	4.36E-12	3.51E-10
WT<TPPdeltaARE	Ildr1	1.50154153	0.00189262	0.03533321
WT<TPPdeltaARE	Ms4a7	1.49404649	2.80E-06	0.00010751
WT<TPPdeltaARE	Hspa1b	1.48945262	0.00040452	0.00973502
WT<TPPdeltaARE	Naip6	1.47648913	8.41E-08	4.33E-06
WT<TPPdeltaARE	Cd28	1.47438749	0.00263848	0.04644297

WT<TPPdeltaARE	Plce1	1.46962665	1.74E-05	0.00057102
WT<TPPdeltaARE	Depp1	1.46713353	9.21E-07	3.88E-05
WT<TPPdeltaARE	Fabp4	1.45923876	0.00024336	0.00621726
WT<TPPdeltaARE	Ryr1	1.43953163	4.07E-08	2.23E-06
WT<TPPdeltaARE	Tnfrsf11a	1.43922606	7.95E-07	3.41E-05
WT<TPPdeltaARE	Cnr2	1.43611199	9.01E-10	5.85E-08
WT<TPPdeltaARE	C1qc	1.4349521	0.00100235	0.02101077
WT<TPPdeltaARE	Tpm4	1.43188287	1.30E-12	1.10E-10
WT<TPPdeltaARE	Rnf43	1.43122062	2.52E-05	0.00079265
WT<TPPdeltaARE	Cd300lf	1.42765525	0.0016339	0.03148549
WT<TPPdeltaARE	Chga	1.42254966	5.01E-07	2.26E-05
WT<TPPdeltaARE	Kcnk6	1.42086189	1.91E-06	7.56E-05
WT<TPPdeltaARE	Ltb4r1	1.4187345	2.77E-09	1.72E-07
WT<TPPdeltaARE	Cd68	1.41670637	2.00E-14	1.94E-12
WT<TPPdeltaARE	Pik3r5	1.41586666	1.27E-09	8.24E-08
WT<TPPdeltaARE	Adgre4	1.41393899	0.0002756	0.00694657
WT<TPPdeltaARE	Gm26586	1.41049287	5.54E-11	4.10E-09
WT<TPPdeltaARE	Slc40a1	1.40692067	0.00070268	0.01553478
WT<TPPdeltaARE	6430548M08	1.40418208	2.16E-05	0.00069112
WT<TPPdeltaARE	Lpxn	1.39201055	1.32E-10	9.16E-09
WT<TPPdeltaARE	Fgr	1.38662532	7.88E-09	4.70E-07
WT<TPPdeltaARE	Ddx4	1.38457393	2.95E-08	1.66E-06
WT<TPPdeltaARE	Phf11b	1.382845	1.22E-08	7.10E-07
WT<TPPdeltaARE	Il13ra1	1.38222718	0.00216924	0.03966796
WT<TPPdeltaARE	Fgl2	1.38166343	5.06E-12	4.03E-10
WT<TPPdeltaARE	Mt1	1.37705261	0.00177531	0.03359447
WT<TPPdeltaARE	Klf1	1.37657407	0.00142122	0.02805826
WT<TPPdeltaARE	AC174780.1	1.37387411	0.00105756	0.0219057
WT<TPPdeltaARE	Camk1d	1.37244288	2.54E-12	2.07E-10
WT<TPPdeltaARE	Tnni2	1.36842795	1.81E-08	1.04E-06
WT<TPPdeltaARE	Gpr137b	1.36621054	5.09E-07	2.27E-05
WT<TPPdeltaARE	Nrg2	1.36583142	6.89E-06	0.00025027
WT<TPPdeltaARE	Cd86	1.36566226	6.38E-08	3.36E-06
WT<TPPdeltaARE	Adgra2	1.36097428	1.49E-06	6.02E-05
WT<TPPdeltaARE	Rtn4rl1	1.35687554	0.00083617	0.01794356
WT<TPPdeltaARE	Gm42372	1.35475844	1.57E-07	7.80E-06
WT<TPPdeltaARE	Adgre5	1.354261	8.19E-07	3.50E-05
WT<TPPdeltaARE	Pqlc2	1.35192851	3.53E-07	1.64E-05
WT<TPPdeltaARE	Gm26716	1.34866775	2.69E-07	1.28E-05
WT<TPPdeltaARE	Mtus1	1.34855507	3.15E-05	0.00097072
WT<TPPdeltaARE	Lpl	1.34278644	0.00171624	0.03283132
WT<TPPdeltaARE	Il4i1	1.33835596	6.12E-06	0.00022425
WT<TPPdeltaARE	Cacna1d	1.33704219	1.81E-05	0.00058994
WT<TPPdeltaARE	Havcr2	1.33206138	3.10E-10	2.09E-08
WT<TPPdeltaARE	Dock4	1.32769819	1.14E-06	4.71E-05

WT<TPPdeltaARE	Cd38	1.32385077	0.00016153	0.00435243
WT<TPPdeltaARE	Tspan33	1.32014937	1.93E-09	1.23E-07
WT<TPPdeltaARE	Slc31a2	1.31867747	3.86E-09	2.37E-07
WT<TPPdeltaARE	Skap1	1.31785684	1.67E-05	0.00055107
WT<TPPdeltaARE	Per1	1.31325596	7.90E-05	0.00228101
WT<TPPdeltaARE	Il17rb	1.30633865	2.68E-07	1.28E-05
WT<TPPdeltaARE	Evi2a	1.30632729	1.85E-09	1.18E-07
WT<TPPdeltaARE	S100a4	1.30075983	9.11E-07	3.85E-05
WT<TPPdeltaARE	Ppt1	1.29544301	1.76E-12	1.47E-10
WT<TPPdeltaARE	Sdc3	1.28983538	9.50E-07	3.98E-05
WT<TPPdeltaARE	Sptbn5	1.28918615	1.38E-05	0.00046496
WT<TPPdeltaARE	Slfn5	1.28911088	4.90E-07	2.21E-05
WT<TPPdeltaARE	Anxa3	1.2890632	0.00172044	0.03287189
WT<TPPdeltaARE	Pkib	1.28861844	8.75E-07	3.73E-05
WT<TPPdeltaARE	Slamf6	1.28786709	2.32E-05	0.00073295
WT<TPPdeltaARE	Wfdc17	1.28521669	3.19E-07	1.50E-05
WT<TPPdeltaARE	Zc3h12d	1.28452922	0.00023187	0.00600167
WT<TPPdeltaARE	Prr5l	1.28409901	2.94E-08	1.66E-06
WT<TPPdeltaARE	Ppm1m	1.28333953	2.13E-10	1.46E-08
WT<TPPdeltaARE	Mapk13	1.28319422	9.34E-06	0.0003301
WT<TPPdeltaARE	AB124611	1.28193776	7.20E-07	3.13E-05
WT<TPPdeltaARE	Gimap7	1.27378679	8.77E-09	5.22E-07
WT<TPPdeltaARE	Uchl1	1.2657789	2.42E-07	1.17E-05
WT<TPPdeltaARE	Gm42928	1.26138402	1.35E-06	5.48E-05
WT<TPPdeltaARE	Tmem255b	1.25893719	6.75E-05	0.00197801
WT<TPPdeltaARE	Fau-ps2	1.25176216	0.00168791	0.03240747
WT<TPPdeltaARE	Card11	1.24370518	1.62E-08	9.30E-07
WT<TPPdeltaARE	Il10ra	1.24367677	4.77E-08	2.58E-06
WT<TPPdeltaARE	Sgk1	1.24367523	1.96E-09	1.25E-07
WT<TPPdeltaARE	Tyrobp	1.23374877	2.59E-07	1.24E-05
WT<TPPdeltaARE	Ighj4	1.2323171	0.00089078	0.01887279
WT<TPPdeltaARE	Siglecg	1.23044117	1.27E-05	0.00043556
WT<TPPdeltaARE	Psap	1.22957278	6.32E-12	4.98E-10
WT<TPPdeltaARE	Stbd1	1.22924282	5.66E-05	0.00168666
WT<TPPdeltaARE	Bank1	1.22917837	0.00281734	0.04925294
WT<TPPdeltaARE	Ccl3	1.22561711	5.54E-06	0.00020487
WT<TPPdeltaARE	Trib1	1.22225534	0.00076766	0.01680631
WT<TPPdeltaARE	Zbp1	1.22144634	5.07E-07	2.27E-05
WT<TPPdeltaARE	Sulf2	1.22016348	1.83E-06	7.28E-05
WT<TPPdeltaARE	Rgs11	1.21550535	2.21E-06	8.64E-05
WT<TPPdeltaARE	Gm35551	1.20452777	6.45E-05	0.00189347
WT<TPPdeltaARE	Gm49500	1.20231197	1.59E-06	6.36E-05
WT<TPPdeltaARE	Tmsb4x	1.19712369	3.80E-11	2.88E-09
WT<TPPdeltaARE	Crybg1	1.19533395	1.18E-05	0.00041077
WT<TPPdeltaARE	Trpm2	1.1949656	4.10E-08	2.24E-06

WT<TPPdeltaARE	Il31ra	1.19443103	0.00023804	0.00612097
WT<TPPdeltaARE	Stxbp6	1.19414754	1.24E-05	0.00042926
WT<TPPdeltaARE	Pparg	1.19286082	2.32E-07	1.13E-05
WT<TPPdeltaARE	Ighv1-77	1.18744538	2.99E-05	0.00093259
WT<TPPdeltaARE	Adora2b	1.18443601	5.78E-06	0.00021294
WT<TPPdeltaARE	Lsr	1.18125865	3.08E-07	1.45E-05
WT<TPPdeltaARE	Rab11fip4	1.17441722	2.63E-06	0.00010179
WT<TPPdeltaARE	Rgs10	1.16894862	8.81E-07	3.74E-05
WT<TPPdeltaARE	Zbtb7b	1.16330376	0.00020314	0.00533685
WT<TPPdeltaARE	Lgals1	1.15869611	2.53E-07	1.22E-05
WT<TPPdeltaARE	Ust	1.15733538	1.33E-06	5.42E-05
WT<TPPdeltaARE	Fgd2	1.15190626	6.83E-09	4.10E-07
WT<TPPdeltaARE	Nod2	1.15069258	2.10E-05	0.00067241
WT<TPPdeltaARE	Plvap	1.15068928	2.49E-05	0.00078475
WT<TPPdeltaARE	Rag2	1.14854447	1.35E-05	0.00045995
WT<TPPdeltaARE	Cnn3	1.14832968	1.28E-06	5.24E-05
WT<TPPdeltaARE	Ms4a6c	1.14737676	4.21E-05	0.00127852
WT<TPPdeltaARE	Lmo1	1.14138898	5.12E-06	0.00019079
WT<TPPdeltaARE	Gas6	1.14073363	0.00230174	0.04165602
WT<TPPdeltaARE	Nlrp1b	1.14056248	8.12E-05	0.00233701
WT<TPPdeltaARE	Unc93b1	1.13884304	2.01E-08	1.14E-06
WT<TPPdeltaARE	Chdh	1.1356365	9.81E-06	0.00034606
WT<TPPdeltaARE	Phf11c	1.1316346	1.40E-06	5.66E-05
WT<TPPdeltaARE	Ttll3	1.12703327	0.00027462	0.00694657
WT<TPPdeltaARE	Trib2	1.1249166	0.00016066	0.00433648
WT<TPPdeltaARE	Cdkn1a	1.12294162	8.56E-06	0.00030593
WT<TPPdeltaARE	Ncf1	1.11570142	1.23E-05	0.00042489
WT<TPPdeltaARE	Slc8b1	1.11501994	3.00E-07	1.42E-05
WT<TPPdeltaARE	Slfn5os	1.11357963	4.59E-06	0.00017242
WT<TPPdeltaARE	Irf7	1.1126402	6.85E-08	3.59E-06
WT<TPPdeltaARE	Neurl1a	1.10784224	0.00041937	0.01003463
WT<TPPdeltaARE	P2ry10	1.10733193	6.49E-07	2.85E-05
WT<TPPdeltaARE	Ifit3	1.10644916	1.90E-05	0.00061441
WT<TPPdeltaARE	Kif5a	1.10235823	1.37E-05	0.00046496
WT<TPPdeltaARE	Tlr12	1.08925567	9.19E-08	4.70E-06
WT<TPPdeltaARE	Fcer1g	1.08097972	1.69E-07	8.37E-06
WT<TPPdeltaARE	6330537M06	1.07916267	3.14E-05	0.00097072
WT<TPPdeltaARE	Tbc1d9	1.0780167	1.67E-05	0.00055034
WT<TPPdeltaARE	Celf4	1.07510783	0.00027511	0.00694657
WT<TPPdeltaARE	Ifi27l2a	1.07331895	1.40E-05	0.00047098
WT<TPPdeltaARE	Bcl2l14	1.07131204	2.85E-06	0.00010873
WT<TPPdeltaARE	Bfsp2	1.06883038	3.27E-05	0.00100603
WT<TPPdeltaARE	Iigp1	1.06803823	3.33E-08	1.84E-06
WT<TPPdeltaARE	Stk36	1.0623915	0.00016337	0.00438707
WT<TPPdeltaARE	Sh3bp4	1.05872835	7.66E-07	3.31E-05

WT<TPPdeltaARE	Gm35853	1.05640863	7.18E-06	0.00026029
WT<TPPdeltaARE	Il1r2	1.05175738	0.00023153	0.00600167
WT<TPPdeltaARE	Phf11d	1.04703197	5.61E-06	0.00020701
WT<TPPdeltaARE	Irf5	1.0421061	1.81E-07	8.89E-06
WT<TPPdeltaARE	Itgb2	1.04035687	3.51E-08	1.93E-06
WT<TPPdeltaARE	Lbh	1.03463587	2.74E-06	0.00010582
WT<TPPdeltaARE	Lsp1	1.03061557	5.03E-08	2.68E-06
WT<TPPdeltaARE	Samhd1	1.03034156	5.09E-07	2.27E-05
WT<TPPdeltaARE	F630028O10	1.02696845	1.36E-05	0.00046063
WT<TPPdeltaARE	Tmem255a	1.02669627	0.00113698	0.02333618
WT<TPPdeltaARE	Ccl5	1.02471373	0.00221202	0.04026336
WT<TPPdeltaARE	I830077J02R	1.02445481	0.00034109	0.00841408
WT<TPPdeltaARE	Tulp3	1.02139972	0.00052102	0.01202458
WT<TPPdeltaARE	Csf1r	1.01699936	0.00011058	0.0030688
WT<TPPdeltaARE	Igf1	1.0159161	0.00244804	0.04385051
WT<TPPdeltaARE	BE692007	1.01131087	0.00023569	0.00608052
WT<TPPdeltaARE	Btbd16	1.00809879	0.00070693	0.01560693
WT<TPPdeltaARE	Icosl	1.00128292	5.43E-07	2.42E-05
WT<TPPdeltaARE	Maf	1.00077131	4.90E-05	0.00147313
WT<TPPdeltaARE	C130026I21R	1.00054756	9.89E-06	0.00034802
WT<TPPdeltaARE	Tubb6	0.99610926	2.12E-06	8.33E-05
WT<TPPdeltaARE	Card10	0.99325248	0.00040158	0.00969391
WT<TPPdeltaARE	Lmna	0.98903882	0.00010024	0.00281151
WT<TPPdeltaARE	Ap3s1	0.97622945	0.00011977	0.00330044
WT<TPPdeltaARE	Bmf	0.9753649	0.00094768	0.01997949
WT<TPPdeltaARE	Themis2	0.97315774	4.08E-07	1.88E-05
WT<TPPdeltaARE	Dtx4	0.96885179	0.00048762	0.0113378
WT<TPPdeltaARE	Plekha1	0.9675659	9.25E-07	3.89E-05
WT<TPPdeltaARE	Raph1	0.96419132	3.44E-05	0.00105465
WT<TPPdeltaARE	Rgs8	0.95816667	0.000691	0.01531966
WT<TPPdeltaARE	Aoah	0.94967059	0.00213531	0.03918373
WT<TPPdeltaARE	Ccdc102a	0.94806415	3.65E-06	0.00013864
WT<TPPdeltaARE	Hdac9	0.94685105	0.00088917	0.01887279
WT<TPPdeltaARE	Gsn	0.94106908	0.00033427	0.00828467
WT<TPPdeltaARE	Map7d2	0.94062067	0.00124463	0.0251205
WT<TPPdeltaARE	Anxa2	0.93753524	7.34E-07	3.18E-05
WT<TPPdeltaARE	Kdm7a	0.93565358	6.46E-06	0.00023584
WT<TPPdeltaARE	Tstd3	0.9337495	0.00174731	0.03330457
WT<TPPdeltaARE	5330406M23	0.92643852	0.00117751	0.02404278
WT<TPPdeltaARE	Lgmn	0.92109242	1.35E-05	0.00045995
WT<TPPdeltaARE	Pde3b	0.91892413	0.00027587	0.00694657
WT<TPPdeltaARE	H2-Oa	0.91875934	6.73E-07	2.95E-05
WT<TPPdeltaARE	Gm6277	0.91623133	0.00251593	0.04486273
WT<TPPdeltaARE	Gm43915	0.91604943	0.00263991	0.04644297
WT<TPPdeltaARE	Otulinl	0.91340333	1.59E-06	6.36E-05

WT<TPPdeltaARE	Tmem50b	0.91329536	7.42E-06	0.00026773
WT<TPPdeltaARE	Slc22a23	0.91051351	0.00081436	0.0176086
WT<TPPdeltaARE	Prkcb	0.90987851	0.00017399	0.0046249
WT<TPPdeltaARE	Gm14455	0.90844179	0.00101395	0.02119739
WT<TPPdeltaARE	Cfp	0.90782858	1.27E-05	0.00043654
WT<TPPdeltaARE	Slc27a2	0.90703837	0.00095829	0.02016756
WT<TPPdeltaARE	Cd33	0.89853965	0.00052471	0.01207456
WT<TPPdeltaARE	Gpc1	0.89811959	5.62E-05	0.00167863
WT<TPPdeltaARE	5031439G07	0.89362649	5.49E-06	0.00020376
WT<TPPdeltaARE	Tsc22d3	0.88938022	0.00141733	0.02805826
WT<TPPdeltaARE	Tmsb10	0.88896556	1.72E-06	6.84E-05
WT<TPPdeltaARE	Spint1	0.88492968	0.00036945	0.00905698
WT<TPPdeltaARE	Stox2	0.8815261	0.00155077	0.03006739
WT<TPPdeltaARE	9530052E02I	0.88111087	0.00110292	0.02272595
WT<TPPdeltaARE	Dok3	0.88106355	0.00121227	0.02462502
WT<TPPdeltaARE	Rtl8b	0.88060039	4.72E-05	0.00142422
WT<TPPdeltaARE	Rgmb	0.87780473	0.0005341	0.01223693
WT<TPPdeltaARE	Csf2ra	0.87483734	2.18E-05	0.00069479
WT<TPPdeltaARE	2310040G24	0.8728714	0.00100404	0.02101816
WT<TPPdeltaARE	Gramd3	0.87020094	9.55E-05	0.00270192
WT<TPPdeltaARE	Pik3cb	0.86857723	2.61E-05	0.00081916
WT<TPPdeltaARE	Gm10645	0.86371022	0.00175685	0.03336538
WT<TPPdeltaARE	Rubcnl	0.86083243	7.21E-05	0.0020917
WT<TPPdeltaARE	Lrp1	0.86080545	0.00149201	0.02921566
WT<TPPdeltaARE	Cass4	0.85450009	0.00143482	0.02827131
WT<TPPdeltaARE	Sh3tc1	0.85074775	0.00018616	0.00493177
WT<TPPdeltaARE	Gm27252	0.84803578	0.00189409	0.03533321
WT<TPPdeltaARE	Gm11767	0.83812574	0.00153209	0.02981515
WT<TPPdeltaARE	Btg2	0.83659884	0.00021337	0.00556845
WT<TPPdeltaARE	Gm4951	0.83491405	0.00015995	0.00432473
WT<TPPdeltaARE	Ankmy1	0.83411667	0.00038154	0.00932434
WT<TPPdeltaARE	Rnase6	0.83197527	4.74E-06	0.00017762
WT<TPPdeltaARE	Met	0.83031803	0.00112944	0.02321167
WT<TPPdeltaARE	Klrb1f	0.82789955	0.00107956	0.02230289
WT<TPPdeltaARE	Bcl6	0.82724633	0.00138216	0.02750882
WT<TPPdeltaARE	Ccnd1	0.82657377	0.00117023	0.02392528
WT<TPPdeltaARE	Gm26597	0.82269571	0.00101665	0.02122569
WT<TPPdeltaARE	Crip1	0.81541059	9.12E-06	0.00032313
WT<TPPdeltaARE	4930528J11F	0.81018415	0.00246337	0.04407493
WT<TPPdeltaARE	Myadm	0.80893237	4.50E-05	0.00136473
WT<TPPdeltaARE	Abcd2	0.80873197	0.00139011	0.02763215
WT<TPPdeltaARE	Ifngr1	0.80695986	1.82E-05	0.00058994
WT<TPPdeltaARE	Gm32401	0.80584147	0.0013081	0.026167
WT<TPPdeltaARE	BC147527	0.8007181	6.08E-05	0.00179853
WT<TPPdeltaARE	Fam129a	0.79666205	2.08E-05	0.00066844

WT<TPPdeltaARE	Psd	0.79617093	0.0022047	0.04017658
WT<TPPdeltaARE	Dhx40	0.7937265	0.00083523	0.01794356
WT<TPPdeltaARE	Rnf144b	0.79218404	0.00086391	0.01842726
WT<TPPdeltaARE	Ctsz	0.79111602	2.39E-05	0.00075381
WT<TPPdeltaARE	Gm8093	0.79109471	0.00251108	0.04482801
WT<TPPdeltaARE	BC051537	0.78341776	0.00013736	0.003746
WT<TPPdeltaARE	Naga	0.78327737	1.51E-05	0.00050184
WT<TPPdeltaARE	Klf6	0.78022238	0.00017276	0.00460781
WT<TPPdeltaARE	Aph1b	0.77787217	0.00054471	0.01240785
WT<TPPdeltaARE	Mospd2	0.77742044	0.00010061	0.00281699
WT<TPPdeltaARE	Clec7a	0.7676966	0.00048258	0.01125286
WT<TPPdeltaARE	Rtl8c	0.76439698	0.00189392	0.03533321
WT<TPPdeltaARE	Gm9844	0.76280293	0.00048149	0.01124404
WT<TPPdeltaARE	Gm2a	0.7602714	1.58E-05	0.00052463
WT<TPPdeltaARE	Lck	0.75716958	8.75E-05	0.00250287
WT<TPPdeltaARE	Slc9a9	0.7547065	0.00025286	0.00643917
WT<TPPdeltaARE	Hk2	0.75432177	0.00029395	0.00736651
WT<TPPdeltaARE	Tgfbi	0.74903641	0.00053784	0.01229598
WT<TPPdeltaARE	Scpep1os	0.74609529	0.00067385	0.01504509
WT<TPPdeltaARE	Anxa6	0.74510664	7.70E-05	0.0022258
WT<TPPdeltaARE	Rab43	0.7446853	0.00046703	0.01095505
WT<TPPdeltaARE	A530040E14	0.74303073	0.0012038	0.02448926
WT<TPPdeltaARE	Cpeb4	0.7419444	0.00041952	0.01003463
WT<TPPdeltaARE	Plekhm3	0.73855715	0.00074675	0.01644002
WT<TPPdeltaARE	Ece1	0.73852647	0.00019173	0.00506245
WT<TPPdeltaARE	Tifa	0.73783573	7.04E-05	0.00205628
WT<TPPdeltaARE	Fcgrt	0.7376508	0.0004097	0.0098297
WT<TPPdeltaARE	Dusp6	0.73687673	0.00081601	0.01762019
WT<TPPdeltaARE	Rhob	0.73676582	0.00046418	0.01092622
WT<TPPdeltaARE	Isg15	0.73658083	0.0008033	0.01744141
WT<TPPdeltaARE	Lrrk2	0.73511606	0.00147662	0.0289863
WT<TPPdeltaARE	Rasa4	0.73494379	0.00195829	0.03635871
WT<TPPdeltaARE	Btg1	0.7319674	0.00013389	0.00365761
WT<TPPdeltaARE	Cyth4	0.73082288	0.00056925	0.01286503
WT<TPPdeltaARE	Nfil3	0.72993847	0.00072632	0.01601252
WT<TPPdeltaARE	Gpr65	0.72957621	8.86E-05	0.00252541
WT<TPPdeltaARE	Slc35d3	0.72531764	0.00038618	0.0094231
WT<TPPdeltaARE	Il6ra	0.72411453	0.00052627	0.01207696
WT<TPPdeltaARE	Gm4673	0.72265289	0.00085592	0.01828156
WT<TPPdeltaARE	Gm16175	0.72039395	0.00215585	0.03946877
WT<TPPdeltaARE	S100a6	0.71486373	0.00076675	0.01680631
WT<TPPdeltaARE	Hpse	0.71276639	0.00213382	0.03918373
WT<TPPdeltaARE	Gpt2	0.71096874	0.00083781	0.01794356
WT<TPPdeltaARE	Ier5	0.70900627	0.002533	0.04511593
WT<TPPdeltaARE	Igtp	0.70889296	0.0005563	0.0126172

WT<TPPdeltaARE	Vsir	0.7071251	0.00107448	0.022227
WT<TPPdeltaARE	Cd2ap	0.70277986	0.00068173	0.01519957
WT<TPPdeltaARE	Pfkp	0.69361055	0.0002417	0.00619707
WT<TPPdeltaARE	H2-Ob	0.69198617	0.00042645	0.01016952
WT<TPPdeltaARE	D1Ertd622e	0.69135911	0.00201085	0.03720299
WT<TPPdeltaARE	Sorl1	0.68603542	0.00077588	0.01693937
WT<TPPdeltaARE	Slc12a9	0.68295738	0.00024231	0.00620046
WT<TPPdeltaARE	H2-DMa	0.68018336	0.0001996	0.00525725
WT<TPPdeltaARE	Rara	0.67766472	0.0005672	0.01284602
WT<TPPdeltaARE	Scpep1	0.67538232	0.00082224	0.01770629
WT<TPPdeltaARE	Gbp2	0.65950807	0.00032287	0.00802735
WT<TPPdeltaARE	Rassf4	0.65799628	0.00055303	0.01256104
WT<TPPdeltaARE	Ifngr2	0.65694144	0.00045948	0.01087514
WT<TPPdeltaARE	Syngr2	0.65460037	0.00061566	0.01382421
WT<TPPdeltaARE	Adarb1	0.65131539	0.00219872	0.04011395
WT<TPPdeltaARE	Plin2	0.64910247	0.00040116	0.00969391
WT<TPPdeltaARE	Gm47566	0.64444786	0.00081749	0.01762793
WT<TPPdeltaARE	Stat1	0.63698826	0.00056968	0.01286503
WT<TPPdeltaARE	Gm12250	0.63643451	0.00173961	0.03319796
WT<TPPdeltaARE	Tgtp1	0.6356096	0.00031872	0.0079492
WT<TPPdeltaARE	Gbp5	0.63489676	0.00260063	0.04595718
WT<TPPdeltaARE	Nckipsd	0.63400903	0.00188677	0.035322
WT<TPPdeltaARE	H2-K2	0.62785206	0.00238388	0.04299433
WT<TPPdeltaARE	Sla	0.623122	0.00101979	0.02126321
WT<TPPdeltaARE	Sat1	0.61747983	0.00104831	0.02174276
WT<TPPdeltaARE	Tmem202	0.61652757	0.00224444	0.0406657
WT<TPPdeltaARE	Stx7	0.6143521	0.0008375	0.01794356
WT<TPPdeltaARE	Taldo1	0.60242421	0.001286	0.02575749
WT<TPPdeltaARE	Slc25a38	0.60159095	0.00186291	0.03491688
WT<TPPdeltaARE	Nptn	0.6001919	0.00118101	0.02408308
WT<TPPdeltaARE	Bloc1s2	0.59976718	0.00120403	0.02448926
WT<TPPdeltaARE	Unc119b	0.59758117	0.00134105	0.02675817
WT<TPPdeltaARE	Ahcyl2	0.57820824	0.00141391	0.02803462
WT<TPPdeltaARE	Kbtbd11	0.57724771	0.00281838	0.04925294
WT<TPPdeltaARE	Calm1	0.57232453	0.00282631	0.04933674
WT<TPPdeltaARE	Rab32	0.54535625	0.00222665	0.04046857
WT>TPPdeltaARE	Tie1	-0.5746144	0.0023567	0.04255289
WT>TPPdeltaARE	Rab11fip5	-0.5925504	0.00184972	0.03471094
WT>TPPdeltaARE	Pard3b	-0.6160776	0.00182157	0.03438364
WT>TPPdeltaARE	Tceanc	-0.6162544	0.00240054	0.0431467
WT>TPPdeltaARE	Lamc1	-0.6226192	0.00268334	0.04715442
WT>TPPdeltaARE	Clvs1	-0.6467082	0.00239845	0.0431467
WT>TPPdeltaARE	Esam	-0.6470029	0.00203293	0.03752357
WT>TPPdeltaARE	Tbxa2r	-0.6476769	0.00094809	0.01997949
WT>TPPdeltaARE	Itm2a	-0.6526541	0.00148218	0.02905927

WT>TPPdeltaARE	Mfsd4b4	-0.6528079	0.00285394	0.04965419
WT>TPPdeltaARE	Dlg3	-0.6720478	0.00111542	0.02295342
WT>TPPdeltaARE	Gab1	-0.6749589	0.00198107	0.03673833
WT>TPPdeltaARE	Zfp947	-0.6893169	0.00241483	0.0433541
WT>TPPdeltaARE	Ppp1r3d	-0.6908015	0.00124901	0.02517667
WT>TPPdeltaARE	C1rl	-0.6921728	0.00162088	0.03134957
WT>TPPdeltaARE	D130058E05	-0.6928672	0.00230743	0.04171108
WT>TPPdeltaARE	Rab17	-0.7002826	0.00175597	0.03336538
WT>TPPdeltaARE	Alg6	-0.7121699	0.00066843	0.01496655
WT>TPPdeltaARE	Zfp108	-0.7186904	0.00284573	0.04959311
WT>TPPdeltaARE	Zgrf1	-0.7256455	0.00144902	0.02847997
WT>TPPdeltaARE	Kcnd1	-0.7322421	0.00284729	0.04959311
WT>TPPdeltaARE	Gm49760	-0.7348462	0.00255641	0.04537912
WT>TPPdeltaARE	Aif1l	-0.7386591	0.00142223	0.02805826
WT>TPPdeltaARE	Ralgps1	-0.7413698	0.0010468	0.02173999
WT>TPPdeltaARE	Gp9	-0.7553092	0.00182995	0.0344629
WT>TPPdeltaARE	E130311K13I	-0.7671372	0.00151629	0.02958079
WT>TPPdeltaARE	BC030343	-0.7700994	0.00254189	0.04522322
WT>TPPdeltaARE	Zfp93	-0.7720616	0.0004308	0.01025794
WT>TPPdeltaARE	Zc3h6	-0.7762506	0.00020857	0.00546139
WT>TPPdeltaARE	Gng11	-0.7815945	0.00022625	0.00588502
WT>TPPdeltaARE	Ptpdc1	-0.7837589	0.00077998	0.01700528
WT>TPPdeltaARE	S1pr3	-0.7891101	0.0021267	0.0391168
WT>TPPdeltaARE	Itga2b	-0.8002613	0.00040607	0.00975736
WT>TPPdeltaARE	Slfn9	-0.8034181	0.0002771	0.00696638
WT>TPPdeltaARE	Spef1	-0.8064771	0.00136988	0.0272988
WT>TPPdeltaARE	Fam171a2	-0.807184	0.00027382	0.00693918
WT>TPPdeltaARE	Gp5	-0.854193	0.00029347	0.0073661
WT>TPPdeltaARE	Fbln1	-0.8636295	0.00176816	0.03353971
WT>TPPdeltaARE	Prx	-0.8675486	6.26E-05	0.0018433
WT>TPPdeltaARE	Dock1	-0.8780898	4.73E-05	0.00142422
WT>TPPdeltaARE	Gm43858	-0.8827346	0.00150758	0.02944722
WT>TPPdeltaARE	Fbxo16	-0.8901166	0.00251114	0.04482801
WT>TPPdeltaARE	Il1r1	-0.9005055	6.37E-05	0.00187402
WT>TPPdeltaARE	Gm45667	-0.9011262	0.0017527	0.03336538
WT>TPPdeltaARE	Pcdh7	-0.9027408	0.00051408	0.0119167
WT>TPPdeltaARE	Rgs7bp	-0.9149024	0.00038826	0.00944554
WT>TPPdeltaARE	4930432K21I	-0.9221725	0.0003883	0.00944554
WT>TPPdeltaARE	Klhl4	-0.9269746	0.00014153	0.00385304
WT>TPPdeltaARE	Shroom4	-0.9270911	0.00010644	0.0029695
WT>TPPdeltaARE	Camk2b	-0.9308422	0.00017382	0.0046249
WT>TPPdeltaARE	Cyb561	-0.9313677	0.00015165	0.00410721
WT>TPPdeltaARE	1700123M08	-0.9335251	0.00123931	0.02505431
WT>TPPdeltaARE	Ajuba	-0.9341388	0.00254828	0.04528575
WT>TPPdeltaARE	Lrig3	-0.9372524	0.00051597	0.01194316

WT>TPPdeltaARE	Snx7	-0.9384209	1.63E-05	0.0005392
WT>TPPdeltaARE	Trp53cor1	-0.9421215	0.00035235	0.00865111
WT>TPPdeltaARE	Tcaf1	-0.9436243	1.46E-05	0.00048792
WT>TPPdeltaARE	Obsl1	-0.9523485	0.00017202	0.00459576
WT>TPPdeltaARE	Asb1	-0.9547295	0.00014514	0.00393784
WT>TPPdeltaARE	Rab27b	-0.9793523	2.17E-06	8.51E-05
WT>TPPdeltaARE	Ppic	-0.9918549	2.74E-07	1.30E-05
WT>TPPdeltaARE	Exoc3l2	-1.0021615	0.00068625	0.01525712
WT>TPPdeltaARE	Slc16a12	-1.0059831	0.00012176	0.00334947
WT>TPPdeltaARE	Sox6	-1.0071044	7.82E-07	3.37E-05
WT>TPPdeltaARE	Tmem74	-1.0222763	0.00037973	0.00929453
WT>TPPdeltaARE	Dmc1	-1.0273676	0.0005509	0.01253079
WT>TPPdeltaARE	Hist1h3d	-1.0421363	0.00183788	0.03457096
WT>TPPdeltaARE	Gm32742	-1.0540641	0.00020537	0.00538653
WT>TPPdeltaARE	5730414N17	-1.0693701	0.00141067	0.02800559
WT>TPPdeltaARE	Fzd8	-1.0827831	7.30E-05	0.0021142
WT>TPPdeltaARE	Mmp14	-1.0838672	0.00026963	0.00685513
WT>TPPdeltaARE	Gp1ba	-1.0883331	0.00044416	0.01055997
WT>TPPdeltaARE	Gfi1b	-1.0957108	3.53E-05	0.00107868
WT>TPPdeltaARE	Gm41556	-1.1120547	0.00077522	0.01693937
WT>TPPdeltaARE	Tgfbr3	-1.1217883	3.93E-07	1.81E-05
WT>TPPdeltaARE	Pdgfrb	-1.1324124	2.67E-05	0.00083366
WT>TPPdeltaARE	Mpdz	-1.135135	0.00123976	0.02505431
WT>TPPdeltaARE	Igsf10	-1.1366554	1.38E-05	0.00046553
WT>TPPdeltaARE	Ifi203-ps	-1.1428123	0.00076765	0.01680631
WT>TPPdeltaARE	4732491K20I	-1.1750452	0.00110208	0.02272595
WT>TPPdeltaARE	Pcdhgc4	-1.2037178	0.00047741	0.0111819
WT>TPPdeltaARE	Nhsl1	-1.2241824	0.00033796	0.00834988
WT>TPPdeltaARE	Phldb2	-1.2258541	1.18E-06	4.87E-05
WT>TPPdeltaARE	Vldlr	-1.2638367	7.16E-05	0.00208215
WT>TPPdeltaARE	Plscr2	-1.2776626	9.22E-05	0.00261894
WT>TPPdeltaARE	Tnfsf4	-1.2948881	3.90E-05	0.00118751
WT>TPPdeltaARE	Gm16548	-1.3164067	0.00127773	0.02562451
WT>TPPdeltaARE	Tek	-1.3447235	2.17E-10	1.48E-08
WT>TPPdeltaARE	Zkscan4	-1.3910627	2.07E-06	8.16E-05
WT>TPPdeltaARE	Stag3	-1.4261725	0.0012548	0.02522889
WT>TPPdeltaARE	4932441J04F	-1.5445751	4.91E-06	0.00018339
WT>TPPdeltaARE	Arhgef28	-1.5449543	2.39E-07	1.16E-05
WT>TPPdeltaARE	Mid2	-1.5576353	1.58E-05	0.00052428
WT>TPPdeltaARE	Gm42031	-1.6722517	0.0004622	0.01090664
WT>TPPdeltaARE	Hs6st2	-1.6730688	0.00010777	0.00300139
WT>TPPdeltaARE	Selp	-1.7276402	0.00045251	0.01072606
WT>TPPdeltaARE	Gm49980	-1.9720241	2.23E-05	0.00070851
WT>TPPdeltaARE	Podxl	-1.9883942	2.14E-09	1.35E-07
WT>TPPdeltaARE	AC158975.2	-2.1821211	1.10E-05	0.00038469

WT>TPPdeltaARE	Fhdc1	-2.8934411	1.34E-05	0.00045831
WT>TPPdeltaARE	Igkv6-17	-10.753279	7.33E-08	3.82E-06
WT>TPPdeltaARE	Igkv5-39	-13.055596	1.70E-11	1.32E-09

Expression_Direction	Gene	logFC	PValue	FDR
WT<TPPdeltaARE	Ighv1-71	3.77889206	0.00119389	0.03200516
WT<TPPdeltaARE	Ndnf	2.5407146	2.24E-28	1.22E-24
WT<TPPdeltaARE	Nrsn2	2.38571458	1.32E-16	1.14E-13
WT<TPPdeltaARE	Ms4a4b	2.04390994	5.97E-05	0.00312665
WT<TPPdeltaARE	Tmc1	2.0319616	1.21E-07	1.60E-05
WT<TPPdeltaARE	Gimap3	1.94599751	4.72E-24	1.28E-20
WT<TPPdeltaARE	Eya4	1.94275929	1.50E-09	3.94E-07
WT<TPPdeltaARE	Cxcr6	1.92901294	4.95E-06	0.00039981
WT<TPPdeltaARE	Insm1	1.88953412	1.47E-07	1.90E-05
WT<TPPdeltaARE	Ubash3a	1.80966487	4.84E-07	5.51E-05
WT<TPPdeltaARE	P2rx7	1.79833885	1.17E-17	1.12E-14
WT<TPPdeltaARE	Nckap5	1.75207685	3.98E-07	4.67E-05
WT<TPPdeltaARE	Ccl3	1.67837889	2.75E-12	1.32E-09
WT<TPPdeltaARE	Siglecg	1.6749287	4.14E-10	1.21E-07
WT<TPPdeltaARE	Gimap7	1.63145256	1.63E-19	2.04E-16
WT<TPPdeltaARE	Kcnk10	1.59438713	1.53E-10	5.41E-08
WT<TPPdeltaARE	Ak5	1.53063189	3.61E-10	1.07E-07
WT<TPPdeltaARE	Dipk2b	1.51932517	4.06E-05	0.00231178
WT<TPPdeltaARE	Slc27a2	1.51541755	2.48E-06	0.00023084
WT<TPPdeltaARE	Slc38a11	1.47629872	8.62E-06	0.00061627
WT<TPPdeltaARE	Abcd2	1.45439021	2.47E-08	4.24E-06
WT<TPPdeltaARE	Abi3bp	1.43827214	3.32E-13	1.85E-10
WT<TPPdeltaARE	Kif5c	1.3510038	9.79E-07	0.0001036
WT<TPPdeltaARE	Shisa9	1.3466493	2.02E-08	3.53E-06
WT<TPPdeltaARE	1110032F04I	1.34111377	4.08E-05	0.00231178
WT<TPPdeltaARE	Trpc1	1.33328607	2.09E-11	8.50E-09
WT<TPPdeltaARE	Dusp26	1.33048028	1.19E-06	0.00012225
WT<TPPdeltaARE	Rnf39	1.31932686	9.80E-09	1.96E-06
WT<TPPdeltaARE	Igf1	1.30146148	5.50E-06	0.00043187
WT<TPPdeltaARE	Ildr1	1.29347268	4.29E-08	6.67E-06
WT<TPPdeltaARE	Bcat1	1.28299647	6.14E-09	1.35E-06
WT<TPPdeltaARE	Aldh3a1	1.27892151	2.21E-09	5.47E-07
WT<TPPdeltaARE	Stxbp6	1.26368391	5.51E-06	0.00043187
WT<TPPdeltaARE	Ndrg4	1.25798145	4.04E-09	9.28E-07
WT<TPPdeltaARE	Cyp26b1	1.25340804	2.34E-06	0.00022145
WT<TPPdeltaARE	Gm42047	1.25324656	2.91E-08	4.80E-06
WT<TPPdeltaARE	Il2rb	1.24328891	0.00095265	0.02714543
WT<TPPdeltaARE	Gm43534	1.22673128	3.09E-06	0.00027703
WT<TPPdeltaARE	Icam5	1.21677587	9.70E-08	1.34E-05
WT<TPPdeltaARE	Mab21l2	1.20562496	6.30E-11	2.33E-08
WT<TPPdeltaARE	Itgb5	1.20511041	6.01E-06	0.00046458
WT<TPPdeltaARE	Kcnb2	1.1964424	0.00070094	0.02147485
WT<TPPdeltaARE	Luzp2	1.19448491	6.76E-06	0.00051228
WT<TPPdeltaARE	Pls1	1.18546904	6.29E-07	6.93E-05

WT<TPPdeltaARE	Gas6	1.17805779	5.15E-14	3.36E-11
WT<TPPdeltaARE	Gm4117	1.16494049	0.0001162	0.00530508
WT<TPPdeltaARE	Creb5	1.16334741	0.0013669	0.03505028
WT<TPPdeltaARE	Gpr150	1.15508178	4.43E-07	5.08E-05
WT<TPPdeltaARE	Hopx	1.13200746	2.96E-09	7.00E-07
WT<TPPdeltaARE	Card11	1.13079987	7.53E-09	1.57E-06
WT<TPPdeltaARE	Tgm5	1.12933906	7.67E-08	1.12E-05
WT<TPPdeltaARE	Myl10	1.12733424	1.27E-12	6.29E-10
WT<TPPdeltaARE	Mcc	1.11690642	2.95E-08	4.81E-06
WT<TPPdeltaARE	Sidt1	1.11664876	1.79E-09	4.49E-07
WT<TPPdeltaARE	Psg16	1.11152728	3.48E-06	0.00029831
WT<TPPdeltaARE	Zan	1.08596364	0.00190494	0.04435455
WT<TPPdeltaARE	Jakmip1	1.08152456	8.24E-06	0.00059934
WT<TPPdeltaARE	Plek	1.08051308	6.03E-08	9.10E-06
WT<TPPdeltaARE	Synpo2	1.07238177	0.00024759	0.00960572
WT<TPPdeltaARE	Ank2	1.07237733	7.47E-05	0.00374415
WT<TPPdeltaARE	Yap1	1.06978627	8.01E-06	0.00058571
WT<TPPdeltaARE	Fcer2a	1.06914022	5.23E-06	0.00041393
WT<TPPdeltaARE	Clec4g	1.06635504	1.55E-08	2.85E-06
WT<TPPdeltaARE	Car12	1.06544071	0.00012403	0.00558449
WT<TPPdeltaARE	Gm37509	1.06042936	2.76E-07	3.38E-05
WT<TPPdeltaARE	Ncam2	1.05754776	3.82E-06	0.00032029
WT<TPPdeltaARE	Schip1	1.05114039	9.35E-07	9.96E-05
WT<TPPdeltaARE	Sema4a	1.04595139	0.00042607	0.01462006
WT<TPPdeltaARE	Sbspon	1.04249758	4.17E-08	6.53E-06
WT<TPPdeltaARE	Hopxos	1.0418325	4.08E-05	0.00231178
WT<TPPdeltaARE	Tnfrsf25	1.03771885	4.16E-08	6.53E-06
WT<TPPdeltaARE	Slc17a8	1.03671636	8.06E-10	2.27E-07
WT<TPPdeltaARE	Fbp1	1.01685062	3.42E-10	1.06E-07
WT<TPPdeltaARE	Hoxc6	1.00807545	7.12E-05	0.00361665
WT<TPPdeltaARE	Zfp36	1.00672337	5.69E-11	2.16E-08
WT<TPPdeltaARE	Islr	1.00211308	1.53E-08	2.83E-06
WT<TPPdeltaARE	Cfap57	0.99138201	0.00079489	0.02381611
WT<TPPdeltaARE	Lamb3	0.98176743	3.19E-05	0.00187074
WT<TPPdeltaARE	Snx31	0.97197143	1.42E-08	2.65E-06
WT<TPPdeltaARE	Prom2	0.97083878	6.33E-12	2.71E-09
WT<TPPdeltaARE	Cd28	0.97039112	1.97E-06	0.00019006
WT<TPPdeltaARE	Tmem255b	0.96586793	7.95E-07	8.58E-05
WT<TPPdeltaARE	Chst2	0.9635125	2.81E-06	0.00025705
WT<TPPdeltaARE	Tubg2	0.9622439	8.29E-06	0.00059954
WT<TPPdeltaARE	Dock4	0.95714317	0.00029953	0.01114625
WT<TPPdeltaARE	Esrrg	0.95289646	0.00062512	0.0197458
WT<TPPdeltaARE	Gm14964	0.95258355	2.63E-09	6.39E-07
WT<TPPdeltaARE	Ptprg	0.95100477	9.81E-05	0.00463463
WT<TPPdeltaARE	Dok3	0.94488487	5.82E-05	0.00305789

WT<TPPdeltaARE	Gm15816	0.93902511	0.00047792	0.0159623
WT<TPPdeltaARE	Gem	0.93805643	2.95E-09	7.00E-07
WT<TPPdeltaARE	Xdh	0.93695032	7.91E-09	1.63E-06
WT<TPPdeltaARE	Cx3cl1	0.93638731	8.15E-08	1.16E-05
WT<TPPdeltaARE	Unc5cl	0.93381149	1.29E-06	0.00013086
WT<TPPdeltaARE	Rab19	0.93308121	6.58E-09	1.41E-06
WT<TPPdeltaARE	Ebi3	0.9180567	1.01E-05	0.00070325
WT<TPPdeltaARE	Otos	0.91374316	9.79E-08	1.34E-05
WT<TPPdeltaARE	Mmp15	0.91068409	0.00075049	0.02263615
WT<TPPdeltaARE	Adora2b	0.91057557	6.46E-05	0.00334758
WT<TPPdeltaARE	Bhlhe41	0.90468402	1.75E-05	0.00115336
WT<TPPdeltaARE	Tmem30b	0.90428026	0.00050155	0.01649464
WT<TPPdeltaARE	Rorb	0.90133905	0.00021281	0.00862813
WT<TPPdeltaARE	Tbx1	0.89672432	1.23E-05	0.00083163
WT<TPPdeltaARE	Spry4	0.89473637	1.21E-07	1.60E-05
WT<TPPdeltaARE	Ovgp1	0.893256	0.00013149	0.00583951
WT<TPPdeltaARE	Slc16a11	0.880129	0.00013039	0.00580869
WT<TPPdeltaARE	Celf4	0.8792585	1.10E-05	0.00076388
WT<TPPdeltaARE	Scube3	0.87795666	0.00062066	0.01964309
WT<TPPdeltaARE	Elovl4	0.87753064	0.00029787	0.01110977
WT<TPPdeltaARE	Ciart	0.8673591	0.00019976	0.00824254
WT<TPPdeltaARE	Gimap4	0.86604523	1.83E-08	3.30E-06
WT<TPPdeltaARE	Emp2	0.86595057	3.01E-07	3.67E-05
WT<TPPdeltaARE	Slamf8	0.8642384	0.00013378	0.0059252
WT<TPPdeltaARE	Proser2	0.85908559	0.00017931	0.00755184
WT<TPPdeltaARE	Rdh10	0.85641297	6.71E-08	9.94E-06
WT<TPPdeltaARE	Dzank1	0.85197486	0.00074782	0.02261347
WT<TPPdeltaARE	Net1	0.85184346	7.45E-08	1.09E-05
WT<TPPdeltaARE	Chrm3	0.84500254	0.00186503	0.04361281
WT<TPPdeltaARE	Ank1	0.84199782	4.68E-05	0.00263033
WT<TPPdeltaARE	Flrt3	0.84100365	0.00032997	0.01203186
WT<TPPdeltaARE	Gm28053	0.83764067	0.00010889	0.00501331
WT<TPPdeltaARE	Bcl6b	0.83601038	5.04E-05	0.00277359
WT<TPPdeltaARE	Nrap	0.83550176	5.75E-05	0.00304026
WT<TPPdeltaARE	Itgb7	0.83456642	2.01E-07	2.56E-05
WT<TPPdeltaARE	Gm42686	0.82842959	8.91E-05	0.0043304
WT<TPPdeltaARE	Fbxo2	0.82597901	0.00045682	0.01540933
WT<TPPdeltaARE	Cd200r4	0.82196688	4.59E-06	0.00037199
WT<TPPdeltaARE	Gm16712	0.82158517	7.30E-05	0.00369574
WT<TPPdeltaARE	Camk1d	0.81913777	4.78E-05	0.00266941
WT<TPPdeltaARE	Nr0b2	0.81497038	1.63E-06	0.00016033
WT<TPPdeltaARE	Nrxn2	0.81495851	7.40E-06	0.00055039
WT<TPPdeltaARE	Glt8d2	0.81051246	7.34E-05	0.00370382
WT<TPPdeltaARE	Rasef	0.8090759	0.00051761	0.01690695
WT<TPPdeltaARE	Hbb-bt	0.8089415	0.00058716	0.01876479

WT<TPPdeltaARE	Sox5	0.80749379	1.69E-06	0.00016457
WT<TPPdeltaARE	Gm49871	0.80274388	0.00125767	0.03333127
WT<TPPdeltaARE	Ttc34	0.80260731	0.0005015	0.01649464
WT<TPPdeltaARE	Il18r1	0.80014714	0.00020157	0.00829647
WT<TPPdeltaARE	Slc1a1	0.79146268	0.0010579	0.0293744
WT<TPPdeltaARE	Sh3gl3	0.7908446	5.77E-05	0.0030445
WT<TPPdeltaARE	Xlr4a	0.79026857	0.00054514	0.01773487
WT<TPPdeltaARE	Nsun7	0.78365663	0.00027897	0.01050109
WT<TPPdeltaARE	Dusp4	0.77298494	6.33E-08	9.47E-06
WT<TPPdeltaARE	Kcnj2	0.7641131	0.00170519	0.04111376
WT<TPPdeltaARE	Slc25a21	0.75940776	0.00170825	0.04112674
WT<TPPdeltaARE	Gm11802	0.75909169	4.14E-07	4.83E-05
WT<TPPdeltaARE	P2ry10	0.75765556	0.00202677	0.04665866
WT<TPPdeltaARE	Pvt1	0.7567806	0.00096253	0.02733145
WT<TPPdeltaARE	Cd86	0.75240622	0.00062917	0.01979686
WT<TPPdeltaARE	Il1r2	0.75160868	0.00201114	0.04636441
WT<TPPdeltaARE	Rps6kl1	0.74968104	0.00170316	0.04111376
WT<TPPdeltaARE	Pcbp4	0.74847666	6.20E-05	0.0032379
WT<TPPdeltaARE	Ehd2	0.74641423	2.00E-05	0.00129367
WT<TPPdeltaARE	Pcdhb22	0.74471928	0.00209701	0.04785238
WT<TPPdeltaARE	Gpr160	0.74408305	1.40E-06	0.0001406
WT<TPPdeltaARE	Kazald1	0.73672306	4.29E-07	4.96E-05
WT<TPPdeltaARE	Haao	0.72845378	0.00127985	0.03353749
WT<TPPdeltaARE	4930539E08I	0.72520718	0.00072359	0.02204454
WT<TPPdeltaARE	A530010L16I	0.72163024	0.0010238	0.02871736
WT<TPPdeltaARE	Pdgfc	0.717863	0.00010005	0.00468612
WT<TPPdeltaARE	Gm20528	0.71721344	2.93E-05	0.00176518
WT<TPPdeltaARE	Pir	0.71594904	0.0020901	0.04777918
WT<TPPdeltaARE	Bpifb5	0.71483189	0.00017606	0.00747304
WT<TPPdeltaARE	Plk2	0.7121169	0.00049419	0.01633838
WT<TPPdeltaARE	Ugt1a7c	0.70805577	3.42E-06	0.00029615
WT<TPPdeltaARE	Papss2	0.70762524	0.00209918	0.04785238
WT<TPPdeltaARE	Atp8b5	0.70714564	0.00010277	0.00479544
WT<TPPdeltaARE	Gm11511	0.70583128	0.00218899	0.04948462
WT<TPPdeltaARE	Gm35028	0.70180653	0.00039844	0.01393603
WT<TPPdeltaARE	Pglyrp1	0.69921902	3.03E-06	0.00027609
WT<TPPdeltaARE	AB124611	0.69818873	0.00128755	0.03361457
WT<TPPdeltaARE	Lsp1	0.69289168	8.44E-07	9.05E-05
WT<TPPdeltaARE	Tnfaip8l2	0.69278172	4.70E-05	0.00263052
WT<TPPdeltaARE	Fosl1	0.69003407	0.00045758	0.01540933
WT<TPPdeltaARE	2900011O08	0.68751089	6.28E-07	6.93E-05
WT<TPPdeltaARE	Ankrd29	0.6869339	0.00119378	0.03200516
WT<TPPdeltaARE	Slc6a13	0.68580617	2.73E-05	0.0016819
WT<TPPdeltaARE	Spo11	0.68529319	8.96E-06	0.00063802
WT<TPPdeltaARE	Fam171b	0.68449496	8.36E-06	0.00060054

WT<TPPdeltaARE	Mroh2a	0.68154363	0.00086804	0.02544646
WT<TPPdeltaARE	Hpgds	0.68150086	1.13E-05	0.00077085
WT<TPPdeltaARE	Ccl4	0.68146346	0.00045099	0.0152503
WT<TPPdeltaARE	Ablim1	0.67948483	3.27E-05	0.00189755
WT<TPPdeltaARE	Gab3	0.67336795	0.00026624	0.01013877
WT<TPPdeltaARE	Car11	0.66471027	0.00100429	0.02822224
WT<TPPdeltaARE	Mc5r	0.66443377	0.00108579	0.02999548
WT<TPPdeltaARE	Insig1	0.66347524	9.81E-05	0.00463463
WT<TPPdeltaARE	4833407H14	0.66260417	2.32E-05	0.00145262
WT<TPPdeltaARE	Gm37549	0.65995602	0.00064305	0.02011728
WT<TPPdeltaARE	Gbp9	0.65903866	1.11E-05	0.00076423
WT<TPPdeltaARE	Anxa6	0.65701615	7.11E-06	0.00053672
WT<TPPdeltaARE	Hk3	0.65484224	8.82E-08	1.24E-05
WT<TPPdeltaARE	Thrb	0.65390596	1.45E-05	0.00096667
WT<TPPdeltaARE	C130026I21R	0.64995965	0.00129104	0.03361457
WT<TPPdeltaARE	Gm15701	0.64806638	0.00087988	0.02565501
WT<TPPdeltaARE	Rab34	0.64573268	3.25E-05	0.00189173
WT<TPPdeltaARE	Sec16b	0.64360089	0.00023463	0.00928077
WT<TPPdeltaARE	9930111J21R	0.64299875	0.00018367	0.00769585
WT<TPPdeltaARE	Ckb	0.64002082	4.03E-05	0.00230713
WT<TPPdeltaARE	4833418N02	0.64001404	0.00033881	0.01221721
WT<TPPdeltaARE	Gfi1	0.63947215	7.36E-06	0.00055018
WT<TPPdeltaARE	Ghdc	0.63384449	0.00025029	0.00966686
WT<TPPdeltaARE	A530040E14	0.63345152	5.14E-05	0.00282226
WT<TPPdeltaARE	Zbtb7b	0.6321812	0.00020511	0.00841267
WT<TPPdeltaARE	Arhgap30	0.63130119	9.58E-05	0.00456357
WT<TPPdeltaARE	Uchl1	0.62793708	0.00024254	0.00950267
WT<TPPdeltaARE	Bgn	0.62759013	7.89E-06	0.00057911
WT<TPPdeltaARE	Adrb2	0.62651523	0.00022529	0.00901855
WT<TPPdeltaARE	Tox	0.62410044	3.68E-05	0.00211387
WT<TPPdeltaARE	Gjb3	0.62073076	0.00016082	0.00693458
WT<TPPdeltaARE	Rasa4	0.62021692	0.00023517	0.00928077
WT<TPPdeltaARE	Abcb1a	0.61698256	0.00019748	0.00819001
WT<TPPdeltaARE	Krt80	0.61683695	0.00066251	0.02053193
WT<TPPdeltaARE	Gpx3	0.61646211	0.00036623	0.01297663
WT<TPPdeltaARE	Trim58	0.61611476	0.00047331	0.01584079
WT<TPPdeltaARE	Clec14a	0.61493522	0.0003409	0.01226576
WT<TPPdeltaARE	Gm15411	0.61486564	0.00011149	0.0051187
WT<TPPdeltaARE	Ankmy1	0.61259823	0.00054699	0.01775968
WT<TPPdeltaARE	Clec9a	0.6078748	0.00014485	0.00631238
WT<TPPdeltaARE	Fam20c	0.60540754	0.00105619	0.0293744
WT<TPPdeltaARE	Bcam	0.60150553	5.81E-06	0.0004529
WT<TPPdeltaARE	Gm9877	0.5945446	0.00154671	0.03848833
WT<TPPdeltaARE	Triqk	0.59291176	1.60E-05	0.00105834
WT<TPPdeltaARE	Gadd45a	0.59179298	0.00083288	0.02463729

WT<TPPdeltaARE	Ntn4	0.58830267	0.00070658	0.02160713
WT<TPPdeltaARE	Sytl1	0.58757097	3.29E-06	0.00028798
WT<TPPdeltaARE	Trib3	0.58687791	0.00033716	0.01218489
WT<TPPdeltaARE	Gsn	0.58604899	9.05E-05	0.00436779
WT<TPPdeltaARE	Gm35551	0.58542331	0.00016833	0.00718236
WT<TPPdeltaARE	Maf	0.5852734	0.00019973	0.00824254
WT<TPPdeltaARE	Car2	0.58178613	1.56E-05	0.00103081
WT<TPPdeltaARE	Lmcd1	0.58050456	0.00164278	0.03996369
WT<TPPdeltaARE	Akap17b	0.58032968	0.00097885	0.02765045
WT<TPPdeltaARE	Mmp16	0.57377012	0.00066261	0.02053193
WT<TPPdeltaARE	Arhgap15	0.57059375	1.83E-05	0.00118557
WT<TPPdeltaARE	Arhgap15os	0.56807882	0.00115869	0.0315284
WT<TPPdeltaARE	Zc3h6	0.56667512	0.000667	0.02062882
WT<TPPdeltaARE	Ppp1r15a	0.56659199	0.00110514	0.03032448
WT<TPPdeltaARE	Ifitm7	0.56508892	0.00010306	0.00479544
WT<TPPdeltaARE	Smad6	0.56426585	0.0003548	0.01263831
WT<TPPdeltaARE	Vegfa	0.56059421	0.00018521	0.00774032
WT<TPPdeltaARE	Bdh2	0.55543971	0.00046075	0.01548389
WT<TPPdeltaARE	Npas2	0.55501778	0.000321	0.01181047
WT<TPPdeltaARE	Gucy1a1	0.55358702	0.00010795	0.00498727
WT<TPPdeltaARE	Osgin1	0.55113539	2.69E-05	0.00166585
WT<TPPdeltaARE	Cd276	0.550433	0.00130513	0.03387305
WT<TPPdeltaARE	Pqlc3	0.54790699	9.45E-05	0.00451891
WT<TPPdeltaARE	Alox12	0.54759153	0.00155093	0.03853452
WT<TPPdeltaARE	Cirbp	0.5475227	0.00035247	0.01259851
WT<TPPdeltaARE	Gm6634	0.54450014	0.0022204	0.04994133
WT<TPPdeltaARE	Tinagl1	0.54236703	0.00016761	0.0071757
WT<TPPdeltaARE	Rora	0.53833103	0.00024811	0.00960572
WT<TPPdeltaARE	Gm10499	0.53704343	0.00013044	0.00580869
WT<TPPdeltaARE	Padi4	0.53486016	2.05E-05	0.00130926
WT<TPPdeltaARE	Gm15441	0.53433036	0.00126529	0.03342461
WT<TPPdeltaARE	Txnip	0.53172384	0.00140235	0.0356581
WT<TPPdeltaARE	5330406M23	0.53051644	0.00179761	0.042586
WT<TPPdeltaARE	Aspa	0.52942363	0.00119261	0.03200516
WT<TPPdeltaARE	Tpm4	0.52629082	0.0002773	0.01046211
WT<TPPdeltaARE	Zbtb46	0.52517778	0.00043801	0.01496683
WT<TPPdeltaARE	Gm13881	0.52470607	0.0019155	0.04447405
WT<TPPdeltaARE	Cav2	0.52371388	2.79E-05	0.00169698
WT<TPPdeltaARE	Clec1a	0.52091632	0.00087086	0.02548322
WT<TPPdeltaARE	Clip3	0.52041611	0.00041408	0.01429907
WT<TPPdeltaARE	Cdkn1c	0.50950655	0.00011981	0.00543927
WT<TPPdeltaARE	Mirt1	0.50807934	0.00080793	0.02407402
WT<TPPdeltaARE	Gas2l3	0.50382611	0.0001239	0.00558449
WT<TPPdeltaARE	Cd24a	0.50369941	2.79E-05	0.00169698
WT<TPPdeltaARE	Rgcc	0.49956008	0.00176911	0.04211942

WT<TPPdeltaARE	Pygm	0.49922411	0.00092973	0.02658537
WT<TPPdeltaARE	Lzts1	0.49573501	0.0016238	0.03956097
WT<TPPdeltaARE	Pkn3	0.49534782	0.00103194	0.02882899
WT<TPPdeltaARE	Slc25a33	0.49482316	0.00117747	0.03187983
WT<TPPdeltaARE	Gpc1	0.49246756	0.00023262	0.00922505
WT<TPPdeltaARE	Pde3b	0.490162	0.0004412	0.01500944
WT<TPPdeltaARE	Tef	0.49013409	0.00071101	0.02170177
WT<TPPdeltaARE	Apobr	0.48598743	0.00092515	0.02654511
WT<TPPdeltaARE	P2ry14	0.48560103	0.000958	0.02725021
WT<TPPdeltaARE	H2-T-ps	0.48525974	0.00182021	0.04299644
WT<TPPdeltaARE	Etv5	0.47479322	0.00041198	0.01425643
WT<TPPdeltaARE	Syne1	0.47459224	0.00102543	0.02871736
WT<TPPdeltaARE	Cyth1	0.47305039	0.00073548	0.02232317
WT<TPPdeltaARE	Fam169b	0.47204183	0.00127372	0.03343061
WT<TPPdeltaARE	Diaph3	0.47131115	0.0015811	0.03886922
WT<TPPdeltaARE	St3gal6	0.46950254	0.00158859	0.03893607
WT<TPPdeltaARE	Ppcdc	0.45690665	0.0002465	0.00960572
WT<TPPdeltaARE	Gm44732	0.4567649	0.00118209	0.03189881
WT<TPPdeltaARE	Camk2d	0.45380964	0.00034334	0.01232625
WT<TPPdeltaARE	Tnip3	0.44585242	0.00114942	0.03137766
WT<TPPdeltaARE	Sh2d3c	0.43900626	0.00088601	0.02574157
WT<TPPdeltaARE	Pcsk4	0.43135649	0.00156608	0.03867509
WT<TPPdeltaARE	Il1rapl2	0.42853914	0.00091374	0.02631848
WT<TPPdeltaARE	Traip	0.42473045	0.00208603	0.047753
WT<TPPdeltaARE	Stk26	0.42360999	0.00183276	0.04315251
WT<TPPdeltaARE	Pik3r1	0.42318033	0.00136788	0.03505028
WT<TPPdeltaARE	Procr	0.42116227	0.00064039	0.02007263
WT<TPPdeltaARE	Fcho1	0.42034222	0.0006806	0.02096993
WT<TPPdeltaARE	Il21r	0.4201405	0.00177016	0.04211942
WT<TPPdeltaARE	Glipr2	0.41969113	0.00129779	0.03373637
WT<TPPdeltaARE	Trpv2	0.41727844	0.00065019	0.02030172
WT<TPPdeltaARE	Slamf1	0.4112211	0.00143237	0.03619563
WT<TPPdeltaARE	Gm2a	0.41082111	0.00039258	0.01376048
WT<TPPdeltaARE	Stap1	0.39926145	0.00060734	0.01929644
WT<TPPdeltaARE	Hoxa9	0.37488991	0.00158514	0.03891001
WT<TPPdeltaARE	Traf3ip3	0.35425548	0.00200185	0.04621553
WT>TPPdeltaARE	Heatr1	-0.3766191	0.00161413	0.03938422
WT>TPPdeltaARE	Jak3	-0.3893445	0.00153645	0.03835007
WT>TPPdeltaARE	Cbx5	-0.4056573	0.00157762	0.03884236
WT>TPPdeltaARE	Peg13	-0.4062502	0.00131816	0.03408848
WT>TPPdeltaARE	Eps8	-0.4416569	0.00160865	0.03936849
WT>TPPdeltaARE	Cad	-0.4478675	0.00090353	0.02615752
WT>TPPdeltaARE	Trim62	-0.4483008	0.00109131	0.03008257
WT>TPPdeltaARE	Foxk2	-0.4515475	0.00222145	0.04994133
WT>TPPdeltaARE	Pbx1	-0.4613583	0.001191	0.03200516

WT>TPPdeltaARE	Nckipsd	-0.4761151	0.00056556	0.01814567
WT>TPPdeltaARE	Il1r1	-0.479363	0.00109783	0.03017448
WT>TPPdeltaARE	Gm15657	-0.5096594	0.00028162	0.01055189
WT>TPPdeltaARE	Xbp1	-0.5110384	0.00032233	0.01183112
WT>TPPdeltaARE	Cxcr4	-0.5120929	0.00087831	0.02565501
WT>TPPdeltaARE	Rhoj	-0.5145321	0.00014451	0.00631238
WT>TPPdeltaARE	Fry	-0.5226979	0.00098425	0.02775488
WT>TPPdeltaARE	S1pr1	-0.524265	0.00021158	0.00862138
WT>TPPdeltaARE	Cdc6	-0.5313505	0.00164931	0.04000303
WT>TPPdeltaARE	Podxl	-0.5406137	9.67E-05	0.00459709
WT>TPPdeltaARE	Gm26885	-0.5461363	0.00025761	0.00987576
WT>TPPdeltaARE	1700025G04	-0.5478498	0.0006353	0.01995139
WT>TPPdeltaARE	Fgfr1	-0.5555782	7.91E-05	0.00389619
WT>TPPdeltaARE	Pdxp	-0.5577216	0.00096654	0.02735002
WT>TPPdeltaARE	Pim1	-0.563565	0.00040171	0.01402033
WT>TPPdeltaARE	Pcdhgc3	-0.5779858	0.00108174	0.02998519
WT>TPPdeltaARE	Pclo	-0.5792308	0.00092669	0.02654511
WT>TPPdeltaARE	Dhx40	-0.5817347	0.00082252	0.02441948
WT>TPPdeltaARE	Ak4	-0.5850053	6.49E-05	0.00334758
WT>TPPdeltaARE	Gm16587	-0.5902251	0.00210337	0.04788093
WT>TPPdeltaARE	Nsg1	-0.5931466	0.00014242	0.00624002
WT>TPPdeltaARE	Chek1	-0.5957387	0.00143201	0.03619563
WT>TPPdeltaARE	Ppp1r3d	-0.5957774	5.54E-05	0.00298165
WT>TPPdeltaARE	Klhl13	-0.5991213	0.00150812	0.03775854
WT>TPPdeltaARE	Adgrl1	-0.6017367	0.00171753	0.04122834
WT>TPPdeltaARE	Ajuba	-0.6100772	0.00051596	0.01688688
WT>TPPdeltaARE	Gm16548	-0.6126638	0.00049043	0.01627997
WT>TPPdeltaARE	Cldn5	-0.6175679	0.00012503	0.00561371
WT>TPPdeltaARE	Brca1	-0.6212511	0.00050395	0.01652695
WT>TPPdeltaARE	Rbm19	-0.6213358	8.49E-05	0.00414072
WT>TPPdeltaARE	Dynll1	-0.6261516	0.0014546	0.03647465
WT>TPPdeltaARE	Mcm10	-0.6294763	0.00109264	0.03008257
WT>TPPdeltaARE	Ecm2	-0.6300073	0.00091394	0.02631848
WT>TPPdeltaARE	3-Mar	-0.6343983	7.67E-05	0.00380925
WT>TPPdeltaARE	Muc13	-0.6353874	3.35E-05	0.00193597
WT>TPPdeltaARE	Selp	-0.6405145	0.00022433	0.00901855
WT>TPPdeltaARE	Gm19265	-0.6421183	0.00190764	0.04435455
WT>TPPdeltaARE	Tspan12	-0.6466574	0.00083062	0.02461503
WT>TPPdeltaARE	Prkca	-0.6525854	3.00E-05	0.00177967
WT>TPPdeltaARE	Zfp948	-0.6614111	0.00013886	0.00611692
WT>TPPdeltaARE	Nav1	-0.6614161	4.34E-05	0.00244698
WT>TPPdeltaARE	Mmp14	-0.6666806	9.45E-06	0.00066674
WT>TPPdeltaARE	Per1	-0.6697549	9.97E-05	0.00468612
WT>TPPdeltaARE	E2f1	-0.6734541	0.00136185	0.03501059
WT>TPPdeltaARE	Plbd1	-0.6738454	0.00010801	0.00498727

WT>TPPdeltaARE	Psma8	-0.6773466	2.09E-05	0.00132864
WT>TPPdeltaARE	Sdc3	-0.681543	2.25E-05	0.00141742
WT>TPPdeltaARE	Igsf10	-0.684523	2.97E-05	0.00177967
WT>TPPdeltaARE	E2f7	-0.6968328	0.00048226	0.01607446
WT>TPPdeltaARE	Gstm2	-0.6973893	8.31E-06	0.00059954
WT>TPPdeltaARE	Cd34	-0.7008977	6.17E-07	6.89E-05
WT>TPPdeltaARE	Actn1	-0.7014291	2.84E-05	0.0017157
WT>TPPdeltaARE	Cdk6	-0.7016848	0.00024129	0.00947643
WT>TPPdeltaARE	Serpinb1a	-0.7024136	1.09E-06	0.00011369
WT>TPPdeltaARE	Ifi203-ps	-0.7046038	0.00079924	0.02390254
WT>TPPdeltaARE	Plppr2	-0.7050416	0.00113144	0.03099392
WT>TPPdeltaARE	Igfbp7	-0.7306176	7.49E-05	0.00374415
WT>TPPdeltaARE	Slc12a4	-0.7379015	0.00040634	0.01415156
WT>TPPdeltaARE	Slfn9	-0.7409602	7.57E-06	0.00056058
WT>TPPdeltaARE	Gm16897	-0.7511859	1.29E-05	0.00086359
WT>TPPdeltaARE	Cebpd	-0.7558198	1.77E-05	0.00115987
WT>TPPdeltaARE	Grhl1	-0.7558749	0.0003846	0.01351
WT>TPPdeltaARE	Clec4e	-0.7580173	1.30E-05	0.00086599
WT>TPPdeltaARE	Pawr	-0.7681623	0.00017775	0.00751853
WT>TPPdeltaARE	Scara3	-0.76868	0.00172638	0.04131907
WT>TPPdeltaARE	Pde9a	-0.7701465	2.01E-05	0.00129763
WT>TPPdeltaARE	Cxadr	-0.7739009	0.00065547	0.02042734
WT>TPPdeltaARE	Csf3r	-0.7789142	2.48E-06	0.00023084
WT>TPPdeltaARE	Gpx8	-0.7854194	0.00030214	0.0112178
WT>TPPdeltaARE	Gpc3	-0.786977	0.00144487	0.03639864
WT>TPPdeltaARE	Ncam1	-0.7878295	0.00024745	0.00960572
WT>TPPdeltaARE	Gm16175	-0.8009067	0.00056506	0.01814567
WT>TPPdeltaARE	Spint1	-0.8010544	0.00203817	0.04685491
WT>TPPdeltaARE	Gm37274	-0.8070302	0.00044053	0.01500944
WT>TPPdeltaARE	Rps4l	-0.8087784	3.09E-06	0.00027703
WT>TPPdeltaARE	Shroom4	-0.8095048	7.79E-06	0.00057421
WT>TPPdeltaARE	Slc16a4	-0.814832	0.00031573	0.01165789
WT>TPPdeltaARE	Steap3	-0.8350282	0.00115123	0.03137766
WT>TPPdeltaARE	B4galt2	-0.8383395	0.0018645	0.04361281
WT>TPPdeltaARE	Rin1	-0.8493851	0.00044294	0.01500944
WT>TPPdeltaARE	Rasgef1b	-0.8511978	2.57E-08	4.37E-06
WT>TPPdeltaARE	Gm48375	-0.8592305	0.00028365	0.01060385
WT>TPPdeltaARE	BC037039	-0.8604001	3.11E-05	0.00183648
WT>TPPdeltaARE	Ldb2	-0.8677786	0.000191	0.00794153
WT>TPPdeltaARE	Fermt2	-0.8744862	0.00178304	0.04236407
WT>TPPdeltaARE	Cnr2	-0.8767879	0.00055857	0.01802801
WT>TPPdeltaARE	Gm37035	-0.8796461	8.93E-05	0.0043304
WT>TPPdeltaARE	Gm26716	-0.8801714	0.00026838	0.01019672
WT>TPPdeltaARE	Ggt5	-0.8818907	0.00011443	0.00523903
WT>TPPdeltaARE	Bcl3	-0.8931617	4.87E-05	0.00270859

WT>TPPdeltaARE	Gm37194	-0.8973613	0.00016588	0.00713379
WT>TPPdeltaARE	P2ry1	-0.897829	7.62E-07	8.28E-05
WT>TPPdeltaARE	Nradd	-0.8994183	7.17E-06	0.00053834
WT>TPPdeltaARE	Insl3	-0.9011634	0.0001825	0.00766641
WT>TPPdeltaARE	Epas1	-0.9011816	5.58E-05	0.00299169
WT>TPPdeltaARE	Myo1e	-0.9110808	9.88E-07	0.00010393
WT>TPPdeltaARE	Serpinh1	-0.9110841	2.71E-08	4.56E-06
WT>TPPdeltaARE	Rps6ka3	-0.9113099	3.22E-08	5.15E-06
WT>TPPdeltaARE	Gm10644	-0.9129601	0.00022311	0.00900111
WT>TPPdeltaARE	Nxpe2	-0.9154699	1.80E-05	0.00117557
WT>TPPdeltaARE	Tsix	-0.9197409	9.98E-05	0.00468612
WT>TPPdeltaARE	Gipc2	-0.9252962	0.00011744	0.00534676
WT>TPPdeltaARE	A730091E23	-0.9289061	5.87E-06	0.0004554
WT>TPPdeltaARE	Gm37524	-0.9369879	0.00022485	0.00901855
WT>TPPdeltaARE	Nrtn	-0.9418843	0.0007429	0.0225065
WT>TPPdeltaARE	Slc22a3	-0.9431194	1.34E-07	1.76E-05
WT>TPPdeltaARE	Fcrla	-0.9500555	0.00103296	0.02882899
WT>TPPdeltaARE	Flt4	-0.9747606	7.96E-05	0.00390694
WT>TPPdeltaARE	Pim2	-0.9814792	4.43E-06	0.00036318
WT>TPPdeltaARE	Plac8	-0.9943274	5.71E-05	0.00302997
WT>TPPdeltaARE	Itga2b	-0.9951582	3.57E-10	1.07E-07
WT>TPPdeltaARE	Igha	-1.0014902	0.00138346	0.03528784
WT>TPPdeltaARE	Gm35189	-1.0018896	5.00E-06	0.00040137
WT>TPPdeltaARE	Oas2	-1.0057432	0.00050195	0.01649464
WT>TPPdeltaARE	Ffar2	-1.0064394	5.07E-06	0.00040502
WT>TPPdeltaARE	Adcy4	-1.0131894	0.00024529	0.00958763
WT>TPPdeltaARE	Xist	-1.017582	1.24E-06	0.00012643
WT>TPPdeltaARE	Pak6	-1.0299033	0.00038016	0.01341185
WT>TPPdeltaARE	Gm13431	-1.0313303	0.00032302	0.01183112
WT>TPPdeltaARE	Tjp1	-1.0319665	2.16E-05	0.00136779
WT>TPPdeltaARE	Plxnb2	-1.0351776	3.07E-13	1.79E-10
WT>TPPdeltaARE	Gm5833	-1.0373878	6.35E-09	1.38E-06
WT>TPPdeltaARE	Fgr	-1.0539995	2.98E-08	4.81E-06
WT>TPPdeltaARE	Lgmn	-1.084784	2.57E-07	3.17E-05
WT>TPPdeltaARE	Rian	-1.0955763	5.67E-05	0.00302575
WT>TPPdeltaARE	Map3k6	-1.1095101	0.00014212	0.00624002
WT>TPPdeltaARE	Gm11149	-1.1104266	3.52E-06	0.0002987
WT>TPPdeltaARE	Lrrn1	-1.1263723	5.14E-06	0.000409
WT>TPPdeltaARE	Nrep	-1.1263848	2.81E-05	0.00170151
WT>TPPdeltaARE	Pparg	-1.1312284	5.68E-05	0.00302575
WT>TPPdeltaARE	Itgb3	-1.1367958	1.51E-14	1.07E-11
WT>TPPdeltaARE	Prkd1	-1.1424471	6.40E-06	0.00048781
WT>TPPdeltaARE	Osmr	-1.1427045	4.47E-06	0.00036458
WT>TPPdeltaARE	Ripor3	-1.1474186	1.24E-05	0.00083502
WT>TPPdeltaARE	Ggt1	-1.1629385	6.24E-06	0.0004792

WT>TPPdeltaARE	Tgm1	-1.162955	1.38E-08	2.61E-06
WT>TPPdeltaARE	Igkc	-1.1690628	0.00211971	0.04818569
WT>TPPdeltaARE	Gli3	-1.1724367	0.00127368	0.03343061
WT>TPPdeltaARE	Tnfaip8l1	-1.2333158	0.00012272	0.00555615
WT>TPPdeltaARE	Depp1	-1.2372609	0.00090623	0.02618914
WT>TPPdeltaARE	Zg16	-1.250205	3.39E-06	0.00029567
WT>TPPdeltaARE	Iglv3	-1.2650115	0.00140554	0.03568366
WT>TPPdeltaARE	Spats2	-1.290759	2.35E-10	7.99E-08
WT>TPPdeltaARE	Pdgfrb	-1.2908042	2.07E-11	8.50E-09
WT>TPPdeltaARE	Col18a1	-1.3083275	2.48E-11	9.62E-09
WT>TPPdeltaARE	Gpr4	-1.3179599	7.68E-14	4.81E-11
WT>TPPdeltaARE	Rbpjl	-1.3205759	5.37E-12	2.43E-09
WT>TPPdeltaARE	Vcam1	-1.3279121	1.11E-06	0.00011472
WT>TPPdeltaARE	Mmrn2	-1.3319803	2.30E-06	0.00021966
WT>TPPdeltaARE	Stab2	-1.345092	9.06E-05	0.00436779
WT>TPPdeltaARE	Serping1	-1.3681316	3.09E-09	7.20E-07
WT>TPPdeltaARE	Abca4	-1.3725277	6.04E-12	2.66E-09
WT>TPPdeltaARE	Lox	-1.4065379	4.17E-06	0.00034539
WT>TPPdeltaARE	Fabp4	-1.4337611	3.46E-07	4.17E-05
WT>TPPdeltaARE	S100a8	-1.5013421	3.69E-06	0.0003115
WT>TPPdeltaARE	Clu	-1.5023266	1.55E-17	1.41E-14
WT>TPPdeltaARE	Slpi	-1.5191709	2.57E-05	0.00159713
WT>TPPdeltaARE	Ackr1	-1.5610075	6.26E-06	0.0004792
WT>TPPdeltaARE	S100a9	-1.5843906	1.17E-07	1.58E-05
WT>TPPdeltaARE	Sparc	-1.5844946	5.87E-09	1.31E-06
WT>TPPdeltaARE	Fzd1	-1.5870275	2.46E-08	4.24E-06
WT>TPPdeltaARE	Abcc9	-1.5872854	3.19E-06	0.0002814
WT>TPPdeltaARE	Gpm6a	-1.6220736	6.17E-10	1.76E-07
WT>TPPdeltaARE	Matn4	-1.6274574	4.41E-25	1.44E-21
WT>TPPdeltaARE	Ighj2	-1.7119167	0.00025812	0.00987576
WT>TPPdeltaARE	Socs3	-1.7259924	1.10E-08	2.13E-06
WT>TPPdeltaARE	Clca3a1	-1.7581403	8.54E-21	1.39E-17
WT>TPPdeltaARE	Fgf3	-1.7979897	2.76E-14	1.88E-11
WT>TPPdeltaARE	Gm16299	-1.8951776	1.30E-08	2.49E-06
WT>TPPdeltaARE	Gm34866	-1.9327071	5.68E-21	1.03E-17
WT>TPPdeltaARE	Plvap	-1.9415518	1.16E-07	1.58E-05
WT>TPPdeltaARE	Ushbp1	-1.9435105	1.70E-09	4.33E-07
WT>TPPdeltaARE	Tgfbi	-2.0388855	1.33E-13	8.02E-11
WT>TPPdeltaARE	Tspan7	-2.1554525	3.36E-10	1.06E-07
WT>TPPdeltaARE	Mrc1	-2.1715042	1.81E-10	6.26E-08
WT>TPPdeltaARE	Iglv1	-2.2101748	0.00033447	0.01216846
WT>TPPdeltaARE	Gm26906	-2.2203621	8.08E-15	5.99E-12
WT>TPPdeltaARE	Dlk1	-2.2219784	8.57E-22	2.00E-18
WT>TPPdeltaARE	Meg3	-2.227692	6.31E-20	9.35E-17
WT>TPPdeltaARE	Muc1	-2.2800693	9.56E-09	1.95E-06

WT>TPPdeltaARE	Igkj4	-2.4690731	0.00132684	0.03421873
WT>TPPdeltaARE	Vldlr	-2.6701872	2.11E-16	1.72E-13
WT>TPPdeltaARE	C4b	-2.6834082	5.18E-34	4.22E-30
WT>TPPdeltaARE	Lrg1	-2.8240854	6.87E-11	2.49E-08
WT>TPPdeltaARE	Igkv5-43	-2.892368	1.21E-06	0.00012367
WT>TPPdeltaARE	Tmem215	-2.9090775	1.17E-09	3.22E-07
WT>TPPdeltaARE	Rgs4	-3.56514	3.40E-13	1.85E-10
WT>TPPdeltaARE	Igkv1-117	-4.8645284	0.00161331	0.03938422
WT>TPPdeltaARE	Ighv1-55	-5.7594361	0.00020832	0.00850989
WT>TPPdeltaARE	Ighv1-34	-5.82545	0.00080246	0.02395482
WT>TPPdeltaARE	Ighv7-3	-6.1731209	0.00121766	0.03242919
WT>TPPdeltaARE	Igkv6-13	-6.8713943	9.89E-09	1.96E-06
WT>TPPdeltaARE	Igkv10-96	-7.5121406	3.12E-06	0.00027753
WT>TPPdeltaARE	Igkv6-17	-7.5231434	1.27E-09	3.44E-07
WT>TPPdeltaARE	Igkv19-93	-7.5545182	3.00E-05	0.00177967
WT>TPPdeltaARE	Ighv1-75	-7.6351054	6.43E-07	7.03E-05
WT>TPPdeltaARE	Ighv1-18	-7.6563689	2.17E-06	0.00020834
WT>TPPdeltaARE	Igkv4-59	-8.2728209	0.00164857	0.04000303
WT>TPPdeltaARE	Ighv2-2	-8.833843	7.06E-09	1.49E-06
WT>TPPdeltaARE	Ighg2c	-9.4781817	1.84E-08	3.30E-06
WT>TPPdeltaARE	Ighv5-16	-10.238275	1.06E-05	0.00073508
WT>TPPdeltaARE	Igkv9-124	-10.571607	3.34E-10	1.06E-07
WT>TPPdeltaARE	Igkv17-127	-10.963849	2.05E-26	8.34E-23
WT>TPPdeltaARE	Igkv17-121	-11.231941	1.44E-06	0.00014178
WT>TPPdeltaARE	Ighv1-22	-11.585662	6.52E-109	1.06E-104
WT>TPPdeltaARE	Ighv1-4	-11.879971	7.30E-19	8.50E-16
WT>TPPdeltaARE	Igkv5-48	-11.906767	2.42E-06	0.00022818
WT>TPPdeltaARE	Ighv1-58	-12.067973	1.42E-06	0.00014148
WT>TPPdeltaARE	Ighv14-2	-12.929963	2.43E-11	9.62E-09
WT>TPPdeltaARE	Igkv1-135	-14.666346	9.37E-08	1.31E-05
WT>TPPdeltaARE	Igkv15-103	-15.089772	2.53E-07	3.15E-05

Expression_Direction	Gene	logFC	PValue	FDR
WT<TPPdeltaARE	Ighv10-1	12.8758135	1.85E-89	2.91E-85
WT<TPPdeltaARE	Ighg3	7.66920957	6.76E-08	3.55E-06
WT<TPPdeltaARE	Slfn1	6.40299056	4.82E-57	2.53E-53
WT<TPPdeltaARE	Gpr33	5.95598847	1.09E-52	2.87E-49
WT<TPPdeltaARE	Iglc1	5.93524499	0.00154125	0.02991964
WT<TPPdeltaARE	Ifi205	5.50080942	3.91E-50	7.71E-47
WT<TPPdeltaARE	Cldn1	5.45268119	2.95E-24	6.55E-22
WT<TPPdeltaARE	Xcr1	5.42531192	8.27E-29	2.96E-26
WT<TPPdeltaARE	Iglj1	5.36752271	0.00115871	0.02375135
WT<TPPdeltaARE	Tlr13	5.27883352	3.70E-39	3.24E-36
WT<TPPdeltaARE	Mycl	5.22850575	3.62E-45	3.80E-42
WT<TPPdeltaARE	Cd209a	5.11740253	1.05E-17	1.33E-15
WT<TPPdeltaARE	Batf3	5.00531617	2.46E-34	1.44E-31
WT<TPPdeltaARE	Gpr141b	4.98323236	2.10E-27	6.35E-25
WT<TPPdeltaARE	Cxcl9	4.95958121	7.69E-62	6.06E-58
WT<TPPdeltaARE	Ccl8	4.88183914	1.61E-32	7.46E-30
WT<TPPdeltaARE	Zfp366	4.70930754	5.61E-54	1.77E-50
WT<TPPdeltaARE	Cd226	4.59524178	2.41E-42	2.24E-39
WT<TPPdeltaARE	Clec9a	4.58772838	2.10E-44	2.07E-41
WT<TPPdeltaARE	Gm36723	4.52615958	1.26E-23	2.62E-21
WT<TPPdeltaARE	Tlr11	4.4658517	1.17E-22	2.22E-20
WT<TPPdeltaARE	Olfm1	4.46571368	1.34E-33	6.60E-31
WT<TPPdeltaARE	Klrd1	4.45495025	1.70E-46	2.23E-43
WT<TPPdeltaARE	Plbd1	4.4537877	9.60E-22	1.68E-19
WT<TPPdeltaARE	Adam8	4.39860138	2.43E-38	2.02E-35
WT<TPPdeltaARE	Gramd2	4.3805796	5.93E-33	2.83E-30
WT<TPPdeltaARE	Gpr34	4.34406017	7.52E-30	3.12E-27
WT<TPPdeltaARE	Ifitm6	4.3074131	4.65E-29	1.75E-26
WT<TPPdeltaARE	Arhgap22	4.29473649	3.24E-30	1.38E-27
WT<TPPdeltaARE	Gpr141	4.27655572	3.19E-12	2.59E-10
WT<TPPdeltaARE	Prss34	4.22798625	8.99E-07	3.81E-05
WT<TPPdeltaARE	Plxdc1	4.21572594	1.14E-24	2.60E-22
WT<TPPdeltaARE	Ccr5	4.1971889	6.88E-18	8.89E-16
WT<TPPdeltaARE	Klrk1	4.1925997	2.21E-35	1.59E-32
WT<TPPdeltaARE	Igkv19-93	4.17558423	0.00212064	0.03909671
WT<TPPdeltaARE	Mefv	4.17468654	8.36E-08	4.32E-06
WT<TPPdeltaARE	Iglv1	4.16253079	0.00184838	0.03471094
WT<TPPdeltaARE	Scimp	4.13712157	2.10E-32	9.48E-30
WT<TPPdeltaARE	Ccr2	4.13018793	8.75E-51	1.97E-47
WT<TPPdeltaARE	Gm6377	4.06914574	1.61E-15	1.69E-13
WT<TPPdeltaARE	Lilra6	4.06407943	8.72E-21	1.40E-18
WT<TPPdeltaARE	Gpr35	4.06321224	2.93E-31	1.28E-28
WT<TPPdeltaARE	Slamf8	4.03328332	3.59E-38	2.83E-35
WT<TPPdeltaARE	Naaa	4.01682308	4.51E-37	3.38E-34

WT<TPPdeltaARE	Jaml	4.00337704	2.13E-18	2.89E-16
WT<TPPdeltaARE	Dnase1l3	3.98824488	6.89E-25	1.62E-22
WT<TPPdeltaARE	1-Mar	3.98627053	2.74E-28	9.40E-26
WT<TPPdeltaARE	Tlr3	3.97389772	1.09E-25	2.68E-23
WT<TPPdeltaARE	Adgrg5	3.9726857	1.19E-20	1.88E-18
WT<TPPdeltaARE	Prss30	3.942335	1.13E-25	2.73E-23
WT<TPPdeltaARE	Car1	3.92932936	1.96E-05	0.0006306
WT<TPPdeltaARE	7-Sep	3.92802453	5.09E-48	7.29E-45
WT<TPPdeltaARE	H2-Eb1	3.92637235	2.36E-54	9.32E-51
WT<TPPdeltaARE	Rab7b	3.92131368	4.93E-22	8.93E-20
WT<TPPdeltaARE	Kdr	3.92079226	2.35E-23	4.69E-21
WT<TPPdeltaARE	Phf11a	3.85297813	3.37E-24	7.38E-22
WT<TPPdeltaARE	P2ry6	3.84828269	4.62E-29	1.75E-26
WT<TPPdeltaARE	Klf2	3.83885115	1.32E-45	1.60E-42
WT<TPPdeltaARE	Ms4a4c	3.82091575	1.47E-26	4.16E-24
WT<TPPdeltaARE	Cxcr3	3.79540399	6.72E-29	2.46E-26
WT<TPPdeltaARE	Bcl2a1d	3.739132	1.23E-23	2.59E-21
WT<TPPdeltaARE	Serpinb10	3.68930375	2.76E-29	1.09E-26
WT<TPPdeltaARE	Mpeg1	3.66578404	2.05E-22	3.75E-20
WT<TPPdeltaARE	Gm15512	3.66138681	1.53E-21	2.62E-19
WT<TPPdeltaARE	Gm10684	3.63425325	3.53E-26	9.77E-24
WT<TPPdeltaARE	Mcemp1	3.61238191	2.37E-15	2.44E-13
WT<TPPdeltaARE	Pid1	3.5822274	2.12E-14	2.04E-12
WT<TPPdeltaARE	Id2	3.5686595	2.46E-35	1.69E-32
WT<TPPdeltaARE	Kmo	3.56656915	1.24E-33	6.28E-31
WT<TPPdeltaARE	Rtn1	3.56528631	7.19E-25	1.67E-22
WT<TPPdeltaARE	Atf3	3.53400727	3.83E-13	3.38E-11
WT<TPPdeltaARE	Itga8	3.50636678	2.15E-23	4.35E-21
WT<TPPdeltaARE	Rasgrp1	3.50165543	4.82E-07	2.18E-05
WT<TPPdeltaARE	Ptpro	3.49672963	1.91E-34	1.16E-31
WT<TPPdeltaARE	Ly86	3.49571811	7.11E-21	1.16E-18
WT<TPPdeltaARE	H2-Aa	3.45765793	5.06E-48	7.29E-45
WT<TPPdeltaARE	Ckb	3.45505288	3.88E-26	1.05E-23
WT<TPPdeltaARE	Sowahc	3.45196352	1.54E-19	2.25E-17
WT<TPPdeltaARE	Cd36	3.42728374	1.03E-15	1.13E-13
WT<TPPdeltaARE	Adam19	3.3972549	1.64E-11	1.27E-09
WT<TPPdeltaARE	Sema4a	3.39454029	5.44E-21	9.02E-19
WT<TPPdeltaARE	Itgax	3.38882112	1.24E-17	1.57E-15
WT<TPPdeltaARE	Il2rb	3.3760208	7.73E-15	7.62E-13
WT<TPPdeltaARE	Gm30489	3.36438132	9.36E-09	5.53E-07
WT<TPPdeltaARE	Cfh	3.34933895	5.89E-21	9.68E-19
WT<TPPdeltaARE	H2-Ab1	3.33298655	1.84E-49	3.23E-46
WT<TPPdeltaARE	Hbb-bs	3.30411685	4.51E-10	2.99E-08
WT<TPPdeltaARE	Ifi209	3.29491734	4.69E-17	5.77E-15
WT<TPPdeltaARE	Cysltr1	3.27407806	2.20E-13	2.00E-11

WT<TPPdeltaARE	Ifi211	3.25996812	3.64E-15	3.73E-13
WT<TPPdeltaARE	Zbtb46	3.2565766	3.29E-35	2.16E-32
WT<TPPdeltaARE	Cd40	3.23797631	6.97E-15	6.91E-13
WT<TPPdeltaARE	Irf8	3.2197426	3.55E-20	5.44E-18
WT<TPPdeltaARE	C1qtnf1	3.20334323	5.70E-05	0.00169154
WT<TPPdeltaARE	Cdh1	3.19136268	5.07E-05	0.00152104
WT<TPPdeltaARE	Col23a1	3.18783815	3.14E-16	3.64E-14
WT<TPPdeltaARE	Mcub	3.16930854	8.21E-24	1.75E-21
WT<TPPdeltaARE	Gprc5c	3.15087481	1.63E-13	1.52E-11
WT<TPPdeltaARE	Cd74	3.14896244	3.45E-19	4.90E-17
WT<TPPdeltaARE	F13a1	3.14352608	1.10E-05	0.00038416
WT<TPPdeltaARE	Gm14964	3.13210797	1.65E-09	1.06E-07
WT<TPPdeltaARE	Hba-a1	3.12997429	3.69E-08	2.03E-06
WT<TPPdeltaARE	1600010M07	3.09862279	7.91E-18	1.01E-15
WT<TPPdeltaARE	Hp	3.09851934	1.12E-08	6.56E-07
WT<TPPdeltaARE	Fam149a	3.09298508	1.46E-20	2.28E-18
WT<TPPdeltaARE	Pld4	3.06646426	1.52E-19	2.24E-17
WT<TPPdeltaARE	Fndc7	3.05303762	5.13E-22	9.18E-20
WT<TPPdeltaARE	Mcpt8	3.05188559	6.13E-08	3.24E-06
WT<TPPdeltaARE	Ly6i	3.01336012	5.47E-07	2.43E-05
WT<TPPdeltaARE	Ear2	3.01178483	6.34E-20	9.52E-18
WT<TPPdeltaARE	Qpct	2.99664635	5.21E-23	1.01E-20
WT<TPPdeltaARE	Asb2	2.98519055	7.75E-22	1.37E-19
WT<TPPdeltaARE	Ciita	2.9809301	4.41E-35	2.78E-32
WT<TPPdeltaARE	Fcmr	2.97315074	1.46E-05	0.00048792
WT<TPPdeltaARE	Tifab	2.95403837	5.77E-15	5.80E-13
WT<TPPdeltaARE	Fcgr4	2.94662766	1.82E-21	3.05E-19
WT<TPPdeltaARE	Aif1	2.90051773	1.37E-15	1.46E-13
WT<TPPdeltaARE	Rab39	2.88626503	5.11E-18	6.66E-16
WT<TPPdeltaARE	Hck	2.87944236	8.73E-34	4.74E-31
WT<TPPdeltaARE	Klf4	2.86770948	1.82E-15	1.89E-13
WT<TPPdeltaARE	Gpr183	2.82702165	1.12E-16	1.34E-14
WT<TPPdeltaARE	Iglc2	2.82557522	0.00034249	0.00843534
WT<TPPdeltaARE	Acvrl1	2.81485849	7.81E-27	2.28E-24
WT<TPPdeltaARE	Abca9	2.81343957	1.82E-05	0.00059111
WT<TPPdeltaARE	Strip2	2.79782737	9.96E-15	9.75E-13
WT<TPPdeltaARE	Treml4	2.78925663	9.68E-19	1.35E-16
WT<TPPdeltaARE	Ifi204	2.77150125	1.13E-21	1.97E-19
WT<TPPdeltaARE	Cd22	2.76278631	1.52E-22	2.82E-20
WT<TPPdeltaARE	Cd83	2.74435961	9.25E-08	4.71E-06
WT<TPPdeltaARE	Pmaip1	2.73737903	7.13E-11	5.11E-09
WT<TPPdeltaARE	Gm37787	2.71782768	0.00024178	0.00619707
WT<TPPdeltaARE	Ifi207	2.71023811	4.47E-19	6.29E-17
WT<TPPdeltaARE	Sema4f	2.70299673	1.28E-15	1.38E-13
WT<TPPdeltaARE	Nostrin	2.67848636	4.87E-12	3.90E-10

WT<TPPdeltaARE	Adgre1	2.67465383	1.06E-20	1.68E-18
WT<TPPdeltaARE	Xdh	2.6563742	7.94E-34	4.47E-31
WT<TPPdeltaARE	Tlr1	2.62706591	4.94E-18	6.51E-16
WT<TPPdeltaARE	Grap	2.60693394	6.41E-10	4.21E-08
WT<TPPdeltaARE	Gbp4	2.59072027	3.91E-09	2.39E-07
WT<TPPdeltaARE	Slfn2	2.58286975	8.31E-23	1.60E-20
WT<TPPdeltaARE	Cd300lg	2.57630664	1.61E-15	1.69E-13
WT<TPPdeltaARE	Cxcl16	2.57265834	2.73E-13	2.43E-11
WT<TPPdeltaARE	Slamf7	2.57026434	1.16E-05	0.00040442
WT<TPPdeltaARE	Dpep2	2.53488977	1.00E-12	8.58E-11
WT<TPPdeltaARE	Pirb	2.52942642	1.08E-29	4.37E-27
WT<TPPdeltaARE	Ms4a2	2.51152481	4.22E-10	2.80E-08
WT<TPPdeltaARE	Nrp1	2.50470311	8.46E-26	2.17E-23
WT<TPPdeltaARE	Ifi30	2.48529716	1.40E-23	2.86E-21
WT<TPPdeltaARE	Cd300c2	2.47120721	1.71E-16	2.03E-14
WT<TPPdeltaARE	Rnd3	2.45512518	8.88E-28	2.74E-25
WT<TPPdeltaARE	Ccl6	2.44741395	4.10E-18	5.47E-16
WT<TPPdeltaARE	Grap2	2.44125732	6.14E-16	6.82E-14
WT<TPPdeltaARE	Gdpd5	2.4358041	1.08E-15	1.18E-13
WT<TPPdeltaARE	H2-DMb2	2.43462689	8.52E-26	2.17E-23
WT<TPPdeltaARE	Ppfia4	2.42304293	9.95E-11	7.03E-09
WT<TPPdeltaARE	Ccr9	2.40642673	4.53E-07	2.06E-05
WT<TPPdeltaARE	Cxcl10	2.40230473	9.75E-08	4.94E-06
WT<TPPdeltaARE	L1cam	2.37797624	3.46E-07	1.61E-05
WT<TPPdeltaARE	Hdc	2.37690042	3.22E-12	2.60E-10
WT<TPPdeltaARE	Elane	2.36247092	4.14E-13	3.62E-11
WT<TPPdeltaARE	Gpr68	2.35973346	1.89E-19	2.71E-17
WT<TPPdeltaARE	Anpep	2.33388879	4.87E-20	7.39E-18
WT<TPPdeltaARE	Clec5a	2.33328187	1.72E-11	1.32E-09
WT<TPPdeltaARE	Notch4	2.33209312	1.46E-14	1.42E-12
WT<TPPdeltaARE	Lilrb4a	2.32431882	1.11E-08	6.52E-07
WT<TPPdeltaARE	Cd7	2.30979067	1.67E-17	2.08E-15
WT<TPPdeltaARE	Cst3	2.30659789	1.48E-26	4.16E-24
WT<TPPdeltaARE	Mpzl3	2.29442192	6.21E-13	5.41E-11
WT<TPPdeltaARE	Ahnak	2.29349344	2.03E-13	1.86E-11
WT<TPPdeltaARE	Slc45a3	2.27097192	4.39E-07	2.00E-05
WT<TPPdeltaARE	C3	2.25375558	7.18E-07	3.13E-05
WT<TPPdeltaARE	Igsf6	2.24461766	1.20E-12	1.02E-10
WT<TPPdeltaARE	Nrxn2	2.23391516	5.92E-11	4.36E-09
WT<TPPdeltaARE	Cd6	2.23306439	4.44E-06	0.0001672
WT<TPPdeltaARE	Btla	2.21590262	5.78E-28	1.82E-25
WT<TPPdeltaARE	Gm21188	2.21342543	5.04E-08	2.68E-06
WT<TPPdeltaARE	Cyp27a1	2.19699244	4.29E-24	9.27E-22
WT<TPPdeltaARE	Lrrc3	2.16649247	2.55E-13	2.30E-11
WT<TPPdeltaARE	Itgae	2.1638907	2.88E-16	3.37E-14

WT<TPPdeltaARE	Hepacam2	2.15418629	0.00075791	0.01666228
WT<TPPdeltaARE	Gm26740	2.14246758	9.46E-12	7.42E-10
WT<TPPdeltaARE	Clec4a3	2.13629135	1.81E-05	0.00058994
WT<TPPdeltaARE	Ctsh	2.10515118	1.82E-24	4.09E-22
WT<TPPdeltaARE	Nucb2	2.10452326	2.48E-13	2.25E-11
WT<TPPdeltaARE	P3h2	2.08916281	9.22E-16	1.02E-13
WT<TPPdeltaARE	Gimap3	2.07874642	1.01E-07	5.09E-06
WT<TPPdeltaARE	Rasgrp3	2.07469036	0.00019159	0.00506245
WT<TPPdeltaARE	Cyp4f18	2.0596099	9.71E-05	0.00273476
WT<TPPdeltaARE	Slc46a3	2.04759866	1.94E-18	2.65E-16
WT<TPPdeltaARE	Trbc2	2.04517137	0.00014227	0.00386642
WT<TPPdeltaARE	Slc2a6	2.04275519	8.65E-06	0.0003084
WT<TPPdeltaARE	Gm37759	2.03415906	0.0001175	0.00324379
WT<TPPdeltaARE	Pik3r6	2.02984841	4.89E-08	2.62E-06
WT<TPPdeltaARE	Cx3cr1	2.02154316	5.68E-09	3.43E-07
WT<TPPdeltaARE	Cadm1	2.00742343	2.01E-07	9.87E-06
WT<TPPdeltaARE	Cttnbp2nl	1.99189729	7.09E-10	4.64E-08
WT<TPPdeltaARE	Atp1a3	1.99097044	1.27E-05	0.00043654
WT<TPPdeltaARE	Adcy4	1.98012463	5.15E-10	3.39E-08
WT<TPPdeltaARE	Ifi213	1.97256759	1.13E-15	1.22E-13
WT<TPPdeltaARE	Serpina3f	1.96621448	1.36E-07	6.74E-06
WT<TPPdeltaARE	Gcsam	1.9609811	5.83E-16	6.61E-14
WT<TPPdeltaARE	Fcgr3	1.95822298	1.93E-13	1.79E-11
WT<TPPdeltaARE	Mx1	1.95447193	2.98E-10	2.02E-08
WT<TPPdeltaARE	Cd300lb	1.94823913	2.29E-07	1.12E-05
WT<TPPdeltaARE	Ly6a2	1.94500655	7.14E-05	0.00208098
WT<TPPdeltaARE	Gria2	1.93226938	5.67E-05	0.00168666
WT<TPPdeltaARE	Clec12a	1.92719472	9.76E-12	7.62E-10
WT<TPPdeltaARE	Hbb-bt	1.9166981	4.09E-10	2.73E-08
WT<TPPdeltaARE	Gbp9	1.91646137	3.47E-09	2.15E-07
WT<TPPdeltaARE	Cybb	1.91372469	1.82E-05	0.00058994
WT<TPPdeltaARE	Lgals3	1.90246729	4.64E-11	3.48E-09
WT<TPPdeltaARE	Gm43109	1.90176559	1.57E-06	6.33E-05
WT<TPPdeltaARE	Vcam1	1.89806544	1.01E-05	0.00035534
WT<TPPdeltaARE	Gm21887	1.8925577	7.40E-08	3.85E-06
WT<TPPdeltaARE	Dbn1	1.88652395	6.14E-16	6.82E-14
WT<TPPdeltaARE	Wfdc18	1.87636043	4.27E-11	3.22E-09
WT<TPPdeltaARE	Ctnnd2	1.87508383	6.15E-07	2.71E-05
WT<TPPdeltaARE	Gimap4	1.85968093	2.42E-06	9.44E-05
WT<TPPdeltaARE	Filip1l	1.85948781	2.99E-10	2.02E-08
WT<TPPdeltaARE	Dse	1.8569335	4.44E-06	0.0001672
WT<TPPdeltaARE	Tubb2a	1.83904588	5.44E-12	4.31E-10
WT<TPPdeltaARE	A530010L16I	1.83419605	1.98E-08	1.13E-06
WT<TPPdeltaARE	St3gal5	1.82887033	7.89E-17	9.50E-15
WT<TPPdeltaARE	Fcgr2b	1.82387555	6.70E-17	8.13E-15

WT<TPPdeltaARE	Tmem51	1.79061371	7.47E-14	7.05E-12
WT<TPPdeltaARE	Zfp36	1.77503805	2.01E-13	1.85E-11
WT<TPPdeltaARE	Adap2	1.77467507	6.41E-06	0.00023436
WT<TPPdeltaARE	Gas7	1.76005517	2.43E-07	1.17E-05
WT<TPPdeltaARE	Shtn1	1.75987468	2.53E-10	1.73E-08
WT<TPPdeltaARE	Vmn2r97	1.7574194	0.00010491	0.00293221
WT<TPPdeltaARE	Ms4a3	1.75254526	5.93E-05	0.00175696
WT<TPPdeltaARE	Ccr7	1.75124442	1.86E-08	1.06E-06
WT<TPPdeltaARE	Cacna1s	1.74371815	0.00042534	0.01015851
WT<TPPdeltaARE	Fcrl1	1.74344637	4.43E-08	2.40E-06
WT<TPPdeltaARE	Tppp3	1.73725003	6.24E-11	4.51E-09
WT<TPPdeltaARE	Itga1	1.72324784	1.30E-08	7.54E-07
WT<TPPdeltaARE	Kynu	1.72158484	4.80E-09	2.92E-07
WT<TPPdeltaARE	Gbp8	1.71929598	1.96E-05	0.00063121
WT<TPPdeltaARE	Sult1a1	1.71563965	3.32E-08	1.84E-06
WT<TPPdeltaARE	H2-DMb1	1.7107831	1.58E-19	2.29E-17
WT<TPPdeltaARE	Apba1	1.70987615	7.69E-11	5.48E-09
WT<TPPdeltaARE	Plk2	1.70344891	0.00088654	0.01885892
WT<TPPdeltaARE	Ccr3	1.69538041	0.00150131	0.02936125
WT<TPPdeltaARE	Itgb7	1.68187945	4.53E-16	5.21E-14
WT<TPPdeltaARE	Mertk	1.66289039	0.00059919	0.0134736
WT<TPPdeltaARE	Ctsg	1.654661	0.00199146	0.03688771
WT<TPPdeltaARE	B430306N03	1.65244708	2.63E-13	2.36E-11
WT<TPPdeltaARE	Fcgr1	1.64856171	4.81E-09	2.92E-07
WT<TPPdeltaARE	Ms4a6d	1.64317584	4.30E-08	2.34E-06
WT<TPPdeltaARE	Clec4a1	1.6394403	0.00039947	0.00967267
WT<TPPdeltaARE	Chd7	1.63221398	6.89E-11	4.96E-09
WT<TPPdeltaARE	Mafb	1.6304411	0.00031991	0.00796652
WT<TPPdeltaARE	Evl	1.6185851	1.09E-10	7.63E-09
WT<TPPdeltaARE	Gapt	1.61139761	8.42E-13	7.29E-11
WT<TPPdeltaARE	Degs2	1.60930236	8.05E-11	5.72E-09
WT<TPPdeltaARE	Aldh3b1	1.60868884	0.00180851	0.03418178
WT<TPPdeltaARE	Papss2	1.60644153	0.00088966	0.01887279
WT<TPPdeltaARE	Tmem156	1.60586804	3.58E-09	2.21E-07
WT<TPPdeltaARE	C1qa	1.59989503	0.00032875	0.00816082
WT<TPPdeltaARE	Traf1	1.59977909	1.10E-10	7.65E-09
WT<TPPdeltaARE	Gm36161	1.59514916	3.27E-08	1.82E-06
WT<TPPdeltaARE	Art3	1.59469491	2.79E-05	0.00086941
WT<TPPdeltaARE	E330020D12	1.59382047	0.00021233	0.00555047
WT<TPPdeltaARE	Gm34680	1.59242851	0.00012423	0.00341158
WT<TPPdeltaARE	Clec4a2	1.58926368	8.44E-08	4.33E-06
WT<TPPdeltaARE	Il1rl1	1.58917388	6.16E-05	0.00181872
WT<TPPdeltaARE	Gm5431	1.58801883	1.04E-06	4.34E-05
WT<TPPdeltaARE	Mrvi1	1.58757469	2.09E-07	1.02E-05
WT<TPPdeltaARE	Clec2i	1.58509755	1.52E-10	1.05E-08

WT<TPPdeltaARE	Zmynd15	1.58010329	3.38E-06	0.00012853
WT<TPPdeltaARE	Prdm1	1.57554684	0.00281801	0.04925294
WT<TPPdeltaARE	Lifr	1.5706293	3.11E-05	0.00096283
WT<TPPdeltaARE	Dab2	1.56893959	9.27E-08	4.71E-06
WT<TPPdeltaARE	Crispld2	1.56831287	3.23E-07	1.51E-05
WT<TPPdeltaARE	Slc11a1	1.54485979	7.45E-06	0.00026817
WT<TPPdeltaARE	C1qb	1.54017075	8.81E-05	0.00251661
WT<TPPdeltaARE	Pla2g7	1.5327317	6.39E-07	2.81E-05
WT<TPPdeltaARE	P2rx7	1.52335888	4.62E-05	0.00139713
WT<TPPdeltaARE	Trim7	1.51820143	7.30E-10	4.75E-08
WT<TPPdeltaARE	Cd52	1.51731423	1.09E-07	5.45E-06
WT<TPPdeltaARE	Ctss	1.51142977	8.89E-05	0.00252856
WT<TPPdeltaARE	Gbp6	1.50841879	3.77E-05	0.00115031
WT<TPPdeltaARE	Il1rapl2	1.50787136	0.00280462	0.04917604
WT<TPPdeltaARE	Tfcp2l1	1.50409812	6.85E-07	2.99E-05
WT<TPPdeltaARE	Gpr18	1.50207821	4.36E-12	3.51E-10
WT<TPPdeltaARE	Ildr1	1.50154153	0.00189262	0.03533321
WT<TPPdeltaARE	Ms4a7	1.49404649	2.80E-06	0.00010751
WT<TPPdeltaARE	Hspa1b	1.48945262	0.00040452	0.00973502
WT<TPPdeltaARE	Naip6	1.47648913	8.41E-08	4.33E-06
WT<TPPdeltaARE	Cd28	1.47438749	0.00263848	0.04644297
WT<TPPdeltaARE	Plce1	1.46962665	1.74E-05	0.00057102
WT<TPPdeltaARE	Depp1	1.46713353	9.21E-07	3.88E-05
WT<TPPdeltaARE	Fabp4	1.45923876	0.00024336	0.00621726
WT<TPPdeltaARE	Ryr1	1.43953163	4.07E-08	2.23E-06
WT<TPPdeltaARE	Tnfrsf11a	1.43922606	7.95E-07	3.41E-05
WT<TPPdeltaARE	Cnr2	1.43611199	9.01E-10	5.85E-08
WT<TPPdeltaARE	C1qc	1.4349521	0.00100235	0.02101077
WT<TPPdeltaARE	Tpm4	1.43188287	1.30E-12	1.10E-10
WT<TPPdeltaARE	Rnf43	1.43122062	2.52E-05	0.00079265
WT<TPPdeltaARE	Cd300lf	1.42765525	0.0016339	0.03148549
WT<TPPdeltaARE	Chga	1.42254966	5.01E-07	2.26E-05
WT<TPPdeltaARE	Kcnk6	1.42086189	1.91E-06	7.56E-05
WT<TPPdeltaARE	Ltb4r1	1.4187345	2.77E-09	1.72E-07
WT<TPPdeltaARE	Cd68	1.41670637	2.00E-14	1.94E-12
WT<TPPdeltaARE	Pik3r5	1.41586666	1.27E-09	8.24E-08
WT<TPPdeltaARE	Adgre4	1.41393899	0.0002756	0.00694657
WT<TPPdeltaARE	Gm26586	1.41049287	5.54E-11	4.10E-09
WT<TPPdeltaARE	Slc40a1	1.40692067	0.00070268	0.01553478
WT<TPPdeltaARE	6430548M08	1.40418208	2.16E-05	0.00069112
WT<TPPdeltaARE	Lpxn	1.39201055	1.32E-10	9.16E-09
WT<TPPdeltaARE	Fgr	1.38662532	7.88E-09	4.70E-07
WT<TPPdeltaARE	Ddx4	1.38457393	2.95E-08	1.66E-06
WT<TPPdeltaARE	Phf11b	1.382845	1.22E-08	7.10E-07
WT<TPPdeltaARE	Il13ra1	1.38222718	0.00216924	0.03966796

WT<TPPdeltaARE	Fgl2	1.38166343	5.06E-12	4.03E-10
WT<TPPdeltaARE	Mt1	1.37705261	0.00177531	0.03359447
WT<TPPdeltaARE	Klf1	1.37657407	0.00142122	0.02805826
WT<TPPdeltaARE	AC174780.1	1.37387411	0.00105756	0.0219057
WT<TPPdeltaARE	Camk1d	1.37244288	2.54E-12	2.07E-10
WT<TPPdeltaARE	Tnni2	1.36842795	1.81E-08	1.04E-06
WT<TPPdeltaARE	Gpr137b	1.36621054	5.09E-07	2.27E-05
WT<TPPdeltaARE	Nrg2	1.36583142	6.89E-06	0.00025027
WT<TPPdeltaARE	Cd86	1.36566226	6.38E-08	3.36E-06
WT<TPPdeltaARE	Adgra2	1.36097428	1.49E-06	6.02E-05
WT<TPPdeltaARE	Rtn4rl1	1.35687554	0.00083617	0.01794356
WT<TPPdeltaARE	Gm42372	1.35475844	1.57E-07	7.80E-06
WT<TPPdeltaARE	Adgre5	1.354261	8.19E-07	3.50E-05
WT<TPPdeltaARE	Pqlc2	1.35192851	3.53E-07	1.64E-05
WT<TPPdeltaARE	Gm26716	1.34866775	2.69E-07	1.28E-05
WT<TPPdeltaARE	Mtus1	1.34855507	3.15E-05	0.00097072
WT<TPPdeltaARE	Lpl	1.34278644	0.00171624	0.03283132
WT<TPPdeltaARE	Il4i1	1.33835596	6.12E-06	0.00022425
WT<TPPdeltaARE	Cacna1d	1.33704219	1.81E-05	0.00058994
WT<TPPdeltaARE	Havcr2	1.33206138	3.10E-10	2.09E-08
WT<TPPdeltaARE	Dock4	1.32769819	1.14E-06	4.71E-05
WT<TPPdeltaARE	Cd38	1.32385077	0.00016153	0.00435243
WT<TPPdeltaARE	Tspan33	1.32014937	1.93E-09	1.23E-07
WT<TPPdeltaARE	Slc31a2	1.31867747	3.86E-09	2.37E-07
WT<TPPdeltaARE	Skap1	1.31785684	1.67E-05	0.00055107
WT<TPPdeltaARE	Per1	1.31325596	7.90E-05	0.00228101
WT<TPPdeltaARE	Il17rb	1.30633865	2.68E-07	1.28E-05
WT<TPPdeltaARE	Evi2a	1.30632729	1.85E-09	1.18E-07
WT<TPPdeltaARE	S100a4	1.30075983	9.11E-07	3.85E-05
WT<TPPdeltaARE	Ppt1	1.29544301	1.76E-12	1.47E-10
WT<TPPdeltaARE	Sdc3	1.28983538	9.50E-07	3.98E-05
WT<TPPdeltaARE	Sptbn5	1.28918615	1.38E-05	0.00046496
WT<TPPdeltaARE	Slfn5	1.28911088	4.90E-07	2.21E-05
WT<TPPdeltaARE	Anxa3	1.2890632	0.00172044	0.03287189
WT<TPPdeltaARE	Pkib	1.28861844	8.75E-07	3.73E-05
WT<TPPdeltaARE	Slamf6	1.28786709	2.32E-05	0.00073295
WT<TPPdeltaARE	Wfdc17	1.28521669	3.19E-07	1.50E-05
WT<TPPdeltaARE	Zc3h12d	1.28452922	0.00023187	0.00600167
WT<TPPdeltaARE	Prr5l	1.28409901	2.94E-08	1.66E-06
WT<TPPdeltaARE	Ppm1m	1.28333953	2.13E-10	1.46E-08
WT<TPPdeltaARE	Mapk13	1.28319422	9.34E-06	0.0003301
WT<TPPdeltaARE	AB124611	1.28193776	7.20E-07	3.13E-05
WT<TPPdeltaARE	Gimap7	1.27378679	8.77E-09	5.22E-07
WT<TPPdeltaARE	Uchl1	1.2657789	2.42E-07	1.17E-05
WT<TPPdeltaARE	Gm42928	1.26138402	1.35E-06	5.48E-05

WT<TPPdeltaARE	Tmem255b	1.25893719	6.75E-05	0.00197801
WT<TPPdeltaARE	Fau-ps2	1.25176216	0.00168791	0.03240747
WT<TPPdeltaARE	Card11	1.24370518	1.62E-08	9.30E-07
WT<TPPdeltaARE	Il10ra	1.24367677	4.77E-08	2.58E-06
WT<TPPdeltaARE	Sgk1	1.24367523	1.96E-09	1.25E-07
WT<TPPdeltaARE	Tyrobp	1.23374877	2.59E-07	1.24E-05
WT<TPPdeltaARE	Ighj4	1.2323171	0.00089078	0.01887279
WT<TPPdeltaARE	Siglecg	1.23044117	1.27E-05	0.00043556
WT<TPPdeltaARE	Psap	1.22957278	6.32E-12	4.98E-10
WT<TPPdeltaARE	Stbd1	1.22924282	5.66E-05	0.00168666
WT<TPPdeltaARE	Bank1	1.22917837	0.00281734	0.04925294
WT<TPPdeltaARE	Ccl3	1.22561711	5.54E-06	0.00020487
WT<TPPdeltaARE	Trib1	1.22225534	0.00076766	0.01680631
WT<TPPdeltaARE	Zbp1	1.22144634	5.07E-07	2.27E-05
WT<TPPdeltaARE	Sulf2	1.22016348	1.83E-06	7.28E-05
WT<TPPdeltaARE	Rgs11	1.21550535	2.21E-06	8.64E-05
WT<TPPdeltaARE	Gm35551	1.20452777	6.45E-05	0.00189347
WT<TPPdeltaARE	Gm49500	1.20231197	1.59E-06	6.36E-05
WT<TPPdeltaARE	Tmsb4x	1.19712369	3.80E-11	2.88E-09
WT<TPPdeltaARE	Crybg1	1.19533395	1.18E-05	0.00041077
WT<TPPdeltaARE	Trpm2	1.1949656	4.10E-08	2.24E-06
WT<TPPdeltaARE	Il31ra	1.19443103	0.00023804	0.00612097
WT<TPPdeltaARE	Stxbp6	1.19414754	1.24E-05	0.00042926
WT<TPPdeltaARE	Pparg	1.19286082	2.32E-07	1.13E-05
WT<TPPdeltaARE	Ighv1-77	1.18744538	2.99E-05	0.00093259
WT<TPPdeltaARE	Adora2b	1.18443601	5.78E-06	0.00021294
WT<TPPdeltaARE	Lsr	1.18125865	3.08E-07	1.45E-05
WT<TPPdeltaARE	Rab11fip4	1.17441722	2.63E-06	0.00010179
WT<TPPdeltaARE	Rgs10	1.16894862	8.81E-07	3.74E-05
WT<TPPdeltaARE	Zbtb7b	1.16330376	0.00020314	0.00533685
WT<TPPdeltaARE	Lgals1	1.15869611	2.53E-07	1.22E-05
WT<TPPdeltaARE	Ust	1.15733538	1.33E-06	5.42E-05
WT<TPPdeltaARE	Fgd2	1.15190626	6.83E-09	4.10E-07
WT<TPPdeltaARE	Nod2	1.15069258	2.10E-05	0.00067241
WT<TPPdeltaARE	Plvap	1.15068928	2.49E-05	0.00078475
WT<TPPdeltaARE	Rag2	1.14854447	1.35E-05	0.00045995
WT<TPPdeltaARE	Cnn3	1.14832968	1.28E-06	5.24E-05
WT<TPPdeltaARE	Ms4a6c	1.14737676	4.21E-05	0.00127852
WT<TPPdeltaARE	Lmo1	1.14138898	5.12E-06	0.00019079
WT<TPPdeltaARE	Gas6	1.14073363	0.00230174	0.04165602
WT<TPPdeltaARE	Nlrp1b	1.14056248	8.12E-05	0.00233701
WT<TPPdeltaARE	Unc93b1	1.13884304	2.01E-08	1.14E-06
WT<TPPdeltaARE	Chdh	1.1356365	9.81E-06	0.00034606
WT<TPPdeltaARE	Phf11c	1.1316346	1.40E-06	5.66E-05
WT<TPPdeltaARE	Ttll3	1.12703327	0.00027462	0.00694657

WT<TPPdeltaARE	Trib2	1.1249166	0.00016066	0.00433648
WT<TPPdeltaARE	Cdkn1a	1.12294162	8.56E-06	0.00030593
WT<TPPdeltaARE	Ncf1	1.11570142	1.23E-05	0.00042489
WT<TPPdeltaARE	Slc8b1	1.11501994	3.00E-07	1.42E-05
WT<TPPdeltaARE	Slfn5os	1.11357963	4.59E-06	0.00017242
WT<TPPdeltaARE	Irf7	1.1126402	6.85E-08	3.59E-06
WT<TPPdeltaARE	Neurl1a	1.10784224	0.00041937	0.01003463
WT<TPPdeltaARE	P2ry10	1.10733193	6.49E-07	2.85E-05
WT<TPPdeltaARE	Ifit3	1.10644916	1.90E-05	0.00061441
WT<TPPdeltaARE	Kif5a	1.10235823	1.37E-05	0.00046496
WT<TPPdeltaARE	Tlr12	1.08925567	9.19E-08	4.70E-06
WT<TPPdeltaARE	Fcer1g	1.08097972	1.69E-07	8.37E-06
WT<TPPdeltaARE	6330537M06	1.07916267	3.14E-05	0.00097072
WT<TPPdeltaARE	Tbc1d9	1.0780167	1.67E-05	0.00055034
WT<TPPdeltaARE	Celf4	1.07510783	0.00027511	0.00694657
WT<TPPdeltaARE	Ifi27l2a	1.07331895	1.40E-05	0.00047098
WT<TPPdeltaARE	Bcl2l14	1.07131204	2.85E-06	0.00010873
WT<TPPdeltaARE	Bfsp2	1.06883038	3.27E-05	0.00100603
WT<TPPdeltaARE	Iigp1	1.06803823	3.33E-08	1.84E-06
WT<TPPdeltaARE	Stk36	1.0623915	0.00016337	0.00438707
WT<TPPdeltaARE	Sh3bp4	1.05872835	7.66E-07	3.31E-05
WT<TPPdeltaARE	Gm35853	1.05640863	7.18E-06	0.00026029
WT<TPPdeltaARE	Il1r2	1.05175738	0.00023153	0.00600167
WT<TPPdeltaARE	Phf11d	1.04703197	5.61E-06	0.00020701
WT<TPPdeltaARE	Irf5	1.0421061	1.81E-07	8.89E-06
WT<TPPdeltaARE	Itgb2	1.04035687	3.51E-08	1.93E-06
WT<TPPdeltaARE	Lbh	1.03463587	2.74E-06	0.00010582
WT<TPPdeltaARE	Lsp1	1.03061557	5.03E-08	2.68E-06
WT<TPPdeltaARE	Samhd1	1.03034156	5.09E-07	2.27E-05
WT<TPPdeltaARE	F630028O10	1.02696845	1.36E-05	0.00046063
WT<TPPdeltaARE	Tmem255a	1.02669627	0.00113698	0.02333618
WT<TPPdeltaARE	Ccl5	1.02471373	0.00221202	0.04026336
WT<TPPdeltaARE	I830077J02R	1.02445481	0.00034109	0.00841408
WT<TPPdeltaARE	Tulp3	1.02139972	0.00052102	0.01202458
WT<TPPdeltaARE	Csf1r	1.01699936	0.00011058	0.0030688
WT<TPPdeltaARE	Igf1	1.0159161	0.00244804	0.04385051
WT<TPPdeltaARE	BE692007	1.01131087	0.00023569	0.00608052
WT<TPPdeltaARE	Btbd16	1.00809879	0.00070693	0.01560693
WT<TPPdeltaARE	Icosl	1.00128292	5.43E-07	2.42E-05
WT<TPPdeltaARE	Maf	1.00077131	4.90E-05	0.00147313
WT<TPPdeltaARE	C130026I21R	1.00054756	9.89E-06	0.00034802
WT<TPPdeltaARE	Tubb6	0.99610926	2.12E-06	8.33E-05
WT<TPPdeltaARE	Card10	0.99325248	0.00040158	0.00969391
WT<TPPdeltaARE	Lmna	0.98903882	0.00010024	0.00281151
WT<TPPdeltaARE	Ap3s1	0.97622945	0.00011977	0.00330044

WT<TPPdeltaARE	Bmf	0.9753649	0.00094768	0.01997949
WT<TPPdeltaARE	Themis2	0.97315774	4.08E-07	1.88E-05
WT<TPPdeltaARE	Dtx4	0.96885179	0.00048762	0.0113378
WT<TPPdeltaARE	Plekha1	0.9675659	9.25E-07	3.89E-05
WT<TPPdeltaARE	Raph1	0.96419132	3.44E-05	0.00105465
WT<TPPdeltaARE	Rgs8	0.95816667	0.000691	0.01531966
WT<TPPdeltaARE	Aoah	0.94967059	0.00213531	0.03918373
WT<TPPdeltaARE	Ccdc102a	0.94806415	3.65E-06	0.00013864
WT<TPPdeltaARE	Hdac9	0.94685105	0.00088917	0.01887279
WT<TPPdeltaARE	Gsn	0.94106908	0.00033427	0.00828467
WT<TPPdeltaARE	Map7d2	0.94062067	0.00124463	0.0251205
WT<TPPdeltaARE	Anxa2	0.93753524	7.34E-07	3.18E-05
WT<TPPdeltaARE	Kdm7a	0.93565358	6.46E-06	0.00023584
WT<TPPdeltaARE	Tstd3	0.9337495	0.00174731	0.03330457
WT<TPPdeltaARE	5330406M23	0.92643852	0.00117751	0.02404278
WT<TPPdeltaARE	Lgmn	0.92109242	1.35E-05	0.00045995
WT<TPPdeltaARE	Pde3b	0.91892413	0.00027587	0.00694657
WT<TPPdeltaARE	H2-Oa	0.91875934	6.73E-07	2.95E-05
WT<TPPdeltaARE	Gm6277	0.91623133	0.00251593	0.04486273
WT<TPPdeltaARE	Gm43915	0.91604943	0.00263991	0.04644297
WT<TPPdeltaARE	Otulinl	0.91340333	1.59E-06	6.36E-05
WT<TPPdeltaARE	Tmem50b	0.91329536	7.42E-06	0.00026773
WT<TPPdeltaARE	Slc22a23	0.91051351	0.00081436	0.0176086
WT<TPPdeltaARE	Prkcb	0.90987851	0.00017399	0.0046249
WT<TPPdeltaARE	Gm14455	0.90844179	0.00101395	0.02119739
WT<TPPdeltaARE	Cfp	0.90782858	1.27E-05	0.00043654
WT<TPPdeltaARE	Slc27a2	0.90703837	0.00095829	0.02016756
WT<TPPdeltaARE	Cd33	0.89853965	0.00052471	0.01207456
WT<TPPdeltaARE	Gpc1	0.89811959	5.62E-05	0.00167863
WT<TPPdeltaARE	5031439G07	0.89362649	5.49E-06	0.00020376
WT<TPPdeltaARE	Tsc22d3	0.88938022	0.00141733	0.02805826
WT<TPPdeltaARE	Tmsb10	0.88896556	1.72E-06	6.84E-05
WT<TPPdeltaARE	Spint1	0.88492968	0.00036945	0.00905698
WT<TPPdeltaARE	Stox2	0.8815261	0.00155077	0.03006739
WT<TPPdeltaARE	9530052E02I	0.88111087	0.00110292	0.02272595
WT<TPPdeltaARE	Dok3	0.88106355	0.00121227	0.02462502
WT<TPPdeltaARE	Rtl8b	0.88060039	4.72E-05	0.00142422
WT<TPPdeltaARE	Rgmb	0.87780473	0.0005341	0.01223693
WT<TPPdeltaARE	Csf2ra	0.87483734	2.18E-05	0.00069479
WT<TPPdeltaARE	2310040G24	0.8728714	0.00100404	0.02101816
WT<TPPdeltaARE	Gramd3	0.87020094	9.55E-05	0.00270192
WT<TPPdeltaARE	Pik3cb	0.86857723	2.61E-05	0.00081916
WT<TPPdeltaARE	Gm10645	0.86371022	0.00175685	0.03336538
WT<TPPdeltaARE	Rubcnl	0.86083243	7.21E-05	0.0020917
WT<TPPdeltaARE	Lrp1	0.86080545	0.00149201	0.02921566

WT<TPPdeltaARE	Cass4	0.85450009	0.00143482	0.02827131
WT<TPPdeltaARE	Sh3tc1	0.85074775	0.00018616	0.00493177
WT<TPPdeltaARE	Gm27252	0.84803578	0.00189409	0.03533321
WT<TPPdeltaARE	Gm11767	0.83812574	0.00153209	0.02981515
WT<TPPdeltaARE	Btg2	0.83659884	0.00021337	0.00556845
WT<TPPdeltaARE	Gm4951	0.83491405	0.00015995	0.00432473
WT<TPPdeltaARE	Ankmy1	0.83411667	0.00038154	0.00932434
WT<TPPdeltaARE	Rnase6	0.83197527	4.74E-06	0.00017762
WT<TPPdeltaARE	Met	0.83031803	0.00112944	0.02321167
WT<TPPdeltaARE	Klrb1f	0.82789955	0.00107956	0.02230289
WT<TPPdeltaARE	Bcl6	0.82724633	0.00138216	0.02750882
WT<TPPdeltaARE	Ccnd1	0.82657377	0.00117023	0.02392528
WT<TPPdeltaARE	Gm26597	0.82269571	0.00101665	0.02122569
WT<TPPdeltaARE	Crip1	0.81541059	9.12E-06	0.00032313
WT<TPPdeltaARE	4930528J11F	0.81018415	0.00246337	0.04407493
WT<TPPdeltaARE	Myadm	0.80893237	4.50E-05	0.00136473
WT<TPPdeltaARE	Abcd2	0.80873197	0.00139011	0.02763215
WT<TPPdeltaARE	Ifngr1	0.80695986	1.82E-05	0.00058994
WT<TPPdeltaARE	Gm32401	0.80584147	0.0013081	0.026167
WT<TPPdeltaARE	BC147527	0.8007181	6.08E-05	0.00179853
WT<TPPdeltaARE	Fam129a	0.79666205	2.08E-05	0.00066844
WT<TPPdeltaARE	Psd	0.79617093	0.0022047	0.04017658
WT<TPPdeltaARE	Dhx40	0.7937265	0.00083523	0.01794356
WT<TPPdeltaARE	Rnf144b	0.79218404	0.00086391	0.01842726
WT<TPPdeltaARE	Ctsz	0.79111602	2.39E-05	0.00075381
WT<TPPdeltaARE	Gm8093	0.79109471	0.00251108	0.04482801
WT<TPPdeltaARE	BC051537	0.78341776	0.00013736	0.003746
WT<TPPdeltaARE	Naga	0.78327737	1.51E-05	0.00050184
WT<TPPdeltaARE	Klf6	0.78022238	0.00017276	0.00460781
WT<TPPdeltaARE	Aph1b	0.77787217	0.00054471	0.01240785
WT<TPPdeltaARE	Mospd2	0.77742044	0.00010061	0.00281699
WT<TPPdeltaARE	Clec7a	0.7676966	0.00048258	0.01125286
WT<TPPdeltaARE	Rtl8c	0.76439698	0.00189392	0.03533321
WT<TPPdeltaARE	Gm9844	0.76280293	0.00048149	0.01124404
WT<TPPdeltaARE	Gm2a	0.7602714	1.58E-05	0.00052463
WT<TPPdeltaARE	Lck	0.75716958	8.75E-05	0.00250287
WT<TPPdeltaARE	Slc9a9	0.7547065	0.00025286	0.00643917
WT<TPPdeltaARE	Hk2	0.75432177	0.00029395	0.00736651
WT<TPPdeltaARE	Tgfbi	0.74903641	0.00053784	0.01229598
WT<TPPdeltaARE	Scpep1os	0.74609529	0.00067385	0.01504509
WT<TPPdeltaARE	Anxa6	0.74510664	7.70E-05	0.0022258
WT<TPPdeltaARE	Rab43	0.7446853	0.00046703	0.01095505
WT<TPPdeltaARE	A530040E14	0.74303073	0.0012038	0.02448926
WT<TPPdeltaARE	Cpeb4	0.7419444	0.00041952	0.01003463
WT<TPPdeltaARE	Plekhm3	0.73855715	0.00074675	0.01644002

WT<TPPdeltaARE	Ece1	0.73852647	0.00019173	0.00506245
WT<TPPdeltaARE	Tifa	0.73783573	7.04E-05	0.00205628
WT<TPPdeltaARE	Fcgrt	0.7376508	0.0004097	0.0098297
WT<TPPdeltaARE	Dusp6	0.73687673	0.00081601	0.01762019
WT<TPPdeltaARE	Rhob	0.73676582	0.00046418	0.01092622
WT<TPPdeltaARE	Isg15	0.73658083	0.0008033	0.01744141
WT<TPPdeltaARE	Lrrk2	0.73511606	0.00147662	0.0289863
WT<TPPdeltaARE	Rasa4	0.73494379	0.00195829	0.03635871
WT<TPPdeltaARE	Btg1	0.7319674	0.00013389	0.00365761
WT<TPPdeltaARE	Cyth4	0.73082288	0.00056925	0.01286503
WT<TPPdeltaARE	Nfil3	0.72993847	0.00072632	0.01601252
WT<TPPdeltaARE	Gpr65	0.72957621	8.86E-05	0.00252541
WT<TPPdeltaARE	Slc35d3	0.72531764	0.00038618	0.0094231
WT<TPPdeltaARE	Il6ra	0.72411453	0.00052627	0.01207696
WT<TPPdeltaARE	Gm4673	0.72265289	0.00085592	0.01828156
WT<TPPdeltaARE	Gm16175	0.72039395	0.00215585	0.03946877
WT<TPPdeltaARE	S100a6	0.71486373	0.00076675	0.01680631
WT<TPPdeltaARE	Hpse	0.71276639	0.00213382	0.03918373
WT<TPPdeltaARE	Gpt2	0.71096874	0.00083781	0.01794356
WT<TPPdeltaARE	Ier5	0.70900627	0.002533	0.04511593
WT<TPPdeltaARE	Igtp	0.70889296	0.0005563	0.0126172
WT<TPPdeltaARE	Vsir	0.7071251	0.00107448	0.022227
WT<TPPdeltaARE	Cd2ap	0.70277986	0.00068173	0.01519957
WT<TPPdeltaARE	Pfkp	0.69361055	0.0002417	0.00619707
WT<TPPdeltaARE	H2-Ob	0.69198617	0.00042645	0.01016952
WT<TPPdeltaARE	D1Ertd622e	0.69135911	0.00201085	0.03720299
WT<TPPdeltaARE	Sorl1	0.68603542	0.00077588	0.01693937
WT<TPPdeltaARE	Slc12a9	0.68295738	0.00024231	0.00620046
WT<TPPdeltaARE	H2-DMa	0.68018336	0.0001996	0.00525725
WT<TPPdeltaARE	Rara	0.67766472	0.0005672	0.01284602
WT<TPPdeltaARE	Scpep1	0.67538232	0.00082224	0.01770629
WT<TPPdeltaARE	Gbp2	0.65950807	0.00032287	0.00802735
WT<TPPdeltaARE	Rassf4	0.65799628	0.00055303	0.01256104
WT<TPPdeltaARE	Ifngr2	0.65694144	0.00045948	0.01087514
WT<TPPdeltaARE	Syngr2	0.65460037	0.00061566	0.01382421
WT<TPPdeltaARE	Adarb1	0.65131539	0.00219872	0.04011395
WT<TPPdeltaARE	Plin2	0.64910247	0.00040116	0.00969391
WT<TPPdeltaARE	Gm47566	0.64444786	0.00081749	0.01762793
WT<TPPdeltaARE	Stat1	0.63698826	0.00056968	0.01286503
WT<TPPdeltaARE	Gm12250	0.63643451	0.00173961	0.03319796
WT<TPPdeltaARE	Tgtp1	0.6356096	0.00031872	0.0079492
WT<TPPdeltaARE	Gbp5	0.63489676	0.00260063	0.04595718
WT<TPPdeltaARE	Nckipsd	0.63400903	0.00188677	0.035322
WT<TPPdeltaARE	H2-K2	0.62785206	0.00238388	0.04299433
WT<TPPdeltaARE	Sla	0.623122	0.00101979	0.02126321

WT<TPPdeltaARE	Sat1	0.61747983	0.00104831	0.02174276
WT<TPPdeltaARE	Tmem202	0.61652757	0.00224444	0.0406657
WT<TPPdeltaARE	Stx7	0.6143521	0.0008375	0.01794356
WT<TPPdeltaARE	Taldo1	0.60242421	0.001286	0.02575749
WT<TPPdeltaARE	Slc25a38	0.60159095	0.00186291	0.03491688
WT<TPPdeltaARE	Nptn	0.6001919	0.00118101	0.02408308
WT<TPPdeltaARE	Bloc1s2	0.59976718	0.00120403	0.02448926
WT<TPPdeltaARE	Unc119b	0.59758117	0.00134105	0.02675817
WT<TPPdeltaARE	Ahcyl2	0.57820824	0.00141391	0.02803462
WT<TPPdeltaARE	Kbtbd11	0.57724771	0.00281838	0.04925294
WT<TPPdeltaARE	Calm1	0.57232453	0.00282631	0.04933674
WT<TPPdeltaARE	Rab32	0.54535625	0.00222665	0.04046857
WT>TPPdeltaARE	Tie1	-0.5746144	0.0023567	0.04255289
WT>TPPdeltaARE	Rab11fip5	-0.5925504	0.00184972	0.03471094
WT>TPPdeltaARE	Pard3b	-0.6160776	0.00182157	0.03438364
WT>TPPdeltaARE	Tceanc	-0.6162544	0.00240054	0.0431467
WT>TPPdeltaARE	Lamc1	-0.6226192	0.00268334	0.04715442
WT>TPPdeltaARE	Clvs1	-0.6467082	0.00239845	0.0431467
WT>TPPdeltaARE	Esam	-0.6470029	0.00203293	0.03752357
WT>TPPdeltaARE	Tbxa2r	-0.6476769	0.00094809	0.01997949
WT>TPPdeltaARE	Itm2a	-0.6526541	0.00148218	0.02905927
WT>TPPdeltaARE	Mfsd4b4	-0.6528079	0.00285394	0.04965419
WT>TPPdeltaARE	Dlg3	-0.6720478	0.00111542	0.02295342
WT>TPPdeltaARE	Gab1	-0.6749589	0.00198107	0.03673833
WT>TPPdeltaARE	Zfp947	-0.6893169	0.00241483	0.0433541
WT>TPPdeltaARE	Ppp1r3d	-0.6908015	0.00124901	0.02517667
WT>TPPdeltaARE	C1rl	-0.6921728	0.00162088	0.03134957
WT>TPPdeltaARE	D130058E05	-0.6928672	0.00230743	0.04171108
WT>TPPdeltaARE	Rab17	-0.7002826	0.00175597	0.03336538
WT>TPPdeltaARE	Alg6	-0.7121699	0.00066843	0.01496655
WT>TPPdeltaARE	Zfp108	-0.7186904	0.00284573	0.04959311
WT>TPPdeltaARE	Zgrf1	-0.7256455	0.00144902	0.02847997
WT>TPPdeltaARE	Kcnd1	-0.7322421	0.00284729	0.04959311
WT>TPPdeltaARE	Gm49760	-0.7348462	0.00255641	0.04537912
WT>TPPdeltaARE	Aif1l	-0.7386591	0.00142223	0.02805826
WT>TPPdeltaARE	Ralgps1	-0.7413698	0.0010468	0.02173999
WT>TPPdeltaARE	Gp9	-0.7553092	0.00182995	0.0344629
WT>TPPdeltaARE	E130311K13I	-0.7671372	0.00151629	0.02958079
WT>TPPdeltaARE	BC030343	-0.7700994	0.00254189	0.04522322
WT>TPPdeltaARE	Zfp93	-0.7720616	0.0004308	0.01025794
WT>TPPdeltaARE	Zc3h6	-0.7762506	0.00020857	0.00546139
WT>TPPdeltaARE	Gng11	-0.7815945	0.00022625	0.00588502
WT>TPPdeltaARE	Ptpdc1	-0.7837589	0.00077998	0.01700528
WT>TPPdeltaARE	S1pr3	-0.7891101	0.0021267	0.0391168
WT>TPPdeltaARE	Itga2b	-0.8002613	0.00040607	0.00975736

WT>TPPdeltaARE	Slfn9	-0.8034181	0.0002771	0.00696638
WT>TPPdeltaARE	Spef1	-0.8064771	0.00136988	0.0272988
WT>TPPdeltaARE	Fam171a2	-0.807184	0.00027382	0.00693918
WT>TPPdeltaARE	Gp5	-0.854193	0.00029347	0.0073661
WT>TPPdeltaARE	Fbln1	-0.8636295	0.00176816	0.03353971
WT>TPPdeltaARE	Prx	-0.8675486	6.26E-05	0.0018433
WT>TPPdeltaARE	Dock1	-0.8780898	4.73E-05	0.00142422
WT>TPPdeltaARE	Gm43858	-0.8827346	0.00150758	0.02944722
WT>TPPdeltaARE	Fbxo16	-0.8901166	0.00251114	0.04482801
WT>TPPdeltaARE	Il1r1	-0.9005055	6.37E-05	0.00187402
WT>TPPdeltaARE	Gm45667	-0.9011262	0.0017527	0.03336538
WT>TPPdeltaARE	Pcdh7	-0.9027408	0.00051408	0.0119167
WT>TPPdeltaARE	Rgs7bp	-0.9149024	0.00038826	0.00944554
WT>TPPdeltaARE	4930432K21I	-0.9221725	0.0003883	0.00944554
WT>TPPdeltaARE	Klhl4	-0.9269746	0.00014153	0.00385304
WT>TPPdeltaARE	Shroom4	-0.9270911	0.00010644	0.0029695
WT>TPPdeltaARE	Camk2b	-0.9308422	0.00017382	0.0046249
WT>TPPdeltaARE	Cyb561	-0.9313677	0.00015165	0.00410721
WT>TPPdeltaARE	1700123M08	-0.9335251	0.00123931	0.02505431
WT>TPPdeltaARE	Ajuba	-0.9341388	0.00254828	0.04528575
WT>TPPdeltaARE	Lrig3	-0.9372524	0.00051597	0.01194316
WT>TPPdeltaARE	Snx7	-0.9384209	1.63E-05	0.0005392
WT>TPPdeltaARE	Trp53cor1	-0.9421215	0.00035235	0.00865111
WT>TPPdeltaARE	Tcaf1	-0.9436243	1.46E-05	0.00048792
WT>TPPdeltaARE	Obsl1	-0.9523485	0.00017202	0.00459576
WT>TPPdeltaARE	Asb1	-0.9547295	0.00014514	0.00393784
WT>TPPdeltaARE	Rab27b	-0.9793523	2.17E-06	8.51E-05
WT>TPPdeltaARE	Ppic	-0.9918549	2.74E-07	1.30E-05
WT>TPPdeltaARE	Exoc3l2	-1.0021615	0.00068625	0.01525712
WT>TPPdeltaARE	Slc16a12	-1.0059831	0.00012176	0.00334947
WT>TPPdeltaARE	Sox6	-1.0071044	7.82E-07	3.37E-05
WT>TPPdeltaARE	Tmem74	-1.0222763	0.00037973	0.00929453
WT>TPPdeltaARE	Dmc1	-1.0273676	0.0005509	0.01253079
WT>TPPdeltaARE	Hist1h3d	-1.0421363	0.00183788	0.03457096
WT>TPPdeltaARE	Gm32742	-1.0540641	0.00020537	0.00538653
WT>TPPdeltaARE	5730414N17	-1.0693701	0.00141067	0.02800559
WT>TPPdeltaARE	Fzd8	-1.0827831	7.30E-05	0.0021142
WT>TPPdeltaARE	Mmp14	-1.0838672	0.00026963	0.00685513
WT>TPPdeltaARE	Gp1ba	-1.0883331	0.00044416	0.01055997
WT>TPPdeltaARE	Gfi1b	-1.0957108	3.53E-05	0.00107868
WT>TPPdeltaARE	Gm41556	-1.1120547	0.00077522	0.01693937
WT>TPPdeltaARE	Tgfbr3	-1.1217883	3.93E-07	1.81E-05
WT>TPPdeltaARE	Pdgfrb	-1.1324124	2.67E-05	0.00083366
WT>TPPdeltaARE	Mpdz	-1.135135	0.00123976	0.02505431
WT>TPPdeltaARE	Igsf10	-1.1366554	1.38E-05	0.00046553

WT>TPPdeltaARE	Ifi203-ps	-1.1428123	0.00076765	0.01680631
WT>TPPdeltaARE	4732491K20I	-1.1750452	0.00110208	0.02272595
WT>TPPdeltaARE	Pcdhgc4	-1.2037178	0.00047741	0.0111819
WT>TPPdeltaARE	Nhsl1	-1.2241824	0.00033796	0.00834988
WT>TPPdeltaARE	Phldb2	-1.2258541	1.18E-06	4.87E-05
WT>TPPdeltaARE	Vldlr	-1.2638367	7.16E-05	0.00208215
WT>TPPdeltaARE	Plscr2	-1.2776626	9.22E-05	0.00261894
WT>TPPdeltaARE	Tnfsf4	-1.2948881	3.90E-05	0.00118751
WT>TPPdeltaARE	Gm16548	-1.3164067	0.00127773	0.02562451
WT>TPPdeltaARE	Tek	-1.3447235	2.17E-10	1.48E-08
WT>TPPdeltaARE	Zkscan4	-1.3910627	2.07E-06	8.16E-05
WT>TPPdeltaARE	Stag3	-1.4261725	0.0012548	0.02522889
WT>TPPdeltaARE	4932441J04F	-1.5445751	4.91E-06	0.00018339
WT>TPPdeltaARE	Arhgef28	-1.5449543	2.39E-07	1.16E-05
WT>TPPdeltaARE	Mid2	-1.5576353	1.58E-05	0.00052428
WT>TPPdeltaARE	Gm42031	-1.6722517	0.0004622	0.01090664
WT>TPPdeltaARE	Hs6st2	-1.6730688	0.00010777	0.00300139
WT>TPPdeltaARE	Selp	-1.7276402	0.00045251	0.01072606
WT>TPPdeltaARE	Gm49980	-1.9720241	2.23E-05	0.00070851
WT>TPPdeltaARE	Podxl	-1.9883942	2.14E-09	1.35E-07
WT>TPPdeltaARE	AC158975.2	-2.1821211	1.10E-05	0.00038469
WT>TPPdeltaARE	Fhdc1	-2.8934411	1.34E-05	0.00045831
WT>TPPdeltaARE	Igkv6-17	-10.753279	7.33E-08	3.82E-06
WT>TPPdeltaARE	Igkv5-39	-13.055596	1.70E-11	1.32E-09
