# Supplementary material for: Tristetraprolin overexpression drives hematopoietic changes in young and middle-aged mice generating dominant mitigating effects on induced inflammation in murine models
Source: GeroScience. 2023 Aug 3;46(1):1271–84. doi: 10.1007/s11357-023-00879-2 (PMC10828162; doi:10.1007/s11357-023-00879-2)
Supplement: Supplementary file 3 — Supplementary file3 (PDF 531 KB) [file 11357_2023_879_MOESM3_ESM.pdf]

Supplemental Table 2: Overlap of HSC aging transcription changes and TTP overxpression in HSCs

Name	logFC aging signature	logFC young TTP vs WT HSC	logFC mid-TTP vs mid-WT HSC
Kcnb2	4.72	-	1.20
Ntf3	4.67	-	-
Mab21l2	4.47	-	1.21
Eya4	4.25	-	1.94
Sbspon	4.19	1.30	1.04
Osmr	4.05	-1.48	-1.14
Cntn1	4.04	-	-
Gabra4	4.03	-	-
Gipc2	3.77	-	-0.93
Clu	3.54	-	-1.50
Rbpjl	3.45	-	-1.32
Zg16	3.15	-	-1.25
Selp	3.04	-0.91	-0.64
Tdrd9	2.97	-	-
Efemp1	2.97	-	-
Ptprz1	2.92	-	-
Mt2	2.9	1.43	-
Lrrn1	2.89	-	-1.13
Clca3a1	2.85	-1.56	-1.76
Ripk4	2.84	-	-
Tc2n	2.79	-	-
Plscr2	2.79	-1.01	-
Matn4	2.78	-	-1.63
Fap	2.7	-	-
Muc1	2.69	-	-2.28
Ramp2	2.58	-	-
Tmem215	2.56	-	-2.91
Trpc1	2.53	-	1.33
Chrna7	2.52	-	-
C4a	2.52	-	-
Rgn	2.5	-	-
Rorb	2.49	-	0.90
Tm4sf1	2.46	-	-
H2-T3	2.46	-	-
Gm5833	2.44	-0.91	-1.04
Wwtr1	2.43	-	-
Zswim5	2.41	-	-
Gpr183	2.41	0.63	-
Ntn4	2.35	0.59	0.59

C130026I21Ril	2.32	-	0.65
Agtr1a	2.32	-	-
C4b	2.28	-	-2.68
A530040E14Ri	2.28	-	0.63
Sspn	2.28	-	-
Nupr1	2.27	-	-
Vopp1	2.27	-	-
Gstm2	2.26	-	-0.70
Bmpr1a	2.21	-0.70	-
Kdf1	2.19	-	-
Tmem47	2.18	-	-
Gda	2.17	-0.44	-
Bmp4	2.16	-	-
Klf6	2.16	-	-
Sult1a1	2.15	0.61	-
Tmem56	2.14	-	-
Mt1	2.13	0.87	-
Lhfp	2.07	-	-
Aspa	2.03	-	0.53
Cyb561	2.03	-	-
Neo1	2.02	0.46	-
Adgrg2	2.01	-	-
Synm	1.99	-	-
Pclo	1.99	-1.20	-0.58
Gm10419	1.98	-	-
Epha2	1.98	-	-
Klrb1c	1.98	0.78	-
Gpx8	1.96	-	-0.79
Hes1	1.95	-	-
Jam2	1.94	-	-
Gm14964	1.94	1.17	0.95
Ptgfrn	1.92	-	-
Maf	1.91	-	0.59
Dpy19l2	1.9	-	-
Perp	1.87	-	-
Dsg2	1.87	-0.78	-
Lpl	1.85	-0.62	-
Pgr	1.84	-	-
Ddr1	1.83	-	-
Clec1a	1.8	-	0.52
Dnm3	1.8	-	-
B3galt1	1.79	-	-
Uhrf1bp1l	1.78	-	-

Meis2	1.78	-0.79	-
Dsc2	1.76	-0.70	-
Ptprk	1.76	-0.96	-
Scd1	1.75	-	-
Ehd3	1.72	-	-
Aldh1a1	1.71	-	-
Bhlhe40	1.71	-	-
Ncam1	1.71	-0.63	-0.79
Cavin2	1.69	-	-
Cd53	1.69	-	-
Sema6d	1.68	-1.28	-
Clec7a	1.67	-	-
Phf11d	1.66	-	-
Abat	1.66	-	-
Runx1t1	1.66	-	-
Amotl2	1.66	-	-
Fam169a	1.65	-	-
Tent5c	1.65	-	-
Exoc3l4	1.63	-	-
Cd200r4	1.62	-	0.82
Acpp	1.62	-	-
Pfn2	1.61	-	-
Kdr	1.61	-1.25	-
Alcam	1.59	-	-
Asb4	1.58	-0.58	-
Cd38	1.57	-	-
Klhl4	1.57	-	-
Rab2a	1.57	-	-
Col18a1	1.56	-	-1.31
Il1rapl2	1.55	-	0.43
Enpp5	1.55	-	-
Ror2	1.55	-	-
Gadd45g	1.54	-	-
Timp3	1.54	-0.84	-
Sfrp1	1.53	-	-
Zfp36	1.52	0.98	1.01
Clec9a	1.51	-	0.61
Nrg4	1.51	-	-
Calml4	1.51	-0.67	-
Cpne8	1.5	-	-
Plk2	1.49	-	0.71
Cysltr2	1.47	-	-
Plcl1	1.47	-	-

Fhdc1	1.47	-0.75	-
Clec1b	1.44	-	-
Abca4	1.44	-0.82	-1.37
Sdcbp	1.42	-	-
Vldlr	1.42	-1.44	-2.67
Pcdhb16	1.41	-	-
Cpt1c	1.41	-	-
Pde9a	1.4	-	-0.77
Hpgds	1.4	-	0.68
Abcb1a	1.39	-	0.62
Nudt10	1.39	-	-
Gkn3	1.39	0.94	-
Rab40b	1.38	-	-
Vwf	1.37	-	-
\730089K16Ri	1.37	-	-
Sgk1	1.37	-	-
Ghr	1.36	-	-
Slc6a15	1.36	-	-
AA986860	1.36	-	-
S1pr1	1.35	-0.43	-0.52
Mmp14	1.35	-0.75	-0.67
Klrb1b	1.34	-	-
Pkp2	1.34	-	-
Id2	1.33	0.91	-
Zfp334	1.33	-0.67	-
Gem	1.32	0.67	0.94
Ocln	1.31	-	-
Slc7a7	1.31	-	-
Enkur	1.31	-1.01	-
Dock9	1.28	-	-
Plek	1.27	-	1.08
Ptger4	1.27	-	-
Ldhd	1.26	-	-
Tmem254b	1.26	-	-
Slco2a1	1.26	-0.89	-
Thbd	1.26	-1.35	-
Itgb3	1.25	-	-1.14
Hoxb6	1.25	-	-
S100a6	1.23	-	-
Lgals3bp	1.23	-	-
Dhrs3	1.22	-0.69	-
Rhoj	1.22	-0.86	-0.51
Pls3	1.2	-	-

Rdh10	1.2	0.46	0.86
Fhl1	1.19	-	-
Oxr1	1.19	-	-
Kiss1r	1.19	-	-
Rab34	1.18	-	0.65
Rorc	1.18	-	-
Myo1e	1.15	-	-0.91
Cyyr1	1.15	-	-
Stxbp4	1.14	-	-
Dennd5b	1.13	-	-
Ampd3	1.13	-	-
Stom	1.12	-	-
Lsr	1.12	-	-
Ccl6	1.11	-	-
Cd74	1.11	-	-
Cyp26b1	1.11	0.91	1.25
Plscr1	1.1	-	-
Casp12	1.1	-	-
Mllt3	1.1	-	-
Pbx3	1.09	-	-
Tacstd2	1.09	-	-
Evc2	1.09	-	-
Cd55	1.08	-	-
Cpeb2	1.08	-	-
Gabarapl1	1.08	-	-
Bcl6	1.07	-	-
Pdgfd	1.07	-	-
Egr1	1.06	-	-
Cytip	1.05	-	-
Serinc3	1.05	-	-
Exoc3l2	1.05	-0.67	-
Tox	1.04	-	0.62
Ndrg1	1.04	-	-
Cd9	1.04	-	-
Itga6	1.04	-	-
Efna1	1.03	-	-
Vmp1	1.03	-	-
Dstn	1.03	-	-
Erp27	1.03	-	-
Rnf150	1.03	-	-
Trim47	1.01	-	-
Gm3470	1.01	-	-
Tgm2	1	-	-

Rab27b	1	-	-
Ctsc	0.99	-	-
Pip5k1b	0.99	-	-
Chst2	0.98	-	0.96
Arhgap29	0.98	-	-
Serpinb8	0.98	-	-
Nabp1	0.98	-	-
Prcp	0.97	-	-
Pros1	0.97	-	-
Prtn3	0.97	-	-
Nckap1	0.97	-	-
Phactr1	0.97	-	-
Tnfsf4	0.97	-	-
Wdfy1	0.97	-	-
Tbc1d8	0.96	-	-
Car5b	0.96	-	-
Cd302	0.96	-	-
Slc14a1	0.95	-	-
Slc44a1	0.95	-	-
Arhgef28	0.95	-0.62	-
Gpx3	0.94	-	0.62
Lamp2	0.94	-	-
Acsl4	0.94	-	-
Cxcl16	0.94	-	-
Pbx1	0.94	-0.43	-0.46
Dhx40	0.93	-	-0.58
Sema7a	0.93	-	-
Rbpms2	0.93	-	-
Slamf1	0.92	-	0.41
Gm13212	0.92	-	-
Art4	0.91	-	-
Col16a1	0.91	-	-
Selenom	0.91	-	-
Tnfaip2	0.91	-	-
Fosl2	0.91	1.09	-
Jun	0.91	-0.64	-
Serpinb6a	0.89	-	-
Zfp979	0.89	-	-
Fyb	0.88	-	-
Nrgn	0.88	-	-
Evc	0.87	-	-
Kcnip3	0.87	-	-
Dnajb4	0.87	-	-

Zfyve9	0.87	-	-
Mef2c	0.86	-	-
Hoxb5	0.86	-	-
Tnip3	0.85	-	0.45
Abcb1b	0.85	-	-
Ctsw	0.85	-	-
Sat1	0.85	-	-
Epb41l5	0.85	-0.60	-
Clec14a	0.84	-	0.61
Lpar6	0.84	-	-
Prnp	0.84	-	-
Rasgef1b	0.83	-	-0.85
Chac2	0.83	-	-
Exoc6b	0.82	-	-
Gpr146	0.82	-	-
Pla2g4a	0.81	-	-
Ezh1	0.81	-	-
Gnai3	0.81	-	-
Elovl5	0.8	-	-
Ndn	0.79	-	-
Tbxa2r	0.79	-	-
Npdc1	0.78	-	-
Galnt6	0.78	-	-
Myo6	0.77	-	-
Plscr4	0.76	-	-
Anxa5	0.76	-	-
Arid5b	0.76	-	-
Enpp4	0.76	-	-
Tnfsf10	0.75	-	-
Il1r1	0.75	-0.78	-0.48
Hk2	0.74	-	-
Nt5c3	0.71	-	-
Stat3	0.71	-	-
Ppp1r16b	0.7	-	-
Clca1	0.7	-	-
F2rl3	0.7	-	-
Il6st	0.7	-	-
Gstm1	0.69	-	-
Ctse	0.69	-	-
Coq8a	0.68	-	-
Ifi47	0.68	-	-
Ptger3	0.68	-	-
Gstm7	0.67	-	-

Camkk1	0.67	-	-
Cdcp1	0.67	-	-
Pign	0.66	-	-
Mmrn1	0.65	-	-
Cldn12	0.64	-	-
Muc13	0.63	-	-0.64
Procr	0.63	-	0.42
Tmem176a	0.62	-	-
Slco4a1	0.62	-	-
Ctso	0.61	-	-
Rab11a	0.61	-	-
Arhgap6	0.6	-	-
Mindy1	0.6	-	-
Mpzl1	0.6	-	-
Stx7	0.6	-	-
Uba7	0.6	-	-
Trpc6	0.54	-	-
Elmo3	0.54	-	-
Wwp2	0.53	-	-
Il18bp	0.52	-	-
Ly6e	0.51	-	-
Gnpda2	0.51	-	-
Btg2	0.45	-	-
Cd63	0.45	-	-
Zfp932	0.44	-	-
Rpl5	0.36	-	-
Rpl7	0.36	-	-
Gng11	0.34	-	-
Tsc22d1	0.27	-	-
Tmem181b-ps	0	-	-
Map2k7	-0.01	-	-
Asph	-0.03	-	-
Rnf11	-0.03	-	-
Pdzk1ip1	-0.04	-	-
Il17re	-0.08	-	-
Avpi1	-0.1	-	-
Parvg	-0.11	-	-
Fbxo22	-0.12	-	-
Ms4a4c	-0.28	-	-
Rrm2	-0.32	-	-
Gimap4	-0.32	0.81	0.87
Usp1	-0.36	-	-
Adam15	-0.42	-	-

Itgb2	-0.46	-	-
Gng2	-0.47	-	-
Pask	-0.5	-	-
Prim1	-0.53	-	-
Irf2bp2	-0.54	-	-
Lsp1	-0.55	-	0.69
Dtl	-0.55	-	-
Slc28a2	-0.61	-	-
Rin3	-0.62	-	-
Rfc2	-0.64	-	-
Coro2a	-0.64	-	-
Gmnn	-0.64	-	-
Ptpn7	-0.64	-	-
Ptprv	-0.64	-	-
Sla	-0.66	-	-
Ctss	-0.67	-	-
Srm	-0.68	-	-
Zbtb20	-0.68	-	-
Lig1	-0.69	-	-
Dock10	-0.7	-	-
Mlec	-0.73	-	-
Il15	-0.74	-	-
Ccnd2	-0.74	-	-
Anxa2	-0.76	-	-
Syk	-0.76	-	-
Gna15	-0.76	-	-
Arrb2	-0.77	-	-
Ect2	-0.78	-	-
Mcm5	-0.8	-	-
Lmnb1	-0.8	-	-
Snx29	-0.8	-	-
Syncrip	-0.8	-	-
Antxr2	-0.81	-	-
Mamdc2	-0.81	-	-
Gata1	-0.82	-	-
Dnmt1	-0.83	-	-
Cd34	-0.85	-	-0.70
C1qb	-0.85	0.96	-
Ms4a6b	-0.87	-	-
Slc22a3	-0.9	-	-0.94
Plxnd1	-0.9	-	-
Timeless	-0.91	-	-
Arhgap30	-0.92	-	0.63

Lst1	-0.93	-	-
Mgst1	-0.95	-	-
Atp13a2	-0.95	-	-
Arid3b	-0.96	-	-
Uhrf1	-0.96	-	-
Ipcef1	-0.97	-	-
Ttc28	-0.99	-	-
Col4a2	-1	-	-
Phlda2	-1.01	-	-
Tm6sf1	-1.02	-	-
Ebi3	-1.03	-	0.92
Igf2bp2	-1.05	-	-
Dnmt3b	-1.07	-	-
Gria3	-1.07	-	-
Kcna3	-1.07	-	-
Cd37	-1.08	-	-
Plxdc2	-1.1	-	-
Rnase6	-1.12	-	-
Flt3	-1.13	-	-
Sell	-1.14	-	-
Jakmip1	-1.23	-	1.08
Rassf4	-1.26	-	-
Ms4a6c	-1.29	-	-
Cyp27a1	-1.3	-	-
Csf2rb	-1.3	-0.71	-
Phgdh	-1.32	-	-
Plac8	-1.36	-	-0.99
Mcm7	-1.36	-	-
Map10	-1.36	-	-
Rps4l	-1.38	-	-0.81
Gm10384	-1.38	-	-
P2ry14	-1.39	-	0.49
Cd86	-1.39	-	0.75
Hnf4a	-1.4	-	-
Socs2	-1.41	-	-
Igf1	-1.42	-	1.30
Il12rb2	-1.46	-	-
Satb1	-1.47	-	-
Hmga2	-1.58	-	-
Nrk	-1.68	-	-
Cd48	-1.68	-	-
Camk1d	-1.69	-	0.82
Tacc3	-1.84	-	-

Lgals1	-1.86	0.55	-
Dna2	-1.87	-	-
Rgs7bp	-1.94	-	-
Anxa6	-2.05	-	0.66
Mmp2	-2.43	-0.75	-

upplemental Table 2:

Name	logFC aging signature	logFC young TTP vs WT HSC	logFC mid TTP vs WT HSC
Kcnb2	4.72	#N/A	1.1964424
Ntf3	4.67	#N/A	#N/A
Mab21l2	4.47	#N/A	1.20562496
Eya4	4.25	#N/A	1.94275929
Sbspon	4.19	1.2985119	1.04249758
Osmr	4.05	-1.478165	-1.1427045
Cntn1	4.04	#N/A	#N/A
Gabra4	4.03	#N/A	#N/A
Gipc2	3.77	#N/A	-0.9252962
Clu	3.54	#N/A	-1.5023266
Rbpjl	3.45	#N/A	-1.3205759
Zg16	3.15	#N/A	-1.250205
Selp	3.04	-0.912829	-0.6405145
Tdrd9	2.97	#N/A	#N/A
Efemp1	2.97	#N/A	#N/A
Ptprz1	2.92	#N/A	#N/A
Mt2	2.9	1.4260981	#N/A
Lrrn1	2.89	#N/A	-1.1263723
Clca3a1	2.85	-1.556423	-1.7581403
Ripk4	2.84	#N/A	#N/A
Tc2n	2.79	#N/A	#N/A
Plscr2	2.79	-1.007555	#N/A
Matn4	2.78	#N/A	-1.6274574
Fap	2.7	#N/A	#N/A
Muc1	2.69	#N/A	-2.2800693
Ramp2	2.58	#N/A	#N/A
Tmem215	2.56	#N/A	-2.9090775
Trpc1	2.53	#N/A	1.33328607
Chrna7	2.52	#N/A	#N/A
C4a	2.52	#N/A	#N/A
Rgn	2.5	#N/A	#N/A
Rorb	2.49	#N/A	0.90133905
Tm4sf1	2.46	#N/A	#N/A
H2-T3	2.46	#N/A	#N/A
Gm5833	2.44	-0.911099	-1.0373878
Wwtr1	2.43	#N/A	#N/A
Zswim5	2.41	#N/A	#N/A
Gpr183	2.41	0.6323656	#N/A
Ntn4	2.35	0.5857365	0.58830267
C130026I21Rik	2.32	#N/A	0.64995965
Agtr1a	2.32	#N/A	#N/A
C4b	2.28	#N/A	-2.6834082
A530040E14Rik	2.28	#N/A	0.63345152
Sspn	2.28	#N/A	#N/A
Nupr1	2.27	#N/A	#N/A
Vopp1	2.27	#N/A	#N/A
Gstm2	2.26	#N/A	-0.6973893
Bmpr1a	2.21	-0.696951	#N/A
Kdf1	2.19	#N/A	#N/A

Tmem47	2.18	#N/A	#N/A
Gda	2.17	-0.44331	#N/A
Bmp4	2.16	#N/A	#N/A
Klf6	2.16	#N/A	#N/A
Sult1a1	2.15	0.6146267	#N/A
Tmem56	2.14	#N/A	#N/A
Mt1	2.13	0.868561	#N/A
Lhfp	2.07	#N/A	#N/A
Aspa	2.03	#N/A	0.52942363
Cyb561	2.03	#N/A	#N/A
Neo1	2.02	0.4572799	#N/A
Adgrg2	2.01	#N/A	#N/A
Synm	1.99	#N/A	#N/A
Pclo	1.99	-1.197224	-0.5792308
Gm10419	1.98	#N/A	#N/A
Epha2	1.98	#N/A	#N/A
Klrb1c	1.98	0.7835072	#N/A
Gpx8	1.96	#N/A	-0.7854194
Hes1	1.95	#N/A	#N/A
Jam2	1.94	#N/A	#N/A
Gm14964	1.94	1.1732611	0.95258355
Ptgfrn	1.92	#N/A	#N/A
Maf	1.91	#N/A	0.5852734
Dpy19l2	1.9	#N/A	#N/A
Perp	1.87	#N/A	#N/A
Dsg2	1.87	-0.775476	#N/A
Lpl	1.85	-0.619232	#N/A
Pgr	1.84	#N/A	#N/A
Ddr1	1.83	#N/A	#N/A
Clec1a	1.8	#N/A	0.52091632
Dnm3	1.8	#N/A	#N/A
B3galt1	1.79	#N/A	#N/A
Uhrf1bp1l	1.78	#N/A	#N/A
Meis2	1.78	-0.787176	#N/A
Dsc2	1.76	-0.701876	#N/A
Ptprk	1.76	-0.960764	#N/A
Scd1	1.75	#N/A	#N/A
Ehd3	1.72	#N/A	#N/A
Aldh1a1	1.71	#N/A	#N/A
Bhlhe40	1.71	#N/A	#N/A
Ncam1	1.71	-0.626367	-0.7878295
Cavin2	1.69	#N/A	#N/A
Cd53	1.69	#N/A	#N/A
Sema6d	1.68	-1.282368	#N/A
Clec7a	1.67	#N/A	#N/A
Phf11d	1.66	#N/A	#N/A
Abat	1.66	#N/A	#N/A
Runx1t1	1.66	#N/A	#N/A
Amotl2	1.66	#N/A	#N/A
Fam169a	1.65	#N/A	#N/A
Tent5c	1.65	#N/A	#N/A
Exoc3l4	1.63	#N/A	#N/A
Cd200r4	1.62	#N/A	0.82196688
Acpp	1.62	#N/A	#N/A

Pfn2	1.61	#N/A	#N/A
Kdr	1.61	-1.25283	#N/A
Alcam	1.59	#N/A	#N/A
Asb4	1.58	-0.578735	#N/A
Cd38	1.57	#N/A	#N/A
Klhl4	1.57	#N/A	#N/A
Rab2a	1.57	#N/A	#N/A
Col18a1	1.56	#N/A	-1.3083275
Il1rapl2	1.55	#N/A	0.42853914
Enpp5	1.55	#N/A	#N/A
Ror2	1.55	#N/A	#N/A
Gadd45g	1.54	#N/A	#N/A
Timp3	1.54	-0.843192	#N/A
Sfrp1	1.53	#N/A	#N/A
Zfp36	1.52	0.980229	1.00672337
Clec9a	1.51	#N/A	0.6078748
Nrg4	1.51	#N/A	#N/A
Calml4	1.51	-0.665321	#N/A
Cpne8	1.5	#N/A	#N/A
Plk2	1.49	#N/A	0.7121169
Cysltr2	1.47	#N/A	#N/A
Plcl1	1.47	#N/A	#N/A
Fhdc1	1.47	-0.754719	#N/A
Clec1b	1.44	#N/A	#N/A
Abca4	1.44	-0.817924	-1.3725277
Sdcbp	1.42	#N/A	#N/A
Vldlr	1.42	-1.442702	-2.6701872
Pcdhb16	1.41	#N/A	#N/A
Cpt1c	1.41	#N/A	#N/A
Pde9a	1.4	#N/A	-0.7701465
Hpgds	1.4	#N/A	0.68150086
Abcb1a	1.39	#N/A	0.61698256
Nudt10	1.39	#N/A	#N/A
Gkn3	1.39	0.9392928	#N/A
Rab40b	1.38	#N/A	#N/A
Vwf	1.37	#N/A	#N/A
A730089K16Rik	1.37	#N/A	#N/A
Sgk1	1.37	#N/A	#N/A
Ghr	1.36	#N/A	#N/A
Slc6a15	1.36	#N/A	#N/A
AA986860	1.36	#N/A	#N/A
S1pr1	1.35	-0.430027	-0.524265
Mmp14	1.35	-0.747968	-0.6666806
Klrb1b	1.34	#N/A	#N/A
Pkp2	1.34	#N/A	#N/A
Id2	1.33	0.9074485	#N/A
Zfp334	1.33	-0.668357	#N/A
Gem	1.32	0.6713917	0.93805643
Ocln	1.31	#N/A	#N/A
Slc7a7	1.31	#N/A	#N/A
Enkur	1.31	-1.010509	#N/A
Dock9	1.28	#N/A	#N/A
Plek	1.27	#N/A	1.08051308
Ptger4	1.27	#N/A	#N/A

Ldhd	1.26	#N/A	#N/A
Tmem254b	1.26	#N/A	#N/A
Slco2a1	1.26	-0.88687	#N/A
Thbd	1.26	-1.35102	#N/A
Itgb3	1.25	#N/A	-1.1367958
Hoxb6	1.25	#N/A	#N/A
S100a6	1.23	#N/A	#N/A
Lgals3bp	1.23	#N/A	#N/A
Dhrs3	1.22	-0.68957	#N/A
Rhoj	1.22	-0.862329	-0.5145321
Pls3	1.2	#N/A	#N/A
Rdh10	1.2	0.4610163	0.85641297
Fhl1	1.19	#N/A	#N/A
Oxr1	1.19	#N/A	#N/A
Kiss1r	1.19	#N/A	#N/A
Rab34	1.18	#N/A	0.64573268
Rorc	1.18	#N/A	#N/A
Myo1e	1.15	#N/A	-0.9110808
Cyyr1	1.15	#N/A	#N/A
Stxbp4	1.14	#N/A	#N/A
Dennd5b	1.13	#N/A	#N/A
Ampd3	1.13	#N/A	#N/A
Stom	1.12	#N/A	#N/A
Lsr	1.12	#N/A	#N/A
Ccl6	1.11	#N/A	#N/A
Cd74	1.11	#N/A	#N/A
Cyp26b1	1.11	0.9075286	1.25340804
Plscr1	1.1	#N/A	#N/A
Casp12	1.1	#N/A	#N/A
Mllt3	1.1	#N/A	#N/A
Pbx3	1.09	#N/A	#N/A
Tacstd2	1.09	#N/A	#N/A
Evc2	1.09	#N/A	#N/A
Cd55	1.08	#N/A	#N/A
Cpeb2	1.08	#N/A	#N/A
Gabarapl1	1.08	#N/A	#N/A
Bcl6	1.07	#N/A	#N/A
Pdgfd	1.07	#N/A	#N/A
Egr1	1.06	#N/A	#N/A
Cytip	1.05	#N/A	#N/A
Serinc3	1.05	#N/A	#N/A
Exoc3l2	1.05	-0.665187	#N/A
Tox	1.04	#N/A	0.62410044
Ndrg1	1.04	#N/A	#N/A
Cd9	1.04	#N/A	#N/A
Itga6	1.04	#N/A	#N/A
Efna1	1.03	#N/A	#N/A
Vmp1	1.03	#N/A	#N/A
Dstn	1.03	#N/A	#N/A
Erp27	1.03	#N/A	#N/A
Rnf150	1.03	#N/A	#N/A
Trim47	1.01	#N/A	#N/A
Gm3470	1.01	#N/A	#N/A
Tgm2	1	#N/A	#N/A

Rab27b	1	#N/A	#N/A
Ctsc	0.99	#N/A	#N/A
Pip5k1b	0.99	#N/A	#N/A
Chst2	0.98	#N/A	0.9635125
Arhgap29	0.98	#N/A	#N/A
Serpinb8	0.98	#N/A	#N/A
Nabp1	0.98	#N/A	#N/A
Prcp	0.97	#N/A	#N/A
Pros1	0.97	#N/A	#N/A
Prtn3	0.97	#N/A	#N/A
Nckap1	0.97	#N/A	#N/A
Phactr1	0.97	#N/A	#N/A
Tnfsf4	0.97	#N/A	#N/A
Wdfy1	0.97	#N/A	#N/A
Tbc1d8	0.96	#N/A	#N/A
Car5b	0.96	#N/A	#N/A
Cd302	0.96	#N/A	#N/A
Slc14a1	0.95	#N/A	#N/A
Slc44a1	0.95	#N/A	#N/A
Arhgef28	0.95	-0.618876	#N/A
Gpx3	0.94	#N/A	0.61646211
Lamp2	0.94	#N/A	#N/A
Acsl4	0.94	#N/A	#N/A
Cxcl16	0.94	#N/A	#N/A
Pbx1	0.94	-0.427106	-0.4613583
Dhx40	0.93	#N/A	-0.5817347
Sema7a	0.93	#N/A	#N/A
Rbpms2	0.93	#N/A	#N/A
Slamf1	0.92	#N/A	0.4112211
Gm13212	0.92	#N/A	#N/A
Art4	0.91	#N/A	#N/A
Col16a1	0.91	#N/A	#N/A
Selenom	0.91	#N/A	#N/A
Tnfaip2	0.91	#N/A	#N/A
Fosl2	0.91	1.0884155	#N/A
Jun	0.91	-0.643396	#N/A
Serpinb6a	0.89	#N/A	#N/A
Zfp979	0.89	#N/A	#N/A
Fyb	0.88	#N/A	#N/A
Nrgn	0.88	#N/A	#N/A
Evc	0.87	#N/A	#N/A
Kcnip3	0.87	#N/A	#N/A
Dnajb4	0.87	#N/A	#N/A
Zfyve9	0.87	#N/A	#N/A
Mef2c	0.86	#N/A	#N/A
Hoxb5	0.86	#N/A	#N/A
Tnip3	0.85	#N/A	0.44585242
Abcb1b	0.85	#N/A	#N/A
Ctsw	0.85	#N/A	#N/A
Sat1	0.85	#N/A	#N/A
Epb41l5	0.85	-0.595534	#N/A
Clec14a	0.84	#N/A	0.61493522
Lpar6	0.84	#N/A	#N/A
Prnp	0.84	#N/A	#N/A

Rasgef1b	0.83	#N/A	-0.8511978
Chac2	0.83	#N/A	#N/A
Exoc6b	0.82	#N/A	#N/A
Gpr146	0.82	#N/A	#N/A
Pla2g4a	0.81	#N/A	#N/A
Ezh1	0.81	#N/A	#N/A
Gnai3	0.81	#N/A	#N/A
Elovl5	0.8	#N/A	#N/A
Ndn	0.79	#N/A	#N/A
Tbxa2r	0.79	#N/A	#N/A
Npdc1	0.78	#N/A	#N/A
Galnt6	0.78	#N/A	#N/A
Myo6	0.77	#N/A	#N/A
Plscr4	0.76	#N/A	#N/A
Anxa5	0.76	#N/A	#N/A
Arid5b	0.76	#N/A	#N/A
Enpp4	0.76	#N/A	#N/A
Tnfsf10	0.75	#N/A	#N/A
Il1r1	0.75	-0.776767	-0.479363
Hk2	0.74	#N/A	#N/A
Nt5c3	0.71	#N/A	#N/A
Stat3	0.71	#N/A	#N/A
Ppp1r16b	0.7	#N/A	#N/A
Clca1	0.7	#N/A	#N/A
F2rl3	0.7	#N/A	#N/A
Il6st	0.7	#N/A	#N/A
Gstm1	0.69	#N/A	#N/A
Ctse	0.69	#N/A	#N/A
Coq8a	0.68	#N/A	#N/A
Ifi47	0.68	#N/A	#N/A
Ptger3	0.68	#N/A	#N/A
Gstm7	0.67	#N/A	#N/A
Camkk1	0.67	#N/A	#N/A
Cdcp1	0.67	#N/A	#N/A
Pign	0.66	#N/A	#N/A
Mmrn1	0.65	#N/A	#N/A
Cldn12	0.64	#N/A	#N/A
Muc13	0.63	#N/A	-0.6353874
Procr	0.63	#N/A	0.42116227
Tmem176a	0.62	#N/A	#N/A
Slco4a1	0.62	#N/A	#N/A
Ctso	0.61	#N/A	#N/A
Rab11a	0.61	#N/A	#N/A
Arhgap6	0.6	#N/A	#N/A
Mindy1	0.6	#N/A	#N/A
Mpzl1	0.6	#N/A	#N/A
Stx7	0.6	#N/A	#N/A
Uba7	0.6	#N/A	#N/A
Trpc6	0.54	#N/A	#N/A
Elmo3	0.54	#N/A	#N/A
Wwp2	0.53	#N/A	#N/A
Il18bp	0.52	#N/A	#N/A
Ly6e	0.51	#N/A	#N/A
Gnpda2	0.51	#N/A	#N/A

Btg2	0.45	#N/A	#N/A
Cd63	0.45	#N/A	#N/A
Zfp932	0.44	#N/A	#N/A
Rpl5	0.36	#N/A	#N/A
Rpl7	0.36	#N/A	#N/A
Gng11	0.34	#N/A	#N/A
Tsc22d1	0.27	#N/A	#N/A
Tmem181b-ps	0	#N/A	#N/A
Map2k7	-0.01	#N/A	#N/A
Asph	-0.03	#N/A	#N/A
Rnf11	-0.03	#N/A	#N/A
Pdzk1ip1	-0.04	#N/A	#N/A
Il17re	-0.08	#N/A	#N/A
Avpi1	-0.1	#N/A	#N/A
Parvg	-0.11	#N/A	#N/A
Fbxo22	-0.12	#N/A	#N/A
Ms4a4c	-0.28	#N/A	#N/A
Rrm2	-0.32	#N/A	#N/A
Gimap4	-0.32	0.8102715	0.86604523
Usp1	-0.36	#N/A	#N/A
Adam15	-0.42	#N/A	#N/A
Itgb2	-0.46	#N/A	#N/A
Gng2	-0.47	#N/A	#N/A
Pask	-0.5	#N/A	#N/A
Prim1	-0.53	#N/A	#N/A
Irf2bp2	-0.54	#N/A	#N/A
Lsp1	-0.55	#N/A	0.69289168
Dtl	-0.55	#N/A	#N/A
Slc28a2	-0.61	#N/A	#N/A
Rin3	-0.62	#N/A	#N/A
Rfc2	-0.64	#N/A	#N/A
Coro2a	-0.64	#N/A	#N/A
Gmnn	-0.64	#N/A	#N/A
Ptpn7	-0.64	#N/A	#N/A
Ptprv	-0.64	#N/A	#N/A
Sla	-0.66	#N/A	#N/A
Ctss	-0.67	#N/A	#N/A
Srm	-0.68	#N/A	#N/A
Zbtb20	-0.68	#N/A	#N/A
Lig1	-0.69	#N/A	#N/A
Dock10	-0.7	#N/A	#N/A
Mlec	-0.73	#N/A	#N/A
Il15	-0.74	#N/A	#N/A
Ccnd2	-0.74	#N/A	#N/A
Anxa2	-0.76	#N/A	#N/A
Syk	-0.76	#N/A	#N/A
Gna15	-0.76	#N/A	#N/A
Arrb2	-0.77	#N/A	#N/A
Ect2	-0.78	#N/A	#N/A
Mcm5	-0.8	#N/A	#N/A
Lmnb1	-0.8	#N/A	#N/A
Snx29	-0.8	#N/A	#N/A
Syncrip	-0.8	#N/A	#N/A
Antxr2	-0.81	#N/A	#N/A

Mamdc2	-0.81	#N/A	#N/A
Gata1	-0.82	#N/A	#N/A
Dnmt1	-0.83	#N/A	#N/A
Cd34	-0.85	#N/A	-0.7008977
C1qb	-0.85	0.9637288	#N/A
Ms4a6b	-0.87	#N/A	#N/A
Slc22a3	-0.9	#N/A	-0.9431194
Plxnd1	-0.9	#N/A	#N/A
Timeless	-0.91	#N/A	#N/A
Arhgap30	-0.92	#N/A	0.63130119
Lst1	-0.93	#N/A	#N/A
Mgst1	-0.95	#N/A	#N/A
Atp13a2	-0.95	#N/A	#N/A
Arid3b	-0.96	#N/A	#N/A
Uhrf1	-0.96	#N/A	#N/A
Ipcef1	-0.97	#N/A	#N/A
Ttc28	-0.99	#N/A	#N/A
Col4a2	-1	#N/A	#N/A
Phlda2	-1.01	#N/A	#N/A
Tm6sf1	-1.02	#N/A	#N/A
Ebi3	-1.03	#N/A	0.9180567
Igf2bp2	-1.05	#N/A	#N/A
Dnmt3b	-1.07	#N/A	#N/A
Gria3	-1.07	#N/A	#N/A
Kcna3	-1.07	#N/A	#N/A
Cd37	-1.08	#N/A	#N/A
Plxdc2	-1.1	#N/A	#N/A
Rnase6	-1.12	#N/A	#N/A
Flt3	-1.13	#N/A	#N/A
Sell	-1.14	#N/A	#N/A
Jakmip1	-1.23	#N/A	1.08152456
Rassf4	-1.26	#N/A	#N/A
Ms4a6c	-1.29	#N/A	#N/A
Cyp27a1	-1.3	#N/A	#N/A
Csf2rb	-1.3	-0.714487	#N/A
Phgdh	-1.32	#N/A	#N/A
Plac8	-1.36	#N/A	-0.9943274
Mcm7	-1.36	#N/A	#N/A
Map10	-1.36	#N/A	#N/A
Rps4l	-1.38	#N/A	-0.8087784
Gm10384	-1.38	#N/A	#N/A
P2ry14	-1.39	#N/A	0.48560103
Cd86	-1.39	#N/A	0.75240622
Hnf4a	-1.4	#N/A	#N/A
Socs2	-1.41	#N/A	#N/A
Igf1	-1.42	#N/A	1.30146148
Il12rb2	-1.46	#N/A	#N/A
Satb1	-1.47	#N/A	#N/A
Hmga2	-1.58	#N/A	#N/A
Nrk	-1.68	#N/A	#N/A
Cd48	-1.68	#N/A	#N/A
Camk1d	-1.69	#N/A	0.81913777
Tacc3	-1.84	#N/A	#N/A
Lgals1	-1.86	0.5478138	#N/A

Dna2	-1.87	#N/A	#N/A
Rgs7bp	-1.94	#N/A	#N/A
Anxa6	-2.05	#N/A	0.65701615
Mmp2	-2.43	-0.751231	#N/A
