# Supplementary material for: Tristetraprolin overexpression drives hematopoietic changes in young and middle-aged mice generating dominant mitigating effects on induced inflammation in murine models
Source: GeroScience. 2023 Aug 3;46(1):1271–84. doi: 10.1007/s11357-023-00879-2 (PMC10828162; doi:10.1007/s11357-023-00879-2)
Supplement: Supplementary file 4 — Supplementary file4 (PDF 348 KB) [file 11357_2023_879_MOESM4_ESM.pdf]

Supplemental Table 3_POSTAR3_CLIP

Target gene symbol	Target gene ID	Target gene type	Protocol	Binding site records
Fam107b	ENSMUSG00000026655	protein_coding	HITS-CLIP,Piranha_0.01	2397
Picalm	ENSMUSG00000039361	protein_coding	HITS-CLIP,Piranha_0.01	705
Bach2	ENSMUSG00000040270	protein_coding	HITS-CLIP,Piranha_0.01	453
Satb1	ENSMUSG00000023927	protein_coding	HITS-CLIP,Piranha_0.01	451
Jarid2	ENSMUSG00000038518	protein_coding	HITS-CLIP,Piranha_0.01	418
Malat1	ENSMUSG00000092341	lincRNA	HITS-CLIP,Piranha_0.01	396
Rpl13a	ENSMUSG00000074129	protein_coding	HITS-CLIP,Piranha_0.01	363
Il2ra	ENSMUSG00000026770	protein_coding	HITS-CLIP,Piranha_0.01	361
Nfkb1	ENSMUSG00000028163	protein_coding	HITS-CLIP,Piranha_0.01	360
Rbpj	ENSMUSG00000039191	protein_coding	HITS-CLIP,Piranha_0.01	322
Maml2	ENSMUSG00000031925	protein_coding	HITS-CLIP,Piranha_0.01	304
Furin	ENSMUSG00000030530	protein_coding	HITS-CLIP,Piranha_0.01	293
Mbnl1	ENSMUSG00000027763	protein_coding	HITS-CLIP,Piranha_0.01	231
CT010467.1	ENSMUSG00000106106	rRNA	HITS-CLIP,Piranha_0.01	229
Cd44	ENSMUSG00000005087	protein_coding	HITS-CLIP,Piranha_0.01	221
Cd247	ENSMUSG00000005763	protein_coding	HITS-CLIP,Piranha_0.01	197
Irf4	ENSMUSG00000021356	protein_coding	HITS-CLIP,Piranha_0.01	196
Malt1	ENSMUSG00000032688	protein_coding	HITS-CLIP,Piranha_0.01	192
Nfat5	ENSMUSG00000003847	protein_coding	HITS-CLIP,Piranha_0.01	188
Ptprc	ENSMUSG00000026395	protein_coding	HITS-CLIP,Piranha_0.01	187
Jak2	ENSMUSG00000024789	protein_coding	HITS-CLIP,Piranha_0.01	178
Myc	ENSMUSG00000022346	protein_coding	HITS-CLIP,Piranha_0.01	178
Cdk8	ENSMUSG00000029635	protein_coding	HITS-CLIP,Piranha_0.01	175
Cblb	ENSMUSG00000022637	protein_coding	HITS-CLIP,Piranha_0.01	162
Gm47283	ENSMUSG00000096768	lincRNA	HITS-CLIP,Piranha_0.01	147
Hif1a	ENSMUSG00000021109	protein_coding	HITS-CLIP,Piranha_0.01	139
Nr4a3	ENSMUSG00000028341	protein_coding	HITS-CLIP,Piranha_0.01	137
Gramd1b	ENSMUSG00000040111	protein_coding	HITS-CLIP,Piranha_0.01	134
Peli1	ENSMUSG00000020134	protein_coding	HITS-CLIP,Piranha_0.01	131
Rabggtb	ENSMUSG00000038975	protein_coding	HITS-CLIP,Piranha_0.01	131
Srgn	ENSMUSG00000020077	protein_coding	HITS-CLIP,Piranha_0.01	128
Rora	ENSMUSG00000032238	protein_coding	HITS-CLIP,Piranha_0.01	125
Cdk8	ENSMUSG00000029635	protein_coding	HITS-CLIP,CTK	120
Morrbid	ENSMUSG00000074813	lincRNA	HITS-CLIP,Piranha_0.01	116
Trpm3	ENSMUSG00000052387	protein_coding	HITS-CLIP,Piranha_0.01	116
Tnfsf8	ENSMUSG00000028362	protein_coding	HITS-CLIP,Piranha_0.01	115
Kcnip1	ENSMUSG00000053519	protein_coding	HITS-CLIP,Piranha_0.01	114
Lif	ENSMUSG00000034394	protein_coding	HITS-CLIP,Piranha_0.01	111
Gas5	ENSMUSG00000053332	processed_transcri	HITS-CLIP,Piranha_0.01	105
Il21	ENSMUSG00000027718	protein_coding	HITS-CLIP,Piranha_0.01	103
Il31ra	ENSMUSG00000050377	protein_coding	HITS-CLIP,Piranha_0.01	102
Hnrnpk	ENSMUSG00000021546	protein_coding	HITS-CLIP,Piranha_0.01	100
Mir155hg	ENSMUSG00000097418	lincRNA	HITS-CLIP,Piranha_0.01	98
Actb	ENSMUSG00000029580	protein_coding	HITS-CLIP,Piranha_0.01	95
Sh3kbp1	ENSMUSG00000040990	protein_coding	HITS-CLIP,Piranha_0.01	93
Snhg1	ENSMUSG00000108414	processed_transcri	HITS-CLIP,Piranha_0.01	93
Sik3	ENSMUSG00000034135	protein_coding	HITS-CLIP,Piranha_0.01	92
Il2	ENSMUSG00000027720	protein_coding	HITS-CLIP,Piranha_0.01	85
Lrrfip1	ENSMUSG00000026305	protein_coding	HITS-CLIP,Piranha_0.01	84
Tor1aip2	ENSMUSG00000050565	protein_coding	HITS-CLIP,Piranha_0.01	84
Ikzf1	ENSMUSG00000018654	protein_coding	HITS-CLIP,Piranha_0.01	83
Tnf	ENSMUSG00000024401	protein_coding	HITS-CLIP,Piranha_0.01	82
Spry1	ENSMUSG00000037211	protein_coding	HITS-CLIP,Piranha_0.01	81
Emp1	ENSMUSG00000030208	protein_coding	HITS-CLIP,Piranha_0.01	80

Ets1	ENSMUSG00000032035	protein_coding	HITS-CLIP,Piranha_0.01	80
Smc4	ENSMUSG00000034349	protein_coding	HITS-CLIP,Piranha_0.01	80
Hspa5	ENSMUSG00000026864	protein_coding	HITS-CLIP,Piranha_0.01	78
Slco3a1	ENSMUSG00000025790	protein_coding	HITS-CLIP,Piranha_0.01	75
Spata5	ENSMUSG00000027722	protein_coding	HITS-CLIP,Piranha_0.01	75
Ccnd2	ENSMUSG00000000184	protein_coding	HITS-CLIP,Piranha_0.01	74
Lars2	ENSMUSG00000035202	protein_coding	HITS-CLIP,Piranha_0.01	74
Acsl4	ENSMUSG00000031278	protein_coding	HITS-CLIP,Piranha_0.01	72
Aebp2	ENSMUSG00000030232	protein_coding	HITS-CLIP,Piranha_0.01	72
Macf1	ENSMUSG00000028649	protein_coding	HITS-CLIP,Piranha_0.01	72
Rpl3	ENSMUSG00000060036	protein_coding	HITS-CLIP,Piranha_0.01	72
Rps8	ENSMUSG00000047675	protein_coding	HITS-CLIP,Piranha_0.01	72
Il17f	ENSMUSG00000041872	protein_coding	HITS-CLIP,Piranha_0.01	69
Fyn	ENSMUSG00000019843	protein_coding	HITS-CLIP,Piranha_0.01	67
Eif5a	ENSMUSG00000078812	protein_coding	HITS-CLIP,Piranha_0.01	66
Snhg6	ENSMUSG00000098234	lincRNA	HITS-CLIP,Piranha_0.01	66
Trrap	ENSMUSG00000045482	protein_coding	HITS-CLIP,Piranha_0.01	66
Hnrnpa2b1	ENSMUSG00000004980	protein_coding	HITS-CLIP,Piranha_0.01	65
Mdfic	ENSMUSG00000041390	protein_coding	HITS-CLIP,Piranha_0.01	64
Nr4a1	ENSMUSG00000023034	protein_coding	HITS-CLIP,Piranha_0.01	64
Rps3	ENSMUSG00000030744	protein_coding	HITS-CLIP,Piranha_0.01	63
Tmsb4x	ENSMUSG00000049775	protein_coding	HITS-CLIP,Piranha_0.01	63
Zfp36l1	ENSMUSG00000021127	protein_coding	HITS-CLIP,Piranha_0.01	61
Cdk17	ENSMUSG00000020015	protein_coding	HITS-CLIP,Piranha_0.01	60
Dock10	ENSMUSG00000038608	protein_coding	HITS-CLIP,Piranha_0.01	60
Myo10	ENSMUSG00000022272	protein_coding	HITS-CLIP,Piranha_0.01	60
Rgs1	ENSMUSG00000026358	protein_coding	HITS-CLIP,Piranha_0.01	59
CT010467.1	ENSMUSG00000106106	rRNA	HITS-CLIP,CTK	58
Hnrnpc	ENSMUSG00000060373	protein_coding	HITS-CLIP,Piranha_0.01	58
Noct	ENSMUSG00000023087	protein_coding	HITS-CLIP,Piranha_0.01	58
Camk4	ENSMUSG00000038128	protein_coding	HITS-CLIP,Piranha_0.01	56
Myl12a	ENSMUSG00000024048	protein_coding	HITS-CLIP,Piranha_0.01	56
Pum2	ENSMUSG00000020594	protein_coding	HITS-CLIP,Piranha_0.01	56
Acly	ENSMUSG00000020917	protein_coding	HITS-CLIP,Piranha_0.01	55
Arl5b	ENSMUSG00000017418	protein_coding	HITS-CLIP,Piranha_0.01	55
Ext1	ENSMUSG00000061731	protein_coding	HITS-CLIP,Piranha_0.01	55
Sla	ENSMUSG00000022372	protein_coding	HITS-CLIP,Piranha_0.01	55
Inpp4b	ENSMUSG00000037940	protein_coding	HITS-CLIP,Piranha_0.01	54
Stx11	ENSMUSG00000039232	protein_coding	HITS-CLIP,Piranha_0.01	54
Itgav	ENSMUSG00000027087	protein_coding	HITS-CLIP,Piranha_0.01	52
Dnmt3a	ENSMUSG00000020661	protein_coding	HITS-CLIP,Piranha_0.01	51
Gnas	ENSMUSG00000027523	protein_coding	HITS-CLIP,Piranha_0.01	51
Aopep	ENSMUSG00000021458	protein_coding	HITS-CLIP,Piranha_0.01	50
Camk2d	ENSMUSG00000053819	protein_coding	HITS-CLIP,Piranha_0.01	50
Man1a	ENSMUSG00000003746	protein_coding	HITS-CLIP,Piranha_0.01	50
Rnu3a	ENSMUSG00000106147	snoRNA	HITS-CLIP,Piranha_0.01	50
Cep295	ENSMUSG00000046111	protein_coding	HITS-CLIP,Piranha_0.01	49
Eif4a1	ENSMUSG00000059796	protein_coding	HITS-CLIP,Piranha_0.01	49
Etv6	ENSMUSG00000030199	protein_coding	HITS-CLIP,Piranha_0.01	49
Matr3	ENSMUSG00000037236	protein_coding	HITS-CLIP,Piranha_0.01	49
Ptbp3	ENSMUSG00000028382	protein_coding	HITS-CLIP,Piranha_0.01	49
Slc4a7	ENSMUSG00000021733	protein_coding	HITS-CLIP,Piranha_0.01	49
Gphn	ENSMUSG00000047454	protein_coding	HITS-CLIP,Piranha_0.01	48
Rc3h2	ENSMUSG00000075376	protein_coding	HITS-CLIP,Piranha_0.01	48
Cnot1	ENSMUSG00000036550	protein_coding	HITS-CLIP,Piranha_0.01	47
Gigyf2	ENSMUSG00000048000	protein_coding	HITS-CLIP,Piranha_0.01	47
Abi1	ENSMUSG00000058835	protein_coding	HITS-CLIP,Piranha_0.01	46
B2m	ENSMUSG00000060802	protein_coding	HITS-CLIP,Piranha_0.01	46

Hnrnpab	ENSMUSG00000020358	protein_coding	HITS-CLIP,Piranha_0.01	46
Cpeb3	ENSMUSG00000039652	protein_coding	HITS-CLIP,Piranha_0.01	45
Kcnq5	ENSMUSG00000028033	protein_coding	HITS-CLIP,Piranha_0.01	45
Spata17	ENSMUSG00000026611	protein_coding	HITS-CLIP,Piranha_0.01	45
Alcam	ENSMUSG00000022636	protein_coding	HITS-CLIP,Piranha_0.01	44
Cd53	ENSMUSG00000040747	protein_coding	HITS-CLIP,Piranha_0.01	44
Dot1l	ENSMUSG00000061589	protein_coding	HITS-CLIP,Piranha_0.01	44
Ifng	ENSMUSG00000055170	protein_coding	HITS-CLIP,Piranha_0.01	44
Pkm	ENSMUSG00000032294	protein_coding	HITS-CLIP,Piranha_0.01	44
Sema4d	ENSMUSG00000021451	protein_coding	HITS-CLIP,Piranha_0.01	44
Zeb1	ENSMUSG00000024238	protein_coding	HITS-CLIP,Piranha_0.01	44
Nop56	ENSMUSG00000027405	protein_coding	HITS-CLIP,Piranha_0.01	43
Sltm	ENSMUSG00000032212	protein_coding	HITS-CLIP,Piranha_0.01	43
Stat5a	ENSMUSG00000004043	protein_coding	HITS-CLIP,Piranha_0.01	43
Acin1	ENSMUSG00000022185	protein_coding	HITS-CLIP,Piranha_0.01	42
Camk1d	ENSMUSG00000039145	protein_coding	HITS-CLIP,Piranha_0.01	42
Cdkn1a	ENSMUSG00000023067	protein_coding	HITS-CLIP,Piranha_0.01	42
Ica1	ENSMUSG00000062995	protein_coding	HITS-CLIP,Piranha_0.01	42
Mid1	ENSMUSG00000035299	protein_coding	HITS-CLIP,Piranha_0.01	42
Pfn1	ENSMUSG00000018293	protein_coding	HITS-CLIP,Piranha_0.01	42
Tcf12	ENSMUSG00000032228	protein_coding	HITS-CLIP,Piranha_0.01	42
Tnfaip3	ENSMUSG00000019850	protein_coding	HITS-CLIP,Piranha_0.01	42
Fbxo11	ENSMUSG00000005371	protein_coding	HITS-CLIP,Piranha_0.01	41
Chn2	ENSMUSG00000004633	protein_coding	HITS-CLIP,Piranha_0.01	40
Epb41	ENSMUSG00000028906	protein_coding	HITS-CLIP,Piranha_0.01	40
Hivep2	ENSMUSG00000015501	protein_coding	HITS-CLIP,Piranha_0.01	40
Kansl2	ENSMUSG00000022992	protein_coding	HITS-CLIP,Piranha_0.01	40
Lcp2	ENSMUSG00000002699	protein_coding	HITS-CLIP,Piranha_0.01	40
Ube2f	ENSMUSG00000034343	protein_coding	HITS-CLIP,Piranha_0.01	39
Zeb2	ENSMUSG00000026872	protein_coding	HITS-CLIP,Piranha_0.01	39
Hexb	ENSMUSG00000021665	protein_coding	HITS-CLIP,Piranha_0.01	38
Themis	ENSMUSG00000049109	protein_coding	HITS-CLIP,Piranha_0.01	38
Cnbp	ENSMUSG00000030057	protein_coding	HITS-CLIP,Piranha_0.01	37
Fnbp1	ENSMUSG00000075415	protein_coding	HITS-CLIP,Piranha_0.01	37
Foxp1	ENSMUSG00000030067	protein_coding	HITS-CLIP,Piranha_0.01	37
Lcp1	ENSMUSG00000021998	protein_coding	HITS-CLIP,Piranha_0.01	37
Ly6a	ENSMUSG00000075602	protein_coding	HITS-CLIP,Piranha_0.01	37
Trp53inp1	ENSMUSG00000028211	protein_coding	HITS-CLIP,Piranha_0.01	37
Clmn	ENSMUSG00000021097	protein_coding	HITS-CLIP,Piranha_0.01	36
Ndfip1	ENSMUSG00000024425	protein_coding	HITS-CLIP,Piranha_0.01	36
Gm19951	ENSMUSG00000113136	lincRNA	HITS-CLIP,Piranha_0.01	35
Hmcn2	ENSMUSG00000055632	protein_coding	HITS-CLIP,Piranha_0.01	35
Egln3	ENSMUSG00000035105	protein_coding	HITS-CLIP,Piranha_0.01	34
Inhba	ENSMUSG00000041324	protein_coding	HITS-CLIP,Piranha_0.01	34
Kpna4	ENSMUSG00000027782	protein_coding	HITS-CLIP,Piranha_0.01	34
Pim1	ENSMUSG00000024014	protein_coding	HITS-CLIP,Piranha_0.01	34
Nfkb2	ENSMUSG00000025225	protein_coding	HITS-CLIP,Piranha_0.01	33
Nfkbid	ENSMUSG00000036931	protein_coding	HITS-CLIP,Piranha_0.01	33
Phf21a	ENSMUSG00000058318	protein_coding	HITS-CLIP,Piranha_0.01	33
Prkch	ENSMUSG00000021108	protein_coding	HITS-CLIP,Piranha_0.01	33
1110038B12Rik	ENSMUSG00000092203	processed_transcri	HITS-CLIP,Piranha_0.01	32
4933404O12Rik	ENSMUSG00000097908	lincRNA	HITS-CLIP,Piranha_0.01	32
Ddx5	ENSMUSG00000020719	protein_coding	HITS-CLIP,Piranha_0.01	32
Il10	ENSMUSG00000016529	protein_coding	HITS-CLIP,Piranha_0.01	32
Larp1	ENSMUSG00000037331	protein_coding	HITS-CLIP,Piranha_0.01	32
Prrc2c	ENSMUSG00000040225	protein_coding	HITS-CLIP,Piranha_0.01	32
Septin2	ENSMUSG00000026276	protein_coding	HITS-CLIP,Piranha_0.01	32
Ybx3	ENSMUSG00000030189	protein_coding	HITS-CLIP,Piranha_0.01	32

Znrf1	ENSMUSG00000033545	protein_coding	HITS-CLIP,Piranha_0.01	32
Atg16l1	ENSMUSG00000026289	protein_coding	HITS-CLIP,Piranha_0.01	30
Cd40lg	ENSMUSG00000031132	protein_coding	HITS-CLIP,Piranha_0.01	30
Dsn1	ENSMUSG00000027635	protein_coding	HITS-CLIP,Piranha_0.01	30
Gm49751	ENSMUSG00000111913	TEC	HITS-CLIP,Piranha_0.01	30
Grik2	ENSMUSG00000056073	protein_coding	HITS-CLIP,Piranha_0.01	30
Il6st	ENSMUSG00000021756	protein_coding	HITS-CLIP,Piranha_0.01	30
Ldha	ENSMUSG00000063229	protein_coding	HITS-CLIP,Piranha_0.01	30
Mdm2	ENSMUSG00000020184	protein_coding	HITS-CLIP,Piranha_0.01	30
Mier1	ENSMUSG00000028522	protein_coding	HITS-CLIP,Piranha_0.01	30
Pbx1	ENSMUSG00000052534	protein_coding	HITS-CLIP,Piranha_0.01	30
Serbp1	ENSMUSG00000036371	protein_coding	HITS-CLIP,Piranha_0.01	30
Socs1	ENSMUSG00000038037	protein_coding	HITS-CLIP,Piranha_0.01	30
Vmp1	ENSMUSG00000018171	protein_coding	HITS-CLIP,Piranha_0.01	30
Cflar	ENSMUSG00000026031	protein_coding	HITS-CLIP,Piranha_0.01	29
Cmss1	ENSMUSG00000022748	protein_coding	HITS-CLIP,Piranha_0.01	29
Tnfrsf9	ENSMUSG00000028965	protein_coding	HITS-CLIP,Piranha_0.01	29
Ahcyl2	ENSMUSG00000029772	protein_coding	HITS-CLIP,Piranha_0.01	28
Btla	ENSMUSG00000052013	protein_coding	HITS-CLIP,Piranha_0.01	28
Camta1	ENSMUSG00000014592	protein_coding	HITS-CLIP,Piranha_0.01	28
Cd47	ENSMUSG00000055447	protein_coding	HITS-CLIP,Piranha_0.01	28
Itk	ENSMUSG00000020395	protein_coding	HITS-CLIP,Piranha_0.01	28
Luc7l2	ENSMUSG00000029823	protein_coding	HITS-CLIP,Piranha_0.01	28
Ptprd	ENSMUSG00000028399	protein_coding	HITS-CLIP,Piranha_0.01	28
Ric3	ENSMUSG00000048330	protein_coding	HITS-CLIP,Piranha_0.01	28
St14	ENSMUSG00000031995	protein_coding	HITS-CLIP,Piranha_0.01	28
Zfp36	ENSMUSG00000044786	protein_coding	HITS-CLIP,Piranha_0.01	28
Btbd9	ENSMUSG00000062202	protein_coding	HITS-CLIP,Piranha_0.01	27
Chd7	ENSMUSG00000041235	protein_coding	HITS-CLIP,Piranha_0.01	27
Nap1l4	ENSMUSG00000059119	protein_coding	HITS-CLIP,Piranha_0.01	27
Arpc5	ENSMUSG00000008475	protein_coding	HITS-CLIP,Piranha_0.01	26
Camk1d	ENSMUSG00000039145	protein_coding	HITS-CLIP,CTK	26
Eef2	ENSMUSG00000034994	protein_coding	HITS-CLIP,Piranha_0.01	26
Hexb	ENSMUSG00000021665	protein_coding	HITS-CLIP,CTK	26
Mapk6	ENSMUSG00000042688	protein_coding	HITS-CLIP,Piranha_0.01	26
Nabp1	ENSMUSG00000026107	protein_coding	HITS-CLIP,Piranha_0.01	26
Npm1	ENSMUSG00000057113	protein_coding	HITS-CLIP,Piranha_0.01	26
Rpl23a	ENSMUSG00000058546	protein_coding	HITS-CLIP,Piranha_0.01	26
Ccl4	ENSMUSG00000018930	protein_coding	HITS-CLIP,Piranha_0.01	25
Eps15	ENSMUSG00000028552	protein_coding	HITS-CLIP,Piranha_0.01	25
Gm45713	ENSMUSG00000089989	protein_coding	HITS-CLIP,Piranha_0.01	25
Hnrnpl	ENSMUSG00000015165	protein_coding	HITS-CLIP,Piranha_0.01	25
Nfe2l1	ENSMUSG00000038615	protein_coding	HITS-CLIP,Piranha_0.01	25
Pfkfb3	ENSMUSG00000026773	protein_coding	HITS-CLIP,Piranha_0.01	25
Ptpn22	ENSMUSG00000027843	protein_coding	HITS-CLIP,Piranha_0.01	25
Pvt1	ENSMUSG00000097039	lincRNA	HITS-CLIP,Piranha_0.01	25
Rbm25	ENSMUSG00000010608	protein_coding	HITS-CLIP,Piranha_0.01	25
Rpl23	ENSMUSG00000071415	protein_coding	HITS-CLIP,Piranha_0.01	25
Rrm2b	ENSMUSG00000022292	protein_coding	HITS-CLIP,Piranha_0.01	25
4930555F03Rik	ENSMUSG00000031559	lincRNA	HITS-CLIP,Piranha_0.01	24
5430416N02Rik	ENSMUSG00000097772	lincRNA	HITS-CLIP,Piranha_0.01	24
Abtb2	ENSMUSG00000032724	protein_coding	HITS-CLIP,Piranha_0.01	24
Atp13a3	ENSMUSG00000022533	protein_coding	HITS-CLIP,Piranha_0.01	24
Cd44	ENSMUSG00000005087	protein_coding	HITS-CLIP,CTK	24
Chchd3	ENSMUSG00000053768	protein_coding	HITS-CLIP,Piranha_0.01	24
Chd3	ENSMUSG00000018474	protein_coding	HITS-CLIP,Piranha_0.01	24
Csrnp1	ENSMUSG00000032515	protein_coding	HITS-CLIP,Piranha_0.01	24
Dhx9	ENSMUSG00000042699	protein_coding	HITS-CLIP,Piranha_0.01	24

Eef1a1	ENSMUSG00000037742	protein_coding	HITS-CLIP,Piranha_0.01	24
Gm20186	ENSMUSG00000106874	lincRNA	HITS-CLIP,Piranha_0.01	24
Gm40645	ENSMUSG00000112766	antisense	HITS-CLIP,Piranha_0.01	24
Nop58	ENSMUSG00000026020	protein_coding	HITS-CLIP,Piranha_0.01	24
Nrg1	ENSMUSG00000062991	protein_coding	HITS-CLIP,Piranha_0.01	24
Plekha5	ENSMUSG00000030231	protein_coding	HITS-CLIP,Piranha_0.01	24
Ppp1r8	ENSMUSG00000028882	protein_coding	HITS-CLIP,Piranha_0.01	24
Rbmx	ENSMUSG00000031134	protein_coding	HITS-CLIP,Piranha_0.01	24
Snhg4	ENSMUSG00000117869	processed_transcri	HITS-CLIP,Piranha_0.01	24
Snora16a	ENSMUSG00000105911	snoRNA	HITS-CLIP,Piranha_0.01	24
Zfand3	ENSMUSG00000044477	protein_coding	HITS-CLIP,Piranha_0.01	24
Agfg1	ENSMUSG00000026159	protein_coding	HITS-CLIP,Piranha_0.01	23
Dusp16	ENSMUSG00000030203	protein_coding	HITS-CLIP,Piranha_0.01	23
Lrig1	ENSMUSG00000030029	protein_coding	HITS-CLIP,Piranha_0.01	23
Msn	ENSMUSG00000031207	protein_coding	HITS-CLIP,Piranha_0.01	23
Zfp36l2	ENSMUSG00000045817	protein_coding	HITS-CLIP,Piranha_0.01	23
B930036N10Rik	ENSMUSG00000091993	antisense	HITS-CLIP,Piranha_0.01	22
Cd69	ENSMUSG00000030156	protein_coding	HITS-CLIP,Piranha_0.01	22
Elavl1	ENSMUSG00000040028	protein_coding	HITS-CLIP,Piranha_0.01	22
Gm22711	ENSMUSG00000105884	snoRNA	HITS-CLIP,Piranha_0.01	22
Hk2	ENSMUSG00000000628	protein_coding	HITS-CLIP,Piranha_0.01	22
Plk3	ENSMUSG00000028680	protein_coding	HITS-CLIP,Piranha_0.01	22
Rps6ka3	ENSMUSG00000031309	protein_coding	HITS-CLIP,Piranha_0.01	22
Tfrc	ENSMUSG00000022797	protein_coding	HITS-CLIP,Piranha_0.01	22
Ablim1	ENSMUSG00000025085	protein_coding	HITS-CLIP,Piranha_0.01	21
Eif1a	ENSMUSG00000057561	protein_coding	HITS-CLIP,Piranha_0.01	21
Gm47087	ENSMUSG00000111212	TEC	HITS-CLIP,Piranha_0.01	21
Ivns1abp	ENSMUSG00000023150	protein_coding	HITS-CLIP,Piranha_0.01	21
Junb	ENSMUSG00000052837	protein_coding	HITS-CLIP,Piranha_0.01	21
Pbx3	ENSMUSG00000038718	protein_coding	HITS-CLIP,Piranha_0.01	21
Rbm6	ENSMUSG00000032582	protein_coding	HITS-CLIP,Piranha_0.01	21
Slc16a3	ENSMUSG00000025161	protein_coding	HITS-CLIP,Piranha_0.01	21
Sorcs1	ENSMUSG00000043531	protein_coding	HITS-CLIP,Piranha_0.01	21
4932438A13Rik	ENSMUSG00000037270	protein_coding	HITS-CLIP,Piranha_0.01	20
Ccnl1	ENSMUSG00000027829	protein_coding	HITS-CLIP,Piranha_0.01	20
Cish	ENSMUSG00000032578	protein_coding	HITS-CLIP,Piranha_0.01	20
Cox7c	ENSMUSG00000017778	protein_coding	HITS-CLIP,Piranha_0.01	20
Cpeb2	ENSMUSG00000039782	protein_coding	HITS-CLIP,Piranha_0.01	20
Cux1	ENSMUSG00000029705	protein_coding	HITS-CLIP,Piranha_0.01	20
Cytip	ENSMUSG00000026832	protein_coding	HITS-CLIP,Piranha_0.01	20
Dcc	ENSMUSG00000060534	protein_coding	HITS-CLIP,Piranha_0.01	20
Eif4g2	ENSMUSG00000005610	protein_coding	HITS-CLIP,Piranha_0.01	20
Gm26447	ENSMUSG00000064382	snoRNA	HITS-CLIP,Piranha_0.01	20
Gm44511	ENSMUSG00000107872	protein_coding	HITS-CLIP,Piranha_0.01	20
Gnb1	ENSMUSG00000029064	protein_coding	HITS-CLIP,Piranha_0.01	20
Kdm6b	ENSMUSG00000018476	protein_coding	HITS-CLIP,Piranha_0.01	20
Macrod2	ENSMUSG00000068205	protein_coding	HITS-CLIP,Piranha_0.01	20
Map2k3	ENSMUSG00000018932	protein_coding	HITS-CLIP,Piranha_0.01	20
Nfatc1	ENSMUSG00000033016	protein_coding	HITS-CLIP,Piranha_0.01	20
Ogt	ENSMUSG00000034160	protein_coding	HITS-CLIP,Piranha_0.01	20
Peak1	ENSMUSG00000074305	protein_coding	HITS-CLIP,Piranha_0.01	20
Pip5k1a	ENSMUSG00000028126	protein_coding	HITS-CLIP,Piranha_0.01	20
Prkca	ENSMUSG00000050965	protein_coding	HITS-CLIP,Piranha_0.01	20
Sema7a	ENSMUSG00000038264	protein_coding	HITS-CLIP,Piranha_0.01	20
Snx5	ENSMUSG00000027423	protein_coding	HITS-CLIP,Piranha_0.01	20
Spag16	ENSMUSG00000053153	protein_coding	HITS-CLIP,Piranha_0.01	20
Stx16	ENSMUSG00000027522	protein_coding	HITS-CLIP,Piranha_0.01	20
Tra2a	ENSMUSG00000029817	protein_coding	HITS-CLIP,Piranha_0.01	20

Trib2	ENSMUSG00000020601	protein_coding	HITS-CLIP,Piranha_0.01	20
Ubash3b	ENSMUSG00000032020	protein_coding	HITS-CLIP,Piranha_0.01	20
Wnk1	ENSMUSG00000045962	protein_coding	HITS-CLIP,Piranha_0.01	20
Cacna1c	ENSMUSG00000051331	protein_coding	HITS-CLIP,Piranha_0.01	19
Hnrnpm	ENSMUSG00000059208	protein_coding	HITS-CLIP,Piranha_0.01	19
Il4	ENSMUSG00000000869	protein_coding	HITS-CLIP,Piranha_0.01	19
Spag9	ENSMUSG00000020859	protein_coding	HITS-CLIP,Piranha_0.01	19
9330185C12Rik	ENSMUSG00000097648	lincRNA	HITS-CLIP,Piranha_0.01	18
Abl1	ENSMUSG00000026842	protein_coding	HITS-CLIP,Piranha_0.01	18
Adgb	ENSMUSG00000050994	protein_coding	HITS-CLIP,Piranha_0.01	18
Brd2	ENSMUSG00000024335	protein_coding	HITS-CLIP,Piranha_0.01	18
Ccl20	ENSMUSG00000026166	protein_coding	HITS-CLIP,Piranha_0.01	18
Cdc7	ENSMUSG00000029283	protein_coding	HITS-CLIP,Piranha_0.01	18
Cdh4	ENSMUSG00000000305	protein_coding	HITS-CLIP,Piranha_0.01	18
Cdv3	ENSMUSG00000032803	protein_coding	HITS-CLIP,Piranha_0.01	18
Dpyd	ENSMUSG00000033308	protein_coding	HITS-CLIP,Piranha_0.01	18
Dst	ENSMUSG00000026131	protein_coding	HITS-CLIP,Piranha_0.01	18
Fam49b	ENSMUSG00000022378	protein_coding	HITS-CLIP,Piranha_0.01	18
Fbxw7	ENSMUSG00000028086	protein_coding	HITS-CLIP,Piranha_0.01	18
Pde4b	ENSMUSG00000028525	protein_coding	HITS-CLIP,Piranha_0.01	18
Rbm39	ENSMUSG00000027620	protein_coding	HITS-CLIP,Piranha_0.01	18
Rmrp	ENSMUSG00000115420	lincRNA	HITS-CLIP,Piranha_0.01	18
Sema6d	ENSMUSG00000027200	protein_coding	HITS-CLIP,Piranha_0.01	18
Septin7	ENSMUSG00000001833	protein_coding	HITS-CLIP,Piranha_0.01	18
Skil	ENSMUSG00000027660	protein_coding	HITS-CLIP,Piranha_0.01	18
Tec	ENSMUSG00000029217	protein_coding	HITS-CLIP,Piranha_0.01	18
Tle3	ENSMUSG00000032280	protein_coding	HITS-CLIP,Piranha_0.01	18
Ubap2	ENSMUSG00000028433	protein_coding	HITS-CLIP,Piranha_0.01	18
Ube2k	ENSMUSG00000029203	protein_coding	HITS-CLIP,Piranha_0.01	18
Vim	ENSMUSG00000026728	protein_coding	HITS-CLIP,Piranha_0.01	18
Wdr43	ENSMUSG00000041057	protein_coding	HITS-CLIP,Piranha_0.01	18
Cldnd1	ENSMUSG00000022744	protein_coding	HITS-CLIP,Piranha_0.01	17
Eif4e	ENSMUSG00000028156	protein_coding	HITS-CLIP,Piranha_0.01	17
Frmpd4	ENSMUSG00000049176	protein_coding	HITS-CLIP,Piranha_0.01	17
Gm48099	ENSMUSG00000114304	lincRNA	HITS-CLIP,Piranha_0.01	17
Nup98	ENSMUSG00000063550	protein_coding	HITS-CLIP,Piranha_0.01	17
Pim3	ENSMUSG00000035828	protein_coding	HITS-CLIP,Piranha_0.01	17
Ppp1cb	ENSMUSG00000014956	protein_coding	HITS-CLIP,Piranha_0.01	17
Prrc2a	ENSMUSG00000024393	protein_coding	HITS-CLIP,Piranha_0.01	17
Ptpn4	ENSMUSG00000026384	protein_coding	HITS-CLIP,Piranha_0.01	17
Snord80	ENSMUSG00000065735	snoRNA	HITS-CLIP,Piranha_0.01	17
St7	ENSMUSG00000029534	protein_coding	HITS-CLIP,Piranha_0.01	17
Tspan5	ENSMUSG00000028152	protein_coding	HITS-CLIP,Piranha_0.01	17
Zfp280d	ENSMUSG00000038535	protein_coding	HITS-CLIP,Piranha_0.01	17
Atp2a2	ENSMUSG00000029467	protein_coding	HITS-CLIP,Piranha_0.01	16
Atxn7l1	ENSMUSG00000020564	protein_coding	HITS-CLIP,Piranha_0.01	16
B230208H11Rik	ENSMUSG00000097378	lincRNA	HITS-CLIP,Piranha_0.01	16
Bhlhe40	ENSMUSG00000030103	protein_coding	HITS-CLIP,Piranha_0.01	16
Bsg	ENSMUSG00000023175	protein_coding	HITS-CLIP,Piranha_0.01	16
Canx	ENSMUSG00000020368	protein_coding	HITS-CLIP,Piranha_0.01	16
Capn3	ENSMUSG00000079110	protein_coding	HITS-CLIP,Piranha_0.01	16
Chd8	ENSMUSG00000053754	protein_coding	HITS-CLIP,Piranha_0.01	16
Fbxl17	ENSMUSG00000023965	protein_coding	HITS-CLIP,Piranha_0.01	16
Gm24451	ENSMUSG00000065287	snoRNA	HITS-CLIP,Piranha_0.01	16
Greb1	ENSMUSG00000036523	protein_coding	HITS-CLIP,Piranha_0.01	16
Mga	ENSMUSG00000033943	protein_coding	HITS-CLIP,Piranha_0.01	16
Mllt3	ENSMUSG00000028496	protein_coding	HITS-CLIP,Piranha_0.01	16
Naa15	ENSMUSG00000063273	protein_coding	HITS-CLIP,Piranha_0.01	16

Nfkbia	ENSMUSG00000021025	protein_coding	HITS-CLIP,Piranha_0.01	16
Pabpc1	ENSMUSG00000022283	protein_coding	HITS-CLIP,Piranha_0.01	16
Piezo2	ENSMUSG00000041482	protein_coding	HITS-CLIP,Piranha_0.01	16
Srsf2	ENSMUSG00000034120	protein_coding	HITS-CLIP,Piranha_0.01	16
St8sia3os	ENSMUSG00000086128	antisense	HITS-CLIP,Piranha_0.01	16
Stk40	ENSMUSG00000042608	protein_coding	HITS-CLIP,Piranha_0.01	16
Tnpo2	ENSMUSG00000031691	protein_coding	HITS-CLIP,Piranha_0.01	16
Vmn2r110	ENSMUSG00000091259	protein_coding	HITS-CLIP,Piranha_0.01	16
Adam5	ENSMUSG00000031554	protein_coding	HITS-CLIP,Piranha_0.01	15
Arl15	ENSMUSG00000042348	protein_coding	HITS-CLIP,Piranha_0.01	15
Atp8b4	ENSMUSG00000060131	protein_coding	HITS-CLIP,Piranha_0.01	15
Cap1	ENSMUSG00000028656	protein_coding	HITS-CLIP,Piranha_0.01	15
Ccl3	ENSMUSG00000000982	protein_coding	HITS-CLIP,Piranha_0.01	15
Ccnb1ip1	ENSMUSG00000071470	protein_coding	HITS-CLIP,Piranha_0.01	15
Chd4	ENSMUSG00000063870	protein_coding	HITS-CLIP,Piranha_0.01	15
Clec16a	ENSMUSG00000068663	protein_coding	HITS-CLIP,Piranha_0.01	15
Csk	ENSMUSG00000032312	protein_coding	HITS-CLIP,Piranha_0.01	15
Csnk1e	ENSMUSG00000022433	protein_coding	HITS-CLIP,Piranha_0.01	15
Dusp1	ENSMUSG00000024190	protein_coding	HITS-CLIP,Piranha_0.01	15
Dusp2	ENSMUSG00000027368	protein_coding	HITS-CLIP,Piranha_0.01	15
Eef1b2	ENSMUSG00000025967	protein_coding	HITS-CLIP,Piranha_0.01	15
Fmr1	ENSMUSG00000000838	protein_coding	HITS-CLIP,Piranha_0.01	15
Gls	ENSMUSG00000026103	protein_coding	HITS-CLIP,Piranha_0.01	15
Gm24044	ENSMUSG00000065041	snoRNA	HITS-CLIP,Piranha_0.01	15
Gm2682	ENSMUSG00000115681	lincRNA	HITS-CLIP,Piranha_0.01	15
Gxylt1	ENSMUSG00000036197	protein_coding	HITS-CLIP,Piranha_0.01	15
Hnrnpd	ENSMUSG00000000568	protein_coding	HITS-CLIP,Piranha_0.01	15
Hspa9	ENSMUSG00000024359	protein_coding	HITS-CLIP,Piranha_0.01	15
Ier3	ENSMUSG00000003541	protein_coding	HITS-CLIP,Piranha_0.01	15
Klhl32	ENSMUSG00000040387	protein_coding	HITS-CLIP,Piranha_0.01	15
Mir142hg	ENSMUSG00000084796	lincRNA	HITS-CLIP,Piranha_0.01	15
Mthfd2	ENSMUSG00000005667	protein_coding	HITS-CLIP,Piranha_0.01	15
Myo16	ENSMUSG00000039057	protein_coding	HITS-CLIP,Piranha_0.01	15
Naa50	ENSMUSG00000022698	protein_coding	HITS-CLIP,Piranha_0.01	15
Nop53	ENSMUSG00000041560	protein_coding	HITS-CLIP,Piranha_0.01	15
Notch1	ENSMUSG00000026923	protein_coding	HITS-CLIP,Piranha_0.01	15
Papola	ENSMUSG00000021111	protein_coding	HITS-CLIP,Piranha_0.01	15
Pde6d	ENSMUSG00000026239	protein_coding	HITS-CLIP,Piranha_0.01	15
Ppm1b	ENSMUSG00000061130	protein_coding	HITS-CLIP,Piranha_0.01	15
Sec63	ENSMUSG00000019802	protein_coding	HITS-CLIP,Piranha_0.01	15
Stambpl1	ENSMUSG00000024776	protein_coding	HITS-CLIP,Piranha_0.01	15
Tank	ENSMUSG00000064289	protein_coding	HITS-CLIP,Piranha_0.01	15
Tfdp1	ENSMUSG00000038482	protein_coding	HITS-CLIP,Piranha_0.01	15
Tgif1	ENSMUSG00000047407	protein_coding	HITS-CLIP,Piranha_0.01	15
Txnrd1	ENSMUSG00000020250	protein_coding	HITS-CLIP,Piranha_0.01	15
Akap13	ENSMUSG00000066406	protein_coding	HITS-CLIP,Piranha_0.01	14
Arhgap6	ENSMUSG00000031355	protein_coding	HITS-CLIP,Piranha_0.01	14
Ccr5	ENSMUSG00000079227	protein_coding	HITS-CLIP,Piranha_0.01	14
Cfl1	ENSMUSG00000056201	protein_coding	HITS-CLIP,Piranha_0.01	14
Dgkd	ENSMUSG00000070738	protein_coding	HITS-CLIP,Piranha_0.01	14
Dlg4	ENSMUSG00000020886	protein_coding	HITS-CLIP,Piranha_0.01	14
Kpna1	ENSMUSG00000022905	protein_coding	HITS-CLIP,Piranha_0.01	14
Magi1	ENSMUSG00000045095	protein_coding	HITS-CLIP,Piranha_0.01	14
Ncl	ENSMUSG00000026234	protein_coding	HITS-CLIP,Piranha_0.01	14
Pfkp	ENSMUSG00000021196	protein_coding	HITS-CLIP,Piranha_0.01	14
Rab8b	ENSMUSG00000036943	protein_coding	HITS-CLIP,Piranha_0.01	14
Rapgef6	ENSMUSG00000037533	protein_coding	HITS-CLIP,Piranha_0.01	14
Rcc1	ENSMUSG00000028896	protein_coding	HITS-CLIP,Piranha_0.01	14

Rnf19b	ENSMUSG00000028793	protein_coding	HITS-CLIP,Piranha_0.01	14
Scml2	ENSMUSG00000000037	protein_coding	HITS-CLIP,Piranha_0.01	14
Slc38a2	ENSMUSG00000022462	protein_coding	HITS-CLIP,Piranha_0.01	14
Slc6a6	ENSMUSG00000030096	protein_coding	HITS-CLIP,Piranha_0.01	14
Snora65	ENSMUSG00000065124	snoRNA	HITS-CLIP,Piranha_0.01	14
Snord104	ENSMUSG00000065126	snoRNA	HITS-CLIP,Piranha_0.01	14
Snord118	ENSMUSG00000064899	snoRNA	HITS-CLIP,Piranha_0.01	14
Tmtc2	ENSMUSG00000036019	protein_coding	HITS-CLIP,Piranha_0.01	14
Tomm34	ENSMUSG00000018322	protein_coding	HITS-CLIP,Piranha_0.01	14
Tshz2	ENSMUSG00000047907	protein_coding	HITS-CLIP,Piranha_0.01	14
Wipf1	ENSMUSG00000075284	protein_coding	HITS-CLIP,Piranha_0.01	14
AA474408	ENSMUSG00000073867	TEC	HITS-CLIP,Piranha_0.01	13
Ago2	ENSMUSG00000036698	protein_coding	HITS-CLIP,Piranha_0.01	13
Ankrd17	ENSMUSG00000055204	protein_coding	HITS-CLIP,Piranha_0.01	13
Arf4	ENSMUSG00000021877	protein_coding	HITS-CLIP,Piranha_0.01	13
Bcl2	ENSMUSG00000057329	protein_coding	HITS-CLIP,Piranha_0.01	13
Cdk6	ENSMUSG00000040274	protein_coding	HITS-CLIP,Piranha_0.01	13
Gm22513	ENSMUSG00000096349	snRNA	HITS-CLIP,Piranha_0.01	13
Gm23201	ENSMUSG00000077565	snoRNA	HITS-CLIP,Piranha_0.01	13
Gm25878	ENSMUSG00000077515	snoRNA	HITS-CLIP,Piranha_0.01	13
Gm25894	ENSMUSG00000065392	snoRNA	HITS-CLIP,Piranha_0.01	13
Hsp90ab1	ENSMUSG00000023944	protein_coding	HITS-CLIP,Piranha_0.01	13
Kras	ENSMUSG00000030265	protein_coding	HITS-CLIP,Piranha_0.01	13
M6pr	ENSMUSG00000007458	protein_coding	HITS-CLIP,Piranha_0.01	13
Mir16-1	ENSMUSG00000092995	miRNA	HITS-CLIP,Piranha_0.01	13
n-R5s136	ENSMUSG00000096037	rRNA	HITS-CLIP,Piranha_0.01	13
n-R5s185	ENSMUSG00000065887	rRNA	HITS-CLIP,Piranha_0.01	13
Nrxn1	ENSMUSG00000024109	protein_coding	HITS-CLIP,Piranha_0.01	13
Pbrm1	ENSMUSG00000042323	protein_coding	HITS-CLIP,Piranha_0.01	13
Plec	ENSMUSG00000022565	protein_coding	HITS-CLIP,Piranha_0.01	13
Rps3a1	ENSMUSG00000028081	protein_coding	HITS-CLIP,Piranha_0.01	13
Slc7a1	ENSMUSG00000041313	protein_coding	HITS-CLIP,Piranha_0.01	13
Snd1	ENSMUSG00000001424	protein_coding	HITS-CLIP,Piranha_0.01	13
Snord99	ENSMUSG00000080615	snoRNA	HITS-CLIP,Piranha_0.01	13
Tgfb1	ENSMUSG00000002603	protein_coding	HITS-CLIP,Piranha_0.01	13
Tnfsf11	ENSMUSG00000022015	protein_coding	HITS-CLIP,Piranha_0.01	13
Tra2b	ENSMUSG00000022858	protein_coding	HITS-CLIP,Piranha_0.01	13
Tubb5	ENSMUSG00000001525	protein_coding	HITS-CLIP,Piranha_0.01	13
Utrn	ENSMUSG00000019820	protein_coding	HITS-CLIP,Piranha_0.01	13
Zbtb20	ENSMUSG00000022708	protein_coding	HITS-CLIP,Piranha_0.01	13
Acot7	ENSMUSG00000028937	protein_coding	HITS-CLIP,Piranha_0.01	12
Adk	ENSMUSG00000039197	protein_coding	HITS-CLIP,Piranha_0.01	12
Alk	ENSMUSG00000055471	protein_coding	HITS-CLIP,Piranha_0.01	12
Arhgap15	ENSMUSG00000049744	protein_coding	HITS-CLIP,Piranha_0.01	12
Arih2	ENSMUSG00000064145	protein_coding	HITS-CLIP,Piranha_0.01	12
Atl3	ENSMUSG00000024759	protein_coding	HITS-CLIP,Piranha_0.01	12
Atp13a2	ENSMUSG00000036622	protein_coding	HITS-CLIP,Piranha_0.01	12
Atp2b4	ENSMUSG00000026463	protein_coding	HITS-CLIP,Piranha_0.01	12
Axl	ENSMUSG00000002602	protein_coding	HITS-CLIP,Piranha_0.01	12
BC031181	ENSMUSG00000036299	protein_coding	HITS-CLIP,Piranha_0.01	12
Btg1	ENSMUSG00000036478	protein_coding	HITS-CLIP,Piranha_0.01	12
Cd82	ENSMUSG00000027215	protein_coding	HITS-CLIP,Piranha_0.01	12
Cpd	ENSMUSG00000020841	protein_coding	HITS-CLIP,Piranha_0.01	12
Ctnna2	ENSMUSG00000063063	protein_coding	HITS-CLIP,Piranha_0.01	12
Dcp1a	ENSMUSG00000021962	protein_coding	HITS-CLIP,Piranha_0.01	12
Dock4	ENSMUSG00000035954	protein_coding	HITS-CLIP,Piranha_0.01	12
Foxo3	ENSMUSG00000048756	protein_coding	HITS-CLIP,Piranha_0.01	12
Gem	ENSMUSG00000028214	protein_coding	HITS-CLIP,Piranha_0.01	12

Gm22357	ENSMUSG00000064816	snoRNA	HITS-CLIP,Piranha_0.01	12
Gm22589	ENSMUSG00000075770	snoRNA	HITS-CLIP,Piranha_0.01	12
Gm23262	ENSMUSG00000088948	snoRNA	HITS-CLIP,Piranha_0.01	12
Gm23301	ENSMUSG00000064647	snoRNA	HITS-CLIP,Piranha_0.01	12
Gm23991	ENSMUSG00000080683	snoRNA	HITS-CLIP,Piranha_0.01	12
Gm24407	ENSMUSG00000094377	snRNA	HITS-CLIP,Piranha_0.01	12
Gm24455	ENSMUSG00000065282	snoRNA	HITS-CLIP,Piranha_0.01	12
Gm26202	ENSMUSG00000064844	snoRNA	HITS-CLIP,Piranha_0.01	12
Gm28638	ENSMUSG00000100197	lincRNA	HITS-CLIP,Piranha_0.01	12
Gm50452	ENSMUSG00000065160	snoRNA	HITS-CLIP,Piranha_0.01	12
Golph3	ENSMUSG00000022200	protein_coding	HITS-CLIP,Piranha_0.01	12
H2-K1	ENSMUSG00000061232	protein_coding	HITS-CLIP,Piranha_0.01	12
H4c4	ENSMUSG00000061482	protein_coding	HITS-CLIP,Piranha_0.01	12
Hdac7	ENSMUSG00000022475	protein_coding	HITS-CLIP,Piranha_0.01	12
Hnrnpf	ENSMUSG00000042079	protein_coding	HITS-CLIP,Piranha_0.01	12
Id2	ENSMUSG00000020644	protein_coding	HITS-CLIP,Piranha_0.01	12
Ipo8	ENSMUSG00000040029	protein_coding	HITS-CLIP,Piranha_0.01	12
Iqca	ENSMUSG00000026301	protein_coding	HITS-CLIP,Piranha_0.01	12
Larp4	ENSMUSG00000023025	protein_coding	HITS-CLIP,Piranha_0.01	12
Mob1a	ENSMUSG00000043131	protein_coding	HITS-CLIP,Piranha_0.01	12
Mpp6	ENSMUSG00000038388	protein_coding	HITS-CLIP,Piranha_0.01	12
n-R5s64	ENSMUSG00000075973	rRNA	HITS-CLIP,Piranha_0.01	12
Nedd9	ENSMUSG00000021365	protein_coding	HITS-CLIP,Piranha_0.01	12
Pfdn4	ENSMUSG00000052033	protein_coding	HITS-CLIP,Piranha_0.01	12
Plod2	ENSMUSG00000032374	protein_coding	HITS-CLIP,Piranha_0.01	12
Ppp1r15a	ENSMUSG00000040435	protein_coding	HITS-CLIP,Piranha_0.01	12
Prrx1	ENSMUSG00000026586	protein_coding	HITS-CLIP,Piranha_0.01	12
Rab6a	ENSMUSG00000030704	protein_coding	HITS-CLIP,Piranha_0.01	12
Rap1b	ENSMUSG00000052681	protein_coding	HITS-CLIP,Piranha_0.01	12
Rhot1	ENSMUSG00000017686	protein_coding	HITS-CLIP,Piranha_0.01	12
Rpl4	ENSMUSG00000032399	protein_coding	HITS-CLIP,Piranha_0.01	12
Smarcc1	ENSMUSG00000032481	protein_coding	HITS-CLIP,Piranha_0.01	12
Smox	ENSMUSG00000027333	protein_coding	HITS-CLIP,Piranha_0.01	12
Snord12	ENSMUSG00000077698	snoRNA	HITS-CLIP,Piranha_0.01	12
Snord57	ENSMUSG00000065272	snoRNA	HITS-CLIP,Piranha_0.01	12
Snord72	ENSMUSG00000077737	snoRNA	HITS-CLIP,Piranha_0.01	12
Spock1	ENSMUSG00000056222	protein_coding	HITS-CLIP,CTK	12
Srsf9	ENSMUSG00000029538	protein_coding	HITS-CLIP,Piranha_0.01	12
Tagln2	ENSMUSG00000026547	protein_coding	HITS-CLIP,Piranha_0.01	12
Tango6	ENSMUSG00000041949	protein_coding	HITS-CLIP,Piranha_0.01	12
Tbrg4	ENSMUSG00000000384	protein_coding	HITS-CLIP,Piranha_0.01	12
Tmcc1	ENSMUSG00000030126	protein_coding	HITS-CLIP,Piranha_0.01	12
Tmf1	ENSMUSG00000030059	protein_coding	HITS-CLIP,Piranha_0.01	12
Tnfaip8	ENSMUSG00000062210	protein_coding	HITS-CLIP,Piranha_0.01	12
Trps1	ENSMUSG00000038679	protein_coding	HITS-CLIP,Piranha_0.01	12
Ubqln1	ENSMUSG00000005312	protein_coding	HITS-CLIP,Piranha_0.01	12
Wdr92	ENSMUSG00000078970	protein_coding	HITS-CLIP,Piranha_0.01	12
Ywhaz	ENSMUSG00000022285	protein_coding	HITS-CLIP,Piranha_0.01	12
Zfand6	ENSMUSG00000030629	protein_coding	HITS-CLIP,Piranha_0.01	12
Arhgef3	ENSMUSG00000021895	protein_coding	HITS-CLIP,Piranha_0.01	11
Arid4b	ENSMUSG00000039219	protein_coding	HITS-CLIP,Piranha_0.01	11
Atp2b1	ENSMUSG00000019943	protein_coding	HITS-CLIP,Piranha_0.01	11
Cpsf7	ENSMUSG00000034820	protein_coding	HITS-CLIP,Piranha_0.01	11
Ddx17	ENSMUSG00000055065	protein_coding	HITS-CLIP,Piranha_0.01	11
Dock2	ENSMUSG00000020143	protein_coding	HITS-CLIP,Piranha_0.01	11
Dusp5	ENSMUSG00000034765	protein_coding	HITS-CLIP,Piranha_0.01	11
Eef1e1	ENSMUSG00000001707	protein_coding	HITS-CLIP,Piranha_0.01	11
Foxo1	ENSMUSG00000044167	protein_coding	HITS-CLIP,Piranha_0.01	11

Gm23969	ENSMUSG00000065258	snoRNA	HITS-CLIP,Piranha_0.01	11
Gm24134	ENSMUSG00000065295	snoRNA	HITS-CLIP,Piranha_0.01	11
Gm24265	ENSMUSG00000096243	snRNA	HITS-CLIP,Piranha_0.01	11
Gm24452	ENSMUSG00000065281	snoRNA	HITS-CLIP,Piranha_0.01	11
Gm24453	ENSMUSG00000065280	snoRNA	HITS-CLIP,Piranha_0.01	11
Gm25091	ENSMUSG00000077185	snoRNA	HITS-CLIP,Piranha_0.01	11
Gm25789	ENSMUSG00000065228	snoRNA	HITS-CLIP,Piranha_0.01	11
Gm25855	ENSMUSG00000064721	snoRNA	HITS-CLIP,Piranha_0.01	11
Itgb2	ENSMUSG00000000290	protein_coding	HITS-CLIP,Piranha_0.01	11
Luc7l3	ENSMUSG00000020863	protein_coding	HITS-CLIP,Piranha_0.01	11
Mir3535	ENSMUSG00000104627	miRNA	HITS-CLIP,Piranha_0.01	11
Numb	ENSMUSG00000021224	protein_coding	HITS-CLIP,Piranha_0.01	11
Otud4	ENSMUSG00000036990	protein_coding	HITS-CLIP,Piranha_0.01	11
Oxr1	ENSMUSG00000022307	protein_coding	HITS-CLIP,Piranha_0.01	11
Pdgfb	ENSMUSG00000000489	protein_coding	HITS-CLIP,Piranha_0.01	11
Pgs1	ENSMUSG00000017715	protein_coding	HITS-CLIP,Piranha_0.01	11
Ptprj	ENSMUSG00000025314	protein_coding	HITS-CLIP,Piranha_0.01	11
R3hdm2	ENSMUSG00000025404	protein_coding	HITS-CLIP,Piranha_0.01	11
Rnu12	ENSMUSG00000065176	snRNA	HITS-CLIP,Piranha_0.01	11
Sfpq	ENSMUSG00000028820	protein_coding	HITS-CLIP,Piranha_0.01	11
Slc38a1	ENSMUSG00000023169	protein_coding	HITS-CLIP,Piranha_0.01	11
Snhg5	ENSMUSG00000097195	processed_transcri	HITS-CLIP,Piranha_0.01	11
Snora28	ENSMUSG00000064493	snoRNA	HITS-CLIP,Piranha_0.01	11
Snord2	ENSMUSG00000088524	snoRNA	HITS-CLIP,Piranha_0.01	11
Snord22	ENSMUSG00000065087	snoRNA	HITS-CLIP,Piranha_0.01	11
Snord34	ENSMUSG00000065878	snoRNA	HITS-CLIP,Piranha_0.01	11
Snord82	ENSMUSG00000064823	snoRNA	HITS-CLIP,Piranha_0.01	11
Snord96a	ENSMUSG00000064725	snoRNA	HITS-CLIP,Piranha_0.01	11
Srrm2	ENSMUSG00000039218	protein_coding	HITS-CLIP,Piranha_0.01	11
Taok3	ENSMUSG00000061288	protein_coding	HITS-CLIP,Piranha_0.01	11
Tprkb	ENSMUSG00000054226	protein_coding	HITS-CLIP,Piranha_0.01	11
Vps54	ENSMUSG00000020128	protein_coding	HITS-CLIP,Piranha_0.01	11
Zfp207	ENSMUSG00000017421	protein_coding	HITS-CLIP,Piranha_0.01	11
Zfp536	ENSMUSG00000043456	protein_coding	HITS-CLIP,Piranha_0.01	11
Abl2	ENSMUSG00000026596	protein_coding	HITS-CLIP,Piranha_0.01	10
Adcy3	ENSMUSG00000020654	protein_coding	HITS-CLIP,Piranha_0.01	10
Anp32a	ENSMUSG00000032249	protein_coding	HITS-CLIP,Piranha_0.01	10
Arhgef2	ENSMUSG00000028059	protein_coding	HITS-CLIP,Piranha_0.01	10
AU020206	ENSMUSG00000097415	lincRNA	HITS-CLIP,Piranha_0.01	10
Baz2a	ENSMUSG00000040054	protein_coding	HITS-CLIP,Piranha_0.01	10
Bcl11b	ENSMUSG00000048251	protein_coding	HITS-CLIP,Piranha_0.01	10
Cacna1b	ENSMUSG00000004113	protein_coding	HITS-CLIP,Piranha_0.01	10
Calcrl	ENSMUSG00000059588	protein_coding	HITS-CLIP,Piranha_0.01	10
Calm1	ENSMUSG00000001175	protein_coding	HITS-CLIP,Piranha_0.01	10
Cdkl5	ENSMUSG00000031292	protein_coding	HITS-CLIP,Piranha_0.01	10
Cdyl	ENSMUSG00000059288	protein_coding	HITS-CLIP,Piranha_0.01	10
Cxcl2	ENSMUSG00000058427	protein_coding	HITS-CLIP,Piranha_0.01	10
Ddx39b	ENSMUSG00000019432	protein_coding	HITS-CLIP,Piranha_0.01	10
Dnmt3b	ENSMUSG00000027478	protein_coding	HITS-CLIP,Piranha_0.01	10
Eea1	ENSMUSG00000036499	protein_coding	HITS-CLIP,Piranha_0.01	10
Evl	ENSMUSG00000021262	protein_coding	HITS-CLIP,Piranha_0.01	10
Ezh2	ENSMUSG00000029687	protein_coding	HITS-CLIP,Piranha_0.01	10
Fasn	ENSMUSG00000025153	protein_coding	HITS-CLIP,Piranha_0.01	10
Fhit	ENSMUSG00000060579	protein_coding	HITS-CLIP,Piranha_0.01	10
Fmn1	ENSMUSG00000044042	protein_coding	HITS-CLIP,Piranha_0.01	10
Foxp4	ENSMUSG00000023991	protein_coding	HITS-CLIP,Piranha_0.01	10
Gja1	ENSMUSG00000050953	protein_coding	HITS-CLIP,Piranha_0.01	10
Gm22744	ENSMUSG00000065378	snoRNA	HITS-CLIP,Piranha_0.01	10

Gm23143	ENSMUSG00000094405	snRNA	HITS-CLIP,Piranha_0.01	10
Gm23287	ENSMUSG00000096280	snRNA	HITS-CLIP,Piranha_0.01	10
Gm24826	ENSMUSG00000065629	snoRNA	HITS-CLIP,Piranha_0.01	10
Gm26448	ENSMUSG00000064380	snoRNA	HITS-CLIP,Piranha_0.01	10
Gm43430	ENSMUSG00000106149	TEC	HITS-CLIP,Piranha_0.01	10
Hccs	ENSMUSG00000031352	protein_coding	HITS-CLIP,Piranha_0.01	10
Il17a	ENSMUSG00000025929	protein_coding	HITS-CLIP,Piranha_0.01	10
Inpp4a	ENSMUSG00000026113	protein_coding	HITS-CLIP,Piranha_0.01	10
Ipo7	ENSMUSG00000066232	protein_coding	HITS-CLIP,Piranha_0.01	10
Klf2	ENSMUSG00000055148	protein_coding	HITS-CLIP,Piranha_0.01	10
Macroh2a1	ENSMUSG00000015937	protein_coding	HITS-CLIP,Piranha_0.01	10
Manf	ENSMUSG00000032575	protein_coding	HITS-CLIP,Piranha_0.01	10
Mapkapk3	ENSMUSG00000032577	protein_coding	HITS-CLIP,Piranha_0.01	10
Mbtd1	ENSMUSG00000059474	protein_coding	HITS-CLIP,Piranha_0.01	10
Mdh2	ENSMUSG00000019179	protein_coding	HITS-CLIP,Piranha_0.01	10
Myo7a	ENSMUSG00000030761	protein_coding	HITS-CLIP,Piranha_0.01	10
Opa3	ENSMUSG00000052214	protein_coding	HITS-CLIP,Piranha_0.01	10
Pcbp2	ENSMUSG00000056851	protein_coding	HITS-CLIP,Piranha_0.01	10
Phactr3	ENSMUSG00000027525	protein_coding	HITS-CLIP,Piranha_0.01	10
Phf20	ENSMUSG00000038116	protein_coding	HITS-CLIP,Piranha_0.01	10
Raly	ENSMUSG00000027593	protein_coding	HITS-CLIP,Piranha_0.01	10
Rcc2	ENSMUSG00000040945	protein_coding	HITS-CLIP,Piranha_0.01	10
Snhg16	ENSMUSG00000020812	antisense	HITS-CLIP,Piranha_0.01	10
Snhg3	ENSMUSG00000085241	processed_transcri	HITS-CLIP,Piranha_0.01	10
Snord49a	ENSMUSG00000065734	snoRNA	HITS-CLIP,Piranha_0.01	10
Snord49b	ENSMUSG00000064437	snoRNA	HITS-CLIP,Piranha_0.01	10
Snord60	ENSMUSG00000064768	snoRNA	HITS-CLIP,Piranha_0.01	10
Sub1	ENSMUSG00000022205	protein_coding	HITS-CLIP,Piranha_0.01	10
Tet3	ENSMUSG00000034832	protein_coding	HITS-CLIP,Piranha_0.01	10
Tpp2	ENSMUSG00000041763	protein_coding	HITS-CLIP,Piranha_0.01	10
Ube2d3	ENSMUSG00000078578	protein_coding	HITS-CLIP,Piranha_0.01	10
Ulk4	ENSMUSG00000040936	protein_coding	HITS-CLIP,Piranha_0.01	10
Wac	ENSMUSG00000024283	protein_coding	HITS-CLIP,Piranha_0.01	10
Yrdc	ENSMUSG00000028889	protein_coding	HITS-CLIP,Piranha_0.01	10
Zfp185	ENSMUSG00000031351	protein_coding	HITS-CLIP,Piranha_0.01	10
2410006H16Rik	ENSMUSG00000086841	processed_transcri	HITS-CLIP,Piranha_0.01	9
2900060B14Rik	ENSMUSG00000107722	antisense	HITS-CLIP,Piranha_0.01	9
Anapc11	ENSMUSG00000025135	protein_coding	HITS-CLIP,Piranha_0.01	9
Anp32e	ENSMUSG00000015749	protein_coding	HITS-CLIP,Piranha_0.01	9
Arid5b	ENSMUSG00000019947	protein_coding	HITS-CLIP,Piranha_0.01	9
Atp5b	ENSMUSG00000025393	protein_coding	HITS-CLIP,Piranha_0.01	9
Bcl2l1	ENSMUSG00000007659	protein_coding	HITS-CLIP,Piranha_0.01	9
C2cd5	ENSMUSG00000030279	protein_coding	HITS-CLIP,Piranha_0.01	9
Csnk1a1	ENSMUSG00000024576	protein_coding	HITS-CLIP,Piranha_0.01	9
Ctdspl2	ENSMUSG00000033411	protein_coding	HITS-CLIP,Piranha_0.01	9
Dip2c	ENSMUSG00000048264	protein_coding	HITS-CLIP,Piranha_0.01	9
Dusp4	ENSMUSG00000031530	protein_coding	HITS-CLIP,Piranha_0.01	9
Ehmt1	ENSMUSG00000036893	protein_coding	HITS-CLIP,Piranha_0.01	9
Eif4g1	ENSMUSG00000045983	protein_coding	HITS-CLIP,Piranha_0.01	9
Elmo1	ENSMUSG00000041112	protein_coding	HITS-CLIP,Piranha_0.01	9
Enc1	ENSMUSG00000041773	protein_coding	HITS-CLIP,Piranha_0.01	9
Erbin	ENSMUSG00000021709	protein_coding	HITS-CLIP,Piranha_0.01	9
Ero1l	ENSMUSG00000021831	protein_coding	HITS-CLIP,Piranha_0.01	9
Gak	ENSMUSG00000062234	protein_coding	HITS-CLIP,Piranha_0.01	9
Gm23246	ENSMUSG00000065305	snoRNA	HITS-CLIP,Piranha_0.01	9
Gm24299	ENSMUSG00000088929	snoRNA	HITS-CLIP,Piranha_0.01	9
Gm25813	ENSMUSG00000064682	snRNA	HITS-CLIP,Piranha_0.01	9
Gm26224	ENSMUSG00000064440	snoRNA	HITS-CLIP,Piranha_0.01	9

Gm26287	ENSMUSG00000089512	snoRNA	HITS-CLIP,Piranha_0.01	9
Gm48099	ENSMUSG00000114304	lincRNA	HITS-CLIP,CTK	9
Gm50449	ENSMUSG00000093245	snoRNA	HITS-CLIP,Piranha_0.01	9
Grcc10	ENSMUSG00000072772	protein_coding	HITS-CLIP,Piranha_0.01	9
Huwe1	ENSMUSG00000025261	protein_coding	HITS-CLIP,Piranha_0.01	9
Il1rapl1	ENSMUSG00000052372	protein_coding	HITS-CLIP,Piranha_0.01	9
Ino80d	ENSMUSG00000040865	protein_coding	HITS-CLIP,Piranha_0.01	9
Iqschfp	ENSMUSG00000102422	protein_coding	HITS-CLIP,Piranha_0.01	9
Itpkb	ENSMUSG00000038855	protein_coding	HITS-CLIP,Piranha_0.01	9
Jmjd1c	ENSMUSG00000037876	protein_coding	HITS-CLIP,Piranha_0.01	9
Klf6	ENSMUSG00000000078	protein_coding	HITS-CLIP,Piranha_0.01	9
Lilr4b	ENSMUSG00000112023	protein_coding	HITS-CLIP,Piranha_0.01	9
Limd1	ENSMUSG00000025239	protein_coding	HITS-CLIP,Piranha_0.01	9
Lipo4	ENSMUSG00000079344	protein_coding	HITS-CLIP,Piranha_0.01	9
Maff	ENSMUSG00000042622	protein_coding	HITS-CLIP,Piranha_0.01	9
Meis2	ENSMUSG00000027210	protein_coding	HITS-CLIP,Piranha_0.01	9
Mir26a-1	ENSMUSG00000065513	miRNA	HITS-CLIP,Piranha_0.01	9
Mir677	ENSMUSG00000104755	miRNA	HITS-CLIP,Piranha_0.01	9
Mrps6	ENSMUSG00000039680	protein_coding	HITS-CLIP,Piranha_0.01	9
n-R5s194	ENSMUSG00000094830	rRNA	HITS-CLIP,Piranha_0.01	9
n-R5s205	ENSMUSG00000065083	rRNA	HITS-CLIP,Piranha_0.01	9
Pan3	ENSMUSG00000029647	protein_coding	HITS-CLIP,Piranha_0.01	9
Pdzrn3	ENSMUSG00000035357	protein_coding	HITS-CLIP,Piranha_0.01	9
Plxdc2	ENSMUSG00000026748	protein_coding	HITS-CLIP,Piranha_0.01	9
Psmd11	ENSMUSG00000017428	protein_coding	HITS-CLIP,Piranha_0.01	9
Retreg1	ENSMUSG00000022270	protein_coding	HITS-CLIP,Piranha_0.01	9
Rgs16	ENSMUSG00000026475	protein_coding	HITS-CLIP,Piranha_0.01	9
Rps15a	ENSMUSG00000008683	protein_coding	HITS-CLIP,Piranha_0.01	9
Septin6	ENSMUSG00000050379	protein_coding	HITS-CLIP,Piranha_0.01	9
Septin9	ENSMUSG00000059248	protein_coding	HITS-CLIP,Piranha_0.01	9
Snora15	ENSMUSG00000093413	snoRNA	HITS-CLIP,Piranha_0.01	9
Snora3	ENSMUSG00000065016	snoRNA	HITS-CLIP,Piranha_0.01	9
Snora62	ENSMUSG00000064925	snoRNA	HITS-CLIP,Piranha_0.01	9
Snord16a	ENSMUSG00000094411	snoRNA	HITS-CLIP,Piranha_0.01	9
Snord21	ENSMUSG00000064453	snoRNA	HITS-CLIP,Piranha_0.01	9
Snord65	ENSMUSG00000077457	snoRNA	HITS-CLIP,Piranha_0.01	9
Snord68	ENSMUSG00000064450	snoRNA	HITS-CLIP,Piranha_0.01	9
Ssb	ENSMUSG00000068882	protein_coding	HITS-CLIP,Piranha_0.01	9
Syncrip	ENSMUSG00000032423	protein_coding	HITS-CLIP,Piranha_0.01	9
Zwint	ENSMUSG00000019923	protein_coding	HITS-CLIP,Piranha_0.01	9
1810055G02Rik	ENSMUSG00000035372	protein_coding	HITS-CLIP,Piranha_0.01	8
2310057M21Rik	ENSMUSG00000040177	protein_coding	HITS-CLIP,Piranha_0.01	8
Adcy6	ENSMUSG00000022994	protein_coding	HITS-CLIP,Piranha_0.01	8
Ahnak	ENSMUSG00000069833	protein_coding	HITS-CLIP,Piranha_0.01	8
Anxa2	ENSMUSG00000032231	protein_coding	HITS-CLIP,Piranha_0.01	8
Arpp21	ENSMUSG00000032503	protein_coding	HITS-CLIP,Piranha_0.01	8
Atxn2l	ENSMUSG00000032637	protein_coding	HITS-CLIP,Piranha_0.01	8
B3gnt2	ENSMUSG00000051650	protein_coding	HITS-CLIP,Piranha_0.01	8
Basp1	ENSMUSG00000045763	protein_coding	HITS-CLIP,Piranha_0.01	8
Bdp1	ENSMUSG00000049658	protein_coding	HITS-CLIP,Piranha_0.01	8
Blm	ENSMUSG00000030528	protein_coding	HITS-CLIP,Piranha_0.01	8
Bud23	ENSMUSG00000005378	protein_coding	HITS-CLIP,Piranha_0.01	8
Cbfb	ENSMUSG00000031885	protein_coding	HITS-CLIP,Piranha_0.01	8
Ccar1	ENSMUSG00000020074	protein_coding	HITS-CLIP,Piranha_0.01	8
Cd200	ENSMUSG00000022661	protein_coding	HITS-CLIP,Piranha_0.01	8
Cep128	ENSMUSG00000061533	protein_coding	HITS-CLIP,Piranha_0.01	8
Clcn3	ENSMUSG00000004319	protein_coding	HITS-CLIP,Piranha_0.01	8
Cox17	ENSMUSG00000046516	protein_coding	HITS-CLIP,Piranha_0.01	8

Crhbp	ENSMUSG00000021680	protein_coding	HITS-CLIP,Piranha_0.01	8
Dab1	ENSMUSG00000028519	protein_coding	HITS-CLIP,Piranha_0.01	8
Dgkz	ENSMUSG00000040479	protein_coding	HITS-CLIP,Piranha_0.01	8
Eif2ak1	ENSMUSG00000029613	protein_coding	HITS-CLIP,Piranha_0.01	8
Elk4	ENSMUSG00000026436	protein_coding	HITS-CLIP,Piranha_0.01	8
Fbln1	ENSMUSG00000006369	protein_coding	HITS-CLIP,Piranha_0.01	8
Fbxw11	ENSMUSG00000020271	protein_coding	HITS-CLIP,Piranha_0.01	8
Fndc3a	ENSMUSG00000033487	protein_coding	HITS-CLIP,Piranha_0.01	8
Fubp1	ENSMUSG00000028034	protein_coding	HITS-CLIP,Piranha_0.01	8
Gm22455	ENSMUSG00000064514	snoRNA	HITS-CLIP,Piranha_0.01	8
Gm22618	ENSMUSG00000095857	snRNA	HITS-CLIP,Piranha_0.01	8
Gm22680	ENSMUSG00000065782	snoRNA	HITS-CLIP,Piranha_0.01	8
Gm22973	ENSMUSG00000065232	snRNA	HITS-CLIP,Piranha_0.01	8
Gm23650	ENSMUSG00000065289	snoRNA	HITS-CLIP,Piranha_0.01	8
Gm25099	ENSMUSG00000095676	snRNA	HITS-CLIP,Piranha_0.01	8
Gm25360	ENSMUSG00000094655	snRNA	HITS-CLIP,Piranha_0.01	8
Gm25970	ENSMUSG00000087790	snoRNA	HITS-CLIP,Piranha_0.01	8
Gm26205	ENSMUSG00000064841	snoRNA	HITS-CLIP,Piranha_0.01	8
Gon4l	ENSMUSG00000054199	protein_coding	HITS-CLIP,Piranha_0.01	8
Hnrnpa0	ENSMUSG00000007836	protein_coding	HITS-CLIP,Piranha_0.01	8
Hnrnpa1	ENSMUSG00000046434	protein_coding	HITS-CLIP,Piranha_0.01	8
Hs6st2	ENSMUSG00000062184	protein_coding	HITS-CLIP,Piranha_0.01	8
Igfbp2	ENSMUSG00000039323	protein_coding	HITS-CLIP,Piranha_0.01	8
Il13	ENSMUSG00000020383	protein_coding	HITS-CLIP,Piranha_0.01	8
Il24	ENSMUSG00000026420	protein_coding	HITS-CLIP,Piranha_0.01	8
Lrrc72	ENSMUSG00000020545	protein_coding	HITS-CLIP,Piranha_0.01	8
Mafk	ENSMUSG00000018143	protein_coding	HITS-CLIP,Piranha_0.01	8
Map4k4	ENSMUSG00000026074	protein_coding	HITS-CLIP,Piranha_0.01	8
Marchf7	ENSMUSG00000026977	protein_coding	HITS-CLIP,Piranha_0.01	8
Mier3	ENSMUSG00000032727	protein_coding	HITS-CLIP,Piranha_0.01	8
Mir1949	ENSMUSG00000088059	snoRNA	HITS-CLIP,Piranha_0.01	8
Mir26b	ENSMUSG00000065468	miRNA	HITS-CLIP,Piranha_0.01	8
Mir6516	ENSMUSG00000104740	miRNA	HITS-CLIP,Piranha_0.01	8
Mir670hg	ENSMUSG00000075020	lincRNA	HITS-CLIP,Piranha_0.01	8
Morc2a	ENSMUSG00000034543	protein_coding	HITS-CLIP,Piranha_0.01	8
Myl12b	ENSMUSG00000034868	protein_coding	HITS-CLIP,Piranha_0.01	8
n-R5s193	ENSMUSG00000064798	rRNA	HITS-CLIP,Piranha_0.01	8
Naa20	ENSMUSG00000002728	protein_coding	HITS-CLIP,Piranha_0.01	8
Ncam1	ENSMUSG00000039542	protein_coding	HITS-CLIP,Piranha_0.01	8
Neat1	ENSMUSG00000092274	lincRNA	HITS-CLIP,Piranha_0.01	8
Ogdh	ENSMUSG00000020456	protein_coding	HITS-CLIP,Piranha_0.01	8
P2ry2	ENSMUSG00000032860	protein_coding	HITS-CLIP,Piranha_0.01	8
Pank3	ENSMUSG00000018846	protein_coding	HITS-CLIP,Piranha_0.01	8
Pcnp	ENSMUSG00000071533	protein_coding	HITS-CLIP,Piranha_0.01	8
Pde1c	ENSMUSG00000004347	protein_coding	HITS-CLIP,Piranha_0.01	8
Pias2	ENSMUSG00000025423	protein_coding	HITS-CLIP,Piranha_0.01	8
Plscr1	ENSMUSG00000032369	protein_coding	HITS-CLIP,Piranha_0.01	8
Ptprz1	ENSMUSG00000068748	protein_coding	HITS-CLIP,Piranha_0.01	8
Rab1a	ENSMUSG00000020149	protein_coding	HITS-CLIP,Piranha_0.01	8
Rabep1	ENSMUSG00000020817	protein_coding	HITS-CLIP,Piranha_0.01	8
Rabgap1l	ENSMUSG00000026721	protein_coding	HITS-CLIP,Piranha_0.01	8
Rn7sk	ENSMUSG00000065037	misc_RNA	HITS-CLIP,Piranha_0.01	8
Rpl39	ENSMUSG00000079641	protein_coding	HITS-CLIP,Piranha_0.01	8
Rps5	ENSMUSG00000012848	protein_coding	HITS-CLIP,Piranha_0.01	8
Sell	ENSMUSG00000026581	protein_coding	HITS-CLIP,Piranha_0.01	8
Serpine1	ENSMUSG00000037411	protein_coding	HITS-CLIP,Piranha_0.01	8
Sertad2	ENSMUSG00000049800	protein_coding	HITS-CLIP,Piranha_0.01	8
Sh3rf1	ENSMUSG00000031642	protein_coding	HITS-CLIP,Piranha_0.01	8

Snhg8	ENSMUSG00000104960	processed_transcri	HITS-CLIP,Piranha_0.01	8
Snord14d	ENSMUSG00000095118	snoRNA	HITS-CLIP,Piranha_0.01	8
Snord37	ENSMUSG00000064441	snoRNA	HITS-CLIP,Piranha_0.01	8
Tars2	ENSMUSG00000028107	protein_coding	HITS-CLIP,Piranha_0.01	8
Thy1	ENSMUSG00000032011	protein_coding	HITS-CLIP,Piranha_0.01	8
Tipin	ENSMUSG00000032397	protein_coding	HITS-CLIP,Piranha_0.01	8
Trip12	ENSMUSG00000026219	protein_coding	HITS-CLIP,Piranha_0.01	8
Ttc14	ENSMUSG00000027677	protein_coding	HITS-CLIP,Piranha_0.01	8
Ugcg	ENSMUSG00000028381	protein_coding	HITS-CLIP,Piranha_0.01	8
Xiap	ENSMUSG00000025860	protein_coding	HITS-CLIP,Piranha_0.01	8
Ywhae	ENSMUSG00000020849	protein_coding	HITS-CLIP,Piranha_0.01	8
Zfp263	ENSMUSG00000022529	protein_coding	HITS-CLIP,Piranha_0.01	8
Zfp791	ENSMUSG00000074194	protein_coding	HITS-CLIP,Piranha_0.01	8
A430093F15Rik	ENSMUSG00000067577	processed_transcri	HITS-CLIP,Piranha_0.01	7
Abi2	ENSMUSG00000026782	protein_coding	HITS-CLIP,Piranha_0.01	7
Actr2	ENSMUSG00000020152	protein_coding	HITS-CLIP,Piranha_0.01	7
Adamts6	ENSMUSG00000046169	protein_coding	HITS-CLIP,Piranha_0.01	7
Ankrd6	ENSMUSG00000040183	protein_coding	HITS-CLIP,Piranha_0.01	7
Atp11b	ENSMUSG00000037400	protein_coding	HITS-CLIP,Piranha_0.01	7
Atp9b	ENSMUSG00000024566	protein_coding	HITS-CLIP,Piranha_0.01	7
Baz2b	ENSMUSG00000026987	protein_coding	HITS-CLIP,Piranha_0.01	7
Bcas3	ENSMUSG00000059439	protein_coding	HITS-CLIP,Piranha_0.01	7
Bcat2	ENSMUSG00000030826	protein_coding	HITS-CLIP,Piranha_0.01	7
Cab39	ENSMUSG00000036707	protein_coding	HITS-CLIP,Piranha_0.01	7
Ccdc88c	ENSMUSG00000021182	protein_coding	HITS-CLIP,Piranha_0.01	7
Chd1	ENSMUSG00000023852	protein_coding	HITS-CLIP,Piranha_0.01	7
Chst11	ENSMUSG00000034612	protein_coding	HITS-CLIP,Piranha_0.01	7
Chsy1	ENSMUSG00000032640	protein_coding	HITS-CLIP,Piranha_0.01	7
Dmtf1	ENSMUSG00000042508	protein_coding	HITS-CLIP,Piranha_0.01	7
Dnajc1	ENSMUSG00000026740	protein_coding	HITS-CLIP,Piranha_0.01	7
Esr1	ENSMUSG00000019768	protein_coding	HITS-CLIP,Piranha_0.01	7
Fars2	ENSMUSG00000021420	protein_coding	HITS-CLIP,Piranha_0.01	7
Flt1	ENSMUSG00000029648	protein_coding	HITS-CLIP,Piranha_0.01	7
Foxn3	ENSMUSG00000033713	protein_coding	HITS-CLIP,Piranha_0.01	7
G3bp1	ENSMUSG00000018583	protein_coding	HITS-CLIP,Piranha_0.01	7
G3bp2	ENSMUSG00000029405	protein_coding	HITS-CLIP,Piranha_0.01	7
Git2	ENSMUSG00000041890	protein_coding	HITS-CLIP,Piranha_0.01	7
Gm15564	ENSMUSG00000086324	antisense	HITS-CLIP,Piranha_0.01	7
Gm22489	ENSMUSG00000080463	snoRNA	HITS-CLIP,Piranha_0.01	7
Gm22884	ENSMUSG00000065061	snoRNA	HITS-CLIP,Piranha_0.01	7
Gm22984	ENSMUSG00000084700	snRNA	HITS-CLIP,Piranha_0.01	7
Gm23136	ENSMUSG00000095868	snoRNA	HITS-CLIP,Piranha_0.01	7
Gm23152	ENSMUSG00000093063	rRNA	HITS-CLIP,Piranha_0.01	7
Gm24573	ENSMUSG00000093240	rRNA	HITS-CLIP,Piranha_0.01	7
Gm25128	ENSMUSG00000065273	snoRNA	HITS-CLIP,Piranha_0.01	7
Gm25162	ENSMUSG00000089531	snoRNA	HITS-CLIP,Piranha_0.01	7
Gm25245	ENSMUSG00000096895	snRNA	HITS-CLIP,Piranha_0.01	7
Gm25791	ENSMUSG00000065226	snoRNA	HITS-CLIP,Piranha_0.01	7
Gm26130	ENSMUSG00000065105	snoRNA	HITS-CLIP,Piranha_0.01	7
Gm45223	ENSMUSG00000109429	TEC	HITS-CLIP,Piranha_0.01	7
Gm47946	ENSMUSG00000112884	lincRNA	HITS-CLIP,Piranha_0.01	7
Gm49692	ENSMUSG00000116736	processed_transcri	HITS-CLIP,Piranha_0.01	7
H3c3	ENSMUSG00000069310	protein_coding	HITS-CLIP,Piranha_0.01	7
Hagh	ENSMUSG00000024158	protein_coding	HITS-CLIP,Piranha_0.01	7
Ifrd1	ENSMUSG00000001627	protein_coding	HITS-CLIP,Piranha_0.01	7
Ikzf3	ENSMUSG00000018168	protein_coding	HITS-CLIP,Piranha_0.01	7
Iqcj	ENSMUSG00000051777	protein_coding	HITS-CLIP,Piranha_0.01	7
Irf8	ENSMUSG00000041515	protein_coding	HITS-CLIP,Piranha_0.01	7

Itgal	ENSMUSG00000030830	protein_coding	HITS-CLIP,Piranha_0.01	7
Lck	ENSMUSG00000000409	protein_coding	HITS-CLIP,Piranha_0.01	7
Mak	ENSMUSG00000021363	protein_coding	HITS-CLIP,Piranha_0.01	7
Mbp	ENSMUSG00000041607	protein_coding	HITS-CLIP,Piranha_0.01	7
Mef2d	ENSMUSG00000001419	protein_coding	HITS-CLIP,Piranha_0.01	7
Mir19b-1	ENSMUSG00000076256	miRNA	HITS-CLIP,Piranha_0.01	7
Mir30d	ENSMUSG00000065437	miRNA	HITS-CLIP,Piranha_0.01	7
Myh9	ENSMUSG00000022443	protein_coding	HITS-CLIP,Piranha_0.01	7
n-R5s151	ENSMUSG00000065893	rRNA	HITS-CLIP,Piranha_0.01	7
n-R5s2	ENSMUSG00000075918	rRNA	HITS-CLIP,Piranha_0.01	7
n-R5s40	ENSMUSG00000064409	rRNA	HITS-CLIP,Piranha_0.01	7
Nmd3	ENSMUSG00000027787	protein_coding	HITS-CLIP,Piranha_0.01	7
Osbpl8	ENSMUSG00000020189	protein_coding	HITS-CLIP,Piranha_0.01	7
Phlda1	ENSMUSG00000020205	protein_coding	HITS-CLIP,Piranha_0.01	7
Pkn2	ENSMUSG00000004591	protein_coding	HITS-CLIP,Piranha_0.01	7
Purb	ENSMUSG00000094483	protein_coding	HITS-CLIP,Piranha_0.01	7
Ralgapa1	ENSMUSG00000021027	protein_coding	HITS-CLIP,Piranha_0.01	7
Rsrc1	ENSMUSG00000034544	protein_coding	HITS-CLIP,Piranha_0.01	7
Rwdd4a	ENSMUSG00000031568	protein_coding	HITS-CLIP,Piranha_0.01	7
Sdhaf4	ENSMUSG00000026154	protein_coding	HITS-CLIP,Piranha_0.01	7
Skap1	ENSMUSG00000057058	protein_coding	HITS-CLIP,Piranha_0.01	7
Slc25a45	ENSMUSG00000024818	protein_coding	HITS-CLIP,Piranha_0.01	7
Snora52	ENSMUSG00000064666	snoRNA	HITS-CLIP,Piranha_0.01	7
Snora61	ENSMUSG00000064949	snoRNA	HITS-CLIP,Piranha_0.01	7
Snora64	ENSMUSG00000077709	snoRNA	HITS-CLIP,Piranha_0.01	7
Snord14e	ENSMUSG00000064791	snoRNA	HITS-CLIP,Piranha_0.01	7
Srp14	ENSMUSG00000009549	protein_coding	HITS-CLIP,Piranha_0.01	7
Tcn2	ENSMUSG00000020432	protein_coding	HITS-CLIP,CTK	7
Thrap3	ENSMUSG00000043962	protein_coding	HITS-CLIP,Piranha_0.01	7
Tlcd4	ENSMUSG00000028132	protein_coding	HITS-CLIP,Piranha_0.01	7
Tmem164	ENSMUSG00000047045	protein_coding	HITS-CLIP,Piranha_0.01	7
Tnpo3	ENSMUSG00000012535	protein_coding	HITS-CLIP,Piranha_0.01	7
Traf3	ENSMUSG00000021277	protein_coding	HITS-CLIP,Piranha_0.01	7
U2af1	ENSMUSG00000061613	protein_coding	HITS-CLIP,Piranha_0.01	7
Ubap2l	ENSMUSG00000042520	protein_coding	HITS-CLIP,Piranha_0.01	7
Usp15	ENSMUSG00000020124	protein_coding	HITS-CLIP,Piranha_0.01	7
Vav1	ENSMUSG00000034116	protein_coding	HITS-CLIP,Piranha_0.01	7
Ywhag	ENSMUSG00000051391	protein_coding	HITS-CLIP,Piranha_0.01	7
Zfp513	ENSMUSG00000043059	protein_coding	HITS-CLIP,Piranha_0.01	7
4930594M22Rik	ENSMUSG00000075463	processed_transcri	HITS-CLIP,Piranha_0.01	6
Abca12	ENSMUSG00000050296	protein_coding	HITS-CLIP,Piranha_0.01	6
Adam10	ENSMUSG00000054693	protein_coding	HITS-CLIP,CTK	6
Adamts12	ENSMUSG00000047497	protein_coding	HITS-CLIP,Piranha_0.01	6
Ankrd44	ENSMUSG00000052331	protein_coding	HITS-CLIP,Piranha_0.01	6
Arid3a	ENSMUSG00000019564	protein_coding	HITS-CLIP,Piranha_0.01	6
Arih1	ENSMUSG00000025234	protein_coding	HITS-CLIP,Piranha_0.01	6
Arpc2	ENSMUSG00000006304	protein_coding	HITS-CLIP,Piranha_0.01	6
Arpc4	ENSMUSG00000079426	protein_coding	HITS-CLIP,Piranha_0.01	6
Atg4b	ENSMUSG00000026280	protein_coding	HITS-CLIP,Piranha_0.01	6
Atp10a	ENSMUSG00000025324	protein_coding	HITS-CLIP,Piranha_0.01	6
Atp5a1	ENSMUSG00000025428	protein_coding	HITS-CLIP,Piranha_0.01	6
Azin1	ENSMUSG00000037458	protein_coding	HITS-CLIP,Piranha_0.01	6
Brd4	ENSMUSG00000024002	protein_coding	HITS-CLIP,Piranha_0.01	6
C2cd2	ENSMUSG00000045975	protein_coding	HITS-CLIP,Piranha_0.01	6
Cbl	ENSMUSG00000034342	protein_coding	HITS-CLIP,Piranha_0.01	6
Cd274	ENSMUSG00000016496	protein_coding	HITS-CLIP,Piranha_0.01	6
Cd3e	ENSMUSG00000032093	protein_coding	HITS-CLIP,Piranha_0.01	6
Cdc42	ENSMUSG00000006699	protein_coding	HITS-CLIP,Piranha_0.01	6

Cdh18	ENSMUSG00000040420	protein_coding	HITS-CLIP,Piranha_0.01	6
Chuk	ENSMUSG00000025199	protein_coding	HITS-CLIP,Piranha_0.01	6
Clhc1	ENSMUSG00000020461	protein_coding	HITS-CLIP,Piranha_0.01	6
Cp	ENSMUSG00000003617	protein_coding	HITS-CLIP,Piranha_0.01	6
Cwf19l1	ENSMUSG00000025200	protein_coding	HITS-CLIP,Piranha_0.01	6
Cxcr4	ENSMUSG00000045382	protein_coding	HITS-CLIP,Piranha_0.01	6
Cyfip1	ENSMUSG00000030447	protein_coding	HITS-CLIP,Piranha_0.01	6
Dcaf5	ENSMUSG00000049106	protein_coding	HITS-CLIP,Piranha_0.01	6
Dcun1d3	ENSMUSG00000048787	protein_coding	HITS-CLIP,Piranha_0.01	6
Dda1	ENSMUSG00000074247	protein_coding	HITS-CLIP,Piranha_0.01	6
Dgka	ENSMUSG00000025357	protein_coding	HITS-CLIP,Piranha_0.01	6
Egr3	ENSMUSG00000033730	protein_coding	HITS-CLIP,Piranha_0.01	6
F3	ENSMUSG00000028128	protein_coding	HITS-CLIP,Piranha_0.01	6
Fam135a	ENSMUSG00000026153	protein_coding	HITS-CLIP,Piranha_0.01	6
Fam53c	ENSMUSG00000034300	protein_coding	HITS-CLIP,Piranha_0.01	6
Far1	ENSMUSG00000030759	protein_coding	HITS-CLIP,Piranha_0.01	6
Fip1l1	ENSMUSG00000029227	protein_coding	HITS-CLIP,Piranha_0.01	6
Fkbp1a	ENSMUSG00000032966	protein_coding	HITS-CLIP,Piranha_0.01	6
Fosl2	ENSMUSG00000029135	protein_coding	HITS-CLIP,Piranha_0.01	6
Gm20594	ENSMUSG00000096887	protein_coding	HITS-CLIP,Piranha_0.01	6
Gm22571	ENSMUSG00000064918	snoRNA	HITS-CLIP,Piranha_0.01	6
Gm22638	ENSMUSG00000094848	snRNA	HITS-CLIP,Piranha_0.01	6
Gm22685	ENSMUSG00000064782	snRNA	HITS-CLIP,Piranha_0.01	6
Gm22748	ENSMUSG00000064427	snoRNA	HITS-CLIP,Piranha_0.01	6
Gm23008	ENSMUSG00000089571	snoRNA	HITS-CLIP,Piranha_0.01	6
Gm23344	ENSMUSG00000065752	snoRNA	HITS-CLIP,Piranha_0.01	6
Gm23639	ENSMUSG00000092819	snoRNA	HITS-CLIP,Piranha_0.01	6
Gm24067	ENSMUSG00000077702	snoRNA	HITS-CLIP,Piranha_0.01	6
Gm24611	ENSMUSG00000065206	snRNA	HITS-CLIP,Piranha_0.01	6
Gm24704	ENSMUSG00000070227	snRNA	HITS-CLIP,Piranha_0.01	6
Gm25131	ENSMUSG00000064587	snRNA	HITS-CLIP,Piranha_0.01	6
Gm25704	ENSMUSG00000065050	snRNA	HITS-CLIP,Piranha_0.01	6
Gm26244	ENSMUSG00000095616	snRNA	HITS-CLIP,Piranha_0.01	6
Gm45221	ENSMUSG00000109005	TEC	HITS-CLIP,Piranha_0.01	6
Gm49339	ENSMUSG00000062593	protein_coding	HITS-CLIP,Piranha_0.01	6
Gtdc1	ENSMUSG00000036890	protein_coding	HITS-CLIP,Piranha_0.01	6
Gzmb	ENSMUSG00000015437	protein_coding	HITS-CLIP,Piranha_0.01	6
H2-Q7	ENSMUSG00000060550	protein_coding	HITS-CLIP,Piranha_0.01	6
Hbegf	ENSMUSG00000024486	protein_coding	HITS-CLIP,Piranha_0.01	6
Hip1	ENSMUSG00000039959	protein_coding	HITS-CLIP,Piranha_0.01	6
Hipk1	ENSMUSG00000008730	protein_coding	HITS-CLIP,Piranha_0.01	6
Hnrnpa3	ENSMUSG00000059005	protein_coding	HITS-CLIP,Piranha_0.01	6
Hnrnph1	ENSMUSG00000007850	protein_coding	HITS-CLIP,Piranha_0.01	6
Hsph1	ENSMUSG00000029657	protein_coding	HITS-CLIP,Piranha_0.01	6
Hyou1	ENSMUSG00000032115	protein_coding	HITS-CLIP,Piranha_0.01	6
Il15	ENSMUSG00000031712	protein_coding	HITS-CLIP,Piranha_0.01	6
Il21r	ENSMUSG00000030745	protein_coding	HITS-CLIP,Piranha_0.01	6
Il31	ENSMUSG00000029437	protein_coding	HITS-CLIP,Piranha_0.01	6
Iqck	ENSMUSG00000073856	protein_coding	HITS-CLIP,Piranha_0.01	6
Irf2	ENSMUSG00000031627	protein_coding	HITS-CLIP,Piranha_0.01	6
Kcmf1	ENSMUSG00000055239	protein_coding	HITS-CLIP,Piranha_0.01	6
Kcnip4	ENSMUSG00000029088	protein_coding	HITS-CLIP,Piranha_0.01	6
Kctd10	ENSMUSG00000001098	protein_coding	HITS-CLIP,Piranha_0.01	6
Kctd9	ENSMUSG00000034327	protein_coding	HITS-CLIP,Piranha_0.01	6
Khdrbs2	ENSMUSG00000026058	protein_coding	HITS-CLIP,Piranha_0.01	6
Kmt2b	ENSMUSG00000006307	protein_coding	HITS-CLIP,Piranha_0.01	6
Lama3	ENSMUSG00000024421	protein_coding	HITS-CLIP,Piranha_0.01	6
Lilrb4a	ENSMUSG00000112148	protein_coding	HITS-CLIP,Piranha_0.01	6

Lrriq3	ENSMUSG00000028182	protein_coding	HITS-CLIP,Piranha_0.01	6
Marchf11	ENSMUSG00000022269	protein_coding	HITS-CLIP,Piranha_0.01	6
Mdm4	ENSMUSG00000054387	protein_coding	HITS-CLIP,Piranha_0.01	6
Mindy3	ENSMUSG00000026767	protein_coding	HITS-CLIP,Piranha_0.01	6
Mir15a	ENSMUSG00000093007	miRNA	HITS-CLIP,Piranha_0.01	6
Mir17hg	ENSMUSG00000089726	lincRNA	HITS-CLIP,Piranha_0.01	6
Mir21a	ENSMUSG00000065455	miRNA	HITS-CLIP,Piranha_0.01	6
Mir29a	ENSMUSG00000065610	miRNA	HITS-CLIP,Piranha_0.01	6
Mllt6	ENSMUSG00000038437	protein_coding	HITS-CLIP,Piranha_0.01	6
Mthfsl	ENSMUSG00000079427	protein_coding	HITS-CLIP,Piranha_0.01	6
n-R5s127	ENSMUSG00000093960	rRNA	HITS-CLIP,Piranha_0.01	6
Nlrc5	ENSMUSG00000074151	protein_coding	HITS-CLIP,Piranha_0.01	6
Nploc4	ENSMUSG00000039703	protein_coding	HITS-CLIP,Piranha_0.01	6
Odf2	ENSMUSG00000026790	protein_coding	HITS-CLIP,Piranha_0.01	6
Olfr56	ENSMUSG00000040328	protein_coding	HITS-CLIP,Piranha_0.01	6
Pitpnb	ENSMUSG00000050017	protein_coding	HITS-CLIP,Piranha_0.01	6
Prelid3a	ENSMUSG00000024530	protein_coding	HITS-CLIP,Piranha_0.01	6
Prpf6	ENSMUSG00000002455	protein_coding	HITS-CLIP,Piranha_0.01	6
Psmd14	ENSMUSG00000026914	protein_coding	HITS-CLIP,Piranha_0.01	6
Ptprs	ENSMUSG00000013236	protein_coding	HITS-CLIP,Piranha_0.01	6
Rap1gds1	ENSMUSG00000028149	protein_coding	HITS-CLIP,Piranha_0.01	6
Rara	ENSMUSG00000037992	protein_coding	HITS-CLIP,Piranha_0.01	6
Rel	ENSMUSG00000020275	protein_coding	HITS-CLIP,Piranha_0.01	6
Rlim	ENSMUSG00000056537	protein_coding	HITS-CLIP,Piranha_0.01	6
Rnf145	ENSMUSG00000019189	protein_coding	HITS-CLIP,Piranha_0.01	6
Rny3	ENSMUSG00000064945	misc_RNA	HITS-CLIP,Piranha_0.01	6
Rpl17	ENSMUSG00000062328	protein_coding	HITS-CLIP,Piranha_0.01	6
Senp2	ENSMUSG00000022855	protein_coding	HITS-CLIP,Piranha_0.01	6
Serpinb5	ENSMUSG00000067006	protein_coding	HITS-CLIP,Piranha_0.01	6
Set	ENSMUSG00000054766	protein_coding	HITS-CLIP,Piranha_0.01	6
Sil1	ENSMUSG00000024357	protein_coding	HITS-CLIP,Piranha_0.01	6
Ski	ENSMUSG00000029050	protein_coding	HITS-CLIP,Piranha_0.01	6
Slamf1	ENSMUSG00000015316	protein_coding	HITS-CLIP,Piranha_0.01	6
Slamf7	ENSMUSG00000038179	protein_coding	HITS-CLIP,Piranha_0.01	6
Slc12a8	ENSMUSG00000035506	protein_coding	HITS-CLIP,Piranha_0.01	6
Slc25a17	ENSMUSG00000022404	protein_coding	HITS-CLIP,Piranha_0.01	6
Slc27a1	ENSMUSG00000031808	protein_coding	HITS-CLIP,Piranha_0.01	6
Slc2a1	ENSMUSG00000028645	protein_coding	HITS-CLIP,Piranha_0.01	6
Slc5a3	ENSMUSG00000089774	protein_coding	HITS-CLIP,Piranha_0.01	6
Smad7	ENSMUSG00000025880	protein_coding	HITS-CLIP,Piranha_0.01	6
Smarcad1	ENSMUSG00000029920	protein_coding	HITS-CLIP,Piranha_0.01	6
Snhg10	ENSMUSG00000113722	lincRNA	HITS-CLIP,Piranha_0.01	6
Snora23	ENSMUSG00000064451	snoRNA	HITS-CLIP,Piranha_0.01	6
Snora41	ENSMUSG00000064602	snoRNA	HITS-CLIP,Piranha_0.01	6
Snora81	ENSMUSG00000087935	snoRNA	HITS-CLIP,Piranha_0.01	6
Snora9	ENSMUSG00000064513	snoRNA	HITS-CLIP,Piranha_0.01	6
Snord11	ENSMUSG00000089093	snoRNA	HITS-CLIP,Piranha_0.01	6
Snord17	ENSMUSG00000077714	snoRNA	HITS-CLIP,Piranha_0.01	6
Snord47	ENSMUSG00000064968	snoRNA	HITS-CLIP,Piranha_0.01	6
Snord59a	ENSMUSG00000064778	snoRNA	HITS-CLIP,Piranha_0.01	6
Snord66	ENSMUSG00000077239	snoRNA	HITS-CLIP,Piranha_0.01	6
Snord70	ENSMUSG00000077345	snoRNA	HITS-CLIP,Piranha_0.01	6
Ssr2	ENSMUSG00000041355	protein_coding	HITS-CLIP,Piranha_0.01	6
St3gal1	ENSMUSG00000013846	protein_coding	HITS-CLIP,Piranha_0.01	6
Stx6	ENSMUSG00000026470	protein_coding	HITS-CLIP,Piranha_0.01	6
Tab2	ENSMUSG00000015755	protein_coding	HITS-CLIP,Piranha_0.01	6
Tbl1x	ENSMUSG00000025246	protein_coding	HITS-CLIP,Piranha_0.01	6
Tent5a	ENSMUSG00000032265	protein_coding	HITS-CLIP,Piranha_0.01	6

Tgoln1	ENSMUSG00000056429	protein_coding	HITS-CLIP,Piranha_0.01	6
Tnrc6b	ENSMUSG00000047888	protein_coding	HITS-CLIP,Piranha_0.01	6
Trim2	ENSMUSG00000027993	protein_coding	HITS-CLIP,Piranha_0.01	6
Uap1	ENSMUSG00000026670	protein_coding	HITS-CLIP,Piranha_0.01	6
Uhmk1	ENSMUSG00000026667	protein_coding	HITS-CLIP,Piranha_0.01	6
Vsir	ENSMUSG00000020101	protein_coding	HITS-CLIP,Piranha_0.01	6
Wdr59	ENSMUSG00000031959	protein_coding	HITS-CLIP,Piranha_0.01	6
Zfyve27	ENSMUSG00000018820	protein_coding	HITS-CLIP,Piranha_0.01	6
Zmynd11	ENSMUSG00000021156	protein_coding	HITS-CLIP,Piranha_0.01	6
1700021P04Rik	ENSMUSG00000096796	lincRNA	HITS-CLIP,Piranha_0.01	5
Actr3	ENSMUSG00000026341	protein_coding	HITS-CLIP,Piranha_0.01	5
AF357399	ENSMUSG00000077711	snoRNA	HITS-CLIP,Piranha_0.01	5
Aig1	ENSMUSG00000019806	protein_coding	HITS-CLIP,Piranha_0.01	5
Alkal1	ENSMUSG00000087247	protein_coding	HITS-CLIP,Piranha_0.01	5
Arf6	ENSMUSG00000044147	protein_coding	HITS-CLIP,Piranha_0.01	5
Asxl2	ENSMUSG00000037486	protein_coding	HITS-CLIP,Piranha_0.01	5
Atp1a1	ENSMUSG00000033161	protein_coding	HITS-CLIP,Piranha_0.01	5
Atxn1	ENSMUSG00000046876	protein_coding	HITS-CLIP,Piranha_0.01	5
Carm1	ENSMUSG00000032185	protein_coding	HITS-CLIP,Piranha_0.01	5
Cdk19	ENSMUSG00000038481	protein_coding	HITS-CLIP,Piranha_0.01	5
Cept1	ENSMUSG00000040774	protein_coding	HITS-CLIP,Piranha_0.01	5
Chmp4b	ENSMUSG00000038467	protein_coding	HITS-CLIP,Piranha_0.01	5
Col25a1	ENSMUSG00000058897	protein_coding	HITS-CLIP,Piranha_0.01	5
Csf2	ENSMUSG00000018916	protein_coding	HITS-CLIP,Piranha_0.01	5
Ddx3x	ENSMUSG00000000787	protein_coding	HITS-CLIP,Piranha_0.01	5
Dennd1b	ENSMUSG00000056268	protein_coding	HITS-CLIP,Piranha_0.01	5
Dnajc5	ENSMUSG00000000826	protein_coding	HITS-CLIP,Piranha_0.01	5
Ehmt2	ENSMUSG00000013787	protein_coding	HITS-CLIP,Piranha_0.01	5
Elovl6	ENSMUSG00000041220	protein_coding	HITS-CLIP,Piranha_0.01	5
Enox1	ENSMUSG00000022012	protein_coding	HITS-CLIP,Piranha_0.01	5
Farp1	ENSMUSG00000025555	protein_coding	HITS-CLIP,Piranha_0.01	5
Fxr2	ENSMUSG00000018765	protein_coding	HITS-CLIP,Piranha_0.01	5
Gbp2	ENSMUSG00000028270	protein_coding	HITS-CLIP,Piranha_0.01	5
Gm22042	ENSMUSG00000064923	snRNA	HITS-CLIP,Piranha_0.01	5
Gm22220	ENSMUSG00000064605	snRNA	HITS-CLIP,Piranha_0.01	5
Gm22265	ENSMUSG00000094131	snRNA	HITS-CLIP,Piranha_0.01	5
Gm22270	ENSMUSG00000077222	snoRNA	HITS-CLIP,Piranha_0.01	5
Gm22307	ENSMUSG00000096126	snRNA	HITS-CLIP,Piranha_0.01	5
Gm22620	ENSMUSG00000064634	snoRNA	HITS-CLIP,Piranha_0.01	5
Gm22628	ENSMUSG00000095584	snRNA	HITS-CLIP,Piranha_0.01	5
Gm22806	ENSMUSG00000064672	snoRNA	HITS-CLIP,Piranha_0.01	5
Gm22879	ENSMUSG00000064829	snoRNA	HITS-CLIP,Piranha_0.01	5
Gm22888	ENSMUSG00000089265	snoRNA	HITS-CLIP,Piranha_0.01	5
Gm23151	ENSMUSG00000094875	snRNA	HITS-CLIP,Piranha_0.01	5
Gm23245	ENSMUSG00000065304	snoRNA	HITS-CLIP,Piranha_0.01	5
Gm23686	ENSMUSG00000096391	snRNA	HITS-CLIP,Piranha_0.01	5
Gm24261	ENSMUSG00000065678	snRNA	HITS-CLIP,Piranha_0.01	5
Gm24888	ENSMUSG00000077575	snoRNA	HITS-CLIP,Piranha_0.01	5
Gm25117	ENSMUSG00000087819	snoRNA	HITS-CLIP,Piranha_0.01	5
Gm25506	ENSMUSG00000077527	snoRNA	HITS-CLIP,Piranha_0.01	5
Gm25939	ENSMUSG00000093843	snRNA	HITS-CLIP,Piranha_0.01	5
Gm26064	ENSMUSG00000065315	snRNA	HITS-CLIP,Piranha_0.01	5
Gm26072	ENSMUSG00000094803	snRNA	HITS-CLIP,Piranha_0.01	5
Gm26127	ENSMUSG00000065127	snRNA	HITS-CLIP,Piranha_0.01	5
Gm26247	ENSMUSG00000080352	snoRNA	HITS-CLIP,Piranha_0.01	5
Gm26293	ENSMUSG00000077358	snoRNA	HITS-CLIP,Piranha_0.01	5
Gm42997	ENSMUSG00000105789	lincRNA	HITS-CLIP,Piranha_0.01	5
Gm47284	ENSMUSG00000105232	miRNA	HITS-CLIP,Piranha_0.01	5

Gpr174	ENSMUSG00000073008	protein_coding	HITS-CLIP,Piranha_0.01	5
Gys2	ENSMUSG00000030244	protein_coding	HITS-CLIP,Piranha_0.01	5
Hmgcr	ENSMUSG00000021670	protein_coding	HITS-CLIP,Piranha_0.01	5
Igsf8	ENSMUSG00000038034	protein_coding	HITS-CLIP,Piranha_0.01	5
Ipcef1	ENSMUSG00000064065	protein_coding	HITS-CLIP,Piranha_0.01	5
Iqgap1	ENSMUSG00000030536	protein_coding	HITS-CLIP,Piranha_0.01	5
Irak2	ENSMUSG00000060477	protein_coding	HITS-CLIP,Piranha_0.01	5
Jak1	ENSMUSG00000028530	protein_coding	HITS-CLIP,Piranha_0.01	5
Lama2	ENSMUSG00000019899	protein_coding	HITS-CLIP,Piranha_0.01	5
Lncpint	ENSMUSG00000044471	lincRNA	HITS-CLIP,Piranha_0.01	5
Mak16	ENSMUSG00000031578	protein_coding	HITS-CLIP,Piranha_0.01	5
Map4	ENSMUSG00000032479	protein_coding	HITS-CLIP,Piranha_0.01	5
Mdn1	ENSMUSG00000058006	protein_coding	HITS-CLIP,Piranha_0.01	5
Mir155	ENSMUSG00000065397	miRNA	HITS-CLIP,Piranha_0.01	5
Mir3068	ENSMUSG00000105428	miRNA	HITS-CLIP,Piranha_0.01	5
Mir6240	ENSMUSG00000098343	miRNA	HITS-CLIP,Piranha_0.01	5
Mllt10	ENSMUSG00000026743	protein_coding	HITS-CLIP,Piranha_0.01	5
Ms4a4b	ENSMUSG00000056290	protein_coding	HITS-CLIP,Piranha_0.01	5
Mt2	ENSMUSG00000031762	protein_coding	HITS-CLIP,Piranha_0.01	5
Myo1c	ENSMUSG00000017774	protein_coding	HITS-CLIP,Piranha_0.01	5
Myo9b	ENSMUSG00000004677	protein_coding	HITS-CLIP,Piranha_0.01	5
n-R5s115	ENSMUSG00000095915	rRNA	HITS-CLIP,Piranha_0.01	5
n-R5s152	ENSMUSG00000070150	rRNA	HITS-CLIP,Piranha_0.01	5
n-R5s93	ENSMUSG00000096219	rRNA	HITS-CLIP,Piranha_0.01	5
Nox4	ENSMUSG00000030562	protein_coding	HITS-CLIP,CTK	5
Nr3c1	ENSMUSG00000024431	protein_coding	HITS-CLIP,Piranha_0.01	5
Ofcc1	ENSMUSG00000047094	protein_coding	HITS-CLIP,Piranha_0.01	5
Papss2	ENSMUSG00000024899	protein_coding	HITS-CLIP,Piranha_0.01	5
Pcbp1	ENSMUSG00000051695	protein_coding	HITS-CLIP,Piranha_0.01	5
Phf6	ENSMUSG00000025626	protein_coding	HITS-CLIP,Piranha_0.01	5
Pisd	ENSMUSG00000023452	protein_coding	HITS-CLIP,Piranha_0.01	5
Polr3c	ENSMUSG00000028099	protein_coding	HITS-CLIP,Piranha_0.01	5
Ppp6c	ENSMUSG00000026753	protein_coding	HITS-CLIP,Piranha_0.01	5
Ptbp2	ENSMUSG00000028134	protein_coding	HITS-CLIP,Piranha_0.01	5
Ptgs2	ENSMUSG00000032487	protein_coding	HITS-CLIP,Piranha_0.01	5
Ptp4a2	ENSMUSG00000028788	protein_coding	HITS-CLIP,Piranha_0.01	5
Rab7	ENSMUSG00000079477	protein_coding	HITS-CLIP,Piranha_0.01	5
Reps1	ENSMUSG00000019854	protein_coding	HITS-CLIP,Piranha_0.01	5
Retreg3	ENSMUSG00000017802	protein_coding	HITS-CLIP,Piranha_0.01	5
Rnu5g	ENSMUSG00000095892	snRNA	HITS-CLIP,Piranha_0.01	5
Rtn4	ENSMUSG00000020458	protein_coding	HITS-CLIP,Piranha_0.01	5
Ryr3	ENSMUSG00000057378	protein_coding	HITS-CLIP,CTK	5
Sardhos	ENSMUSG00000091192	antisense	HITS-CLIP,Piranha_0.01	5
Sbno1	ENSMUSG00000038095	protein_coding	HITS-CLIP,Piranha_0.01	5
Scarna10	ENSMUSG00000089617	snoRNA	HITS-CLIP,Piranha_0.01	5
Senp3	ENSMUSG00000005204	protein_coding	HITS-CLIP,Piranha_0.01	5
Sh3glb1	ENSMUSG00000037062	protein_coding	HITS-CLIP,Piranha_0.01	5
Slfn2	ENSMUSG00000072620	protein_coding	HITS-CLIP,Piranha_0.01	5
Snhg17	ENSMUSG00000085385	processed_transcri	HITS-CLIP,Piranha_0.01	5
Snora70	ENSMUSG00000064981	snoRNA	HITS-CLIP,Piranha_0.01	5
Snora73b	ENSMUSG00000065353	snoRNA	HITS-CLIP,Piranha_0.01	5
Snord1a	ENSMUSG00000065094	snoRNA	HITS-CLIP,Piranha_0.01	5
Snord42a	ENSMUSG00000064540	snoRNA	HITS-CLIP,Piranha_0.01	5
Snord67	ENSMUSG00000077221	snoRNA	HITS-CLIP,Piranha_0.01	5
Socs3	ENSMUSG00000053113	protein_coding	HITS-CLIP,Piranha_0.01	5
Son	ENSMUSG00000022961	protein_coding	HITS-CLIP,Piranha_0.01	5
Srfbp1	ENSMUSG00000024528	protein_coding	HITS-CLIP,Piranha_0.01	5
Srsf10	ENSMUSG00000028676	protein_coding	HITS-CLIP,Piranha_0.01	5

Ss18	ENSMUSG00000037013	protein_coding	HITS-CLIP,Piranha_0.01	5
Stat5b	ENSMUSG00000020919	protein_coding	HITS-CLIP,Piranha_0.01	5
Szrd1	ENSMUSG00000040842	protein_coding	HITS-CLIP,Piranha_0.01	5
Tardbp	ENSMUSG00000041459	protein_coding	HITS-CLIP,Piranha_0.01	5
Tcerg1	ENSMUSG00000024498	protein_coding	HITS-CLIP,Piranha_0.01	5
Tcf20	ENSMUSG00000041852	protein_coding	HITS-CLIP,Piranha_0.01	5
Tlk1	ENSMUSG00000041997	protein_coding	HITS-CLIP,Piranha_0.01	5
Traf6	ENSMUSG00000027164	protein_coding	HITS-CLIP,Piranha_0.01	5
Trpv1	ENSMUSG00000005952	protein_coding	HITS-CLIP,Piranha_0.01	5
Uck2	ENSMUSG00000026558	protein_coding	HITS-CLIP,Piranha_0.01	5
Uvrag	ENSMUSG00000035354	protein_coding	HITS-CLIP,Piranha_0.01	5
Zc3h14	ENSMUSG00000021012	protein_coding	HITS-CLIP,Piranha_0.01	5
Zfp142	ENSMUSG00000026135	protein_coding	HITS-CLIP,Piranha_0.01	5
Zzz3	ENSMUSG00000039068	protein_coding	HITS-CLIP,Piranha_0.01	5
A930007I19Rik	ENSMUSG00000097855	antisense	HITS-CLIP,Piranha_0.01	4
Abcc1	ENSMUSG00000023088	protein_coding	HITS-CLIP,Piranha_0.01	4
Abce1	ENSMUSG00000058355	protein_coding	HITS-CLIP,Piranha_0.01	4
Adipor2	ENSMUSG00000030168	protein_coding	HITS-CLIP,Piranha_0.01	4
Agap1	ENSMUSG00000055013	protein_coding	HITS-CLIP,Piranha_0.01	4
AL935121.1	ENSMUSG00000118623	protein_coding	HITS-CLIP,Piranha_0.01	4
Ankrd11	ENSMUSG00000035569	protein_coding	HITS-CLIP,Piranha_0.01	4
Anxa6	ENSMUSG00000018340	protein_coding	HITS-CLIP,Piranha_0.01	4
Arf1	ENSMUSG00000048076	protein_coding	HITS-CLIP,Piranha_0.01	4
Arhgap30	ENSMUSG00000048865	protein_coding	HITS-CLIP,Piranha_0.01	4
Arl8a	ENSMUSG00000026426	protein_coding	HITS-CLIP,Piranha_0.01	4
Atp11a	ENSMUSG00000031441	protein_coding	HITS-CLIP,Piranha_0.01	4
Atxn7	ENSMUSG00000021738	protein_coding	HITS-CLIP,Piranha_0.01	4
Bax	ENSMUSG00000003873	protein_coding	HITS-CLIP,Piranha_0.01	4
Bcl2l11	ENSMUSG00000027381	protein_coding	HITS-CLIP,Piranha_0.01	4
Birc3	ENSMUSG00000032000	protein_coding	HITS-CLIP,Piranha_0.01	4
Calu	ENSMUSG00000029767	protein_coding	HITS-CLIP,Piranha_0.01	4
Cbx3	ENSMUSG00000029836	protein_coding	HITS-CLIP,Piranha_0.01	4
Ccdc148	ENSMUSG00000036641	protein_coding	HITS-CLIP,Piranha_0.01	4
Cct2	ENSMUSG00000034024	protein_coding	HITS-CLIP,Piranha_0.01	4
Cdca4	ENSMUSG00000047832	protein_coding	HITS-CLIP,Piranha_0.01	4
Cemip2	ENSMUSG00000024754	protein_coding	HITS-CLIP,Piranha_0.01	4
Cetn2	ENSMUSG00000031347	protein_coding	HITS-CLIP,Piranha_0.01	4
Chtf8	ENSMUSG00000046691	protein_coding	HITS-CLIP,Piranha_0.01	4
Cited2	ENSMUSG00000039910	protein_coding	HITS-CLIP,Piranha_0.01	4
Cltc	ENSMUSG00000047126	protein_coding	HITS-CLIP,Piranha_0.01	4
Cmip	ENSMUSG00000034390	protein_coding	HITS-CLIP,Piranha_0.01	4
Cnot2	ENSMUSG00000020166	protein_coding	HITS-CLIP,Piranha_0.01	4
Cog5	ENSMUSG00000035933	protein_coding	HITS-CLIP,Piranha_0.01	4
Commd1b	ENSMUSG00000098650	protein_coding	HITS-CLIP,Piranha_0.01	4
Cops4	ENSMUSG00000035297	protein_coding	HITS-CLIP,Piranha_0.01	4
Cradd	ENSMUSG00000045867	protein_coding	HITS-CLIP,Piranha_0.01	4
Crybg1	ENSMUSG00000019866	protein_coding	HITS-CLIP,Piranha_0.01	4
Csnk2a1	ENSMUSG00000074698	protein_coding	HITS-CLIP,Piranha_0.01	4
Ctla4	ENSMUSG00000026011	protein_coding	HITS-CLIP,Piranha_0.01	4
Dcaf6	ENSMUSG00000026571	protein_coding	HITS-CLIP,Piranha_0.01	4
Dctn3	ENSMUSG00000028447	protein_coding	HITS-CLIP,Piranha_0.01	4
Dctn6	ENSMUSG00000031516	protein_coding	HITS-CLIP,Piranha_0.01	4
Ddx6	ENSMUSG00000032097	protein_coding	HITS-CLIP,Piranha_0.01	4
Dennd1a	ENSMUSG00000035392	protein_coding	HITS-CLIP,Piranha_0.01	4
Dhx15	ENSMUSG00000029169	protein_coding	HITS-CLIP,Piranha_0.01	4
Dnajb11	ENSMUSG00000004460	protein_coding	HITS-CLIP,Piranha_0.01	4
Dnajc5b	ENSMUSG00000027606	protein_coding	HITS-CLIP,Piranha_0.01	4
Dusp11	ENSMUSG00000030002	protein_coding	HITS-CLIP,Piranha_0.01	4

Dync2h1	ENSMUSG00000047193	protein_coding	HITS-CLIP,Piranha_0.01	4
Eaf1	ENSMUSG00000021890	protein_coding	HITS-CLIP,Piranha_0.01	4
Edem1	ENSMUSG00000030104	protein_coding	HITS-CLIP,Piranha_0.01	4
Egr1	ENSMUSG00000038418	protein_coding	HITS-CLIP,Piranha_0.01	4
Eif4b	ENSMUSG00000058655	protein_coding	HITS-CLIP,Piranha_0.01	4
Elovl5	ENSMUSG00000032349	protein_coding	HITS-CLIP,Piranha_0.01	4
Epb41l4a	ENSMUSG00000024376	protein_coding	HITS-CLIP,Piranha_0.01	4
Epha6	ENSMUSG00000055540	protein_coding	HITS-CLIP,Piranha_0.01	4
Erap1	ENSMUSG00000021583	protein_coding	HITS-CLIP,Piranha_0.01	4
Etf1	ENSMUSG00000024360	protein_coding	HITS-CLIP,Piranha_0.01	4
F730043M19Rik	ENSMUSG00000052125	bidirectional_prom	HITS-CLIP,Piranha_0.01	4
Fam120a	ENSMUSG00000038014	protein_coding	HITS-CLIP,Piranha_0.01	4
Fos	ENSMUSG00000021250	protein_coding	HITS-CLIP,Piranha_0.01	4
Foxj3	ENSMUSG00000032998	protein_coding	HITS-CLIP,Piranha_0.01	4
Gabrb1	ENSMUSG00000029212	protein_coding	HITS-CLIP,Piranha_0.01	4
Gatad2a	ENSMUSG00000036180	protein_coding	HITS-CLIP,Piranha_0.01	4
Glis3	ENSMUSG00000052942	protein_coding	HITS-CLIP,Piranha_0.01	4
Gm15832	ENSMUSG00000085894	processed_transcri	HITS-CLIP,Piranha_0.01	4
Gm21972	ENSMUSG00000094638	protein_coding	HITS-CLIP,Piranha_0.01	4
Gm22169	ENSMUSG00000096639	snRNA	HITS-CLIP,Piranha_0.01	4
Gm22423	ENSMUSG00000064795	snRNA	HITS-CLIP,Piranha_0.01	4
Gm22710	ENSMUSG00000080542	snRNA	HITS-CLIP,Piranha_0.01	4
Gm22865	ENSMUSG00000065883	snoRNA	HITS-CLIP,Piranha_0.01	4
Gm23002	ENSMUSG00000094026	snRNA	HITS-CLIP,Piranha_0.01	4
Gm23095	ENSMUSG00000065380	snRNA	HITS-CLIP,Piranha_0.01	4
Gm23238	ENSMUSG00000064941	snRNA	HITS-CLIP,Piranha_0.01	4
Gm23240	ENSMUSG00000064943	snRNA	HITS-CLIP,Piranha_0.01	4
Gm23459	ENSMUSG00000065193	snRNA	HITS-CLIP,Piranha_0.01	4
Gm23547	ENSMUSG00000088835	snoRNA	HITS-CLIP,Piranha_0.01	4
Gm23982	ENSMUSG00000087766	snRNA	HITS-CLIP,Piranha_0.01	4
Gm24144	ENSMUSG00000064566	snRNA	HITS-CLIP,Piranha_0.01	4
Gm24201	ENSMUSG00000064880	snoRNA	HITS-CLIP,Piranha_0.01	4
Gm24305	ENSMUSG00000095590	snRNA	HITS-CLIP,Piranha_0.01	4
Gm24357	ENSMUSG00000064797	snoRNA	HITS-CLIP,Piranha_0.01	4
Gm24449	ENSMUSG00000065919	snRNA	HITS-CLIP,Piranha_0.01	4
Gm24830	ENSMUSG00000095701	snRNA	HITS-CLIP,Piranha_0.01	4
Gm24927	ENSMUSG00000065331	snRNA	HITS-CLIP,Piranha_0.01	4
Gm25202	ENSMUSG00000064772	snRNA	HITS-CLIP,Piranha_0.01	4
Gm25257	ENSMUSG00000094976	snRNA	HITS-CLIP,Piranha_0.01	4
Gm25412	ENSMUSG00000096222	snRNA	HITS-CLIP,Piranha_0.01	4
Gm25514	ENSMUSG00000065794	snRNA	HITS-CLIP,Piranha_0.01	4
Gm26110	ENSMUSG00000065905	snRNA	HITS-CLIP,Piranha_0.01	4
Gm26325	ENSMUSG00000065006	snRNA	HITS-CLIP,Piranha_0.01	4
Gm26330	ENSMUSG00000064751	snoRNA	HITS-CLIP,Piranha_0.01	4
Gm26377	ENSMUSG00000076138	miRNA	HITS-CLIP,Piranha_0.01	4
Gm26820	ENSMUSG00000097031	lincRNA	HITS-CLIP,Piranha_0.01	4
Gm31718	ENSMUSG00000110697	lincRNA	HITS-CLIP,Piranha_0.01	4
Gm3331	ENSMUSG00000099148	lincRNA	HITS-CLIP,Piranha_0.01	4
Gm35692	ENSMUSG00000117139	lincRNA	HITS-CLIP,Piranha_0.01	4
Gm37357	ENSMUSG00000103625	TEC	HITS-CLIP,Piranha_0.01	4
Gm49711	ENSMUSG00000116930	protein_coding	HITS-CLIP,Piranha_0.01	4
Gnptab	ENSMUSG00000035311	protein_coding	HITS-CLIP,Piranha_0.01	4
Gpr171	ENSMUSG00000050075	protein_coding	HITS-CLIP,Piranha_0.01	4
Gtf2f2	ENSMUSG00000067995	protein_coding	HITS-CLIP,Piranha_0.01	4
Gulp1	ENSMUSG00000056870	protein_coding	HITS-CLIP,Piranha_0.01	4
H3f3b	ENSMUSG00000016559	protein_coding	HITS-CLIP,Piranha_0.01	4
Havcr2	ENSMUSG00000020399	protein_coding	HITS-CLIP,Piranha_0.01	4
Hectd1	ENSMUSG00000035247	protein_coding	HITS-CLIP,Piranha_0.01	4

Hivep1	ENSMUSG00000021366	protein_coding	HITS-CLIP,Piranha_0.01	4
Hivep3	ENSMUSG00000028634	protein_coding	HITS-CLIP,Piranha_0.01	4
Hnrnpr	ENSMUSG00000066037	protein_coding	HITS-CLIP,Piranha_0.01	4
Icos	ENSMUSG00000026009	protein_coding	HITS-CLIP,Piranha_0.01	4
Il4ra	ENSMUSG00000030748	protein_coding	HITS-CLIP,Piranha_0.01	4
Irf1	ENSMUSG00000018899	protein_coding	HITS-CLIP,Piranha_0.01	4
Irf2bp2	ENSMUSG00000051495	protein_coding	HITS-CLIP,Piranha_0.01	4
Jade3	ENSMUSG00000037315	protein_coding	HITS-CLIP,Piranha_0.01	4
Jak3	ENSMUSG00000031805	protein_coding	HITS-CLIP,Piranha_0.01	4
Kansl1	ENSMUSG00000018412	protein_coding	HITS-CLIP,Piranha_0.01	4
Klhl31	ENSMUSG00000044938	protein_coding	HITS-CLIP,Piranha_0.01	4
Ksr1	ENSMUSG00000018334	protein_coding	HITS-CLIP,Piranha_0.01	4
Larp4b	ENSMUSG00000033499	protein_coding	HITS-CLIP,Piranha_0.01	4
Ldlr	ENSMUSG00000032193	protein_coding	HITS-CLIP,Piranha_0.01	4
Lef1	ENSMUSG00000027985	protein_coding	HITS-CLIP,Piranha_0.01	4
Lhfpl3	ENSMUSG00000106379	protein_coding	HITS-CLIP,Piranha_0.01	4
Lif	ENSMUSG00000034394	protein_coding	HITS-CLIP,CTK	4
Lrch1	ENSMUSG00000068015	protein_coding	HITS-CLIP,Piranha_0.01	4
Lta	ENSMUSG00000024402	protein_coding	HITS-CLIP,Piranha_0.01	4
Ly6e	ENSMUSG00000022587	protein_coding	HITS-CLIP,Piranha_0.01	4
Map3k8	ENSMUSG00000024235	protein_coding	HITS-CLIP,Piranha_0.01	4
Mapkapk2	ENSMUSG00000016528	protein_coding	HITS-CLIP,Piranha_0.01	4
Mcl1	ENSMUSG00000038612	protein_coding	HITS-CLIP,Piranha_0.01	4
Med1	ENSMUSG00000018160	protein_coding	HITS-CLIP,Piranha_0.01	4
Mef2a	ENSMUSG00000030557	protein_coding	HITS-CLIP,Piranha_0.01	4
Micu1	ENSMUSG00000020111	protein_coding	HITS-CLIP,Piranha_0.01	4
Mir142	ENSMUSG00000105196	miRNA	HITS-CLIP,Piranha_0.01	4
Mir18	ENSMUSG00000065403	miRNA	HITS-CLIP,Piranha_0.01	4
Mir19a	ENSMUSG00000065416	miRNA	HITS-CLIP,Piranha_0.01	4
Mir23b	ENSMUSG00000065599	miRNA	HITS-CLIP,Piranha_0.01	4
Mir26a-2	ENSMUSG00000065430	miRNA	HITS-CLIP,Piranha_0.01	4
Mir6236	ENSMUSG00000098973	miRNA	HITS-CLIP,Piranha_0.01	4
Mmp13	ENSMUSG00000050578	protein_coding	HITS-CLIP,Piranha_0.01	4
Mtdh	ENSMUSG00000022255	protein_coding	HITS-CLIP,Piranha_0.01	4
Mxd1	ENSMUSG00000001156	protein_coding	HITS-CLIP,Piranha_0.01	4
Myt1l	ENSMUSG00000061911	protein_coding	HITS-CLIP,Piranha_0.01	4
Mzt1	ENSMUSG00000033186	protein_coding	HITS-CLIP,Piranha_0.01	4
n-R5-8s1	ENSMUSG00000065922	rRNA	HITS-CLIP,Piranha_0.01	4
n-R5s156	ENSMUSG00000065311	rRNA	HITS-CLIP,Piranha_0.01	4
n-R5s210	ENSMUSG00000095944	rRNA	HITS-CLIP,Piranha_0.01	4
n-R5s58	ENSMUSG00000070192	rRNA	HITS-CLIP,Piranha_0.01	4
Neb	ENSMUSG00000026950	protein_coding	HITS-CLIP,Piranha_0.01	4
Nell1	ENSMUSG00000055409	protein_coding	HITS-CLIP,Piranha_0.01	4
Nfkbiz	ENSMUSG00000035356	protein_coding	HITS-CLIP,Piranha_0.01	4
Nhs	ENSMUSG00000059493	protein_coding	HITS-CLIP,Piranha_0.01	4
Ntrk2	ENSMUSG00000055254	protein_coding	HITS-CLIP,Piranha_0.01	4
Nup205	ENSMUSG00000038759	protein_coding	HITS-CLIP,Piranha_0.01	4
Nus1	ENSMUSG00000023068	protein_coding	HITS-CLIP,Piranha_0.01	4
Odc1	ENSMUSG00000011179	protein_coding	HITS-CLIP,Piranha_0.01	4
Pah	ENSMUSG00000020051	protein_coding	HITS-CLIP,Piranha_0.01	4
Parp8	ENSMUSG00000021725	protein_coding	HITS-CLIP,Piranha_0.01	4
Pdia3	ENSMUSG00000027248	protein_coding	HITS-CLIP,Piranha_0.01	4
Pdia4	ENSMUSG00000025823	protein_coding	HITS-CLIP,Piranha_0.01	4
Pecam1	ENSMUSG00000020717	protein_coding	HITS-CLIP,Piranha_0.01	4
Plcb4	ENSMUSG00000039943	protein_coding	HITS-CLIP,Piranha_0.01	4
Plcxd2	ENSMUSG00000087141	protein_coding	HITS-CLIP,Piranha_0.01	4
Polr3d	ENSMUSG00000000776	protein_coding	HITS-CLIP,Piranha_0.01	4
Ppp1r15b	ENSMUSG00000046062	protein_coding	HITS-CLIP,Piranha_0.01	4

Ppp2r2a	ENSMUSG00000022052	protein_coding	HITS-CLIP,Piranha_0.01	4
Pptc7	ENSMUSG00000038582	protein_coding	HITS-CLIP,Piranha_0.01	4
Prdm2	ENSMUSG00000057637	protein_coding	HITS-CLIP,Piranha_0.01	4
Prkcb	ENSMUSG00000052889	protein_coding	HITS-CLIP,Piranha_0.01	4
Psap	ENSMUSG00000004207	protein_coding	HITS-CLIP,Piranha_0.01	4
Psme3	ENSMUSG00000078652	protein_coding	HITS-CLIP,Piranha_0.01	4
Ptpn13	ENSMUSG00000034573	protein_coding	HITS-CLIP,Piranha_0.01	4
Ptpn9	ENSMUSG00000032290	protein_coding	HITS-CLIP,Piranha_0.01	4
Ptprt	ENSMUSG00000053141	protein_coding	HITS-CLIP,Piranha_0.01	4
Qrich1	ENSMUSG00000006673	protein_coding	HITS-CLIP,Piranha_0.01	4
R3hdm1	ENSMUSG00000056211	protein_coding	HITS-CLIP,Piranha_0.01	4
Ranbp17	ENSMUSG00000040594	protein_coding	HITS-CLIP,Piranha_0.01	4
Rbm26	ENSMUSG00000022119	protein_coding	HITS-CLIP,Piranha_0.01	4
Rhoa	ENSMUSG00000007815	protein_coding	HITS-CLIP,Piranha_0.01	4
Rif1	ENSMUSG00000036202	protein_coding	HITS-CLIP,Piranha_0.01	4
Riox2	ENSMUSG00000022724	protein_coding	HITS-CLIP,Piranha_0.01	4
Rmc1	ENSMUSG00000024410	protein_coding	HITS-CLIP,Piranha_0.01	4
Rmi1	ENSMUSG00000035367	protein_coding	HITS-CLIP,Piranha_0.01	4
Rpl32	ENSMUSG00000057841	protein_coding	HITS-CLIP,Piranha_0.01	4
Rpl37	ENSMUSG00000041841	protein_coding	HITS-CLIP,Piranha_0.01	4
Rpl8	ENSMUSG00000003970	protein_coding	HITS-CLIP,Piranha_0.01	4
Rplp0	ENSMUSG00000067274	protein_coding	HITS-CLIP,Piranha_0.01	4
Runx1	ENSMUSG00000022952	protein_coding	HITS-CLIP,Piranha_0.01	4
Scaper	ENSMUSG00000034007	protein_coding	HITS-CLIP,Piranha_0.01	4
Schip1	ENSMUSG00000027777	protein_coding	HITS-CLIP,Piranha_0.01	4
Sdcbp	ENSMUSG00000028249	protein_coding	HITS-CLIP,Piranha_0.01	4
Sec22b	ENSMUSG00000027879	protein_coding	HITS-CLIP,Piranha_0.01	4
Senp6	ENSMUSG00000034252	protein_coding	HITS-CLIP,Piranha_0.01	4
Sh2d2a	ENSMUSG00000028071	protein_coding	HITS-CLIP,Piranha_0.01	4
Simc1	ENSMUSG00000043183	protein_coding	HITS-CLIP,Piranha_0.01	4
Sirt2	ENSMUSG00000015149	protein_coding	HITS-CLIP,Piranha_0.01	4
Slc12a4	ENSMUSG00000017765	protein_coding	HITS-CLIP,Piranha_0.01	4
Slc22a23	ENSMUSG00000038267	protein_coding	HITS-CLIP,Piranha_0.01	4
Slc22a27	ENSMUSG00000067656	protein_coding	HITS-CLIP,Piranha_0.01	4
Slc7a6	ENSMUSG00000031904	protein_coding	HITS-CLIP,Piranha_0.01	4
Smad4	ENSMUSG00000024515	protein_coding	HITS-CLIP,Piranha_0.01	4
Smarcd3	ENSMUSG00000028949	protein_coding	HITS-CLIP,Piranha_0.01	4
Smg9	ENSMUSG00000002210	protein_coding	HITS-CLIP,Piranha_0.01	4
Snora20	ENSMUSG00000064637	snoRNA	HITS-CLIP,Piranha_0.01	4
Snora26	ENSMUSG00000093355	snoRNA	HITS-CLIP,Piranha_0.01	4
Snora31	ENSMUSG00000065147	snoRNA	HITS-CLIP,Piranha_0.01	4
Snora33	ENSMUSG00000070063	snoRNA	HITS-CLIP,Piranha_0.01	4
Snora73a	ENSMUSG00000064387	snoRNA	HITS-CLIP,Piranha_0.01	4
Snord110	ENSMUSG00000080610	snoRNA	HITS-CLIP,Piranha_0.01	4
Snord14c	ENSMUSG00000096263	snoRNA	HITS-CLIP,Piranha_0.01	4
Snord38a	ENSMUSG00000065680	snoRNA	HITS-CLIP,Piranha_0.01	4
Snx3	ENSMUSG00000019804	protein_coding	HITS-CLIP,Piranha_0.01	4
Sox5	ENSMUSG00000041540	protein_coding	HITS-CLIP,Piranha_0.01	4
Sp3	ENSMUSG00000027109	protein_coding	HITS-CLIP,Piranha_0.01	4
Spcs3	ENSMUSG00000054408	protein_coding	HITS-CLIP,Piranha_0.01	4
Ssr3	ENSMUSG00000027828	protein_coding	HITS-CLIP,Piranha_0.01	4
St13	ENSMUSG00000022403	protein_coding	HITS-CLIP,Piranha_0.01	4
Stk26	ENSMUSG00000031112	protein_coding	HITS-CLIP,Piranha_0.01	4
Stk3	ENSMUSG00000022329	protein_coding	HITS-CLIP,Piranha_0.01	4
Stk39	ENSMUSG00000027030	protein_coding	HITS-CLIP,Piranha_0.01	4
Susd6	ENSMUSG00000021133	protein_coding	HITS-CLIP,Piranha_0.01	4
Syn3	ENSMUSG00000059602	protein_coding	HITS-CLIP,Piranha_0.01	4
Syvn1	ENSMUSG00000024807	protein_coding	HITS-CLIP,Piranha_0.01	4

Taf12	ENSMUSG00000028899	protein_coding	HITS-CLIP,Piranha_0.01	4
Tasp1	ENSMUSG00000039033	protein_coding	HITS-CLIP,Piranha_0.01	4
Tax1bp1	ENSMUSG00000004535	protein_coding	HITS-CLIP,Piranha_0.01	4
Tbp	ENSMUSG00000014767	protein_coding	HITS-CLIP,Piranha_0.01	4
Tent4a	ENSMUSG00000034575	protein_coding	HITS-CLIP,Piranha_0.01	4
Tes	ENSMUSG00000029552	protein_coding	HITS-CLIP,Piranha_0.01	4
Tet2	ENSMUSG00000040943	protein_coding	HITS-CLIP,Piranha_0.01	4
Tg	ENSMUSG00000053469	protein_coding	HITS-CLIP,Piranha_0.01	4
Thsd7b	ENSMUSG00000042581	protein_coding	HITS-CLIP,Piranha_0.01	4
Timp3	ENSMUSG00000020044	protein_coding	HITS-CLIP,Piranha_0.01	4
Tmem248	ENSMUSG00000053094	protein_coding	HITS-CLIP,Piranha_0.01	4
Tnfsf9	ENSMUSG00000035678	protein_coding	HITS-CLIP,Piranha_0.01	4
Tnpo1	ENSMUSG00000009470	protein_coding	HITS-CLIP,Piranha_0.01	4
Top1	ENSMUSG00000070544	protein_coding	HITS-CLIP,Piranha_0.01	4
Tox2	ENSMUSG00000074607	protein_coding	HITS-CLIP,Piranha_0.01	4
Tpm4	ENSMUSG00000031799	protein_coding	HITS-CLIP,Piranha_0.01	4
Tram1	ENSMUSG00000025935	protein_coding	HITS-CLIP,Piranha_0.01	4
Trmt10c	ENSMUSG00000044763	protein_coding	HITS-CLIP,Piranha_0.01	4
Trmt11	ENSMUSG00000019792	protein_coding	HITS-CLIP,Piranha_0.01	4
Tsc22d2	ENSMUSG00000027806	protein_coding	HITS-CLIP,Piranha_0.01	4
Ttc7b	ENSMUSG00000033530	protein_coding	HITS-CLIP,Piranha_0.01	4
Ube2b	ENSMUSG00000020390	protein_coding	HITS-CLIP,Piranha_0.01	4
Ube2n	ENSMUSG00000074781	protein_coding	HITS-CLIP,Piranha_0.01	4
Uimc1	ENSMUSG00000025878	protein_coding	HITS-CLIP,Piranha_0.01	4
Unkl	ENSMUSG00000015127	protein_coding	HITS-CLIP,Piranha_0.01	4
Usf3	ENSMUSG00000068284	protein_coding	HITS-CLIP,Piranha_0.01	4
Wasf2	ENSMUSG00000028868	protein_coding	HITS-CLIP,Piranha_0.01	4
Wdr26	ENSMUSG00000038733	protein_coding	HITS-CLIP,Piranha_0.01	4
Wsb1	ENSMUSG00000017677	protein_coding	HITS-CLIP,Piranha_0.01	4
Ybx1	ENSMUSG00000028639	protein_coding	HITS-CLIP,Piranha_0.01	4
Ywhaq	ENSMUSG00000076432	protein_coding	HITS-CLIP,Piranha_0.01	4
Zcchc7	ENSMUSG00000035649	protein_coding	HITS-CLIP,Piranha_0.01	4
Zfp703	ENSMUSG00000085795	protein_coding	HITS-CLIP,Piranha_0.01	4
Zfr	ENSMUSG00000022201	protein_coding	HITS-CLIP,Piranha_0.01	4
Znrf3	ENSMUSG00000041961	protein_coding	HITS-CLIP,Piranha_0.01	4
2010003K11Rik	ENSMUSG00000042041	protein_coding	HITS-CLIP,Piranha_0.01	3
2410002F23Rik	ENSMUSG00000045411	protein_coding	HITS-CLIP,Piranha_0.01	3
2700097O09Rik	ENSMUSG00000062198	protein_coding	HITS-CLIP,Piranha_0.01	3
3830408C21Rik	ENSMUSG00000071181	processed_transcri	HITS-CLIP,Piranha_0.01	3
4930461G14Rik	ENSMUSG00000097651	lincRNA	HITS-CLIP,Piranha_0.01	3
Acsl3	ENSMUSG00000032883	protein_coding	HITS-CLIP,Piranha_0.01	3
Adam19	ENSMUSG00000011256	protein_coding	HITS-CLIP,Piranha_0.01	3
Adnp	ENSMUSG00000051149	protein_coding	HITS-CLIP,Piranha_0.01	3
Adss	ENSMUSG00000015961	protein_coding	HITS-CLIP,Piranha_0.01	3
Agpat4	ENSMUSG00000023827	protein_coding	HITS-CLIP,Piranha_0.01	3
Ahr	ENSMUSG00000019256	protein_coding	HITS-CLIP,Piranha_0.01	3
AI506816	ENSMUSG00000105987	processed_transcri	HITS-CLIP,Piranha_0.01	3
AL607142.1	ENSMUSG00000115115	protein_coding	HITS-CLIP,Piranha_0.01	3
Alyref	ENSMUSG00000025134	protein_coding	HITS-CLIP,Piranha_0.01	3
Anln	ENSMUSG00000036777	protein_coding	HITS-CLIP,Piranha_0.01	3
Anxa7	ENSMUSG00000021814	protein_coding	HITS-CLIP,Piranha_0.01	3
Aqr	ENSMUSG00000040383	protein_coding	HITS-CLIP,Piranha_0.01	3
Arhgap31	ENSMUSG00000022799	protein_coding	HITS-CLIP,Piranha_0.01	3
Arpc3	ENSMUSG00000029465	protein_coding	HITS-CLIP,Piranha_0.01	3
Asxl1	ENSMUSG00000042548	protein_coding	HITS-CLIP,Piranha_0.01	3
Atg10	ENSMUSG00000021619	protein_coding	HITS-CLIP,Piranha_0.01	3
B230217J21Rik	ENSMUSG00000112410	lincRNA	HITS-CLIP,Piranha_0.01	3
B4galt1	ENSMUSG00000028413	protein_coding	HITS-CLIP,Piranha_0.01	3

Bicdl1	ENSMUSG00000041609	protein_coding	HITS-CLIP,Piranha_0.01	3
Bola3	ENSMUSG00000045160	protein_coding	HITS-CLIP,Piranha_0.01	3
Bora	ENSMUSG00000022070	protein_coding	HITS-CLIP,Piranha_0.01	3
Brca1	ENSMUSG00000017146	protein_coding	HITS-CLIP,Piranha_0.01	3
Btf3	ENSMUSG00000021660	protein_coding	HITS-CLIP,Piranha_0.01	3
Btg2	ENSMUSG00000020423	protein_coding	HITS-CLIP,Piranha_0.01	3
C1qbp	ENSMUSG00000018446	protein_coding	HITS-CLIP,Piranha_0.01	3
Calm2	ENSMUSG00000036438	protein_coding	HITS-CLIP,Piranha_0.01	3
Camsap2	ENSMUSG00000041570	protein_coding	HITS-CLIP,Piranha_0.01	3
Capza1	ENSMUSG00000070372	protein_coding	HITS-CLIP,Piranha_0.01	3
Casc3	ENSMUSG00000078676	protein_coding	HITS-CLIP,Piranha_0.01	3
Ccnt2	ENSMUSG00000026349	protein_coding	HITS-CLIP,Piranha_0.01	3
Cct8	ENSMUSG00000025613	protein_coding	HITS-CLIP,Piranha_0.01	3
Cd2	ENSMUSG00000027863	protein_coding	HITS-CLIP,Piranha_0.01	3
Cd3g	ENSMUSG00000002033	protein_coding	HITS-CLIP,Piranha_0.01	3
Cep83	ENSMUSG00000020024	protein_coding	HITS-CLIP,Piranha_0.01	3
Cfl2	ENSMUSG00000062929	protein_coding	HITS-CLIP,Piranha_0.01	3
Clasp2	ENSMUSG00000033392	protein_coding	HITS-CLIP,Piranha_0.01	3
Clock	ENSMUSG00000029238	protein_coding	HITS-CLIP,Piranha_0.01	3
Cmas	ENSMUSG00000030282	protein_coding	HITS-CLIP,Piranha_0.01	3
Cntnap5a	ENSMUSG00000070695	protein_coding	HITS-CLIP,Piranha_0.01	3
Cops2	ENSMUSG00000027206	protein_coding	HITS-CLIP,Piranha_0.01	3
Csnk1g1	ENSMUSG00000032384	protein_coding	HITS-CLIP,Piranha_0.01	3
Cstf3	ENSMUSG00000027176	protein_coding	HITS-CLIP,Piranha_0.01	3
Cul2	ENSMUSG00000024231	protein_coding	HITS-CLIP,Piranha_0.01	3
Cyb5a	ENSMUSG00000024646	protein_coding	HITS-CLIP,Piranha_0.01	3
Cyb5b	ENSMUSG00000031924	protein_coding	HITS-CLIP,Piranha_0.01	3
Cyba	ENSMUSG00000006519	protein_coding	HITS-CLIP,Piranha_0.01	3
D130009I18Rik	ENSMUSG00000115432	lincRNA	HITS-CLIP,Piranha_0.01	3
D5Ertd579e	ENSMUSG00000029190	protein_coding	HITS-CLIP,Piranha_0.01	3
Dazap1	ENSMUSG00000069565	protein_coding	HITS-CLIP,Piranha_0.01	3
Dcun1d5	ENSMUSG00000032002	protein_coding	HITS-CLIP,Piranha_0.01	3
Ddah1	ENSMUSG00000028194	protein_coding	HITS-CLIP,Piranha_0.01	3
Ddx21	ENSMUSG00000020075	protein_coding	HITS-CLIP,Piranha_0.01	3
Dock11	ENSMUSG00000031093	protein_coding	HITS-CLIP,Piranha_0.01	3
Dpysl2	ENSMUSG00000022048	protein_coding	HITS-CLIP,Piranha_0.01	3
Egf	ENSMUSG00000028017	protein_coding	HITS-CLIP,Piranha_0.01	3
Eif3a	ENSMUSG00000024991	protein_coding	HITS-CLIP,Piranha_0.01	3
Elf1	ENSMUSG00000036461	protein_coding	HITS-CLIP,Piranha_0.01	3
Elmsan1	ENSMUSG00000042507	protein_coding	HITS-CLIP,Piranha_0.01	3
Ezr	ENSMUSG00000052397	protein_coding	HITS-CLIP,Piranha_0.01	3
Fgfr2	ENSMUSG00000030849	protein_coding	HITS-CLIP,Piranha_0.01	3
Fryl	ENSMUSG00000070733	protein_coding	HITS-CLIP,Piranha_0.01	3
Fut2	ENSMUSG00000055978	protein_coding	HITS-CLIP,Piranha_0.01	3
Gabpb1	ENSMUSG00000027361	protein_coding	HITS-CLIP,Piranha_0.01	3
Gata3	ENSMUSG00000015619	protein_coding	HITS-CLIP,Piranha_0.01	3
Gcnt1	ENSMUSG00000038843	protein_coding	HITS-CLIP,Piranha_0.01	3
Gfi1	ENSMUSG00000029275	protein_coding	HITS-CLIP,Piranha_0.01	3
Glrx	ENSMUSG00000021591	protein_coding	HITS-CLIP,Piranha_0.01	3
Gm22129	ENSMUSG00000096434	snRNA	HITS-CLIP,Piranha_0.01	3
Gm22154	ENSMUSG00000065778	snoRNA	HITS-CLIP,Piranha_0.01	3
Gm22886	ENSMUSG00000096341	snRNA	HITS-CLIP,Piranha_0.01	3
Gm22975	ENSMUSG00000065237	snRNA	HITS-CLIP,Piranha_0.01	3
Gm23445	ENSMUSG00000064734	snRNA	HITS-CLIP,Piranha_0.01	3
Gm23455	ENSMUSG00000065265	snoRNA	HITS-CLIP,Piranha_0.01	3
Gm24601	ENSMUSG00000088595	rRNA	HITS-CLIP,Piranha_0.01	3
Gm24616	ENSMUSG00000065208	snoRNA	HITS-CLIP,Piranha_0.01	3
Gm25137	ENSMUSG00000065185	snRNA	HITS-CLIP,Piranha_0.01	3

Gm25189	ENSMUSG00000065845	snRNA	HITS-CLIP,Piranha_0.01	3
Gm25588	ENSMUSG00000064435	snoRNA	HITS-CLIP,Piranha_0.01	3
Gm25663	ENSMUSG00000088323	snoRNA	HITS-CLIP,Piranha_0.01	3
Gm25682	ENSMUSG00000065876	snRNA	HITS-CLIP,Piranha_0.01	3
Gm25788	ENSMUSG00000064655	snoRNA	HITS-CLIP,Piranha_0.01	3
Gm25835	ENSMUSG00000089542	snoRNA	HITS-CLIP,Piranha_0.01	3
Gm26972	ENSMUSG00000097980	lincRNA	HITS-CLIP,Piranha_0.01	3
Gm30159	ENSMUSG00000115164	lincRNA	HITS-CLIP,Piranha_0.01	3
Gm32764	ENSMUSG00000115140	lincRNA	HITS-CLIP,Piranha_0.01	3
Gm38357	ENSMUSG00000103313	TEC	HITS-CLIP,Piranha_0.01	3
Gm39323	ENSMUSG00000111229	lincRNA	HITS-CLIP,Piranha_0.01	3
Gm48877	ENSMUSG00000112873	antisense	HITS-CLIP,Piranha_0.01	3
Gtf2a1	ENSMUSG00000020962	protein_coding	HITS-CLIP,Piranha_0.01	3
Gtf2e2	ENSMUSG00000031585	protein_coding	HITS-CLIP,Piranha_0.01	3
H3c2	ENSMUSG00000069267	protein_coding	HITS-CLIP,Piranha_0.01	3
H4c1	ENSMUSG00000060093	protein_coding	HITS-CLIP,Piranha_0.01	3
H4c2	ENSMUSG00000069266	protein_coding	HITS-CLIP,Piranha_0.01	3
H4c3	ENSMUSG00000060678	protein_coding	HITS-CLIP,Piranha_0.01	3
Hdac9	ENSMUSG00000004698	protein_coding	HITS-CLIP,Piranha_0.01	3
Helz	ENSMUSG00000020721	protein_coding	HITS-CLIP,Piranha_0.01	3
Hs3st1	ENSMUSG00000051022	protein_coding	HITS-CLIP,Piranha_0.01	3
Hspa8	ENSMUSG00000015656	protein_coding	HITS-CLIP,Piranha_0.01	3
Htra1	ENSMUSG00000006205	protein_coding	HITS-CLIP,Piranha_0.01	3
Iars	ENSMUSG00000037851	protein_coding	HITS-CLIP,Piranha_0.01	3
Idh3a	ENSMUSG00000032279	protein_coding	HITS-CLIP,Piranha_0.01	3
Igf1r	ENSMUSG00000005533	protein_coding	HITS-CLIP,Piranha_0.01	3
Il22	ENSMUSG00000074695	protein_coding	HITS-CLIP,Piranha_0.01	3
Insig1	ENSMUSG00000045294	protein_coding	HITS-CLIP,Piranha_0.01	3
Itch	ENSMUSG00000027598	protein_coding	HITS-CLIP,Piranha_0.01	3
Kat6a	ENSMUSG00000031540	protein_coding	HITS-CLIP,Piranha_0.01	3
Kcnn4	ENSMUSG00000054342	protein_coding	HITS-CLIP,Piranha_0.01	3
Kcnq1ot1	ENSMUSG00000101609	antisense	HITS-CLIP,Piranha_0.01	3
Kdm5b	ENSMUSG00000042207	protein_coding	HITS-CLIP,Piranha_0.01	3
Kif13a	ENSMUSG00000021375	protein_coding	HITS-CLIP,Piranha_0.01	3
Klhl2	ENSMUSG00000031605	protein_coding	HITS-CLIP,Piranha_0.01	3
Lamb3	ENSMUSG00000026639	protein_coding	HITS-CLIP,Piranha_0.01	3
Lamc1	ENSMUSG00000026478	protein_coding	HITS-CLIP,Piranha_0.01	3
Lnpep	ENSMUSG00000023845	protein_coding	HITS-CLIP,Piranha_0.01	3
Lpp	ENSMUSG00000033306	protein_coding	HITS-CLIP,Piranha_0.01	3
Lrrc58	ENSMUSG00000034158	protein_coding	HITS-CLIP,Piranha_0.01	3
Mapk1	ENSMUSG00000063358	protein_coding	HITS-CLIP,Piranha_0.01	3
Mbd2	ENSMUSG00000024513	protein_coding	HITS-CLIP,Piranha_0.01	3
Med13	ENSMUSG00000034297	protein_coding	HITS-CLIP,Piranha_0.01	3
Med28	ENSMUSG00000015804	protein_coding	HITS-CLIP,Piranha_0.01	3
Mief1	ENSMUSG00000022412	protein_coding	HITS-CLIP,Piranha_0.01	3
Mir106b	ENSMUSG00000065514	miRNA	HITS-CLIP,Piranha_0.01	3
Mir17	ENSMUSG00000065508	miRNA	HITS-CLIP,Piranha_0.01	3
Mir20a	ENSMUSG00000065442	miRNA	HITS-CLIP,Piranha_0.01	3
Mir23a	ENSMUSG00000065611	miRNA	HITS-CLIP,Piranha_0.01	3
Mir29b-2	ENSMUSG00000065412	miRNA	HITS-CLIP,Piranha_0.01	3
Mir30e	ENSMUSG00000065409	miRNA	HITS-CLIP,Piranha_0.01	3
Mir3963	ENSMUSG00000092830	miRNA	HITS-CLIP,Piranha_0.01	3
Mir664	ENSMUSG00000105770	miRNA	HITS-CLIP,Piranha_0.01	3
Mirlet7g	ENSMUSG00000065440	miRNA	HITS-CLIP,Piranha_0.01	3
Mob1b	ENSMUSG00000006262	protein_coding	HITS-CLIP,Piranha_0.01	3
Mt1	ENSMUSG00000031765	protein_coding	HITS-CLIP,Piranha_0.01	3
Mthfd1l	ENSMUSG00000040675	protein_coding	HITS-CLIP,Piranha_0.01	3
Myzap	ENSMUSG00000041361	protein_coding	HITS-CLIP,Piranha_0.01	3

n-R5s161	ENSMUSG00000065662	rRNA	HITS-CLIP,Piranha_0.01	3
n-R5s33	ENSMUSG00000070225	rRNA	HITS-CLIP,Piranha_0.01	3
n-R5s77	ENSMUSG00000065691	rRNA	HITS-CLIP,Piranha_0.01	3
Naa16	ENSMUSG00000022020	protein_coding	HITS-CLIP,Piranha_0.01	3
Naf1	ENSMUSG00000014907	protein_coding	HITS-CLIP,Piranha_0.01	3
Nampt	ENSMUSG00000020572	protein_coding	HITS-CLIP,Piranha_0.01	3
Ncapd3	ENSMUSG00000035024	protein_coding	HITS-CLIP,Piranha_0.01	3
Ncbp3	ENSMUSG00000020783	protein_coding	HITS-CLIP,Piranha_0.01	3
Ndfip2	ENSMUSG00000053253	protein_coding	HITS-CLIP,Piranha_0.01	3
Ndufb1-ps	ENSMUSG00000113902	protein_coding	HITS-CLIP,Piranha_0.01	3
Nol10	ENSMUSG00000061458	protein_coding	HITS-CLIP,Piranha_0.01	3
Nrg3	ENSMUSG00000041014	protein_coding	HITS-CLIP,Piranha_0.01	3
Nsun2	ENSMUSG00000021595	protein_coding	HITS-CLIP,Piranha_0.01	3
Nufip2	ENSMUSG00000037857	protein_coding	HITS-CLIP,Piranha_0.01	3
Orc2	ENSMUSG00000026037	protein_coding	HITS-CLIP,Piranha_0.01	3
Paip2b	ENSMUSG00000045896	protein_coding	HITS-CLIP,Piranha_0.01	3
Pced1b	ENSMUSG00000044250	protein_coding	HITS-CLIP,Piranha_0.01	3
Pcgf3	ENSMUSG00000033623	protein_coding	HITS-CLIP,Piranha_0.01	3
Pdgfc	ENSMUSG00000028019	protein_coding	HITS-CLIP,Piranha_0.01	3
Pdlim5	ENSMUSG00000028273	protein_coding	HITS-CLIP,Piranha_0.01	3
Pds5b	ENSMUSG00000034021	protein_coding	HITS-CLIP,Piranha_0.01	3
Pdzd8	ENSMUSG00000074746	protein_coding	HITS-CLIP,Piranha_0.01	3
Per1	ENSMUSG00000020893	protein_coding	HITS-CLIP,Piranha_0.01	3
Pex6	ENSMUSG00000002763	protein_coding	HITS-CLIP,Piranha_0.01	3
Pitpnc1	ENSMUSG00000040430	protein_coding	HITS-CLIP,Piranha_0.01	3
Pitpnm2	ENSMUSG00000029406	protein_coding	HITS-CLIP,Piranha_0.01	3
Plekha3	ENSMUSG00000002733	protein_coding	HITS-CLIP,Piranha_0.01	3
Ppat	ENSMUSG00000029246	protein_coding	HITS-CLIP,Piranha_0.01	3
Ppip5k2	ENSMUSG00000040648	protein_coding	HITS-CLIP,CTK	3
Ppip5k2	ENSMUSG00000040648	protein_coding	HITS-CLIP,Piranha_0.01	3
Ppp3cb	ENSMUSG00000021816	protein_coding	HITS-CLIP,Piranha_0.01	3
Ppp6r3	ENSMUSG00000024908	protein_coding	HITS-CLIP,Piranha_0.01	3
Prnp	ENSMUSG00000079037	protein_coding	HITS-CLIP,Piranha_0.01	3
Prpf40a	ENSMUSG00000061136	protein_coding	HITS-CLIP,Piranha_0.01	3
Ptdss1	ENSMUSG00000021518	protein_coding	HITS-CLIP,Piranha_0.01	3
Ptger2	ENSMUSG00000037759	protein_coding	HITS-CLIP,Piranha_0.01	3
Ptgis	ENSMUSG00000017969	protein_coding	HITS-CLIP,Piranha_0.01	3
Ptma	ENSMUSG00000026238	protein_coding	HITS-CLIP,Piranha_0.01	3
Ptpn2	ENSMUSG00000024539	protein_coding	HITS-CLIP,Piranha_0.01	3
Rab5a	ENSMUSG00000017831	protein_coding	HITS-CLIP,Piranha_0.01	3
Ralgds	ENSMUSG00000026821	protein_coding	HITS-CLIP,Piranha_0.01	3
Rap1a	ENSMUSG00000068798	protein_coding	HITS-CLIP,Piranha_0.01	3
Rasef	ENSMUSG00000043003	protein_coding	HITS-CLIP,Piranha_0.01	3
Rassf5	ENSMUSG00000026430	protein_coding	HITS-CLIP,Piranha_0.01	3
Rbms1	ENSMUSG00000026970	protein_coding	HITS-CLIP,Piranha_0.01	3
Reps2	ENSMUSG00000040855	protein_coding	HITS-CLIP,Piranha_0.01	3
Ric1	ENSMUSG00000038658	protein_coding	HITS-CLIP,Piranha_0.01	3
Rnu11	ENSMUSG00000077323	snRNA	HITS-CLIP,Piranha_0.01	3
Robo1	ENSMUSG00000022883	protein_coding	HITS-CLIP,Piranha_0.01	3
Rpl30	ENSMUSG00000058600	protein_coding	HITS-CLIP,Piranha_0.01	3
Rps26	ENSMUSG00000025362	protein_coding	HITS-CLIP,Piranha_0.01	3
Rreb1	ENSMUSG00000039087	protein_coding	HITS-CLIP,Piranha_0.01	3
Runx2	ENSMUSG00000039153	protein_coding	HITS-CLIP,Piranha_0.01	3
S100a10	ENSMUSG00000041959	protein_coding	HITS-CLIP,Piranha_0.01	3
Sarnp	ENSMUSG00000078427	protein_coding	HITS-CLIP,Piranha_0.01	3
Scara5	ENSMUSG00000022032	protein_coding	HITS-CLIP,Piranha_0.01	3
Scarna9	ENSMUSG00000077506	snoRNA	HITS-CLIP,Piranha_0.01	3
Sdf4	ENSMUSG00000029076	protein_coding	HITS-CLIP,Piranha_0.01	3

Sec61a1	ENSMUSG00000030082	protein_coding	HITS-CLIP,Piranha_0.01	3
Selenoi	ENSMUSG00000075703	protein_coding	HITS-CLIP,Piranha_0.01	3
Septin11	ENSMUSG00000058013	protein_coding	HITS-CLIP,Piranha_0.01	3
Sgpl1	ENSMUSG00000020097	protein_coding	HITS-CLIP,Piranha_0.01	3
Slc16a1	ENSMUSG00000032902	protein_coding	HITS-CLIP,Piranha_0.01	3
Slc2a3	ENSMUSG00000003153	protein_coding	HITS-CLIP,Piranha_0.01	3
Slc30a9	ENSMUSG00000029221	protein_coding	HITS-CLIP,Piranha_0.01	3
Slc41a1	ENSMUSG00000013275	protein_coding	HITS-CLIP,Piranha_0.01	3
Smc2	ENSMUSG00000028312	protein_coding	HITS-CLIP,Piranha_0.01	3
Snhg12	ENSMUSG00000086290	lincRNA	HITS-CLIP,Piranha_0.01	3
Snora36b	ENSMUSG00000077571	snoRNA	HITS-CLIP,Piranha_0.01	3
Snora74a	ENSMUSG00000065649	snoRNA	HITS-CLIP,Piranha_0.01	3
Snord1b	ENSMUSG00000077604	snoRNA	HITS-CLIP,Piranha_0.01	3
Snord32a	ENSMUSG00000065219	snoRNA	HITS-CLIP,Piranha_0.01	3
Snord83b	ENSMUSG00000077734	snoRNA	HITS-CLIP,Piranha_0.01	3
Snord91a	ENSMUSG00000077493	snoRNA	HITS-CLIP,Piranha_0.01	3
Snord92	ENSMUSG00000093289	snoRNA	HITS-CLIP,Piranha_0.01	3
Snord93	ENSMUSG00000095205	snoRNA	HITS-CLIP,Piranha_0.01	3
Snord95	ENSMUSG00000064780	snoRNA	HITS-CLIP,Piranha_0.01	3
Snrpf	ENSMUSG00000020018	protein_coding	HITS-CLIP,Piranha_0.01	3
Sos2	ENSMUSG00000034801	protein_coding	HITS-CLIP,Piranha_0.01	3
Sqstm1	ENSMUSG00000015837	protein_coding	HITS-CLIP,Piranha_0.01	3
Stag2	ENSMUSG00000025862	protein_coding	HITS-CLIP,Piranha_0.01	3
Stim2	ENSMUSG00000039156	protein_coding	HITS-CLIP,Piranha_0.01	3
Stk11	ENSMUSG00000003068	protein_coding	HITS-CLIP,Piranha_0.01	3
Stk17b	ENSMUSG00000026094	protein_coding	HITS-CLIP,Piranha_0.01	3
Tanc2	ENSMUSG00000053580	protein_coding	HITS-CLIP,Piranha_0.01	3
Tecpr2	ENSMUSG00000021275	protein_coding	HITS-CLIP,Piranha_0.01	3
Tmem184b	ENSMUSG00000009035	protein_coding	HITS-CLIP,Piranha_0.01	3
Tnfrsf1b	ENSMUSG00000028599	protein_coding	HITS-CLIP,Piranha_0.01	3
Tnip3	ENSMUSG00000044162	protein_coding	HITS-CLIP,Piranha_0.01	3
Tpm3	ENSMUSG00000027940	protein_coding	HITS-CLIP,Piranha_0.01	3
Trim12a	ENSMUSG00000066258	protein_coding	HITS-CLIP,Piranha_0.01	3
Ttc39b	ENSMUSG00000038172	protein_coding	HITS-CLIP,Piranha_0.01	3
Ucma	ENSMUSG00000026668	protein_coding	HITS-CLIP,Piranha_0.01	3
Uggt1	ENSMUSG00000037470	protein_coding	HITS-CLIP,Piranha_0.01	3
Usp24	ENSMUSG00000028514	protein_coding	HITS-CLIP,Piranha_0.01	3
Utp6	ENSMUSG00000035575	protein_coding	HITS-CLIP,Piranha_0.01	3
Vaultrc5	ENSMUSG00000065145	misc_RNA	HITS-CLIP,Piranha_0.01	3
Vgll4	ENSMUSG00000030315	protein_coding	HITS-CLIP,Piranha_0.01	3
Vps37a	ENSMUSG00000031600	protein_coding	HITS-CLIP,Piranha_0.01	3
Wdr89	ENSMUSG00000045690	protein_coding	HITS-CLIP,Piranha_0.01	3
Wipf2	ENSMUSG00000038013	protein_coding	HITS-CLIP,Piranha_0.01	3
Zap70	ENSMUSG00000026117	protein_coding	HITS-CLIP,Piranha_0.01	3
Zfc3h1	ENSMUSG00000034163	protein_coding	HITS-CLIP,Piranha_0.01	3
Zfp148	ENSMUSG00000022811	protein_coding	HITS-CLIP,CTK	3
Zfp827	ENSMUSG00000071064	protein_coding	HITS-CLIP,Piranha_0.01	3
Znrf2	ENSMUSG00000058446	protein_coding	HITS-CLIP,Piranha_0.01	3
1700028E10Rik	ENSMUSG00000097321	processed_transcri	HITS-CLIP,Piranha_0.01	2
1700123O12Rik	ENSMUSG00000086080	lincRNA	HITS-CLIP,Piranha_0.01	2
1810013L24Rik	ENSMUSG00000022507	protein_coding	HITS-CLIP,Piranha_0.01	2
1810037I17Rik	ENSMUSG00000054091	protein_coding	HITS-CLIP,Piranha_0.01	2
2900097C17Rik	ENSMUSG00000102869	lincRNA	HITS-CLIP,Piranha_0.01	2
A230057D06Rik	ENSMUSG00000109394	lincRNA	HITS-CLIP,Piranha_0.01	2
A530021J07Rik	ENSMUSG00000053528	lincRNA	HITS-CLIP,Piranha_0.01	2
Aatf	ENSMUSG00000018697	protein_coding	HITS-CLIP,Piranha_0.01	2
Abhd17b	ENSMUSG00000047368	protein_coding	HITS-CLIP,Piranha_0.01	2
Actr1a	ENSMUSG00000025228	protein_coding	HITS-CLIP,Piranha_0.01	2

Adipor1	ENSMUSG00000026457	protein_coding	HITS-CLIP,Piranha_0.01	2
Aff1	ENSMUSG00000029313	protein_coding	HITS-CLIP,Piranha_0.01	2
Aff4	ENSMUSG00000049470	protein_coding	HITS-CLIP,Piranha_0.01	2
AI504432	ENSMUSG00000056145	lincRNA	HITS-CLIP,Piranha_0.01	2
Ak2	ENSMUSG00000028792	protein_coding	HITS-CLIP,Piranha_0.01	2
Alg11	ENSMUSG00000063362	protein_coding	HITS-CLIP,Piranha_0.01	2
Alkbh5	ENSMUSG00000042650	protein_coding	HITS-CLIP,Piranha_0.01	2
Alpk2	ENSMUSG00000032845	protein_coding	HITS-CLIP,Piranha_0.01	2
Anapc1	ENSMUSG00000014355	protein_coding	HITS-CLIP,Piranha_0.01	2
Ankrd13c	ENSMUSG00000039988	protein_coding	HITS-CLIP,Piranha_0.01	2
Areg	ENSMUSG00000029378	protein_coding	HITS-CLIP,Piranha_0.01	2
Arfgef1	ENSMUSG00000067851	protein_coding	HITS-CLIP,Piranha_0.01	2
Arhgap23	ENSMUSG00000049807	protein_coding	HITS-CLIP,Piranha_0.01	2
Arl6ip1	ENSMUSG00000030654	protein_coding	HITS-CLIP,Piranha_0.01	2
Arpc1b	ENSMUSG00000029622	protein_coding	HITS-CLIP,Piranha_0.01	2
Ascc3	ENSMUSG00000038774	protein_coding	HITS-CLIP,Piranha_0.01	2
Atf4	ENSMUSG00000042406	protein_coding	HITS-CLIP,Piranha_0.01	2
Atl2	ENSMUSG00000059811	protein_coding	HITS-CLIP,Piranha_0.01	2
Avil	ENSMUSG00000025432	protein_coding	HITS-CLIP,Piranha_0.01	2
AW112010	ENSMUSG00000075010	lincRNA	HITS-CLIP,Piranha_0.01	2
BC005537	ENSMUSG00000019132	protein_coding	HITS-CLIP,Piranha_0.01	2
Bclaf1	ENSMUSG00000037608	protein_coding	HITS-CLIP,Piranha_0.01	2
Btaf1	ENSMUSG00000040565	protein_coding	HITS-CLIP,Piranha_0.01	2
Btbd11	ENSMUSG00000020042	protein_coding	HITS-CLIP,Piranha_0.01	2
C230085N15Rik	ENSMUSG00000102212	TEC	HITS-CLIP,Piranha_0.01	2
Calm3	ENSMUSG00000019370	protein_coding	HITS-CLIP,Piranha_0.01	2
Caprin1	ENSMUSG00000027184	protein_coding	HITS-CLIP,Piranha_0.01	2
Ccdc71l	ENSMUSG00000090946	protein_coding	HITS-CLIP,Piranha_0.01	2
Ccndbp1	ENSMUSG00000023572	protein_coding	HITS-CLIP,Piranha_0.01	2
Ccng2	ENSMUSG00000029385	protein_coding	HITS-CLIP,Piranha_0.01	2
Cd4	ENSMUSG00000023274	protein_coding	HITS-CLIP,Piranha_0.01	2
Cdc42se1	ENSMUSG00000046722	protein_coding	HITS-CLIP,Piranha_0.01	2
Cdc42se2	ENSMUSG00000052298	protein_coding	HITS-CLIP,Piranha_0.01	2
Cdc45	ENSMUSG00000000028	protein_coding	HITS-CLIP,Piranha_0.01	2
Cdipt	ENSMUSG00000030682	protein_coding	HITS-CLIP,Piranha_0.01	2
Cdk5r1	ENSMUSG00000048895	protein_coding	HITS-CLIP,Piranha_0.01	2
Chchd4	ENSMUSG00000034203	protein_coding	HITS-CLIP,Piranha_0.01	2
Chmp1b	ENSMUSG00000109901	protein_coding	HITS-CLIP,Piranha_0.01	2
Chordc1	ENSMUSG00000001774	protein_coding	HITS-CLIP,Piranha_0.01	2
Clca2	ENSMUSG00000036960	protein_coding	HITS-CLIP,Piranha_0.01	2
Clic1	ENSMUSG00000007041	protein_coding	HITS-CLIP,Piranha_0.01	2
Clstn2	ENSMUSG00000032452	protein_coding	HITS-CLIP,Piranha_0.01	2
Cpped1	ENSMUSG00000065979	protein_coding	HITS-CLIP,Piranha_0.01	2
Cramp1l	ENSMUSG00000038002	protein_coding	HITS-CLIP,Piranha_0.01	2
Ctla2a	ENSMUSG00000044258	protein_coding	HITS-CLIP,Piranha_0.01	2
Ctss	ENSMUSG00000038642	protein_coding	HITS-CLIP,Piranha_0.01	2
Cyth2	ENSMUSG00000003269	protein_coding	HITS-CLIP,Piranha_0.01	2
D630045J12Rik	ENSMUSG00000063455	protein_coding	HITS-CLIP,Piranha_0.01	2
Dchs2	ENSMUSG00000102692	protein_coding	HITS-CLIP,Piranha_0.01	2
Ddx31	ENSMUSG00000026806	protein_coding	HITS-CLIP,Piranha_0.01	2
Degs1	ENSMUSG00000038633	protein_coding	HITS-CLIP,Piranha_0.01	2
Dennd11	ENSMUSG00000037172	protein_coding	HITS-CLIP,Piranha_0.01	2
Dido1	ENSMUSG00000038914	protein_coding	HITS-CLIP,Piranha_0.01	2
Dlat	ENSMUSG00000000168	protein_coding	HITS-CLIP,Piranha_0.01	2
Dnaja1	ENSMUSG00000028410	protein_coding	HITS-CLIP,Piranha_0.01	2
Dnajc11	ENSMUSG00000039768	protein_coding	HITS-CLIP,Piranha_0.01	2
Dnajc7	ENSMUSG00000014195	protein_coding	HITS-CLIP,Piranha_0.01	2
Drd3	ENSMUSG00000022705	protein_coding	HITS-CLIP,Piranha_0.01	2

Dscam	ENSMUSG00000050272	protein_coding	HITS-CLIP,Piranha_0.01	2
Dstyk	ENSMUSG00000042046	protein_coding	HITS-CLIP,Piranha_0.01	2
Dthd1	ENSMUSG00000090326	protein_coding	HITS-CLIP,Piranha_0.01	2
Dut	ENSMUSG00000027203	protein_coding	HITS-CLIP,Piranha_0.01	2
Dyrk1a	ENSMUSG00000022897	protein_coding	HITS-CLIP,Piranha_0.01	2
Ecm1	ENSMUSG00000028108	protein_coding	HITS-CLIP,Piranha_0.01	2
Efna5	ENSMUSG00000048915	protein_coding	HITS-CLIP,Piranha_0.01	2
Ehd4	ENSMUSG00000027293	protein_coding	HITS-CLIP,Piranha_0.01	2
Ell2	ENSMUSG00000001542	protein_coding	HITS-CLIP,Piranha_0.01	2
Epb41l2	ENSMUSG00000019978	protein_coding	HITS-CLIP,Piranha_0.01	2
Eprs	ENSMUSG00000026615	protein_coding	HITS-CLIP,Piranha_0.01	2
Ets2	ENSMUSG00000022895	protein_coding	HITS-CLIP,Piranha_0.01	2
Ewsr1	ENSMUSG00000009079	protein_coding	HITS-CLIP,Piranha_0.01	2
Fam185a	ENSMUSG00000047221	protein_coding	HITS-CLIP,Piranha_0.01	2
Fbxl13	ENSMUSG00000048520	protein_coding	HITS-CLIP,Piranha_0.01	2
Fbxo33	ENSMUSG00000035329	protein_coding	HITS-CLIP,Piranha_0.01	2
Fbxo42	ENSMUSG00000028920	protein_coding	HITS-CLIP,Piranha_0.01	2
Fhl2	ENSMUSG00000008136	protein_coding	HITS-CLIP,Piranha_0.01	2
Fhl3	ENSMUSG00000032643	protein_coding	HITS-CLIP,Piranha_0.01	2
Foxn2	ENSMUSG00000034998	protein_coding	HITS-CLIP,Piranha_0.01	2
Fth1	ENSMUSG00000024661	protein_coding	HITS-CLIP,Piranha_0.01	2
Galnt2l	ENSMUSG00000092329	protein_coding	HITS-CLIP,Piranha_0.01	2
Gch1	ENSMUSG00000037580	protein_coding	HITS-CLIP,Piranha_0.01	2
Gdi1	ENSMUSG00000015291	protein_coding	HITS-CLIP,Piranha_0.01	2
Gimap3	ENSMUSG00000039264	protein_coding	HITS-CLIP,Piranha_0.01	2
Glb1	ENSMUSG00000045594	protein_coding	HITS-CLIP,Piranha_0.01	2
Glp1r	ENSMUSG00000024027	protein_coding	HITS-CLIP,Piranha_0.01	2
Gltp	ENSMUSG00000011884	protein_coding	HITS-CLIP,Piranha_0.01	2
Glud1	ENSMUSG00000021794	protein_coding	HITS-CLIP,Piranha_0.01	2
Gm11527	ENSMUSG00000085578	lincRNA	HITS-CLIP,Piranha_0.01	2
Gm19466	ENSMUSG00000118362	lincRNA	HITS-CLIP,Piranha_0.01	2
Gm22061	ENSMUSG00000064491	snRNA	HITS-CLIP,Piranha_0.01	2
Gm22165	ENSMUSG00000089039	snRNA	HITS-CLIP,Piranha_0.01	2
Gm22308	ENSMUSG00000096124	snRNA	HITS-CLIP,Piranha_0.01	2
Gm22354	ENSMUSG00000065853	snoRNA	HITS-CLIP,Piranha_0.01	2
Gm22673	ENSMUSG00000077603	snoRNA	HITS-CLIP,Piranha_0.01	2
Gm22858	ENSMUSG00000092713	snoRNA	HITS-CLIP,Piranha_0.01	2
Gm22956	ENSMUSG00000084469	snRNA	HITS-CLIP,Piranha_0.01	2
Gm22988	ENSMUSG00000084708	snoRNA	HITS-CLIP,Piranha_0.01	2
Gm23059	ENSMUSG00000089603	snRNA	HITS-CLIP,Piranha_0.01	2
Gm23102	ENSMUSG00000065658	snRNA	HITS-CLIP,Piranha_0.01	2
Gm23264	ENSMUSG00000094158	snRNA	HITS-CLIP,Piranha_0.01	2
Gm23734	ENSMUSG00000065687	snoRNA	HITS-CLIP,Piranha_0.01	2
Gm24043	ENSMUSG00000065042	snRNA	HITS-CLIP,Piranha_0.01	2
Gm24438	ENSMUSG00000088518	snoRNA	HITS-CLIP,Piranha_0.01	2
Gm24465	ENSMUSG00000096085	snRNA	HITS-CLIP,Piranha_0.01	2
Gm24525	ENSMUSG00000064851	snRNA	HITS-CLIP,Piranha_0.01	2
Gm25052	ENSMUSG00000084610	snRNA	HITS-CLIP,Piranha_0.01	2
Gm25080	ENSMUSG00000064460	snRNA	HITS-CLIP,Piranha_0.01	2
Gm25133	ENSMUSG00000064581	snoRNA	HITS-CLIP,Piranha_0.01	2
Gm25541	ENSMUSG00000080538	snRNA	HITS-CLIP,Piranha_0.01	2
Gm25894	ENSMUSG00000065392	snoRNA	HITS-CLIP,CTK	2
Gm26180	ENSMUSG00000093967	snRNA	HITS-CLIP,Piranha_0.01	2
Gm26387	ENSMUSG00000077426	snoRNA	HITS-CLIP,Piranha_0.01	2
Gm26397	ENSMUSG00000065637	snoRNA	HITS-CLIP,Piranha_0.01	2
Gm27166	ENSMUSG00000098519	lincRNA	HITS-CLIP,Piranha_0.01	2
Gm27694	ENSMUSG00000098462	misc_RNA	HITS-CLIP,Piranha_0.01	2
Gm2a	ENSMUSG00000000594	protein_coding	HITS-CLIP,Piranha_0.01	2

Gm34821	ENSMUSG00000109186	antisense	HITS-CLIP,Piranha_0.01	2
Gm37359	ENSMUSG00000103620	antisense	HITS-CLIP,Piranha_0.01	2
Gm37709	ENSMUSG00000102791	TEC	HITS-CLIP,Piranha_0.01	2
Gm42743	ENSMUSG00000050936	lincRNA	HITS-CLIP,Piranha_0.01	2
Gm43837	ENSMUSG00000105443	lincRNA	HITS-CLIP,Piranha_0.01	2
Gm44532	ENSMUSG00000108869	lincRNA	HITS-CLIP,Piranha_0.01	2
Gm44710	ENSMUSG00000109455	antisense	HITS-CLIP,Piranha_0.01	2
Gm45222	ENSMUSG00000109498	TEC	HITS-CLIP,Piranha_0.01	2
Gm47947	ENSMUSG00000114808	lincRNA	HITS-CLIP,Piranha_0.01	2
Gm50241	ENSMUSG00000117748	protein_coding	HITS-CLIP,Piranha_0.01	2
Gpatch2l	ENSMUSG00000021254	protein_coding	HITS-CLIP,Piranha_0.01	2
Gpc5	ENSMUSG00000022112	protein_coding	HITS-CLIP,Piranha_0.01	2
Grasp	ENSMUSG00000000531	protein_coding	HITS-CLIP,Piranha_0.01	2
Gsk3b	ENSMUSG00000022812	protein_coding	HITS-CLIP,Piranha_0.01	2
H2-D1	ENSMUSG00000073411	protein_coding	HITS-CLIP,Piranha_0.01	2
H2-M3	ENSMUSG00000016206	protein_coding	HITS-CLIP,Piranha_0.01	2
H2bc6	ENSMUSG00000047246	protein_coding	HITS-CLIP,Piranha_0.01	2
H2bc8	ENSMUSG00000058385	protein_coding	HITS-CLIP,Piranha_0.01	2
Hat1	ENSMUSG00000027018	protein_coding	HITS-CLIP,Piranha_0.01	2
Hectd2	ENSMUSG00000041180	protein_coding	HITS-CLIP,Piranha_0.01	2
Hnrnpul2	ENSMUSG00000071659	protein_coding	HITS-CLIP,Piranha_0.01	2
Hoxc6	ENSMUSG00000001661	protein_coding	HITS-CLIP,Piranha_0.01	2
Hs3st3b1	ENSMUSG00000070407	protein_coding	HITS-CLIP,Piranha_0.01	2
Hsf2	ENSMUSG00000019878	protein_coding	HITS-CLIP,Piranha_0.01	2
Hspe1	ENSMUSG00000073676	protein_coding	HITS-CLIP,Piranha_0.01	2
Ibtk	ENSMUSG00000035941	protein_coding	HITS-CLIP,Piranha_0.01	2
Il1r1	ENSMUSG00000026072	protein_coding	HITS-CLIP,Piranha_0.01	2
Il1rapl2	ENSMUSG00000059203	protein_coding	HITS-CLIP,Piranha_0.01	2
Il2	ENSMUSG00000027720	protein_coding	HITS-CLIP,CTK	2
Imp3	ENSMUSG00000032288	protein_coding	HITS-CLIP,Piranha_0.01	2
Ing5	ENSMUSG00000026283	protein_coding	HITS-CLIP,Piranha_0.01	2
Itga1	ENSMUSG00000042284	protein_coding	HITS-CLIP,Piranha_0.01	2
Itga6	ENSMUSG00000027111	protein_coding	HITS-CLIP,Piranha_0.01	2
Jagn1	ENSMUSG00000051256	protein_coding	HITS-CLIP,Piranha_0.01	2
Jun	ENSMUSG00000052684	protein_coding	HITS-CLIP,Piranha_0.01	2
Katnbl1	ENSMUSG00000027132	protein_coding	HITS-CLIP,Piranha_0.01	2
Kbtbd2	ENSMUSG00000059486	protein_coding	HITS-CLIP,Piranha_0.01	2
Kdelr2	ENSMUSG00000079111	protein_coding	HITS-CLIP,Piranha_0.01	2
Kdm4c	ENSMUSG00000028397	protein_coding	HITS-CLIP,Piranha_0.01	2
Kdm7a	ENSMUSG00000042599	protein_coding	HITS-CLIP,Piranha_0.01	2
Khdrbs1	ENSMUSG00000028790	protein_coding	HITS-CLIP,Piranha_0.01	2
Kif13b	ENSMUSG00000060012	protein_coding	HITS-CLIP,Piranha_0.01	2
Kmt2d	ENSMUSG00000048154	protein_coding	HITS-CLIP,Piranha_0.01	2
Kmt2e	ENSMUSG00000029004	protein_coding	HITS-CLIP,Piranha_0.01	2
Lat	ENSMUSG00000030742	protein_coding	HITS-CLIP,Piranha_0.01	2
Leng8	ENSMUSG00000035545	protein_coding	HITS-CLIP,Piranha_0.01	2
Lmnb1	ENSMUSG00000024590	protein_coding	HITS-CLIP,Piranha_0.01	2
Lmtk2	ENSMUSG00000038970	protein_coding	HITS-CLIP,Piranha_0.01	2
Lonp1	ENSMUSG00000041168	protein_coding	HITS-CLIP,Piranha_0.01	2
Lrp1b	ENSMUSG00000049252	protein_coding	HITS-CLIP,Piranha_0.01	2
Lsm12	ENSMUSG00000020922	protein_coding	HITS-CLIP,Piranha_0.01	2
Ly6c1	ENSMUSG00000079018	protein_coding	HITS-CLIP,Piranha_0.01	2
Malt1	ENSMUSG00000032688	protein_coding	HITS-CLIP,CTK	2
Map2k5	ENSMUSG00000058444	protein_coding	HITS-CLIP,Piranha_0.01	2
Marchf6	ENSMUSG00000039100	protein_coding	HITS-CLIP,Piranha_0.01	2
Max	ENSMUSG00000059436	protein_coding	HITS-CLIP,Piranha_0.01	2
Mcu	ENSMUSG00000009647	protein_coding	HITS-CLIP,Piranha_0.01	2
Mfng	ENSMUSG00000018169	protein_coding	HITS-CLIP,Piranha_0.01	2

Mfsd14a	ENSMUSG00000089911	protein_coding	HITS-CLIP,Piranha_0.01	2
Miga2	ENSMUSG00000026858	protein_coding	HITS-CLIP,Piranha_0.01	2
Mir6236	ENSMUSG00000098973	miRNA	HITS-CLIP,CTK	2
Mir6238	ENSMUSG00000099176	miRNA	HITS-CLIP,Piranha_0.01	2
Mirlet7f-1	ENSMUSG00000105621	miRNA	HITS-CLIP,Piranha_0.01	2
Mllt11	ENSMUSG00000053192	protein_coding	HITS-CLIP,Piranha_0.01	2
Mmadhc	ENSMUSG00000026766	protein_coding	HITS-CLIP,Piranha_0.01	2
Mrto4	ENSMUSG00000028741	protein_coding	HITS-CLIP,Piranha_0.01	2
Msl2	ENSMUSG00000066415	protein_coding	HITS-CLIP,Piranha_0.01	2
Mtmr3	ENSMUSG00000034354	protein_coding	HITS-CLIP,Piranha_0.01	2
n-R5s211	ENSMUSG00000096047	rRNA	HITS-CLIP,Piranha_0.01	2
n-R5s43	ENSMUSG00000084688	rRNA	HITS-CLIP,Piranha_0.01	2
Naaladl2	ENSMUSG00000102758	protein_coding	HITS-CLIP,Piranha_0.01	2
Ncaph	ENSMUSG00000034906	protein_coding	HITS-CLIP,Piranha_0.01	2
Nemp1	ENSMUSG00000040195	protein_coding	HITS-CLIP,Piranha_0.01	2
Nkapd1	ENSMUSG00000059820	protein_coding	HITS-CLIP,Piranha_0.01	2
Nkg7	ENSMUSG00000004612	protein_coding	HITS-CLIP,Piranha_0.01	2
Notch2	ENSMUSG00000027878	protein_coding	HITS-CLIP,Piranha_0.01	2
Nr2c2	ENSMUSG00000005893	protein_coding	HITS-CLIP,Piranha_0.01	2
Nt5dc3	ENSMUSG00000054027	protein_coding	HITS-CLIP,Piranha_0.01	2
Nup210	ENSMUSG00000030091	protein_coding	HITS-CLIP,Piranha_0.01	2
Oga	ENSMUSG00000025220	protein_coding	HITS-CLIP,Piranha_0.01	2
Olfr303	ENSMUSG00000039608	protein_coding	HITS-CLIP,Piranha_0.01	2
Ostf1	ENSMUSG00000024725	protein_coding	HITS-CLIP,Piranha_0.01	2
Ostm1	ENSMUSG00000038280	protein_coding	HITS-CLIP,Piranha_0.01	2
Pafah1b2	ENSMUSG00000003131	protein_coding	HITS-CLIP,Piranha_0.01	2
Pak1	ENSMUSG00000030774	protein_coding	HITS-CLIP,Piranha_0.01	2
Pdcd2l	ENSMUSG00000002635	protein_coding	HITS-CLIP,Piranha_0.01	2
Pdcd6ip	ENSMUSG00000032504	protein_coding	HITS-CLIP,Piranha_0.01	2
Pde4dip	ENSMUSG00000038170	protein_coding	HITS-CLIP,Piranha_0.01	2
Pebp4	ENSMUSG00000022085	protein_coding	HITS-CLIP,Piranha_0.01	2
Pfdn2	ENSMUSG00000006412	protein_coding	HITS-CLIP,Piranha_0.01	2
Pgm2	ENSMUSG00000029171	protein_coding	HITS-CLIP,Piranha_0.01	2
Pi4ka	ENSMUSG00000041720	protein_coding	HITS-CLIP,Piranha_0.01	2
Pias1	ENSMUSG00000032405	protein_coding	HITS-CLIP,Piranha_0.01	2
Pip4k2a	ENSMUSG00000026737	protein_coding	HITS-CLIP,Piranha_0.01	2
Plaur	ENSMUSG00000046223	protein_coding	HITS-CLIP,Piranha_0.01	2
Plcl1	ENSMUSG00000038349	protein_coding	HITS-CLIP,Piranha_0.01	2
Plek	ENSMUSG00000020120	protein_coding	HITS-CLIP,Piranha_0.01	2
Plekhj1	ENSMUSG00000035278	protein_coding	HITS-CLIP,Piranha_0.01	2
Plk2	ENSMUSG00000021701	protein_coding	HITS-CLIP,Piranha_0.01	2
Pmaip1	ENSMUSG00000024521	protein_coding	HITS-CLIP,Piranha_0.01	2
Pmpcb	ENSMUSG00000029017	protein_coding	HITS-CLIP,Piranha_0.01	2
Polr2a	ENSMUSG00000005198	protein_coding	HITS-CLIP,Piranha_0.01	2
Pop1	ENSMUSG00000022325	protein_coding	HITS-CLIP,Piranha_0.01	2
Ppm1g	ENSMUSG00000029147	protein_coding	HITS-CLIP,Piranha_0.01	2
Ppm1h	ENSMUSG00000034613	protein_coding	HITS-CLIP,Piranha_0.01	2
Ppp2ca	ENSMUSG00000020349	protein_coding	HITS-CLIP,Piranha_0.01	2
Ppp3r1	ENSMUSG00000033953	protein_coding	HITS-CLIP,Piranha_0.01	2
Pram1	ENSMUSG00000032739	protein_coding	HITS-CLIP,Piranha_0.01	2
Prelid2	ENSMUSG00000056671	protein_coding	HITS-CLIP,Piranha_0.01	2
Prelid3b	ENSMUSG00000016257	protein_coding	HITS-CLIP,Piranha_0.01	2
Prkar1a	ENSMUSG00000020612	protein_coding	HITS-CLIP,Piranha_0.01	2
Prmt3	ENSMUSG00000030505	protein_coding	HITS-CLIP,Piranha_0.01	2
Prn	ENSMUSG00000098754	protein_coding	HITS-CLIP,Piranha_0.01	2
Prpf18	ENSMUSG00000039449	protein_coding	HITS-CLIP,Piranha_0.01	2
Prpf39	ENSMUSG00000035597	protein_coding	HITS-CLIP,Piranha_0.01	2
Prpf8	ENSMUSG00000020850	protein_coding	HITS-CLIP,Piranha_0.01	2

Psmd12	ENSMUSG00000020720	protein_coding	HITS-CLIP,Piranha_0.01	2
Psmf1	ENSMUSG00000032869	protein_coding	HITS-CLIP,Piranha_0.01	2
Ptger4	ENSMUSG00000039942	protein_coding	HITS-CLIP,Piranha_0.01	2
Qk	ENSMUSG00000062078	protein_coding	HITS-CLIP,Piranha_0.01	2
Qtrt2	ENSMUSG00000022704	protein_coding	HITS-CLIP,Piranha_0.01	2
Rab10	ENSMUSG00000020671	protein_coding	HITS-CLIP,Piranha_0.01	2
Rab14	ENSMUSG00000026878	protein_coding	HITS-CLIP,Piranha_0.01	2
Rac1	ENSMUSG00000001847	protein_coding	HITS-CLIP,Piranha_0.01	2
Rack1	ENSMUSG00000020372	protein_coding	HITS-CLIP,Piranha_0.01	2
Ran	ENSMUSG00000029430	protein_coding	HITS-CLIP,Piranha_0.01	2
Raph1	ENSMUSG00000026014	protein_coding	HITS-CLIP,Piranha_0.01	2
Rbm15	ENSMUSG00000048109	protein_coding	HITS-CLIP,Piranha_0.01	2
Rbx1	ENSMUSG00000022400	protein_coding	HITS-CLIP,Piranha_0.01	2
Rc3h1	ENSMUSG00000040423	protein_coding	HITS-CLIP,Piranha_0.01	2
Rdx	ENSMUSG00000032050	protein_coding	HITS-CLIP,Piranha_0.01	2
Rexo1	ENSMUSG00000047417	protein_coding	HITS-CLIP,Piranha_0.01	2
Rgcc	ENSMUSG00000022018	protein_coding	HITS-CLIP,Piranha_0.01	2
Riok1	ENSMUSG00000021428	protein_coding	HITS-CLIP,Piranha_0.01	2
Rnf19a	ENSMUSG00000022280	protein_coding	HITS-CLIP,Piranha_0.01	2
Rnf20	ENSMUSG00000028309	protein_coding	HITS-CLIP,Piranha_0.01	2
Selenos	ENSMUSG00000075701	protein_coding	HITS-CLIP,Piranha_0.01	2
Selenot	ENSMUSG00000075700	protein_coding	HITS-CLIP,Piranha_0.01	2
Serinc3	ENSMUSG00000017707	protein_coding	HITS-CLIP,Piranha_0.01	2
Serp1	ENSMUSG00000027808	protein_coding	HITS-CLIP,Piranha_0.01	2
Sesn3	ENSMUSG00000032009	protein_coding	HITS-CLIP,Piranha_0.01	2
Setd1a	ENSMUSG00000042308	protein_coding	HITS-CLIP,Piranha_0.01	2
Sf3b1	ENSMUSG00000025982	protein_coding	HITS-CLIP,Piranha_0.01	2
Sgcz	ENSMUSG00000039539	protein_coding	HITS-CLIP,Piranha_0.01	2
Shoc2	ENSMUSG00000024976	protein_coding	HITS-CLIP,Piranha_0.01	2
Sik1	ENSMUSG00000024042	protein_coding	HITS-CLIP,Piranha_0.01	2
Slc1a5	ENSMUSG00000001918	protein_coding	HITS-CLIP,Piranha_0.01	2
Slc20a1	ENSMUSG00000027397	protein_coding	HITS-CLIP,Piranha_0.01	2
Slc22a22	ENSMUSG00000022366	protein_coding	HITS-CLIP,Piranha_0.01	2
Slc25a3	ENSMUSG00000061904	protein_coding	HITS-CLIP,Piranha_0.01	2
Slc25a47	ENSMUSG00000048856	protein_coding	HITS-CLIP,Piranha_0.01	2
Slc35b1	ENSMUSG00000020873	protein_coding	HITS-CLIP,Piranha_0.01	2
Slc38a1	ENSMUSG00000023169	protein_coding	HITS-CLIP,CTK	2
Slc7a5	ENSMUSG00000040010	protein_coding	HITS-CLIP,Piranha_0.01	2
Smim3	ENSMUSG00000038059	protein_coding	HITS-CLIP,Piranha_0.01	2
Snhg15	ENSMUSG00000085156	lincRNA	HITS-CLIP,Piranha_0.01	2
Snora21	ENSMUSG00000064901	snoRNA	HITS-CLIP,Piranha_0.01	2
Snora30	ENSMUSG00000065259	snoRNA	HITS-CLIP,Piranha_0.01	2
Snora44	ENSMUSG00000064604	snoRNA	HITS-CLIP,Piranha_0.01	2
Snora68	ENSMUSG00000077563	snoRNA	HITS-CLIP,Piranha_0.01	2
Snora69	ENSMUSG00000065642	snoRNA	HITS-CLIP,Piranha_0.01	2
Snord15a	ENSMUSG00000065822	snoRNA	HITS-CLIP,Piranha_0.01	2
Snord71	ENSMUSG00000077549	snoRNA	HITS-CLIP,Piranha_0.01	2
Snrpa1	ENSMUSG00000030512	protein_coding	HITS-CLIP,Piranha_0.01	2
Snrpb	ENSMUSG00000027404	protein_coding	HITS-CLIP,Piranha_0.01	2
Snrpd3	ENSMUSG00000020180	protein_coding	HITS-CLIP,Piranha_0.01	2
Sp2	ENSMUSG00000018678	protein_coding	HITS-CLIP,Piranha_0.01	2
Spred1	ENSMUSG00000027351	protein_coding	HITS-CLIP,Piranha_0.01	2
Sqle	ENSMUSG00000022351	protein_coding	HITS-CLIP,Piranha_0.01	2
Srsf11	ENSMUSG00000055436	protein_coding	HITS-CLIP,Piranha_0.01	2
Ssr1	ENSMUSG00000021427	protein_coding	HITS-CLIP,Piranha_0.01	2
Stat3	ENSMUSG00000004040	protein_coding	HITS-CLIP,Piranha_0.01	2
Stim1	ENSMUSG00000030987	protein_coding	HITS-CLIP,Piranha_0.01	2
Stip1	ENSMUSG00000024966	protein_coding	HITS-CLIP,Piranha_0.01	2

Stk24	ENSMUSG00000063410	protein_coding	HITS-CLIP,Piranha_0.01	2
Stk4	ENSMUSG00000018209	protein_coding	HITS-CLIP,Piranha_0.01	2
Stt3a	ENSMUSG00000032116	protein_coding	HITS-CLIP,Piranha_0.01	2
Syne1	ENSMUSG00000096054	protein_coding	HITS-CLIP,Piranha_0.01	2
Taf15	ENSMUSG00000020680	protein_coding	HITS-CLIP,Piranha_0.01	2
Taok1	ENSMUSG00000017291	protein_coding	HITS-CLIP,Piranha_0.01	2
Tapt1	ENSMUSG00000046985	protein_coding	HITS-CLIP,Piranha_0.01	2
Tars	ENSMUSG00000022241	protein_coding	HITS-CLIP,Piranha_0.01	2
Tbc1d12	ENSMUSG00000048720	protein_coding	HITS-CLIP,Piranha_0.01	2
Tbk1	ENSMUSG00000020115	protein_coding	HITS-CLIP,Piranha_0.01	2
Tbpl1	ENSMUSG00000071359	protein_coding	HITS-CLIP,Piranha_0.01	2
Tcp1	ENSMUSG00000068039	protein_coding	HITS-CLIP,Piranha_0.01	2
Thada	ENSMUSG00000024251	protein_coding	HITS-CLIP,Piranha_0.01	2
Tm9sf3	ENSMUSG00000025016	protein_coding	HITS-CLIP,Piranha_0.01	2
Tmbim6	ENSMUSG00000023010	protein_coding	HITS-CLIP,Piranha_0.01	2
Tmed5	ENSMUSG00000063406	protein_coding	HITS-CLIP,Piranha_0.01	2
Tnfrsf18	ENSMUSG00000041954	protein_coding	HITS-CLIP,Piranha_0.01	2
Tollip	ENSMUSG00000025139	protein_coding	HITS-CLIP,Piranha_0.01	2
Tomm20	ENSMUSG00000093904	protein_coding	HITS-CLIP,Piranha_0.01	2
Top2b	ENSMUSG00000017485	protein_coding	HITS-CLIP,Piranha_0.01	2
Tpbg	ENSMUSG00000035274	protein_coding	HITS-CLIP,Piranha_0.01	2
Traf4	ENSMUSG00000017386	protein_coding	HITS-CLIP,Piranha_0.01	2
Trim25	ENSMUSG00000000275	protein_coding	HITS-CLIP,Piranha_0.01	2
Trim8	ENSMUSG00000025034	protein_coding	HITS-CLIP,Piranha_0.01	2
Trio	ENSMUSG00000022263	protein_coding	HITS-CLIP,Piranha_0.01	2
Trir	ENSMUSG00000041203	protein_coding	HITS-CLIP,Piranha_0.01	2
Try5	ENSMUSG00000036938	protein_coding	HITS-CLIP,Piranha_0.01	2
Ttc12	ENSMUSG00000040219	protein_coding	HITS-CLIP,Piranha_0.01	2
Ttl	ENSMUSG00000027394	protein_coding	HITS-CLIP,Piranha_0.01	2
Txn1	ENSMUSG00000028367	protein_coding	HITS-CLIP,Piranha_0.01	2
Ubac2	ENSMUSG00000041765	protein_coding	HITS-CLIP,Piranha_0.01	2
Ube2c	ENSMUSG00000001403	protein_coding	HITS-CLIP,Piranha_0.01	2
Ube2q1	ENSMUSG00000042572	protein_coding	HITS-CLIP,Piranha_0.01	2
Ube2z	ENSMUSG00000014349	protein_coding	HITS-CLIP,Piranha_0.01	2
Ube3a	ENSMUSG00000025326	protein_coding	HITS-CLIP,Piranha_0.01	2
Ubl3	ENSMUSG00000001687	protein_coding	HITS-CLIP,Piranha_0.01	2
Ubr1	ENSMUSG00000027272	protein_coding	HITS-CLIP,Piranha_0.01	2
Ubr3	ENSMUSG00000044308	protein_coding	HITS-CLIP,Piranha_0.01	2
Usp1	ENSMUSG00000028560	protein_coding	HITS-CLIP,Piranha_0.01	2
Usp31	ENSMUSG00000063317	protein_coding	HITS-CLIP,Piranha_0.01	2
Ust	ENSMUSG00000047712	protein_coding	HITS-CLIP,Piranha_0.01	2
Vasp	ENSMUSG00000030403	protein_coding	HITS-CLIP,Piranha_0.01	2
Vdac2	ENSMUSG00000021771	protein_coding	HITS-CLIP,Piranha_0.01	2
Washc4	ENSMUSG00000034560	protein_coding	HITS-CLIP,Piranha_0.01	2
Wdr75	ENSMUSG00000025995	protein_coding	HITS-CLIP,Piranha_0.01	2
Wfs1	ENSMUSG00000039474	protein_coding	HITS-CLIP,Piranha_0.01	2
Wnt5b	ENSMUSG00000030170	protein_coding	HITS-CLIP,Piranha_0.01	2
Xrcc4	ENSMUSG00000021615	protein_coding	HITS-CLIP,Piranha_0.01	2
Xrn2	ENSMUSG00000027433	protein_coding	HITS-CLIP,Piranha_0.01	2
Zbtb7a	ENSMUSG00000035011	protein_coding	HITS-CLIP,Piranha_0.01	2
Zc3h15	ENSMUSG00000027091	protein_coding	HITS-CLIP,Piranha_0.01	2
Zfp800	ENSMUSG00000039841	protein_coding	HITS-CLIP,Piranha_0.01	2
Zfp91	ENSMUSG00000024695	protein_coding	HITS-CLIP,Piranha_0.01	2
Zfp930	ENSMUSG00000059897	protein_coding	HITS-CLIP,Piranha_0.01	2
Zhx2	ENSMUSG00000071757	protein_coding	HITS-CLIP,Piranha_0.01	2
Zswim4	ENSMUSG00000035671	protein_coding	HITS-CLIP,Piranha_0.01	2
Zswim6	ENSMUSG00000032846	protein_coding	HITS-CLIP,Piranha_0.01	2
2310030A07Rik	ENSMUSG00000099447	antisense	HITS-CLIP,Piranha_0.01	1

4930417O13Rik	ENSMUSG00000108129	processed_transcri	HITS-CLIP,Piranha_0.01	1
4930471E19Rik	ENSMUSG00000112716	lincRNA	HITS-CLIP,Piranha_0.01	1
4930534D22Rik	ENSMUSG00000097761	antisense	HITS-CLIP,Piranha_0.01	1
4933411E02Rik	ENSMUSG00000110876	lincRNA	HITS-CLIP,Piranha_0.01	1
4933440M02Rik	ENSMUSG00000045928	lincRNA	HITS-CLIP,Piranha_0.01	1
5330409N07Rik	ENSMUSG00000113927	TEC	HITS-CLIP,Piranha_0.01	1
5830444F18Rik	ENSMUSG00000102744	TEC	HITS-CLIP,Piranha_0.01	1
6030443J06Rik	ENSMUSG00000097207	lincRNA	HITS-CLIP,Piranha_0.01	1
6720427I07Rik	ENSMUSG00000097375	lincRNA	HITS-CLIP,Piranha_0.01	1
8030462N17Rik	ENSMUSG00000047466	protein_coding	HITS-CLIP,Piranha_0.01	1
9030624G23Rik	ENSMUSG00000073158	protein_coding	HITS-CLIP,Piranha_0.01	1
9130230L23Rik	ENSMUSG00000054598	protein_coding	HITS-CLIP,Piranha_0.01	1
9630028H03Rik	ENSMUSG00000085631	antisense	HITS-CLIP,Piranha_0.01	1
9930017N22Rik	ENSMUSG00000115276	TEC	HITS-CLIP,Piranha_0.01	1
a	ENSMUSG00000027596	protein_coding	HITS-CLIP,Piranha_0.01	1
A130071D04Rik	ENSMUSG00000104291	TEC	HITS-CLIP,Piranha_0.01	1
A430073D23Rik	ENSMUSG00000105471	lincRNA	HITS-CLIP,Piranha_0.01	1
A530072M11Rik	ENSMUSG00000085112	processed_transcri	HITS-CLIP,Piranha_0.01	1
A630073D07Rik	ENSMUSG00000067541	protein_coding	HITS-CLIP,Piranha_0.01	1
A630081D01Rik	ENSMUSG00000102721	TEC	HITS-CLIP,Piranha_0.01	1
A930015D03Rik	ENSMUSG00000092368	antisense	HITS-CLIP,Piranha_0.01	1
Aco2	ENSMUSG00000022477	protein_coding	HITS-CLIP,Piranha_0.01	1
Actn1	ENSMUSG00000015143	protein_coding	HITS-CLIP,Piranha_0.01	1
Ago3	ENSMUSG00000028842	protein_coding	HITS-CLIP,Piranha_0.01	1
Ahctf1	ENSMUSG00000026491	protein_coding	HITS-CLIP,Piranha_0.01	1
Alkbh6	ENSMUSG00000042831	protein_coding	HITS-CLIP,Piranha_0.01	1
Alx1	ENSMUSG00000036602	protein_coding	HITS-CLIP,Piranha_0.01	1
Ankrd13a	ENSMUSG00000041870	protein_coding	HITS-CLIP,Piranha_0.01	1
Ankrd52	ENSMUSG00000014498	protein_coding	HITS-CLIP,Piranha_0.01	1
Ap3d1	ENSMUSG00000020198	protein_coding	HITS-CLIP,Piranha_0.01	1
Api5	ENSMUSG00000027193	protein_coding	HITS-CLIP,Piranha_0.01	1
Arap2	ENSMUSG00000037999	protein_coding	HITS-CLIP,Piranha_0.01	1
Arglu1	ENSMUSG00000040459	protein_coding	HITS-CLIP,Piranha_0.01	1
Arhgap19	ENSMUSG00000025154	protein_coding	HITS-CLIP,Piranha_0.01	1
Arid4a	ENSMUSG00000048118	protein_coding	HITS-CLIP,Piranha_0.01	1
Arl5a	ENSMUSG00000036093	protein_coding	HITS-CLIP,Piranha_0.01	1
Atad1	ENSMUSG00000013662	protein_coding	HITS-CLIP,Piranha_0.01	1
Atf6	ENSMUSG00000026663	protein_coding	HITS-CLIP,Piranha_0.01	1
Atf6b	ENSMUSG00000015461	protein_coding	HITS-CLIP,Piranha_0.01	1
Atf7	ENSMUSG00000099083	protein_coding	HITS-CLIP,Piranha_0.01	1
Atg4d	ENSMUSG00000002820	protein_coding	HITS-CLIP,Piranha_0.01	1
Atp1b3	ENSMUSG00000032412	protein_coding	HITS-CLIP,Piranha_0.01	1
Atxn10	ENSMUSG00000016541	protein_coding	HITS-CLIP,Piranha_0.01	1
B3galt1	ENSMUSG00000034780	protein_coding	HITS-CLIP,Piranha_0.01	1
B3galt6	ENSMUSG00000050796	protein_coding	HITS-CLIP,Piranha_0.01	1
B4galt5	ENSMUSG00000017929	protein_coding	HITS-CLIP,Piranha_0.01	1
Bcl10	ENSMUSG00000028191	protein_coding	HITS-CLIP,Piranha_0.01	1
Bid	ENSMUSG00000004446	protein_coding	HITS-CLIP,Piranha_0.01	1
Bsdc1	ENSMUSG00000040859	protein_coding	HITS-CLIP,Piranha_0.01	1
Bzw1	ENSMUSG00000051223	protein_coding	HITS-CLIP,Piranha_0.01	1
C030047K22Rik	ENSMUSG00000084873	lincRNA	HITS-CLIP,Piranha_0.01	1
Cacybp	ENSMUSG00000014226	protein_coding	HITS-CLIP,Piranha_0.01	1
Capza2	ENSMUSG00000015733	protein_coding	HITS-CLIP,Piranha_0.01	1
Card19	ENSMUSG00000037960	protein_coding	HITS-CLIP,Piranha_0.01	1
Casp3	ENSMUSG00000031628	protein_coding	HITS-CLIP,Piranha_0.01	1
Cbwd1	ENSMUSG00000024878	protein_coding	HITS-CLIP,Piranha_0.01	1
Ccdc50	ENSMUSG00000038127	protein_coding	HITS-CLIP,Piranha_0.01	1
Ccdc91	ENSMUSG00000030301	protein_coding	HITS-CLIP,Piranha_0.01	1

Ccni	ENSMUSG00000063015	protein_coding	HITS-CLIP,Piranha_0.01	1
Cd24a	ENSMUSG00000047139	protein_coding	HITS-CLIP,Piranha_0.01	1
Cd27	ENSMUSG00000030336	protein_coding	HITS-CLIP,Piranha_0.01	1
Cd5	ENSMUSG00000024669	protein_coding	HITS-CLIP,Piranha_0.01	1
Cd52	ENSMUSG00000000682	protein_coding	HITS-CLIP,Piranha_0.01	1
Cdc73	ENSMUSG00000026361	protein_coding	HITS-CLIP,Piranha_0.01	1
Cdk1	ENSMUSG00000019942	protein_coding	HITS-CLIP,Piranha_0.01	1
Cdk4	ENSMUSG00000006728	protein_coding	HITS-CLIP,Piranha_0.01	1
Cdyl2	ENSMUSG00000031758	protein_coding	HITS-CLIP,Piranha_0.01	1
Cggbp1	ENSMUSG00000054604	protein_coding	HITS-CLIP,Piranha_0.01	1
Ckb	ENSMUSG00000001270	protein_coding	HITS-CLIP,Piranha_0.01	1
Cluh	ENSMUSG00000020741	protein_coding	HITS-CLIP,Piranha_0.01	1
Cmtm6	ENSMUSG00000032434	protein_coding	HITS-CLIP,Piranha_0.01	1
Cnot6	ENSMUSG00000020362	protein_coding	HITS-CLIP,Piranha_0.01	1
Cntnap2	ENSMUSG00000039419	protein_coding	HITS-CLIP,Piranha_0.01	1
Col4a3bp	ENSMUSG00000021669	protein_coding	HITS-CLIP,Piranha_0.01	1
Col6a4	ENSMUSG00000032572	protein_coding	HITS-CLIP,CTK	1
Coq10b	ENSMUSG00000025981	protein_coding	HITS-CLIP,Piranha_0.01	1
Cpox	ENSMUSG00000022742	protein_coding	HITS-CLIP,Piranha_0.01	1
Creb3l2	ENSMUSG00000038648	protein_coding	HITS-CLIP,Piranha_0.01	1
Crebzf	ENSMUSG00000051451	protein_coding	HITS-CLIP,Piranha_0.01	1
Cstf2t	ENSMUSG00000053536	protein_coding	HITS-CLIP,Piranha_0.01	1
Cstf3	ENSMUSG00000027176	protein_coding	HITS-CLIP,CTK	1
Ctps	ENSMUSG00000028633	protein_coding	HITS-CLIP,Piranha_0.01	1
Cwc27	ENSMUSG00000021715	protein_coding	HITS-CLIP,Piranha_0.01	1
Cx3cr1	ENSMUSG00000052336	protein_coding	HITS-CLIP,Piranha_0.01	1
Dagla	ENSMUSG00000035735	protein_coding	HITS-CLIP,Piranha_0.01	1
Dcaf7	ENSMUSG00000049354	protein_coding	HITS-CLIP,Piranha_0.01	1
Dctn4	ENSMUSG00000024603	protein_coding	HITS-CLIP,Piranha_0.01	1
Ddit4	ENSMUSG00000020108	protein_coding	HITS-CLIP,Piranha_0.01	1
Ddx1	ENSMUSG00000037149	protein_coding	HITS-CLIP,Piranha_0.01	1
Ddx10	ENSMUSG00000053289	protein_coding	HITS-CLIP,Piranha_0.01	1
Ddx3y	ENSMUSG00000069045	protein_coding	HITS-CLIP,Piranha_0.01	1
Dek	ENSMUSG00000021377	protein_coding	HITS-CLIP,Piranha_0.01	1
Dennd4a	ENSMUSG00000053641	protein_coding	HITS-CLIP,Piranha_0.01	1
Dhx37	ENSMUSG00000029480	protein_coding	HITS-CLIP,Piranha_0.01	1
Dlg2	ENSMUSG00000052572	protein_coding	HITS-CLIP,Piranha_0.01	1
Dnah7a	ENSMUSG00000096141	protein_coding	HITS-CLIP,Piranha_0.01	1
Dnaja2	ENSMUSG00000031701	protein_coding	HITS-CLIP,Piranha_0.01	1
Dync1h1	ENSMUSG00000018707	protein_coding	HITS-CLIP,Piranha_0.01	1
E430014B02Rik	ENSMUSG00000102973	TEC	HITS-CLIP,Piranha_0.01	1
Efhd2	ENSMUSG00000040659	protein_coding	HITS-CLIP,Piranha_0.01	1
Egln1	ENSMUSG00000031987	protein_coding	HITS-CLIP,Piranha_0.01	1
Eif1	ENSMUSG00000035530	protein_coding	HITS-CLIP,Piranha_0.01	1
Eif2s1	ENSMUSG00000021116	protein_coding	HITS-CLIP,Piranha_0.01	1
Eif3j1	ENSMUSG00000027236	protein_coding	HITS-CLIP,Piranha_0.01	1
Eif4a2	ENSMUSG00000022884	protein_coding	HITS-CLIP,Piranha_0.01	1
Eif4ebp1	ENSMUSG00000031490	protein_coding	HITS-CLIP,Piranha_0.01	1
Elk1	ENSMUSG00000009406	protein_coding	HITS-CLIP,Piranha_0.01	1
Emb	ENSMUSG00000021728	protein_coding	HITS-CLIP,Piranha_0.01	1
Erg28	ENSMUSG00000021252	protein_coding	HITS-CLIP,Piranha_0.01	1
F2r	ENSMUSG00000048376	protein_coding	HITS-CLIP,Piranha_0.01	1
F2rl2	ENSMUSG00000021675	protein_coding	HITS-CLIP,Piranha_0.01	1
Fam102a	ENSMUSG00000039157	protein_coding	HITS-CLIP,Piranha_0.01	1
Foxk2	ENSMUSG00000039275	protein_coding	HITS-CLIP,Piranha_0.01	1
Fpr3	ENSMUSG00000079700	protein_coding	HITS-CLIP,Piranha_0.01	1
Frmd6	ENSMUSG00000048285	protein_coding	HITS-CLIP,Piranha_0.01	1
Fxr1	ENSMUSG00000027680	protein_coding	HITS-CLIP,Piranha_0.01	1

Galntl6	ENSMUSG00000096914	protein_coding	HITS-CLIP,Piranha_0.01	1
Gars	ENSMUSG00000029777	protein_coding	HITS-CLIP,Piranha_0.01	1
Gata5os	ENSMUSG00000056061	antisense	HITS-CLIP,Piranha_0.01	1
Gatad2b	ENSMUSG00000042390	protein_coding	HITS-CLIP,Piranha_0.01	1
Glipr2	ENSMUSG00000028480	protein_coding	HITS-CLIP,Piranha_0.01	1
Gm12381	ENSMUSG00000085632	lincRNA	HITS-CLIP,Piranha_0.01	1
Gm13219	ENSMUSG00000079604	lincRNA	HITS-CLIP,Piranha_0.01	1
Gm15472	ENSMUSG00000084760	sense_intronic	HITS-CLIP,Piranha_0.01	1
Gm17494	ENSMUSG00000057359	processed_transcri	HITS-CLIP,Piranha_0.01	1
Gm17767	ENSMUSG00000099413	lincRNA	HITS-CLIP,Piranha_0.01	1
Gm19500	ENSMUSG00000117864	processed_transcri	HITS-CLIP,Piranha_0.01	1
Gm19610	ENSMUSG00000107350	antisense	HITS-CLIP,Piranha_0.01	1
Gm2000	ENSMUSG00000078193	protein_coding	HITS-CLIP,Piranha_0.01	1
Gm20275	ENSMUSG00000110803	antisense	HITS-CLIP,Piranha_0.01	1
Gm20489	ENSMUSG00000092463	protein_coding	HITS-CLIP,Piranha_0.01	1
Gm20671	ENSMUSG00000093574	protein_coding	HITS-CLIP,Piranha_0.01	1
Gm20716	ENSMUSG00000093752	protein_coding	HITS-CLIP,Piranha_0.01	1
Gm21985	ENSMUSG00000096764	protein_coding	HITS-CLIP,Piranha_0.01	1
Gm22027	ENSMUSG00000084686	snoRNA	HITS-CLIP,Piranha_0.01	1
Gm22053	ENSMUSG00000065328	snoRNA	HITS-CLIP,Piranha_0.01	1
Gm22106	ENSMUSG00000084493	snRNA	HITS-CLIP,Piranha_0.01	1
Gm22192	ENSMUSG00000094382	snoRNA	HITS-CLIP,Piranha_0.01	1
Gm22296	ENSMUSG00000065244	snRNA	HITS-CLIP,Piranha_0.01	1
Gm22303	ENSMUSG00000064620	snoRNA	HITS-CLIP,Piranha_0.01	1
Gm22305	ENSMUSG00000064624	snRNA	HITS-CLIP,Piranha_0.01	1
Gm22392	ENSMUSG00000094551	snRNA	HITS-CLIP,Piranha_0.01	1
Gm22422	ENSMUSG00000064994	snoRNA	HITS-CLIP,CTK	1
Gm22422	ENSMUSG00000064994	snoRNA	HITS-CLIP,Piranha_0.01	1
Gm22513	ENSMUSG00000096349	snRNA	HITS-CLIP,CTK	1
Gm22701	ENSMUSG00000093112	misc_RNA	HITS-CLIP,Piranha_0.01	1
Gm22740	ENSMUSG00000065373	snRNA	HITS-CLIP,Piranha_0.01	1
Gm22959	ENSMUSG00000084463	misc_RNA	HITS-CLIP,Piranha_0.01	1
Gm23095	ENSMUSG00000065380	snRNA	HITS-CLIP,CTK	1
Gm23123	ENSMUSG00000088273	snoRNA	HITS-CLIP,Piranha_0.01	1
Gm23212	ENSMUSG00000084667	snoRNA	HITS-CLIP,Piranha_0.01	1
Gm23241	ENSMUSG00000064948	snRNA	HITS-CLIP,Piranha_0.01	1
Gm23244	ENSMUSG00000065309	snRNA	HITS-CLIP,Piranha_0.01	1
Gm23246	ENSMUSG00000065305	snoRNA	HITS-CLIP,CTK	1
Gm23297	ENSMUSG00000065118	snoRNA	HITS-CLIP,Piranha_0.01	1
Gm23349	ENSMUSG00000089057	snRNA	HITS-CLIP,Piranha_0.01	1
Gm23451	ENSMUSG00000065262	snoRNA	HITS-CLIP,Piranha_0.01	1
Gm23502	ENSMUSG00000065836	snRNA	HITS-CLIP,Piranha_0.01	1
Gm23690	ENSMUSG00000064970	snRNA	HITS-CLIP,Piranha_0.01	1
Gm23755	ENSMUSG00000088203	misc_RNA	HITS-CLIP,Piranha_0.01	1
Gm23764	ENSMUSG00000088971	rRNA	HITS-CLIP,Piranha_0.01	1
Gm24136	ENSMUSG00000095513	snRNA	HITS-CLIP,CTK	1
Gm24146	ENSMUSG00000064694	misc_RNA	HITS-CLIP,Piranha_0.01	1
Gm24148	ENSMUSG00000064696	snoRNA	HITS-CLIP,Piranha_0.01	1
Gm24336	ENSMUSG00000077391	snoRNA	HITS-CLIP,Piranha_0.01	1
Gm24601	ENSMUSG00000088595	rRNA	HITS-CLIP,CTK	1
Gm24698	ENSMUSG00000065817	snoRNA	HITS-CLIP,Piranha_0.01	1
Gm24763	ENSMUSG00000065622	snRNA	HITS-CLIP,Piranha_0.01	1
Gm24859	ENSMUSG00000064663	snRNA	HITS-CLIP,Piranha_0.01	1
Gm24888	ENSMUSG00000077575	snoRNA	HITS-CLIP,CTK	1
Gm24968	ENSMUSG00000096043	snRNA	HITS-CLIP,Piranha_0.01	1
Gm25078	ENSMUSG00000064464	snRNA	HITS-CLIP,Piranha_0.01	1
Gm25106	ENSMUSG00000084621	snRNA	HITS-CLIP,Piranha_0.01	1
Gm25179	ENSMUSG00000084581	snRNA	HITS-CLIP,Piranha_0.01	1

Gm25296	ENSMUSG00000064500	snoRNA	HITS-CLIP,Piranha_0.01	1
Gm25343	ENSMUSG00000088085	snRNA	HITS-CLIP,Piranha_0.01	1
Gm25555	ENSMUSG00000088703	rRNA	HITS-CLIP,Piranha_0.01	1
Gm25636	ENSMUSG00000064600	snoRNA	HITS-CLIP,Piranha_0.01	1
Gm25681	ENSMUSG00000065872	snRNA	HITS-CLIP,Piranha_0.01	1
Gm25855	ENSMUSG00000064721	snoRNA	HITS-CLIP,CTK	1
Gm26020	ENSMUSG00000084678	snRNA	HITS-CLIP,Piranha_0.01	1
Gm26206	ENSMUSG00000064842	snRNA	HITS-CLIP,Piranha_0.01	1
Gm26316	ENSMUSG00000065820	snRNA	HITS-CLIP,Piranha_0.01	1
Gm26373	ENSMUSG00000088458	snRNA	HITS-CLIP,Piranha_0.01	1
Gm26435	ENSMUSG00000096018	snRNA	HITS-CLIP,Piranha_0.01	1
Gm26754	ENSMUSG00000114616	lincRNA	HITS-CLIP,Piranha_0.01	1
Gm27533	ENSMUSG00000098543	misc_RNA	HITS-CLIP,Piranha_0.01	1
Gm29017	ENSMUSG00000101586	antisense	HITS-CLIP,Piranha_0.01	1
Gm29040	ENSMUSG00000099576	lincRNA	HITS-CLIP,Piranha_0.01	1
Gm31121	ENSMUSG00000104785	lincRNA	HITS-CLIP,Piranha_0.01	1
Gm31819	ENSMUSG00000117984	lincRNA	HITS-CLIP,Piranha_0.01	1
Gm32027	ENSMUSG00000117990	processed_transcri	HITS-CLIP,Piranha_0.01	1
Gm32647	ENSMUSG00000108532	lincRNA	HITS-CLIP,Piranha_0.01	1
Gm32850	ENSMUSG00000108868	lincRNA	HITS-CLIP,Piranha_0.01	1
Gm34623	ENSMUSG00000109966	lincRNA	HITS-CLIP,Piranha_0.01	1
Gm34678	ENSMUSG00000115284	antisense	HITS-CLIP,Piranha_0.01	1
Gm35438	ENSMUSG00000118209	lincRNA	HITS-CLIP,Piranha_0.01	1
Gm36279	ENSMUSG00000117393	antisense	HITS-CLIP,Piranha_0.01	1
Gm36931	ENSMUSG00000103779	TEC	HITS-CLIP,Piranha_0.01	1
Gm36933	ENSMUSG00000103772	TEC	HITS-CLIP,Piranha_0.01	1
Gm37033	ENSMUSG00000104388	TEC	HITS-CLIP,Piranha_0.01	1
Gm37084	ENSMUSG00000102856	TEC	HITS-CLIP,Piranha_0.01	1
Gm37186	ENSMUSG00000103937	TEC	HITS-CLIP,Piranha_0.01	1
Gm37312	ENSMUSG00000104099	TEC	HITS-CLIP,Piranha_0.01	1
Gm37645	ENSMUSG00000103192	TEC	HITS-CLIP,Piranha_0.01	1
Gm38034	ENSMUSG00000102844	TEC	HITS-CLIP,Piranha_0.01	1
Gm38062	ENSMUSG00000104488	TEC	HITS-CLIP,Piranha_0.01	1
Gm38228	ENSMUSG00000103945	TEC	HITS-CLIP,Piranha_0.01	1
Gm38394	ENSMUSG00000094410	protein_coding	HITS-CLIP,Piranha_0.01	1
Gm39232	ENSMUSG00000109769	lincRNA	HITS-CLIP,Piranha_0.01	1
Gm41724	ENSMUSG00000118365	antisense	HITS-CLIP,Piranha_0.01	1
Gm43029	ENSMUSG00000106904	TEC	HITS-CLIP,Piranha_0.01	1
Gm43055	ENSMUSG00000105704	lincRNA	HITS-CLIP,Piranha_0.01	1
Gm43065	ENSMUSG00000105986	sense_intronic	HITS-CLIP,Piranha_0.01	1
Gm43481	ENSMUSG00000107000	TEC	HITS-CLIP,Piranha_0.01	1
Gm43731	ENSMUSG00000104531	lincRNA	HITS-CLIP,Piranha_0.01	1
Gm43788	ENSMUSG00000107286	TEC	HITS-CLIP,Piranha_0.01	1
Gm43890	ENSMUSG00000108053	TEC	HITS-CLIP,Piranha_0.01	1
Gm43984	ENSMUSG00000108238	TEC	HITS-CLIP,Piranha_0.01	1
Gm44013	ENSMUSG00000107750	TEC	HITS-CLIP,Piranha_0.01	1
Gm45408	ENSMUSG00000110217	lincRNA	HITS-CLIP,Piranha_0.01	1
Gm45510	ENSMUSG00000109647	antisense	HITS-CLIP,Piranha_0.01	1
Gm47277	ENSMUSG00000104767	miRNA	HITS-CLIP,Piranha_0.01	1
Gm47284	ENSMUSG00000105232	miRNA	HITS-CLIP,CTK	1
Gm47295	ENSMUSG00000105788	miRNA	HITS-CLIP,Piranha_0.01	1
Gm47348	ENSMUSG00000114512	lincRNA	HITS-CLIP,Piranha_0.01	1
Gm47694	ENSMUSG00000114584	TEC	HITS-CLIP,Piranha_0.01	1
Gm47916	ENSMUSG00000113016	lincRNA	HITS-CLIP,Piranha_0.01	1
Gm48606	ENSMUSG00000113586	lincRNA	HITS-CLIP,Piranha_0.01	1
Gm49180	ENSMUSG00000115735	lincRNA	HITS-CLIP,Piranha_0.01	1
Gm49336	ENSMUSG00000114797	protein_coding	HITS-CLIP,Piranha_0.01	1
Gm49346	ENSMUSG00000104599	miRNA	HITS-CLIP,Piranha_0.01	1

Gm49347	ENSMUSG00000104863	miRNA	HITS-CLIP,Piranha_0.01	1
Gm49759	ENSMUSG00000111394	TEC	HITS-CLIP,Piranha_0.01	1
Gm8857	ENSMUSG00000091933	protein_coding	HITS-CLIP,Piranha_0.01	1
Gm8871	ENSMUSG00000095710	protein_coding	HITS-CLIP,Piranha_0.01	1
Gmip	ENSMUSG00000036246	protein_coding	HITS-CLIP,Piranha_0.01	1
Gna13	ENSMUSG00000020611	protein_coding	HITS-CLIP,Piranha_0.01	1
Gnl2	ENSMUSG00000028869	protein_coding	HITS-CLIP,Piranha_0.01	1
Gnl3	ENSMUSG00000042354	protein_coding	HITS-CLIP,Piranha_0.01	1
Golt1b	ENSMUSG00000030245	protein_coding	HITS-CLIP,Piranha_0.01	1
Gosr1	ENSMUSG00000010392	protein_coding	HITS-CLIP,Piranha_0.01	1
Got1	ENSMUSG00000025190	protein_coding	HITS-CLIP,Piranha_0.01	1
Gpr18	ENSMUSG00000050350	protein_coding	HITS-CLIP,Piranha_0.01	1
Gpr183	ENSMUSG00000051212	protein_coding	HITS-CLIP,Piranha_0.01	1
Gpr65	ENSMUSG00000021886	protein_coding	HITS-CLIP,Piranha_0.01	1
Gtf3c3	ENSMUSG00000041303	protein_coding	HITS-CLIP,Piranha_0.01	1
H1f3	ENSMUSG00000052565	protein_coding	HITS-CLIP,Piranha_0.01	1
H2ac4	ENSMUSG00000061615	protein_coding	HITS-CLIP,Piranha_0.01	1
H2ac8	ENSMUSG00000069272	protein_coding	HITS-CLIP,Piranha_0.01	1
H2bc12	ENSMUSG00000062727	protein_coding	HITS-CLIP,Piranha_0.01	1
H2bc3	ENSMUSG00000075031	protein_coding	HITS-CLIP,Piranha_0.01	1
H3c11	ENSMUSG00000101972	protein_coding	HITS-CLIP,Piranha_0.01	1
H3c6	ENSMUSG00000069273	protein_coding	HITS-CLIP,Piranha_0.01	1
H3c7	ENSMUSG00000100210	protein_coding	HITS-CLIP,Piranha_0.01	1
H4c8	ENSMUSG00000060981	protein_coding	HITS-CLIP,Piranha_0.01	1
Hacd3	ENSMUSG00000033629	protein_coding	HITS-CLIP,Piranha_0.01	1
Hapln1	ENSMUSG00000021613	protein_coding	HITS-CLIP,Piranha_0.01	1
Hars	ENSMUSG00000001380	protein_coding	HITS-CLIP,Piranha_0.01	1
Hcn3	ENSMUSG00000028051	protein_coding	HITS-CLIP,Piranha_0.01	1
Hdac2	ENSMUSG00000019777	protein_coding	HITS-CLIP,Piranha_0.01	1
Hexim1	ENSMUSG00000048878	protein_coding	HITS-CLIP,Piranha_0.01	1
Hipk2	ENSMUSG00000061436	protein_coding	HITS-CLIP,Piranha_0.01	1
Hmgb1	ENSMUSG00000066551	protein_coding	HITS-CLIP,Piranha_0.01	1
Hmgb2	ENSMUSG00000054717	protein_coding	HITS-CLIP,Piranha_0.01	1
Hoxc5	ENSMUSG00000022485	protein_coding	HITS-CLIP,Piranha_0.01	1
Hsd17b12	ENSMUSG00000027195	protein_coding	HITS-CLIP,Piranha_0.01	1
Hsd17b7	ENSMUSG00000026675	protein_coding	HITS-CLIP,Piranha_0.01	1
Hsp90b1	ENSMUSG00000020048	protein_coding	HITS-CLIP,Piranha_0.01	1
Hspa14	ENSMUSG00000109865	protein_coding	HITS-CLIP,Piranha_0.01	1
Icam1	ENSMUSG00000037405	protein_coding	HITS-CLIP,Piranha_0.01	1
Igfbp4	ENSMUSG00000017493	protein_coding	HITS-CLIP,Piranha_0.01	1
Igfbpl1	ENSMUSG00000035551	protein_coding	HITS-CLIP,Piranha_0.01	1
Il23r	ENSMUSG00000049093	protein_coding	HITS-CLIP,Piranha_0.01	1
Il2rg	ENSMUSG00000031304	protein_coding	HITS-CLIP,Piranha_0.01	1
Ipo5	ENSMUSG00000030662	protein_coding	HITS-CLIP,Piranha_0.01	1
Itfg1	ENSMUSG00000031703	protein_coding	HITS-CLIP,Piranha_0.01	1
Itga5	ENSMUSG00000000555	protein_coding	HITS-CLIP,Piranha_0.01	1
Itm2b	ENSMUSG00000022108	protein_coding	HITS-CLIP,Piranha_0.01	1
Izumo1r	ENSMUSG00000031933	protein_coding	HITS-CLIP,Piranha_0.01	1
Kctd12	ENSMUSG00000098557	protein_coding	HITS-CLIP,Piranha_0.01	1
Khdc4	ENSMUSG00000028060	protein_coding	HITS-CLIP,Piranha_0.01	1
Kif1b	ENSMUSG00000063077	protein_coding	HITS-CLIP,Piranha_0.01	1
Kif5b	ENSMUSG00000006740	protein_coding	HITS-CLIP,Piranha_0.01	1
Kpna3	ENSMUSG00000021929	protein_coding	HITS-CLIP,Piranha_0.01	1
Kpnb1	ENSMUSG00000001440	protein_coding	HITS-CLIP,Piranha_0.01	1
Lag3	ENSMUSG00000030124	protein_coding	HITS-CLIP,Piranha_0.01	1
Lamp1	ENSMUSG00000031447	protein_coding	HITS-CLIP,Piranha_0.01	1
Lars	ENSMUSG00000024493	protein_coding	HITS-CLIP,Piranha_0.01	1
Lats1	ENSMUSG00000040021	protein_coding	HITS-CLIP,Piranha_0.01	1

Lbr	ENSMUSG00000004880	protein_coding	HITS-CLIP,Piranha_0.01	1
Lemd3	ENSMUSG00000048661	protein_coding	HITS-CLIP,Piranha_0.01	1
Lin7c	ENSMUSG00000027162	protein_coding	HITS-CLIP,Piranha_0.01	1
Lman2	ENSMUSG00000021484	protein_coding	HITS-CLIP,Piranha_0.01	1
Lrrc8c	ENSMUSG00000054720	protein_coding	HITS-CLIP,Piranha_0.01	1
Lsm8	ENSMUSG00000044155	protein_coding	HITS-CLIP,Piranha_0.01	1
Ly75	ENSMUSG00000026980	protein_coding	HITS-CLIP,Piranha_0.01	1
Lyst	ENSMUSG00000019726	protein_coding	HITS-CLIP,Piranha_0.01	1
Maf	ENSMUSG00000055435	protein_coding	HITS-CLIP,Piranha_0.01	1
Magohb	ENSMUSG00000030188	protein_coding	HITS-CLIP,Piranha_0.01	1
Malat1	ENSMUSG00000092341	lincRNA	HITS-CLIP,CTK	1
Malrd1	ENSMUSG00000075520	protein_coding	HITS-CLIP,Piranha_0.01	1
Maml3	ENSMUSG00000061143	protein_coding	HITS-CLIP,Piranha_0.01	1
Manbal	ENSMUSG00000063019	protein_coding	HITS-CLIP,Piranha_0.01	1
Marcksl1	ENSMUSG00000047945	protein_coding	HITS-CLIP,Piranha_0.01	1
Med14	ENSMUSG00000064127	protein_coding	HITS-CLIP,Piranha_0.01	1
Mep1b	ENSMUSG00000024313	protein_coding	HITS-CLIP,Piranha_0.01	1
Mex3c	ENSMUSG00000037253	protein_coding	HITS-CLIP,Piranha_0.01	1
Mir101b	ENSMUSG00000065556	miRNA	HITS-CLIP,Piranha_0.01	1
Mir140	ENSMUSG00000065439	miRNA	HITS-CLIP,Piranha_0.01	1
Mir146	ENSMUSG00000065601	miRNA	HITS-CLIP,Piranha_0.01	1
Mir183	ENSMUSG00000065619	miRNA	HITS-CLIP,Piranha_0.01	1
Mir22	ENSMUSG00000065529	miRNA	HITS-CLIP,Piranha_0.01	1
Mir24-1	ENSMUSG00000105904	miRNA	HITS-CLIP,Piranha_0.01	1
Mkrn2	ENSMUSG00000000439	protein_coding	HITS-CLIP,Piranha_0.01	1
Mlec	ENSMUSG00000048578	protein_coding	HITS-CLIP,Piranha_0.01	1
Mnd1	ENSMUSG00000033752	protein_coding	HITS-CLIP,Piranha_0.01	1
Mnt	ENSMUSG00000000282	protein_coding	HITS-CLIP,Piranha_0.01	1
Mphosph10	ENSMUSG00000030521	protein_coding	HITS-CLIP,Piranha_0.01	1
Msi2	ENSMUSG00000069769	protein_coding	HITS-CLIP,Piranha_0.01	1
Mta2	ENSMUSG00000071646	protein_coding	HITS-CLIP,Piranha_0.01	1
Mtfr2	ENSMUSG00000019992	protein_coding	HITS-CLIP,Piranha_0.01	1
Mtor	ENSMUSG00000028991	protein_coding	HITS-CLIP,Piranha_0.01	1
Mtpn	ENSMUSG00000029840	protein_coding	HITS-CLIP,Piranha_0.01	1
Mtrex	ENSMUSG00000016018	protein_coding	HITS-CLIP,Piranha_0.01	1
n-R5-8s1	ENSMUSG00000065922	rRNA	HITS-CLIP,CTK	1
n-R5s205	ENSMUSG00000065083	rRNA	HITS-CLIP,CTK	1
n-R5s90	ENSMUSG00000077156	rRNA	HITS-CLIP,Piranha_0.01	1
Naa25	ENSMUSG00000042719	protein_coding	HITS-CLIP,Piranha_0.01	1
Naca	ENSMUSG00000061315	protein_coding	HITS-CLIP,Piranha_0.01	1
Nacc1	ENSMUSG00000001910	protein_coding	HITS-CLIP,Piranha_0.01	1
Nap1l1	ENSMUSG00000058799	protein_coding	HITS-CLIP,Piranha_0.01	1
Napa	ENSMUSG00000006024	protein_coding	HITS-CLIP,Piranha_0.01	1
Ncdn	ENSMUSG00000028833	protein_coding	HITS-CLIP,Piranha_0.01	1
Nckap5l	ENSMUSG00000023009	protein_coding	HITS-CLIP,Piranha_0.01	1
Ndel1	ENSMUSG00000018736	protein_coding	HITS-CLIP,Piranha_0.01	1
Nfkbia	ENSMUSG00000021025	protein_coding	HITS-CLIP,CTK	1
Nfkbib	ENSMUSG00000030595	protein_coding	HITS-CLIP,Piranha_0.01	1
Nipsnap3b	ENSMUSG00000015247	protein_coding	HITS-CLIP,Piranha_0.01	1
Nktr	ENSMUSG00000032525	protein_coding	HITS-CLIP,Piranha_0.01	1
Nlk	ENSMUSG00000017376	protein_coding	HITS-CLIP,Piranha_0.01	1
Nomo1	ENSMUSG00000030835	protein_coding	HITS-CLIP,Piranha_0.01	1
Nop10	ENSMUSG00000027133	protein_coding	HITS-CLIP,Piranha_0.01	1
Nos2	ENSMUSG00000020826	protein_coding	HITS-CLIP,Piranha_0.01	1
Npat	ENSMUSG00000033054	protein_coding	HITS-CLIP,Piranha_0.01	1
Nucks1	ENSMUSG00000026434	protein_coding	HITS-CLIP,Piranha_0.01	1
Nup153	ENSMUSG00000021374	protein_coding	HITS-CLIP,Piranha_0.01	1
Nup160	ENSMUSG00000051329	protein_coding	HITS-CLIP,Piranha_0.01	1

Nup188	ENSMUSG00000052533	protein_coding	HITS-CLIP,Piranha_0.01	1
Nxpe3	ENSMUSG00000075033	protein_coding	HITS-CLIP,Piranha_0.01	1
Olfr483	ENSMUSG00000078118	protein_coding	HITS-CLIP,Piranha_0.01	1
Olfr484	ENSMUSG00000110171	protein_coding	HITS-CLIP,Piranha_0.01	1
Olfr743	ENSMUSG00000094285	protein_coding	HITS-CLIP,Piranha_0.01	1
Osbp	ENSMUSG00000024687	protein_coding	HITS-CLIP,Piranha_0.01	1
Osm	ENSMUSG00000058755	protein_coding	HITS-CLIP,Piranha_0.01	1
Pappa	ENSMUSG00000028370	protein_coding	HITS-CLIP,Piranha_0.01	1
Pdlim1	ENSMUSG00000055044	protein_coding	HITS-CLIP,Piranha_0.01	1
Phactr2	ENSMUSG00000062866	protein_coding	HITS-CLIP,Piranha_0.01	1
Pias4	ENSMUSG00000004934	protein_coding	HITS-CLIP,Piranha_0.01	1
Pik3ca	ENSMUSG00000027665	protein_coding	HITS-CLIP,Piranha_0.01	1
Pik3r1	ENSMUSG00000041417	protein_coding	HITS-CLIP,Piranha_0.01	1
Plac8	ENSMUSG00000029322	protein_coding	HITS-CLIP,Piranha_0.01	1
Plekhb2	ENSMUSG00000026123	protein_coding	HITS-CLIP,Piranha_0.01	1
Pofut2	ENSMUSG00000020260	protein_coding	HITS-CLIP,Piranha_0.01	1
Ppp3r2	ENSMUSG00000028310	protein_coding	HITS-CLIP,Piranha_0.01	1
Ppp4r2	ENSMUSG00000052144	protein_coding	HITS-CLIP,Piranha_0.01	1
Prpf4b	ENSMUSG00000021413	protein_coding	HITS-CLIP,Piranha_0.01	1
Prps2	ENSMUSG00000025742	protein_coding	HITS-CLIP,Piranha_0.01	1
Prr14	ENSMUSG00000030822	protein_coding	HITS-CLIP,Piranha_0.01	1
Psd3	ENSMUSG00000030465	protein_coding	HITS-CLIP,Piranha_0.01	1
Ptges3	ENSMUSG00000071072	protein_coding	HITS-CLIP,Piranha_0.01	1
Ptpn1	ENSMUSG00000027540	protein_coding	HITS-CLIP,Piranha_0.01	1
Rab11fip4	ENSMUSG00000017639	protein_coding	HITS-CLIP,Piranha_0.01	1
Rab18	ENSMUSG00000073639	protein_coding	HITS-CLIP,Piranha_0.01	1
Rab21	ENSMUSG00000020132	protein_coding	HITS-CLIP,Piranha_0.01	1
Rad21	ENSMUSG00000022314	protein_coding	HITS-CLIP,Piranha_0.01	1
Rad23b	ENSMUSG00000028426	protein_coding	HITS-CLIP,Piranha_0.01	1
Rad51	ENSMUSG00000027323	protein_coding	HITS-CLIP,Piranha_0.01	1
Rap2b	ENSMUSG00000036894	protein_coding	HITS-CLIP,Piranha_0.01	1
Raver1	ENSMUSG00000010205	protein_coding	HITS-CLIP,Piranha_0.01	1
Rbbp4	ENSMUSG00000057236	protein_coding	HITS-CLIP,Piranha_0.01	1
Rbm33	ENSMUSG00000048271	protein_coding	HITS-CLIP,Piranha_0.01	1
Rbm45	ENSMUSG00000042369	protein_coding	HITS-CLIP,Piranha_0.01	1
Rcn2	ENSMUSG00000032320	protein_coding	HITS-CLIP,Piranha_0.01	1
Rcor1	ENSMUSG00000037896	protein_coding	HITS-CLIP,Piranha_0.01	1
Rdh10	ENSMUSG00000025921	protein_coding	HITS-CLIP,Piranha_0.01	1
Rn7s1	ENSMUSG00000099021	misc_RNA	HITS-CLIP,Piranha_0.01	1
Rnf11	ENSMUSG00000028557	protein_coding	HITS-CLIP,Piranha_0.01	1
Rnf126	ENSMUSG00000035890	protein_coding	HITS-CLIP,Piranha_0.01	1
Rnu11	ENSMUSG00000077323	snRNA	HITS-CLIP,CTK	1
Rock1	ENSMUSG00000024290	protein_coding	HITS-CLIP,Piranha_0.01	1
Rpl13	ENSMUSG00000000740	protein_coding	HITS-CLIP,Piranha_0.01	1
Rplp1	ENSMUSG00000007892	protein_coding	HITS-CLIP,Piranha_0.01	1
Rps20	ENSMUSG00000028234	protein_coding	HITS-CLIP,Piranha_0.01	1
Rrn3	ENSMUSG00000022682	protein_coding	HITS-CLIP,Piranha_0.01	1
Rsl24d1	ENSMUSG00000032215	protein_coding	HITS-CLIP,Piranha_0.01	1
Rsph4a	ENSMUSG00000039552	protein_coding	HITS-CLIP,Piranha_0.01	1
Samsn1	ENSMUSG00000022876	protein_coding	HITS-CLIP,Piranha_0.01	1
Sde2	ENSMUSG00000038806	protein_coding	HITS-CLIP,Piranha_0.01	1
Sem1	ENSMUSG00000042541	protein_coding	HITS-CLIP,Piranha_0.01	1
Sf3b6	ENSMUSG00000037361	protein_coding	HITS-CLIP,Piranha_0.01	1
Sfmbt1	ENSMUSG00000006527	protein_coding	HITS-CLIP,Piranha_0.01	1
Sgcd	ENSMUSG00000020354	protein_coding	HITS-CLIP,Piranha_0.01	1
Sik1	ENSMUSG00000024042	protein_coding	HITS-CLIP,CTK	1
Slc1a6	ENSMUSG00000005357	protein_coding	HITS-CLIP,Piranha_0.01	1
Slc25a36	ENSMUSG00000032449	protein_coding	HITS-CLIP,Piranha_0.01	1

Slc25a4	ENSMUSG00000031633	protein_coding	HITS-CLIP,Piranha_0.01	1
Slc26a2	ENSMUSG00000034320	protein_coding	HITS-CLIP,Piranha_0.01	1
Slc30a7	ENSMUSG00000054414	protein_coding	HITS-CLIP,Piranha_0.01	1
Slc35e1	ENSMUSG00000019731	protein_coding	HITS-CLIP,Piranha_0.01	1
Slc7a11	ENSMUSG00000027737	protein_coding	HITS-CLIP,Piranha_0.01	1
Slf1	ENSMUSG00000021597	protein_coding	HITS-CLIP,Piranha_0.01	1
Smg1	ENSMUSG00000030655	protein_coding	HITS-CLIP,Piranha_0.01	1
Smim31	ENSMUSG00000074300	protein_coding	HITS-CLIP,Piranha_0.01	1
Snapc1l	ENSMUSG00000114046	protein_coding	HITS-CLIP,Piranha_0.01	1
Snora24	ENSMUSG00000092730	snoRNA	HITS-CLIP,CTK	1
Snora24	ENSMUSG00000092730	snoRNA	HITS-CLIP,Piranha_0.01	1
Snora7a	ENSMUSG00000064563	snoRNA	HITS-CLIP,Piranha_0.01	1
Snord15b	ENSMUSG00000064966	snoRNA	HITS-CLIP,Piranha_0.01	1
Snord19	ENSMUSG00000077797	snoRNA	HITS-CLIP,Piranha_0.01	1
Snord38a	ENSMUSG00000065680	snoRNA	HITS-CLIP,CTK	1
Snord61	ENSMUSG00000065110	snoRNA	HITS-CLIP,Piranha_0.01	1
Snord78	ENSMUSG00000077220	snoRNA	HITS-CLIP,Piranha_0.01	1
Snord88c	ENSMUSG00000096296	snoRNA	HITS-CLIP,Piranha_0.01	1
Sod2	ENSMUSG00000006818	protein_coding	HITS-CLIP,Piranha_0.01	1
Spopl	ENSMUSG00000026771	protein_coding	HITS-CLIP,Piranha_0.01	1
Srd5a2	ENSMUSG00000038541	protein_coding	HITS-CLIP,Piranha_0.01	1
Srm	ENSMUSG00000006442	protein_coding	HITS-CLIP,Piranha_0.01	1
Ssh2	ENSMUSG00000037926	protein_coding	HITS-CLIP,Piranha_0.01	1
Strap	ENSMUSG00000030224	protein_coding	HITS-CLIP,Piranha_0.01	1
Suco	ENSMUSG00000040297	protein_coding	HITS-CLIP,Piranha_0.01	1
Supt6	ENSMUSG00000002052	protein_coding	HITS-CLIP,Piranha_0.01	1
Surf6	ENSMUSG00000036160	protein_coding	HITS-CLIP,Piranha_0.01	1
Svet1	ENSMUSG00000109648	TEC	HITS-CLIP,Piranha_0.01	1
Taf2	ENSMUSG00000037343	protein_coding	HITS-CLIP,Piranha_0.01	1
Tat	ENSMUSG00000001670	protein_coding	HITS-CLIP,Piranha_0.01	1
Tespa1	ENSMUSG00000034833	protein_coding	HITS-CLIP,Piranha_0.01	1
Tigar	ENSMUSG00000038028	protein_coding	HITS-CLIP,Piranha_0.01	1
Timm8b	ENSMUSG00000039016	protein_coding	HITS-CLIP,Piranha_0.01	1
Tiparp	ENSMUSG00000034640	protein_coding	HITS-CLIP,Piranha_0.01	1
Tmem185b	ENSMUSG00000098923	protein_coding	HITS-CLIP,Piranha_0.01	1
Tmem242	ENSMUSG00000004945	protein_coding	HITS-CLIP,Piranha_0.01	1
Tmem50a	ENSMUSG00000028822	protein_coding	HITS-CLIP,Piranha_0.01	1
Tmem64	ENSMUSG00000043252	protein_coding	HITS-CLIP,Piranha_0.01	1
Tmx1	ENSMUSG00000021072	protein_coding	HITS-CLIP,Piranha_0.01	1
Tmx3	ENSMUSG00000024614	protein_coding	HITS-CLIP,Piranha_0.01	1
Tnfrsf13b	ENSMUSG00000010142	protein_coding	HITS-CLIP,Piranha_0.01	1
Tnfrsf4	ENSMUSG00000029075	protein_coding	HITS-CLIP,Piranha_0.01	1
Tnfsf14	ENSMUSG00000005824	protein_coding	HITS-CLIP,Piranha_0.01	1
Tnfsf8	ENSMUSG00000028362	protein_coding	HITS-CLIP,CTK	1
Tob1	ENSMUSG00000037573	protein_coding	HITS-CLIP,Piranha_0.01	1
Tprgl	ENSMUSG00000029030	protein_coding	HITS-CLIP,Piranha_0.01	1
Trabd2b	ENSMUSG00000070867	protein_coding	HITS-CLIP,Piranha_0.01	1
Trim37	ENSMUSG00000018548	protein_coding	HITS-CLIP,Piranha_0.01	1
Trim58	ENSMUSG00000037124	protein_coding	HITS-CLIP,Piranha_0.01	1
Tub	ENSMUSG00000031028	protein_coding	HITS-CLIP,Piranha_0.01	1
Twistnb	ENSMUSG00000020561	protein_coding	HITS-CLIP,Piranha_0.01	1
Twnk	ENSMUSG00000025209	protein_coding	HITS-CLIP,Piranha_0.01	1
Ubb	ENSMUSG00000019505	protein_coding	HITS-CLIP,Piranha_0.01	1
Ube2q2	ENSMUSG00000032307	protein_coding	HITS-CLIP,Piranha_0.01	1
Ubr5	ENSMUSG00000037487	protein_coding	HITS-CLIP,Piranha_0.01	1
Ucp2	ENSMUSG00000033685	protein_coding	HITS-CLIP,Piranha_0.01	1
Usp38	ENSMUSG00000038250	protein_coding	HITS-CLIP,Piranha_0.01	1
Vdac1	ENSMUSG00000020402	protein_coding	HITS-CLIP,Piranha_0.01	1

Vmn2r2	ENSMUSG00000043897	protein_coding	HITS-CLIP,Piranha_0.01	1
Wdpcp	ENSMUSG00000020319	protein_coding	HITS-CLIP,Piranha_0.01	1
Xpo5	ENSMUSG00000067150	protein_coding	HITS-CLIP,Piranha_0.01	1
Yme1l1	ENSMUSG00000026775	protein_coding	HITS-CLIP,Piranha_0.01	1
Ythdf2	ENSMUSG00000040025	protein_coding	HITS-CLIP,Piranha_0.01	1
Yy1	ENSMUSG00000021264	protein_coding	HITS-CLIP,Piranha_0.01	1
Zbtb49	ENSMUSG00000029127	protein_coding	HITS-CLIP,Piranha_0.01	1
Zc3h12a	ENSMUSG00000042677	protein_coding	HITS-CLIP,Piranha_0.01	1
Zdhhc13	ENSMUSG00000030471	protein_coding	HITS-CLIP,Piranha_0.01	1
Zdhhc18	ENSMUSG00000037553	protein_coding	HITS-CLIP,Piranha_0.01	1
Zfp113	ENSMUSG00000037007	protein_coding	HITS-CLIP,Piranha_0.01	1
Zfp365	ENSMUSG00000037855	protein_coding	HITS-CLIP,Piranha_0.01	1
Zfp382	ENSMUSG00000074220	protein_coding	HITS-CLIP,Piranha_0.01	1
Zfp445	ENSMUSG00000047036	protein_coding	HITS-CLIP,Piranha_0.01	1
Zfp451	ENSMUSG00000042197	protein_coding	HITS-CLIP,Piranha_0.01	1
Zfp84	ENSMUSG00000046185	protein_coding	HITS-CLIP,Piranha_0.01	1
Zfp871	ENSMUSG00000024298	protein_coding	HITS-CLIP,Piranha_0.01	1
Zmiz1	ENSMUSG00000007817	protein_coding	HITS-CLIP,Piranha_0.01	1
Zmym2	ENSMUSG00000021945	protein_coding	HITS-CLIP,Piranha_0.01	1
Zmynd19	ENSMUSG00000026974	protein_coding	HITS-CLIP,Piranha_0.01	1
