# Supplementary material for: Tristetraprolin overexpression drives hematopoietic changes in young and middle-aged mice generating dominant mitigating effects on induced inflammation in murine models
Source: GeroScience. 2023 Aug 3;46(1):1271–84. doi: 10.1007/s11357-023-00879-2 (PMC10828162; doi:10.1007/s11357-023-00879-2)
Supplement: Supplementary file 5 — Supplementary file5 (PDF 72 KB) [file 11357_2023_879_MOESM5_ESM.pdf]

Supplemental Table 4: Overlap with HELA expression change

	HELA		Young HSC		Mid HSC		Young MPP	
Symbol	logFC	PValue	logFC	PValue	logFC	PValue	logFC	PValue
UBD	7.7788	0.0000	-1.7965	0.0000				
APLNR	-3.7589	0.0013	-1.7502	0.0000				
SCARA5	-0.7437	0.0001	-1.6096	0.0000				
LRRC32	-1.5542	0.0001	-1.5567	0.0000				
DISP2	0.7866	0.0001	-1.5046	0.0000				
DNAH6	1.5701	0.0006	-1.4962	0.0000				
SOX17	1.6173	0.0002	-1.4300	0.0000				
FRMD5	3.6136	0.0002	-1.3727	0.0000				
ABCA12	3.2141	0.0000	-1.3557	0.0001				
SVOPL	-2.4704	0.0005	-1.2484	0.0000				
NAV3	0.9226	0.0005	-1.2099	0.0000				
TFPI	5.9652	0.0001	-1.1680	0.0002				
GRIN2B	5.4750	0.0012	-1.1140	0.0003				
CREB5	1.7843	0.0004	-1.0986	0.0000	1.1633	0.0014		
PDE7B	-0.8667	0.0002	-1.0880	0.0000				
PDGFRL	2.2008	0.0000	-1.0482	0.0001				
CELF3	6.0477	0.0000	-1.0307	0.0002				
LONRF2	-1.5513	0.0002	-1.0133	0.0001				
KCNMA1	-5.5054	0.0012	-1.0105	0.0004				
ID3	0.6238	0.0006	-0.9617	0.0030				
USP29	2.4722	0.0006	-0.9515	0.0027				
TMCC3	0.8458	0.0003	-0.9376	0.0000				
VEPH1	1.4319	0.0000	-0.8905	0.0001				
DLGAP1	5.6931	0.0003	-0.8804	0.0006				
IL1R1	0.6981	0.0008	-0.7768	0.0000	-0.4794	0.0011	-0.9005	0.0001
EXOC3L2	3.5075	0.0007	-0.6652	0.0040			-1.0022	0.0007
GARNL3	-0.7769	0.0002	-0.6472	0.0036				
IGSF10	-1.9607	0.0005	-0.5777	0.0010	-0.6845	0.0000	-1.1367	0.0000
DENND2C	0.8230	0.0004	-0.5310	0.0046				
PRUNE2	2.4372	0.0001	-0.4906	0.0052				
N4BP2L1	5.7897	0.0002	0.4378	0.0030				
SCN3B	2.1556	0.0000	0.7544	0.0035				
ID2	1.0350	0.0000	0.9074	0.0000			3.5687	0.0000
ZFP36	6.0136	0.0000	0.9802	0.0000	1.0067	0.0000	1.7750	0.0000
CCL5	1.4634	0.0000	1.5177	0.0000			1.0247	0.0022
SPHK1	0.5859	0.0011	1.7760	0.0039				
KLF1	-6.4847	0.0000	1.7814	0.0000			1.3766	0.0014
CAMK2B	3.2641	0.0013					-0.9308	0.0002
PRX	-0.7600	0.0001					-0.8675	0.0001
SPEF1	1.1967	0.0004					-0.8065	0.0014
GBP2	0.8873	0.0006					0.6595	0.0003
ISG15	0.9998	0.0000					0.7366	0.0008
PRKCB	-2.0736	0.0000					0.9099	0.0002
MAP7D2	1.8025	0.0003					0.9406	0.0012
IRF5	0.9616	0.0012					1.0421	0.0000
KIF5A	2.4115	0.0001					1.1024	0.0000
IFIT3	1.4269	0.0000					1.1064	0.0000
STXBP6	0.9299	0.0001			1.2637	0.0000	1.1941	0.0000
TSPAN33	1.4506	0.0000					1.3201	0.0000

TRAF1	0.7134	0.0001			1.5998	0.0000
MX1	1.5287	0.0000			1.9545	0.0000
ATP1A3	0.8517	0.0005			1.9910	0.0000
SLAMF7	3.3785	0.0000			2.5703	0.0000
FCMR	0.9134	0.0011			2.9732	0.0000
CD74	3.6383	0.0000			3.1490	0.0000
ADAM19	-1.9091	0.0003			3.3973	0.0000
RASGRP1	4.1091	0.0000			3.5017	0.0000
RTN1	5.5797	0.0012			3.5653	0.0000
PID1	1.1717	0.0000			3.5822	0.0000
GRAMD2	1.8214	0.0000			4.3806	0.0000
OAS2	1.9127	0.0010	-1.0057	0.0005		
GLIPR2	0.8635	0.0010	0.4197	0.0013		
GAB3	3.6684	0.0002	0.6734	0.0003		
RPS6KL1	1.3566	0.0007	0.7497	0.0017		
NSUN7	5.7027	0.0003	0.7837	0.0003		
FLRT3	1.7948	0.0000	0.8410	0.0003		
SIDT1	5.3707	0.0012	1.1166	0.0000		
NDRG4	-1.2519	0.0006	1.2580	0.0000		
TMC1	5.7897	0.0002	2.0320	0.0000		
